# ATF5‐Dependent GDF15 Expression Mediates Anesthesia‐Induced Neuroprotection Against Stroke

**DOI:** 10.1002/advs.202417086

**Published:** 2025-11-26

**Authors:** Xianshu Ju, Tao Zhang, Jianchen Cui, Yulim Lee, Suho Lee, Ho Min Kim, Boohwi Hong, Jiho Park, Chul Hee Choi, Hyon‐Seung Yi, Jun Young Heo, Woosuk Chung

**Affiliations:** ^1^ Department of Medical Science Chungnam National University School of Medicine Daejeon 35015 South Korea; ^2^ Department of Biochemistry Chungnam National University School of Medicine Daejeon 35015 South Korea; ^3^ System Network Inflammation Control Research Center Chungnam National University School of Medicine Daejeon 35015 South Korea; ^4^ Department of Sleep Medicine The First People's Hospital of Yunnan Province. The Affiliated Hospital of Kunming University of Science and Technology Kunming 650032 China; ^5^ Center for Synaptic Brain Dysfunctions Institute for Basic Science (IBS) Daejeon 34126 South Korea; ^6^ Department of Biological Sciences Korea Advanced Institute of Science and Technology (KAIST) Daejeon 34141 South Korea; ^7^ Department of Anesthesiology and Pain Medicine Chungnam National University Hospital Chungnam National University School of Medicine Daejeon 35015 South Korea; ^8^ Department of Microbiology Chungnam National University School of Medicine Daejeon 35015 South Korea; ^9^ Department of Internal Medicine Chungnam National University Hospital Chungnam National University School of Medicine Daejeon 35015 South Korea

**Keywords:** anesthesia, ATF5 / GDF15, preconditioning, stroke

## Abstract

Perioperative stroke is a rare but serious complication with a rising incidence in aging populations. Although preclinical studies consistently demonstrate that anesthetics such as sevoflurane can induce neuroprotective preconditioning against ischemic injury, clinical results have remained inconclusive. In this study, it is demonstrated that sevoflurane‐induced neuroprotection is associated with the upregulation of genes involved in the mitochondrial unfolded protein response (UPR^mt^) and mitochondrial bioenergetic metabolism. The findings emphasize the critical role of ATF5 (activating transcription factor‐5) in mediating these protective effects. Sevoflurane preconditioning markedly increases ATF5 expression and its downstream target GDF15, a key regulator of mitochondrial homeostasis, in the cerebral cortex. However, this protective mechanism is not activated in the aged brain, suggesting that aging impairs the ability to mount a mitochondrial stress response. The results imply a need for age‐specific strategies to reduce perioperative stroke risk, including approaches that target mitochondrial function in elderly patients.

## Graphical abstract

Anesthesia‐induced preconditioning protects against perioperative stroke. By manipulating ATF5 in excitatory neurons, it is shown that anesthesia‐driven ATF5 upregulation activates the mitochondrial unfolded protein response (UPR^mt^) and elevates GDF15 expression. Aging disrupts this ATF5–GDF15–UPR^mt^ axis, offering a molecular explanation for the diminished efficacy of anesthetic preconditioning in older brains.

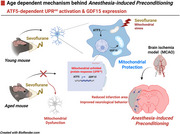

## 1 Introduction

Perioperative stroke, a term applied to strokes that occur during or within 30 days of surgery, is a relatively rare but devastating perioperative complication.^[^ ^1^ ^]^ Although uncommon (0.03–4%) after surgeries other than cardiac surgery and neurosurgery,^[^ ^2^ ^]^ the incidence of perioperative stroke is increasing in aging societies.^[^ ^3^, ^4^, ^5^ ^]^ Studies have reported significantly worse prognosis after perioperative ischemic strokes compared with community‐onset strokes, as reflected in increased 30‐day mortality, longer hospital stays, increased long‐term cognitive impairment, and disability.^[^ ^1^ ^]^ Recently, the significance of covert perioperative strokes (silent brain infarcts, strokes detectable only by imaging) has also been recognized.^[^ ^1^ ^]^ Not only is the incidence of covert strokes much higher (7%), but these strokes are also associated with increased risk of postoperative delirium, cognitive decline, and additional cerebrovascular insults 1 year after surgery.^[^ ^6^ ^]^ Considering that 310 million individuals worldwide are estimated to undergo surgical procedures annually,^[^ ^7^ ^]^ it is imperative that treatment measures that might reduce the negative impacts of perioperative stroke be identified.

One such potential strategy is anesthetic preconditioning (APC), an approach based on the observation that exposure to volatile anesthetic agents causes a transient increase in tolerance against ischemia/reperfusion injury.^[^ ^8^, ^9^ ^]^ Preclinical studies have demonstrated that exposure to volatile anesthetics before an ischemic event provides neuroprotection.^[^ ^10^, ^11^, ^12^, ^13^ ^]^ They have also shown that sevoflurane, currently the most widely used volatile anesthetic agent, can reduce neurological severity, cerebral infarct size, and cerebral edema.^[^ ^14^ ^]^ Unfortunately, despite promising findings in preclinical studies, clinical trials have yielded inconsistent results.^[^ ^15^, ^16^ ^]^ However, the consistent neuroprotective effects observed in preclinical studies are sufficiently compelling to suggest that understanding the mechanism underlying sevoflurane‐induced preconditioning in animals might provide important insights that could help explain the variability seen in clinical studies and identify therapeutic targets for reducing perioperative stroke risk.

Mitochondria dysfunction, a hallmark of ischemia/reperfusion injury, leads to neuronal death, highlighting the importance of preserving mitochondrial function for neurological recovery.^[^ ^17^, ^18^ ^]^ Interestingly, mitochondria are also integrally involved in anesthesia‐induced preconditioning.^[^ ^10^, ^19^, ^20^, ^21^, ^22^, ^23^ ^]^ Although the mechanisms underlying mitochondrial protection are not fully understood, recent studies suggest involvement of the mitochondrial unfolded protein response (UPR^mt^).^[^ ^21^, ^22^, ^24^ ^]^ UPR^mt^, a component of the integrated stress response, is a transcriptional response that serves to restore mitochondrial function and cellular homeostasis.^[^ ^25^, ^26^, ^27^ ^]^ A previous study showed that ischemia‐induced preconditioning fails to develop in cardiac tissue of transgenic mice lacking the transcriptional factor ATF5 (activating transcription factor‐5), a major regulator of UPR^mt^ in mammalian cells.^[^ ^21^ ^]^ A more recent study demonstrated that pharmacological treatment with meclizine, which modulates mitochondrial respiration and reduces oxidative stress through ATF5‐dependent UPR^mt^, provides neuroprotection against ischemia/reperfusion injury.^[^ ^24^ ^]^ Given that sevoflurane exposure also increases the expression of ATF5,^[^ ^28^ ^]^ it is reasonable to infer that ATF5‐dependent UPR^mt^ is directly involved in sevoflurane‐induced preconditioning in the brain.

To confirm the significance of ATF5‐dependent UPR^mt^ in sevoflurane‐induced preconditioning, we first exposed transgenic mice lacking ATF5 specifically in cortical excitatory neurons (*Emx1^cre/+^*; *ATF5* ^fl/fl^ mice) to sevoflurane, followed by middle cerebral artery occlusion (MCAO). After confirming the absence of sevoflurane‐induced preconditioning in these mice, we next investigated the expression of genes related to mitochondria energy metabolism and UPR^mt^ after sevoflurane exposure to identify downstream mediators of ATF5. Interestingly, we found a significant increase in GDF15 (growth differentiation factor‐15), a representative mitokine known to regulate energy metabolism in response to ischemia/reperfusion injury in the heart.^[^ ^27^, ^29^, ^30^, ^31^, ^32^ ^]^ By overexpressing ATF5 specifically in excitatory neurons and studying *Gdf15* whole‐body knockout (KO) mice, we provide direct evidence that sevoflurane exposure induces ATF5‐dependent release of GDF15 in excitatory neurons, resulting in increased mitochondrial function and resistance to ischemic/reperfusion injury. Importantly, we also found that sevoflurane‐induced activation of the ATF5–GDF15 signaling pathway is absent in aged mice, providing a plausible explanation for the inconsistencies in anesthesia‐induced preconditioning observed between preclinical and clinical studies.

## 2 Results

### 2.1 Anesthesia‐Induced Preconditioning Requires Exposure to 2.5% Sevoflurane for At Least 2 h

**Figure 1:**
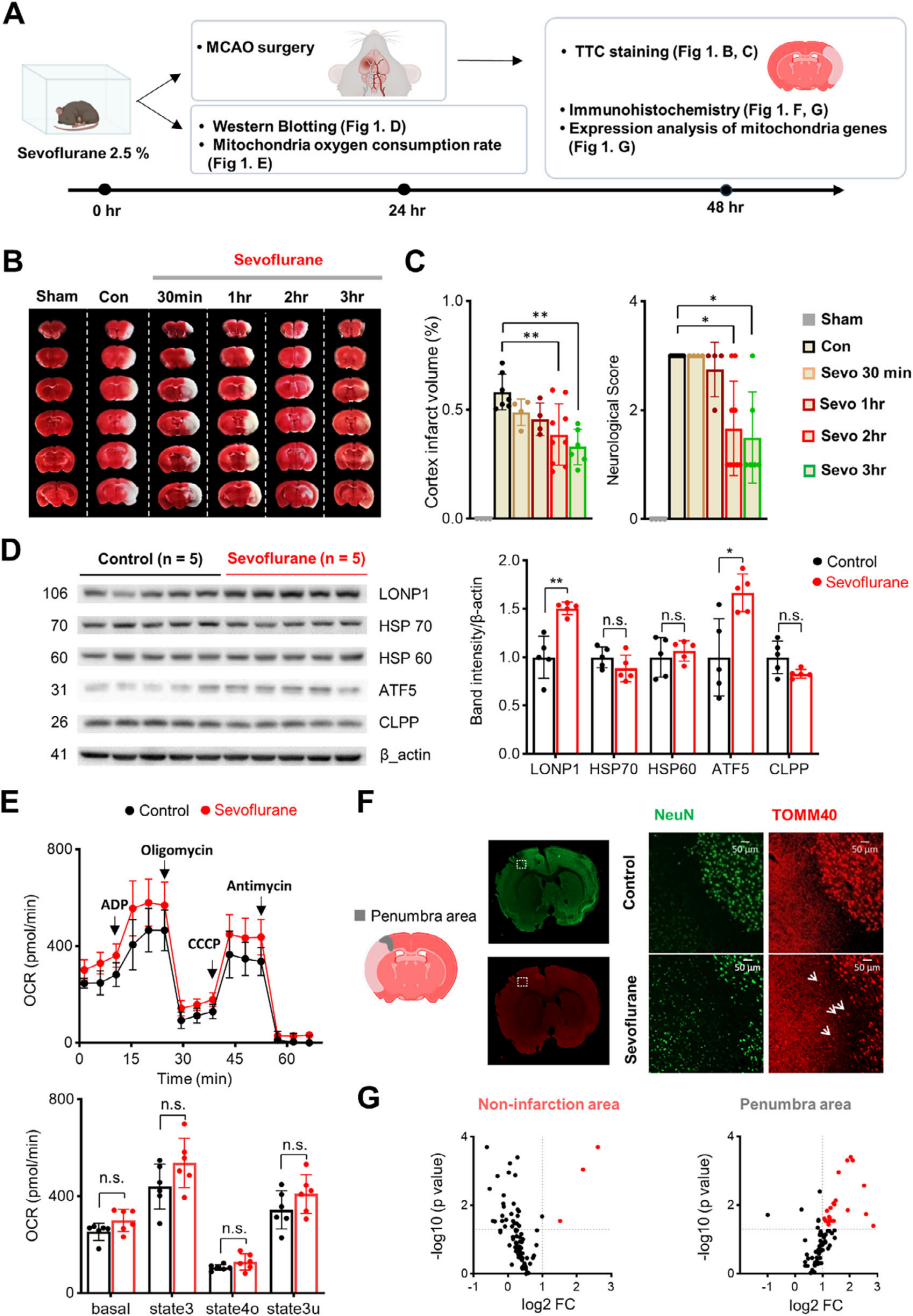
Sevoflurane‐induced preconditioning improves outcomes after cerebral ischemia and upregulates ATF5 and enhances mitochondria function. A) Experimental timeline. B) Representative 2,3,5‐triphenyltetrazolium chloride (TTC)‐stained coronal brain sections showing infarct areas (white) in sham, control, and sevoflurane‐treated groups with different exposure durations (0.5, 1, 2, 3 h). C) Quantification of cortical infarct volume and neurological scores (n = 4–9 per group). D) Western blot analysis of cortical samples obtained 24 h after sevoflurane exposure (n = 5 per group). E) Top: Mitochondrial OCR measured in cortical mitochondria after sequential administration of ADP, oligomycin, CCCP, and antimycin (arrows indicate time of injection). Bottom: Quantification of OCR after subtracting non‐mitochondrial respiration (n = 6 per group). F) TOMM40 and NeuN immunofluorescence staining in coronal brain sections from control and sevoflurane‐treated mice. High magnification images (right panels) show increased TOMM40 signals (white arrows) in the penumbra after sevoflurane preconditioning. G) Differential expression analysis of 83 mitochondria genes in non‐infarct (top) and penumbra (bottom) cortical areas. Mitochondria function‐related genes are listed in Table S1 (Supporting Information). Volcano plots show the number of significantly changed genes (red) in each region after sevoflurane preconditioning and MCAO (n = 4 per group). Values are presented as means ± SD (n.s., not significant; ^*^ *p* < 0.05, ^**^ *p* < 0.01, ^***^ *p* < 0.001).

Previous studies have employed diverse anesthetic conditions to induce preconditioning in the brain,^[^ ^33^ ^]^ but a recent study that systematically compared different sevoflurane concentrations reported that a relatively high concentration is required for inducing neuroprotection.^[^ ^14^ ^]^ Based on this previous study, we exposed mice to 2.5% sevoflurane for varying durations (0.5, 1, 2, or 3 h) to further determine the optimal exposure duration for inducing preconditioning. Twenty‐four hours later, MCAO was performed to assess the induction of preconditioning (**Figure** **1**A). Neuroprotection, defined by reduced infarct volume and improved neurological scores, was observed only in mice that received at least 2 h of sevoflurane exposure (Figure 1B,C). Based on these findings, all subsequent experiments were performed after exposing mice to 2.5% sevoflurane for 2 h. This exposure duration did not result in respiratory depression, as confirmed by blood gas analysis at the end of anesthesia (**Table** **1**).

**Table 1:** Analysis of blood gases and lactate after exposure to 2.5% sevoflurane for 2 h.

	pH	pCO_2_ [mmHg]	pO_2_ [mmHg]	sO_2_ [%]	Lactate [mmol L^−1^]
Animal 1	7.375	34.3	109	98	1.11
Animal 2	7.332	38.4	85	96	0.99
Animal 3	7.346	41.7	91	97	1.19
Mean ± SD	7.35 ± 0.18	38.1 ± 3.0	95 ± 10	97 ± 0.8	1.10 ± 0.08

Note: pCO_2_, partial pressure of carbon dioxide; pO_2,_ partial pressure of oxygen; sO_2,_ oxygen saturation

### 2.2 Sevoflurane‐Induced Neuroprotection Involves Upregulation of Genes Associated with Mitochondrial Metabolism and UPR^mt^

To evaluate the possible role of UPR^mt^ in sevoflurane‐induced preconditioning, we next investigated expression levels of representative UPR^mt^ proteins after sevoflurane exposure (Figure 1D). We found significant increases in expression of the protease, LONP1 (lon peptidase 1, mitochondrial), and the transcriptional factor, ATF5. Given that ATF5 is a key regulator of UPR^mt^,^[^ ^21^ ^]^ it is possible that ATF5‐dependent UPR^mt^ mediates sevoflurane‐induced preconditioning by enhancing mitochondrial function in the brain. However, no significant increase in mitochondrial respiration, measured as oxygen consumption rate (OCR), was detected in mitochondria isolated from the cerebral cortex 24 h after sevoflurane exposure (Figure 1E). Although these findings are based on normal cortex samples rather than ischemia‐affected tissue, our results suggest that ATF5 induction may promote preconditioning not by enhancing, but rather by preserving mitochondrial function. To further evaluate sevoflurane's effects on mitochondrial integrity after MCAO, we conducted an immunohistochemical (IHC) analysis of TOMM40, a mitochondrial outer membrane marker, in the penumbra (Figure 1F). The penumbra, which represents metabolically stressed but potentially salvageable tissue, was identified by NeuN, a neuronal marker.^[^ ^34^ ^]^ Unlike the control group, which showed minimal TOMM40 signal in the penumbra, the sevoflurane‐treated MCAO group exhibited an increase in TOMM40 expression, suggesting preservation of mitochondrial structure within this region (Figure 1F). We next examined whether sevoflurane preconditioning preserves mitochondrial function in the penumbra by conducting targeted expression profiling of 84 mitochondria‐related genes (Figure 1G; Table S1, Supporting Information). Whereas only three genes were upregulated in the non‐infarcted cortex after sevoflurane exposure, 23 genes showed significant upregulation in the penumbra of sevoflurane‐treated mice (Figure 1G). These findings suggest that sevoflurane preconditioning may preferentially protect mitochondrial function in vulnerable brain regions following ischemia/reperfusion injury.

### 2.3 Sevoflurane‐Induced Preconditioning Does Not Develop in Mice with Reduced ATF5 Expression in Excitatory Neurons

**Figure 2:**
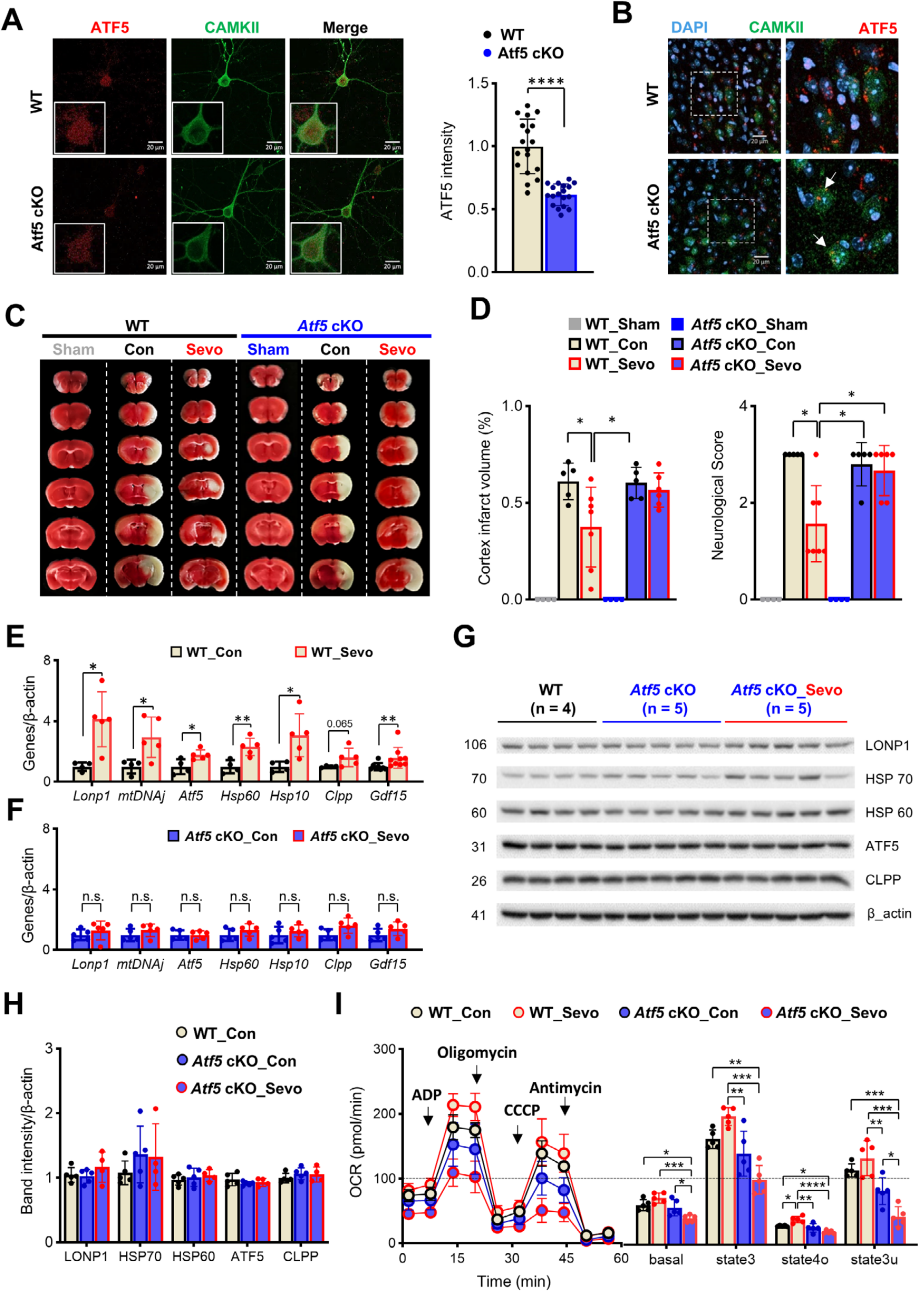
Sevoflurane induces mitochondrial dysfunction and fails to induce preconditioning in *Atf5*‐cKO mice. A) Representative IHC images of cultured primary neurons (n = 18 per group). B) Representative IHC images of anterior cingulate cortex layer V‐VI of *Atf5*‐cKO mice showing the colocalization of CAMKII (green) and ATF5 (red) immunostaining and DAPI‐stained nuclei (blue). White arrows indicate decreased, but remnant, ATF5 expression in excitatory neurons. C) Representative TTC‐stained images of MCAO model Sham, WT, and *Atf5*‐cKO mice after sevoflurane exposure. D) Summary data showing decreased cerebral infarct size and improved neurological score in WT mice but not *Atf5*‐cKO mice (n = 5–7 per group). E) mRNA expression levels of UPR^mt^‐related genes in the cortices of WT mice after exposure to sevoflurane for 6 h (n = 4–9 per group). F) mRNA expression levels of UPR^mt^‐related genes in the cortices of *Atf5*‐cKO mice after exposure to sevoflurane for 6 h (n = 5 per group). G,H) Western blot analysis of cortical samples from *Atf5*‐cKO mice, obtained 24 h after sevoflurane exposure (n = 4–5 per group). I) Left: Mitochondrial function, measured as mitochondrial OCR, determined by assessing respiration of mitochondria isolated from the cortices of mice in control and sevoflurane groups (n = 5 per group). ADP, oligomycin, CCCP, and antimycin were added sequentially, as indicated by arrows. Right: Quantification of OCR after excluding non‐mitochondrial respiration (n = 5 per group). Values are presented as means ± SD (n.s., not significant; ^*^ *p* < 0.05, ^**^ *p* < 0.01, ^***^ *p* < 0.001, ^****^ *p* < 0.0001).

To evaluate the significance of ATF5 in excitatory neurons during sevoflurane preconditioning, we generated *Atf5* conditional knockout (cKO) mice (*Emx1^cre/+^*;*Atf5* ^fl/fl^) by crossing *Emx1‐IRES‐Cre* knock‐in mice with *Atf5* ^fl/fl^ mice (Figure S1A,B, Supporting Information). Quantitative reverse transcription‐polymerase chain reaction (RT‐qPCR) analyses confirmed a significant reduction in the total levels of *Atf5* transcripts and its two splice variants, *Atf5*‐α and ‐β, in samples obtained from the cerebral cortex (Figure S1C, Supporting Information). Because Cre‐mediated recombination in this model is limited to excitatory neurons, we further evaluated ATF5 expression in cultures of primary cortical neurons obtained from *Atf5*‐cKO mice (**Figure** **2**A). Unexpectedly, we found that *ATF5* expression decreased by only ≈30% in excitatory neurons, identified by their expression of CaMKII (calcium/calmodulin‐dependent protein kinase II) (Figure 2A). Immunohistochemistry also confirmed residual ATF5 expression in excitatory neurons in the cerebral cortex of *Atf5*‐cKO mice (Figure 2B). Notably, sevoflurane failed to reduce infarction size or improve neurological scores in MCAO model *Atf5*‐cKO mice, despite the incomplete knockout of *Atf5* (Figure 2C,D). These results confirm the significant role of ATF5 in sevoflurane‐induced preconditioning in the brain.

### 2.4 Sevoflurane Fails to Induce UPR^mt^ or Preserve Mitochondrial Function in *Atf5*‐cKO Mice

ATF5 promotes UPR^mt^ by increasing the expression of genes necessary for overcoming mitochondrial dysfunction and maintaining homeostasis. To evaluate the potential role of ATF5‐dependent UPR^mt^ in sevoflurane‐induced preconditioning, we next evaluated sevoflurane‐induced changes in the expression of UPR^mt^‐related genes in *Atf5*‐cKO mice 6 h after sevoflurane exposure. In contrast to wild‐type (WT) mice, in which various genes involved in UPR^mt^ were significantly upregulated after sevoflurane exposure (Figure 2E), *Atf5*‐cKO mice showed no increase in UPR^mt^‐related genes (Figure 2F). Similarly, protein expression of key UPR^mt^ markers in cortical tissue of *Atf5*‐cKO mice remained unchanged after sevoflurane exposure (Figure 2G, H), consistent with transcriptomic findings (Figure 2F). Notably, overall ATF5 protein levels in cortical lysates from *ATF5*–cKO mice were comparable to those from controls, likely because ATF5 deletion in this model is restricted to excitatory neurons (Figure 2B). Because ATF5 expression remains intact in other cortical cell types, the relatively modest neuron‐specific decrease (≈30%) is not readily apparent in whole‐tissue analyses. Importantly, mitochondrial respiration in isolated mitochondria was significantly decreased 24 h after sevoflurane exposure only in *Atf5*‐cKO mice (Figure 2I, basal and state 3u). These results suggest that the increase in ATF5 expression in excitatory neurons after sevoflurane exposure plays an important role in inducing UPR^mt^ and maintaining mitochondrial function in the brain.

### 2.5 Sevoflurane‐Induced GDF15 Mediates Preconditioning and Mitochondrial Upregulation

**Figure 3:**
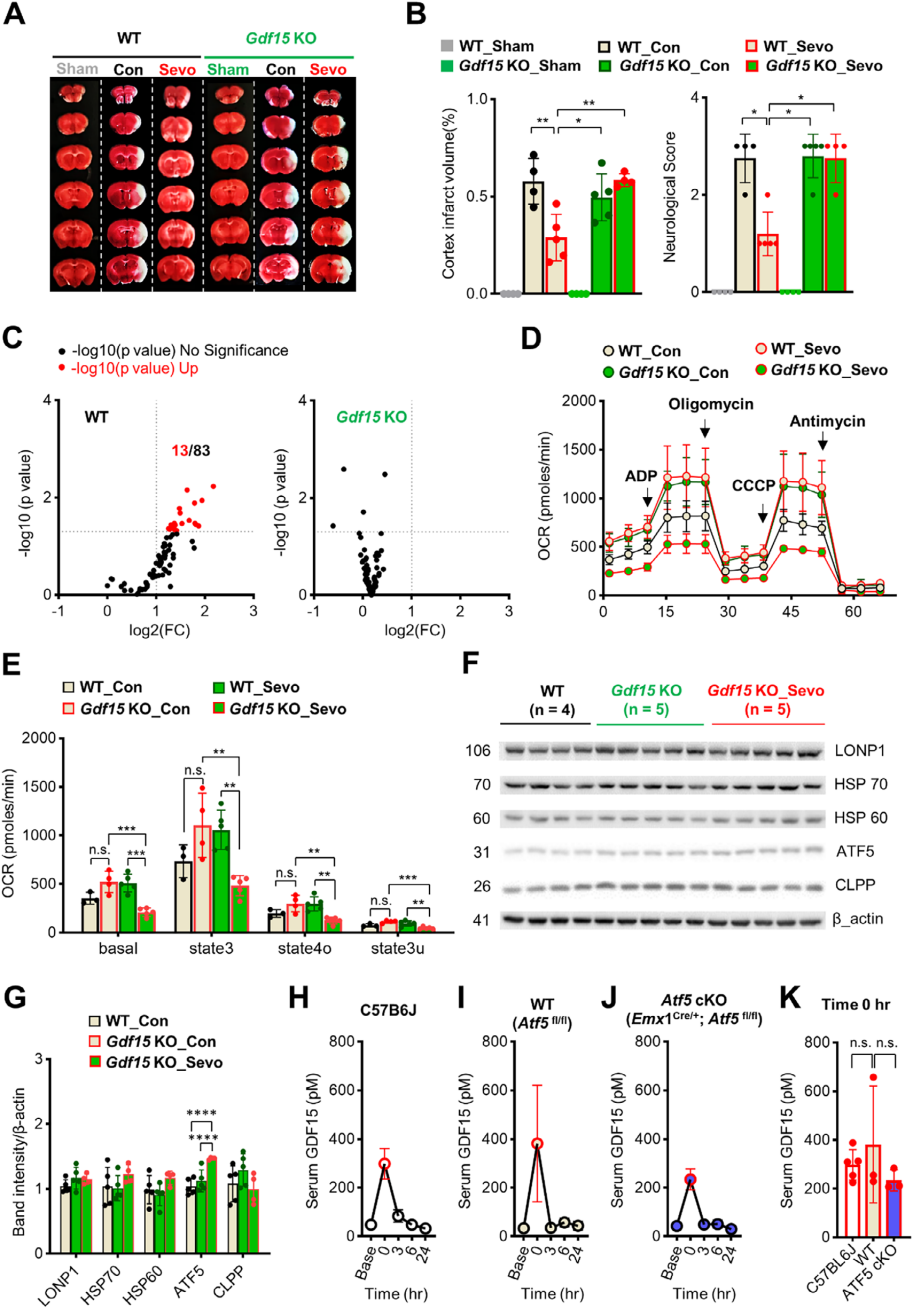
Sevoflurane induces mitochondrial dysfunction and fails to induce preconditioning in *Gdf15*‐KO mice. A) Representative TTC‐stained images of MCAO model WT and *Gdf15*‐KO mice after sevoflurane (Sevo) exposure. B) Summary data showing decreased cerebral infarct volume (n = 4–5 per group). C) Volcano plots showing mitochondrial energy metabolism‐related genes whose levels were increased in the cerebral cortex of WT and *Gdf15*‐KO mice 6 h after exposure to sevoflurane (n = 3–5 per group). D) Mitochondrial function, measured as mitochondrial OCR, was determined by assessing the respiration of mitochondria isolated from the cortices of mice in control and sevoflurane groups. ADP, oligomycin, CCCP, and antimycin were added sequentially, as indicated by arrows. E) Quantification of OCR after excluding non‐mitochondrial respiration (n = 3–5 per group). F,G) Western blot analysis of cortical samples obtained from WT and *Gdf15*‐KO mice 24 h after sevoflurane exposure (n = 4–5 per group). H–J) Serum GDF15 protein levels prior to and 3, 6, and 24 h after exposure to sevoflurane in C57BL/6J, WT, and *Atf5*‐cKO mice. K) Comparison of serum GDF15 directly after sevoflurane exposure in C57BL/6J, WT, and *Atf5*‐cKO mice (n = 3–5 per group). Values are presented as means ± SD (n.s., not significant; ^*^ *p* < 0.05, ^**^ *p* < 0.01, ^***^ *p* < 0.001, ^****^ *p* < 0.0001).

During stressful conditions, cells release stress‐responsive cytokines, also known as mitokines, that act as downstream modulators of UPR^mt^.^[^ ^35^, ^36^ ^]^ GDF15, a representative mitokine that is widely expressed in the brain,^[^ ^32^ ^]^ has been shown to be involved in cardiac preconditioning.^[^ ^37^ ^]^ Interestingly, we also found a significant increase in *Gdf15* mRNA levels in the cerebral cortex 6 h after sevoflurane exposure in WT mice but not in *Atf5‐*cKO mice (Figure 2E,F). These results suggest that GDF15 may function downstream of ATF5 in inducing preconditioning in the brain. To further investigate the role of GDF15, we exposed *Gdf15‐*KO mice to sevoflurane. Consistent with results obtained in *Atf5*‐cKO mice, sevoflurane‐induced preconditioning did not develop in *Gdf15‐*KO mice, as reflected in the absence of a change in infarct size or improvement in neurologic scores (**Figure** **3**A,B). An assessment of differentially expressed genes showed that, whereas 13 of 83 genes related to mitochondrial energy metabolism were increased in WT mice, none of these genes were increased after sevoflurane exposure in the cerebral cortex of *Gdf15*‐KO mice (Figure 3C; Tables S2,S3, Supporting Information). Furthermore, similar to the case for *Atf5*‐cKO mice, sevoflurane exposure significantly decreased mitochondrial respiration in mitochondria isolated from the cerebral cortex of *Gdf15* ‐KO mice 24 h after sevoflurane exposure (Figure 3D, E). To further assess whether the absence of GDF15 influences ATF5 expression following sevoflurane exposure, we measured the expression of ATF5 and other UPR^mt^‐related proteins in the cerebral cortex of *Gdf1*5‐KO mice (Figure 3F,G). Notably, sevoflurane exposure led to a significant increase in ATF5 expression, even in the absence of GDF15, indicating that ATF5 functions upstream of GDF15 in this signaling pathway.

Compared with the brain, other organs, such as the liver and kidney, express significantly higher levels of GDF15.^[^ ^38^ ^]^ Therefore, it is possible that sevoflurane induced in these organs increases systemic GDF15, which may act on the brain. Indeed, we observed an immediate but transient increase in serum GDF15 levels following sevoflurane exposure in C57BL/6J, *Atf5* ^fl/fl^ (WT), and *EMX1^Cre/+^*;*Atf5* ^fl/fl^ (*Atf5*‐cKO) mice (Figure 3H–J). However, considering that sevoflurane‐induced preconditioning did not develop in *Atf5*‐cKO mice despite the significant increase in serum GDF15 levels, our results suggest that ATF5‐dependent GDF15 endogenously released in excitatory neurons is necessary and sufficient for preconditioning. Furthermore, there was no difference in serum GDF15 levels among C57BL/6J, WT, and *Atf5*‐cKO mice (Figure 3K), suggesting that GDF15 expressed in excitatory neurons does not contribute to the increase in serum GDF15 after sevoflurane exposure.

### 2.6 ATF5 Overexpression in Excitatory Neurons Increases *Gdf15* mRNA Expression and Enhances Mitochondrial Respiration

**Figure 4:**
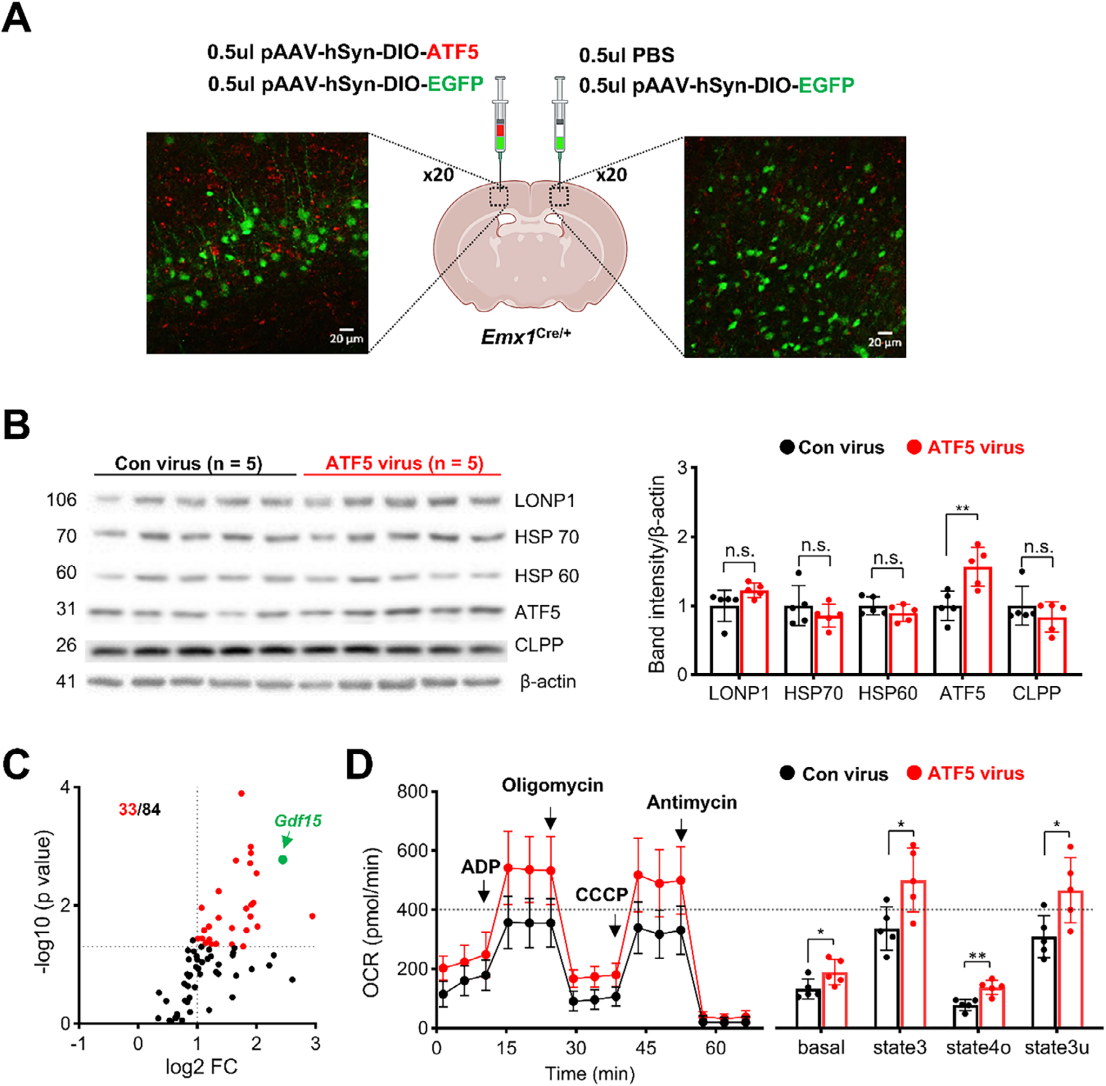
ATF5 overexpression in excitatory neurons upregulates GDF15 and enhances mitochondrial function. A) Representative IHC images confirming pAAV‐hSyn‐DIO‐ATF5 expression after stereotaxic injection together with pAAV‐hSyn‐DIO‐EGFP into the cortex of *Emx1^cre/+^* mice. B) Western blot analysis of cortical samples surrounding the injection site, performed 4 weeks after AAV injection (n = 5 per group). C) Volcano plots showing mitochondrial energy metabolism‐related genes (including *Gdf15*) whose levels were increased around the injection region (n = 5 per group). D) Left: Mitochondrial function, measured as mitochondrial OCR, determined by assessing respiration of mitochondria isolated from the cortex region injected with control or ATF5 virus (n = 5 per group). ADP, oligomycin, CCCP, and antimycin were added sequentially, as indicated by arrows. Right: Quantification of OCR after excluding non‐mitochondrial respiration (n = 5 per group). Values are presented as means ± SD (n.s., not significant; ^*^ *p* < 0.05, ^**^ *p* < 0.01).

Our results suggest that ATF5 regulates GDF15 expression. Although a direct relationship between ATF5 and GDF15 has not been previously established, studies have shown that *GDF15* expression is regulated by ATF4, another major UPR^mt^‐related transcription factor.^[^ ^35^, ^36^ ^]^ Considering that ATF5 expression is regulated by ATF4,^[^ ^39^, ^40^, ^41^ ^]^ it is possible that GDF15 expression is also regulated through ATF5. To confirm this, we constructed a viral vector expressing ATF5 specifically in excitatory neurons (pAAV‐hSyn‐DIO‐ATF5) and validated increased ATF5 expression 4 weeks after injecting AAVs containing this construct into the cerebral cortex of *Emx1^cre/+^* mice (**Figure** **4**A,B). Consistent with our initial results, ATF5 overexpression was associated with increased expression of genes related to mitochondrial energy metabolism (33 of 84), including *Gdf15* (Figure 4C; Table S4, Supporting Information). Respiration was also increased in mitochondria isolated from cerebral cortex regions expressing AAVs (Figure 4D). These results provide direct evidence that expression of GDF15, which plays a significant role in regulating mitochondrial function, is regulated by ATF5 in excitatory neurons.

### 2.7 Widespread ATF5 Overexpression in Excitatory Neurons Induces Neuroprotective Preconditioning Against MCAO

**Figure 5:**
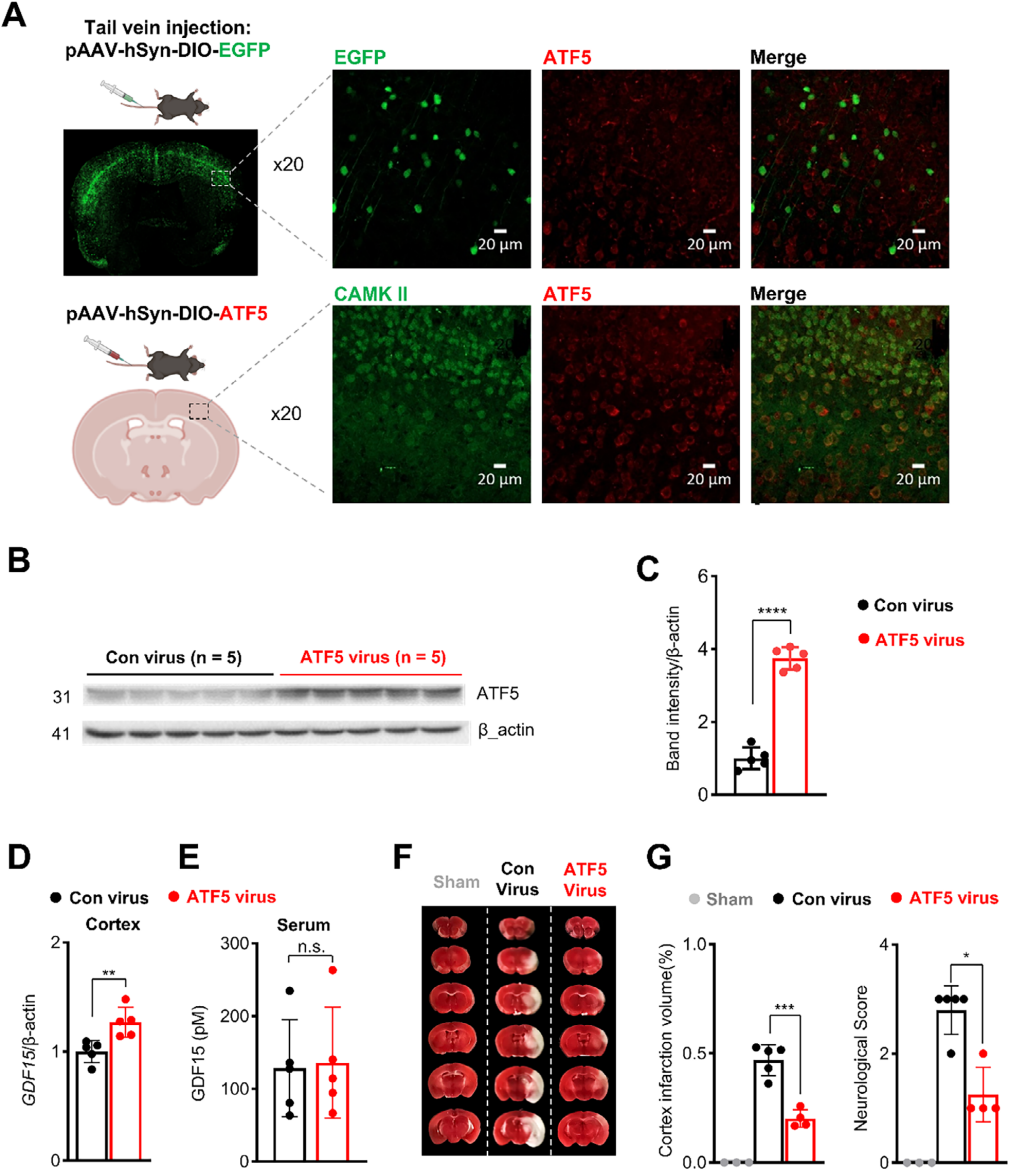
ATF5 overexpression in excitatory neurons provides neuroprotection against ischemic injury. A) Upper panel: Representative image of EGFP (green) and ATF5 (red) expression in the cortex 4 weeks after intravenous injection of pAAV‐hSyn‐DIO‐EGFP. Lower panel: Representative image of CAMKII (green) and ATF5 (red) expression in the cortex 4 weeks after intravenous injection of pAAV‐hSyn‐DIO‐ATF5 in *Emx1^cre/+^* mice. B,C) Western blot analysis of ATF5 protein levels in the cortex after intravenous injection of pAAV‐hSyn‐DIO‐EGFP and pAAV‐hSyn‐DIO‐ATF5 in *Emx1^cre/+^* mice (n = 5 per group). D) RT‐qPCR analysis of *Gdf15* mRNA expression in the cortex after intravenous injection of pAAV‐hSyn‐DIO‐EGFP and pAAV‐hSyn‐DIO‐ATF5 in *Emx1^cre/+^* mice (n = 5 per group). E) Serum GDF15 protein levels in *Emx1^cre/+^* mice 4 weeks after injection with pAAV‐hSyn‐DIO‐EGFP and pAAV‐hSyn‐DIO‐ATF5 (n = 5 per group). F) Representative TTC‐stained coronal brain sections after intravenous injection of pAAV‐hSyn‐DIO‐EGFP and pAAV‐hSyn‐DIO‐ATF5 in *Emx1^cre/+^* mice. G) Quantification of cortical infarct volume and neurological score 24 h after MCAO (n = 4–5 per group). Values are presented as means ± SD (n.s., not significant; ^*^ *p* < 0.05, ^**^ *p* < 0.01, ^***^ *p* < 0.001, ^****^ *p* < 0.0001).

To further investigate whether ATF5 overexpression alone is sufficient to induce neuroprotection against ischemic stress, we performed MCAO in *Emx1^cre/+^* mice 4 weeks after systemic administration of either pAAV‐hSyn‐DIO‐EGFP or pAAV‐hSyn‐DIO‐ATF5 (≈2 × 10^11^ vg mL^−1^; 200 µL per mouse) (**Figure** **5**A). Widespread cortical GFP expression was observed in mice receiving pAAV‐hSyn‐DIO‐EGFP (Figure 5A, upper panel), confirming efficient viral transduction at the administered dose and interval. We next verified ATF5 expression in excitatory neurons of the pAAV‐hSyn‐DIO‐ATF5 group (Figure 5A, lower panel), demonstrating co‐localization of ATF5 signal with CAMKII following systemic viral delivery. Increased ATF5 expression levels were further confirmed by Western blot analysis (Figure 5B,C). We also found that widespread ATF5 expression was associated with increased mRNA levels of *GDF15* (Figure 5D), in the absence of a change in serum GDF15 levels (Figure 5E). Most importantly, mice overexpressing ATF5 displayed significantly reduced infarct volumes and improved neurological scores following MCAO compared with EGFP controls, mirroring the neuroprotective effects observed with sevoflurane preconditioning (Figure 5F,G). These results support the role of GDF15 as an effector of ATF5‐mediated neuroprotection.

### 2.8 Baseline Cortical ATF5 Expression and Mitochondrial Respiration are Preserved in Aged Mice, Whereas *Gdf15* mRNA Levels are Elevated

**Figure 6:**
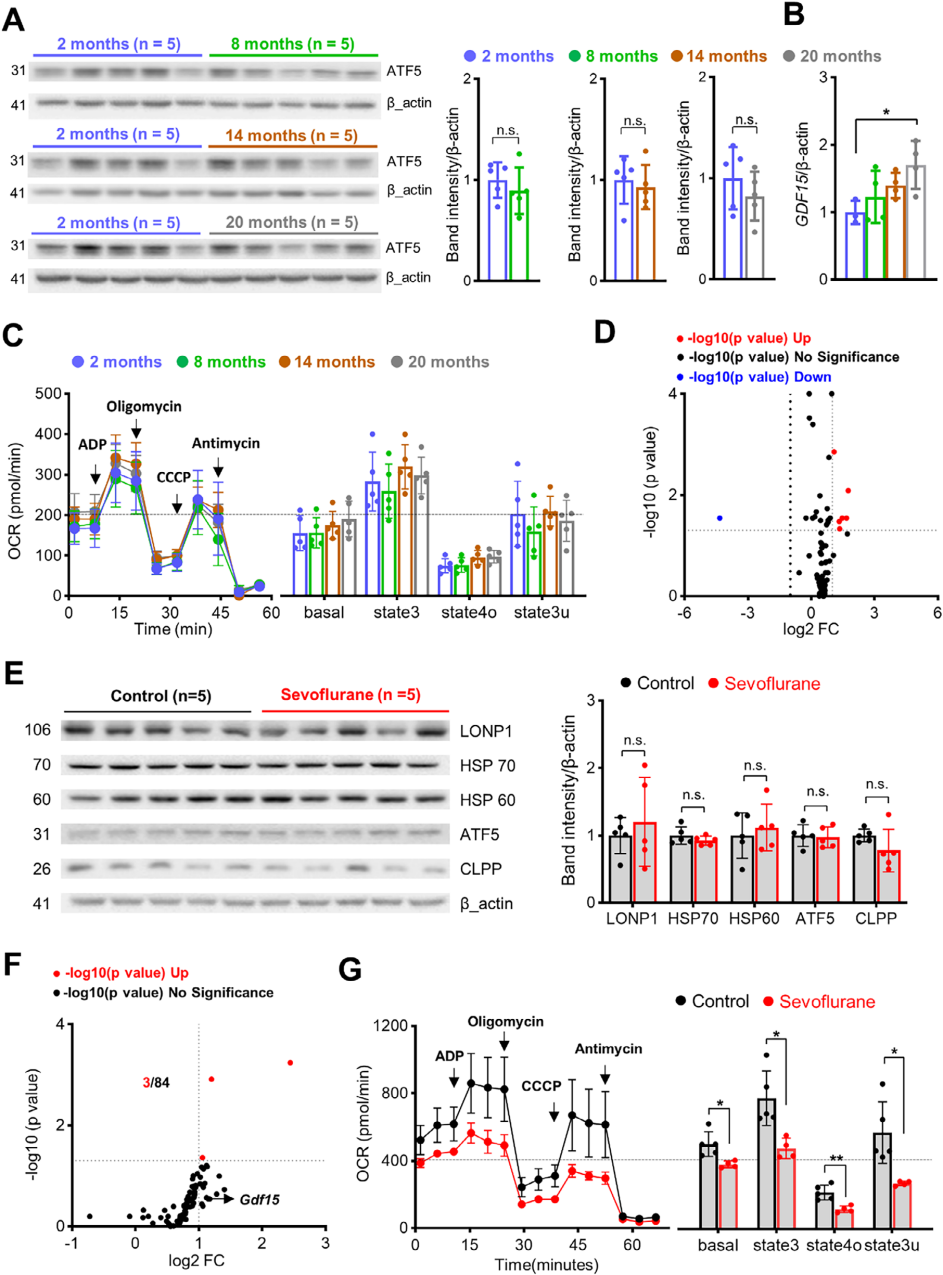
Sevoflurane‐induced activation of ATF5‐dependent UPR^mt^, GDF15 expression, and mitochondrial protection are absent in cerebral cortices of aged mice. A) Western blot analysis of ATF5 protein levels in the cortex of mice at 2‐, 8‐, 14‐, and 20‐month of age (n = 5 per group). B) *Gdf15* mRNA expression in the cortex at 2‐, 8‐, 14‐, and 20‐ months, determined by RT‐qPCR and normalized to β‐actin (n = 3–4 per group). C) Mitochondrial OCR in isolated cortical mitochondria from 2‐, 8‐, 14‐, and 20‐month‐old mice. Left: Representative OCR traces in response to sequential addition of ADP, oligomycin, CCCP, and antimycin. Right: Quantification of basal, state 3, state 4o, and state 3u respiration. D) Volcano plot showing differential expression of 83 mitochondrial energy metabolism‐related genes in the cortex of 20‐month‐old versus 2‐month‐old mice. Red dots, upregulated genes; blue dots, downregulated genes; black dots, non‐significant changes. E) Western blot analysis of proteins in cortical samples from aged mice obtained 24 h after sevoflurane exposure (n = 5 per group). F) Volcano plot showing changes in mitochondrial energy metabolism‐related genes in the cortex of aged mice 6 h after sevoflurane exposure (n = 3–4 per group). G) Left: Mitochondrial function assessed by OCR in isolated cortical mitochondria from aged mice in control and sevoflurane‐treated groups (n = 4–5 per group). Sequential additions of ADP, oligomycin, CCCP, and antimycin are indicated by arrows. Right: Quantification of OCR, excluding non‐mitochondrial respiration (n = 4–5 per group). Values are presented as means ± SD (n.s., not significant; ^*^ *p* < 0.05).

Unlike preclinical studies, which have consistently reported anesthesia‐induced preconditioning, few clinical studies have supported the feasibility of neuroprotection after anesthesia.^[^ ^15^, ^16^ ^]^ Because we found that ATF5‐dependent UPR^mt^ activation, GDF15 expression, and mitochondrial upregulation are key players in sevoflurane‐induced preconditioning, it is possible that these pathways are altered by specific factors in clinical trials. One potential factor is the age of patients. Whereas preclinical studies have been performed in young mice, most clinical studies have been performed in elderly patients.^[^ ^42^, ^43^, ^44^, ^45^ ^]^ Although mitochondrial dysfunction is a well‐established hallmark of aging, age‐related changes in ATF5 expression have not been thoroughly investigated. Thus, it is possible that ATF5 expression declines with age, thereby impairing the induction of anesthesia‐induced preconditioning. Alternatively, the inducibility of UPR^mt^ may be reduced with aging,^[^ ^46^ ^]^ even if baseline ATF5 expression remains unchanged. To investigate these possibilities, we assessed age‐dependent changes in ATF5 expression in cerebral cortex samples from 2‐, 8‐, 14‐, and 20‐month‐old mice. Our results showed no significant difference in ATF5 protein levels across age groups compared with 2‐month‐old mice, indicating that aging does not affect baseline expression of ATF5 (**Figure** **6**A). However, despite being unable to analyze GDF15 protein levels, we did observe a significant increase in *Gdf15* mRNA expression in 20‐month‐old mice relative to 2‐month‐old mice (Figure 6B). Notwithstanding this change in *Gdf15*, mitochondrial respiration, as measured as OCR, remained comparable across all ages (Figure 6C). Furthermore, a transcriptomic analysis of 83 mitochondrial energy metabolism‐related genes in the cortex of 2‐ and 20‐month‐old mice showed similar overall expression profiles, with only one gene downregulated and six genes upregulated in aged mice (Figure 6D; Table S5, Supporting Information).

### 2.9 Sevoflurane‐Induced Activation of ATF5‐Dependent UPR^mt^, Upregulation of GDF15, and Mitochondrial Protection are Absent in the Cerebral Cortex of Aged Mice

Our results suggest that baseline expression of intrinsic mitochondrial and stress‐response factors are relatively well preserved with aging; thus, diminished inducibility of UPR^mt^ may underlie the absence of a neuroprotective response to anesthesia‐induced preconditioning in older individuals. To investigate the impact of aging on sevoflurane‐induced preconditioning, we exposed aged mice (18–20 months) to sevoflurane. Unlike young mice (Figure 1D), these mice showed no increase in the expression of UPR^mt^ proteins 24 h after sevoflurane exposure (Figure 6E). Sevoflurane also failed to increase *Gdf15* expression in aged mice, and only upregulated a limited number of genes related to mitochondrial energy metabolism (Figure 6F; Table S6, Supporting Information). Importantly, and similar to results obtained in *Atf5*‐cKO and *Gdf15*‐KO mice, OCR was significantly decreased in isolated mitochondria from aged mice 24 h after sevoflurane exposure (Figure 6G). These results suggest that sevoflurane fails to activate ATF5‐dependent UPR^mt^ signaling and sustain mitochondrial homeostasis in the aged brain, thereby preventing the development of anesthesia‐induced preconditioning. Unfortunately, we were unable to confirm whether preconditioning was absent following MCAO in aged mice, as none survived beyond 24 h after the procedure.

## 3 Discussion

Neuroprotective strategies may be beneficial in aging societies to reduce the severity of perioperative stroke. In this study, we demonstrated that sevoflurane‐induced neuroprotection is associated with the upregulation of genes involved in UPR^mt^ and mitochondrial metabolism. Our findings emphasize the critical role of ATF5, a key transcription factor, in mediating these protective effects. Specifically, we observed that sevoflurane preconditioning significantly upregulates ATF5 and its downstream target GDF15, a regulator of mitochondrial function, in the cerebral cortex. This upregulation is critical for the neuroprotective effects following ischemic injury. To our knowledge, this is the first demonstration that sevoflurane‐induced preconditioning is mediated by ATF5‐regulated GDF15 expression in the brain. Notably, we found that this mechanistic pathway was not activated in the brain of aged mice, suggesting that age‐specific strategies may be necessary to reduce the risk of perioperative stroke.

We further found that upregulation of ATF5 was necessary for the development of sevoflurane‐induced preconditioning, as evidenced by the failure of sevoflurane exposure to improve infarct size or neurological outcomes in *Atf5*‐cKO mice. ATF5 plays a significant role in maintaining mitochondrial function, particularly in the context of cellular stress responses. A comparison of changes in gene expression following sevoflurane exposure and viral overexpression of ATF5 reinforces the role of ATF5 in regulating mitochondrial function and inducing preconditioning after sevoflurane exposure. Among the 83 mitochondrial energy metabolism‐related genes examined, 13 were upregulated after sevoflurane exposure, 11 of which overlapped with genes increased following ATF5 overexpression in the cortex (Table S7, Supporting Information). Furthermore, widespread ATF5 overexpression prior to MCAO significantly reduced infarct volumes and improved neurological scores, closely recapitulating the neuroprotective phenotype observed with sevoflurane preconditioning. These findings are consistent with previous studies identifying ATF5 as a key regulator of cardioprotection,^[^ ^21^ ^]^ a setting in which ATF5 modulates the expression of genes critical for preserving mitochondrial integrity under stress conditions.^[^ ^47^ ^]^

A significant finding of this study is the identification of GDF15 as a downstream effector of ATF5 in excitatory neurons. Prior in vitro studies have demonstrated that ATF5 regulates GDF15 expression in cancer cell lines, including HepG2 hepatocellular carcinoma cells and an epithelioid hemangioendothelioma patient‐derived cell line.^[^ ^48^, ^49^ ^]^ Our results extend these findings by demonstrating ATF5‐dependent GDF15 expression in a non‐cancer in vivo model, specifically within neurons following anesthetic preconditioning in the setting of cerebral ischemia. GDF15, a member of the transforming growth factor‐beta (TGF‐β) superfamily, is associated with various pathological conditions.^[^ ^50^, ^51^, ^52^, ^53^ ^]^ It is also upregulated in response to ischemic and hypertrophic conditions in the heart and liver, where it acts as a protective mechanism to prevent cellular damage.^[^ ^37^, ^54^, ^55^ ^]^ Although a previous study suggested that GDF15 provides protection against ischemia‐reperfusion injury by inhibiting inflammation, particularly neutrophil infiltration and migration,^[^ ^56^ ^]^ its role in the brain, especially in the context of anesthetic preconditioning, remained unclear.

Importantly, our data also suggests that neuronal, rather than systemic, GDF15 plays a role in mediating the protective effects of sevoflurane. Whereas previous studies emphasized systemic GDF15 actions through the GFRAL (glial cell line‐derived neurotrophic factor [GDNF] family receptor alpha‐like) receptor in the hindbrain,^[^ ^57^ ^]^ we found that preconditioning failed to occur in *Atf5*‐cKO mice, despite elevated serum GDF15 levels, indicating that peripheral GDF15 alone is insufficient to induce neuroprotection. These observations suggest that ATF5‐dependent, neuron‐intrinsic GDF15 expression is essential for preconditioning. Interestingly, GDF15 has also been proposed as a marker of poor general health, with elevated levels commonly seen in chronic conditions and frailty.^[^ ^58^ ^]^ For example, a recent study reported that ponsegromab, a humanized monoclonal antibody targeting GDF15, reduced cachexia symptoms in cancer patients with high circulating GDF15.^[^ ^59^ ^]^ Although our findings suggest that increased serum GDF15 does not contribute to neuroprotection in this context, further studies are warranted to investigate the effects of elevated peripheral GDF15 following anesthetic exposure, particularly in aged or comorbid populations.

Another key finding of our study is the lack of sevoflurane‐induced upregulation of ATF5 in aged mice. Although the idea of providing neuroprotection against ischemia/reperfusion injury through anesthetic exposure initially appeared promising, early studies in aged hearts soon reported that such protective effects may diminish with age.^[^ ^60^ ^]^ For example, one study comparing young (3–5 months) and old (20–24 months) rats demonstrated that isoflurane exposure failed to increase reactive oxygen species levels or reduce infarct size in aged cardiac tissue.^[^ ^61^ ^]^ More recently, the lack of sevoflurane‐induced cardioprotection in 24‐month‐old rats was attributed to the failure to activate NF‐κB–regulated apoptotic genes.^[^ ^62^ ^]^ Although these investigations have focused on cardiac protection, they may offer valuable insights into cerebral preconditioning, as the heart and brain share underlying preconditioning mechanisms.^[^ ^63^, ^64^ ^]^ In the current study, we provide another potential mechanism, showing that sevoflurane fails to activate ATF5‐dependent UPR^mt^ in the brain of aged mice. Our results also suggest that, whereas ATF5 expression remains stable with age, GDF15 is highly sensitive to aging, possibly reflecting accumulated cellular stress or mitochondrial dysfunction in older individuals.^[^ ^57^, ^65^ ^]^ Specifically, when GDF15 is already chronically elevated, the capacity to further enhance protective signaling through the ATF5–GDF15 axis may be reduced. However, our current data do not directly address the functional effects of ATF5 or GDF15 overexpression in aged MCAO models; thus, further studies are necessary to clarify the effects of ATF5‐dependent UPR^mt^ activation in the context of elevated GDF15 expression in the aged brain.^[^ ^66^ ^]^ Interestingly, high‐intensity exercise (4 weeks) has been shown to activate UPR^mt^ in the skeletal muscles of 24‐month‐old mice.^[^ ^67^, ^68^ ^]^ Although mitochondria from different organs may act differently, additional studies may ultimately reveal whether a prolonged or repeated stimulus can provide neuroprotection by reactivating UPR^mt^ in the aging brain.

Our study focused on the neuroprotective effects of sevoflurane mediated by the ATF5–GDF15 axis; however, there is also a widely held concern that general anesthesia is associated with postoperative cognitive dysfunction in elderly patients.^[^ ^69^ ^]^ Although our results suggest that the impaired ability of sevoflurane to induce ATF5 and GDF15 expression may block the induction of preconditioning in the aged brain, it is also possible that the loss of mitochondrial homeostasis after sevoflurane exposure is involved in the development of postoperative cognitive dysfunction specifically seen in aged patients. However, our anesthesia protocol did not affect learning in either young or aged mice in the Barnes maze test (Figure S2, Supporting Information), suggesting the absence of effects on cognition. Our results are in line with recent, large‐scale, randomized clinical trials reporting no differences in the incidence of postoperative delirium between general and regional anesthesia.^[^ ^44^, ^70^ ^]^ Notably, previous studies that have reported anesthesia‐induced cognitive deficits in aged rodents often employed longer or repeated anesthetic exposures.^[^ ^71^ ^]^ Further investigation using varied anesthesia protocols will be essential to determine whether failure to activate ATF5‐dependent UPR^mt^ contributes to cognitive dysfunction in the aging brain.

There are several limitations of the present study. First, although we aimed to selectively knock out ATF5 in excitatory neurons (*Emx1^cre/+^;Atf5* ^fl/fl^), ATF5 expression was only reduced by ≈30%. Although this residual expression complicates a full assessment of ATF5's function, our findings are consistent with a previous study demonstrating the absence of ischemia‐induced preconditioning in whole‐body ATF5‐KO mice.^[^ ^21^ ^]^ Given that preconditioning failed despite residual ATF5, sevoflurane‐induced upregulation of ATF5, rather than its baseline expression, may be essential for preconditioning. Second, although sevoflurane exposure failed to increase ATF5 expression in aged mice, we were unable to directly assess neuroprotection owing to significant mortality within 24 h after MCAO. This high mortality likely reflects increased susceptibility to ischemic injury and impaired endogenous repair mechanisms, including a decline in cerebrovascular reserve,^[^ ^72^ ^]^ increased oxidative stress and impaired antioxidant defenses,^[^ ^73^ ^]^ and dysregulated inflammatory responses.^[^ ^74^ ^]^ Despite this limitation, the established loss of preconditioning in aged hearts supports the likelihood of similar deficits in the brain. Third, the neuroprotective effect of sevoflurane was examined at a single time point (24 h after sevoflurane exposure). Studies have shown that preconditioning develops in 2 phases: an early preconditioning phase that lasts for 2–3 h after anesthesia, and a late preconditioning phase that appears after 12–24 h and lasts up to 72 h.^[^ ^75^, ^76^ ^]^ Although it is possible that ATF5 and GDF15 also play significant roles in the early phase,^[^ ^77^ ^]^ additional studies are needed to confirm the mechanisms in each phase. Fourth, the GDF15 analysis was limited to mRNA expression, as protein levels were undetectable by Western blot or ELISA, a limitation also noted in previous studies.^[^ ^78^ ^]^ However, given the consistent changes in GDF15 mRNA expression observed across multiple experimental conditions in our study, we believe these transcriptional data reliably reflect the regulatory relationship between ATF5 and GDF15. Fifth, although we employed a viral vector system that specifically targets excitatory neurons, our results lack direct cell‐type‐specific evidence for ATF5‐dependent GDF15 regulation. Visualization of GDF15 using immunohistochemistry was unsuccessful, likely because of its low abundance. Although studies suggest that neuronal GDF15 expression predominates under stress conditions,^[^ ^32^, ^79^ ^]^ we cannot rule out the possibility that other cell types, such as astrocytes or microglia, may also contribute.

In conclusion, by manipulating the expression of the transcription factor ATF5 specifically in excitatory neurons, we identified a novel mechanism of sevoflurane‐induced preconditioning. We demonstrated that sevoflurane exposure activates ATF5‐dependent UPR^mt^, resulting in increased expression of genes related to mitochondrial function and energy metabolism, including GDF15, to maintain mitochondrial homeostasis. Unlike most studies, which have focused on systemic GDF15, our results suggest that endogenous neuronal GDF15 is essential for the development of neuroprotection against ischemia/reperfusion injury. Importantly, we also discovered that sevoflurane failed to activate the ATF5–GDF15 signaling pathway in the aged brain, leading to a decrease in mitochondrial function after sevoflurane. Considering that patient populations are steadily aging, therapeutic approaches that enhance mitochondrial function in the aged brain may provide additional protection against perioperative stroke.

## 4 Experimental Section

### Animals

This study was approved by the Committee on Animal Research at Chungnam National University (Daejeon, South Korea; 202203A‐CNU‐050). To control for fluctuations in estrogen levels, which can influence infarct size and neurological outcomes in female mice, only male mice were used. Mice were housed in cages (<6 mice/cage) at 24–25 °C under a 12‐h light/dark regimen and provided food and water ad libitum. Conditional *Atf5* knockout (*Atf5* cKO) mice were generated by crossing *Emx1‐IRES‐Cre* knock‐in mice with floxed *Atf5* (*Atf5* ^fl/fl^) mice in which exon 3 was targeted (Cyagen Biosciences, CA, USA).^[^ ^80^, ^81^ ^]^ The *Atf5* ^fl/fl^ line was backcrossed with C57BL/6J mice (Damul Science, Daejeon, Korea) for more than three generations before crossing. GDF15 KO mice were derived from an inbred C57BL/6 strain that was kindly provided by Dr. S. Lee (Johns Hopkins University School of Medicine, Baltimore, MD, USA).

### Sevoflurane Exposure

Mice were divided into two groups: Control and Sevoflurane. Mice in the Control group were placed in an anesthesia chamber and exposed to a constant flow of fresh gas (fraction of inspired oxygen [FiO_2_], 0.4; flow rate, 4 L min^−1^) for 2 h and 10 min. Mice in the Sevoflurane group were treated identically, but received 2.5% sevoflurane mixed with the fresh gas for 2 h, followed by 10 min of washout with fresh gas for recovery. Body temperature was maintained at 37 °C by immersing the anesthesia chamber in a temperature‐controlled water bath. FiO_2_ and sevoflurane concentrations were monitored using an m‐CAiO gas analyzer module (Datex‐Ohmeda, Helsinki, Finland). Adequate respiration was confirmed by blood gas analysis in a subset of mice (Table 1).

### Blood Gas Analysis

To confirm that the experimental protocol did not induce significant respiratory distress, arteriovenous mixed blood samples were collected by decapitation immediately after sevoflurane exposure^[^ ^82^ ^]^ for blood gas analysis. For ethical reasons, control mice were also briefly exposed to sevoflurane before sampling. Mixed blood gases were analyzed using an i‐STAT analyzer (Abbott Laboratories, Abbott Park, IL, USA).

### MCAO Model

The MCAO model was generated as previously described.^[^ ^83^ ^]^ Following brief induction with 5% sevoflurane for 3 min, tracheal intubation was performed using a 20‐gauge intravenous catheter (REF 382434; BD Angiocath Plus, Singapore). To ensure proper catheter positioning and prevent one‐lung ventilation or gas leakage, the intravenous catheter was modified using the distal portion of a 200 µL pipette tip (Figure S3, Supporting Information). Mice were mechanically ventilated (Minivent Model 845; Havard Apparatus) with 2.5% sevoflurane in 40% oxygen (flow rate 4 L min^−1^; respiratory rate 180 breaths min^−1^; tidal volume 10 µL g^−1^). Rectal temperature was maintained at 37 ± 0.5 °C using a feedback‐controlled heating pad. Adequate ventilation was confirmed by arterial blood gas analysis (Figure S3, Supporting Information). After exposing the right common carotid artery (CCA) via a ventral midline neck incision, the right external carotid artery (ECA) and internal carotid artery (ICA) were identified. The CCA and ECA were permanently clamped, and a 6‐0 silicone rubber‐coated monofilament (L56PK10; Doccol Corporation, Sharon, MA, USA) was carefully inserted into the right CCA, upstream from the ECA clamp, and advanced toward the ICA until slight resistance was felt. The monofilament was inserted into the artery from the bifurcation of the ECA and ICA to the MCA, a distance of ≈9–10 mm. The suture was then tightly fastened around the CCA for 40 min. After the occlusion period, the suture was removed, and the CCA was permanently fastened to prevent blood flow. Changes in microvascular blood flow in the MCA territory (1 mm posterior and 5 mm lateral to Bregma on the right parietal cranial region) were confirmed by laser‐Doppler microscopy (PeriFlux 6000; PERIMED, Sweden) (Figure S4, Supporting Information). Only mice with ≥ 80% flow reduction during the ischemic period were included in the analysis.^[^ ^84^ ^]^

### Evaluation of Neurological Function and Infarct Volume in the MCAO Model

The neurological behavior of MCAO mice was evaluated 24 h after reperfusion using a modified Bederson scoring system, as follows: 0, no deficit; 1, forelimb flexion; 2, forelimb flexion with decreased resistance to lateral push; 3, unidirectional circling; 4, longitudinal spinning or seizure activity; and 5, no spontaneous movement.^[^ ^85^ ^]^ Infarct volume was assessed by 2,3,5‐triphenyltetrazolium chloride (TTC) staining. Briefly, coronal brain slices (1,000 µm thick) were prepared 24 h after MCAO using a VT1200 vibratome (Leica Microsystems, Wetzlar, Germany). Slices were incubated in 2% TTC (Sigma‐Aldrich, St. Louis, MO, USA) prepared in phosphate‐buffered saline (PBS) for 20–25 min at room temperature (RT), then fixed in 10% neutral‐buffered formalin (Sigma–Aldrich) at 4 °C until imaging. Infarct volume was quantified using ImageJ software (NIH, Bethesda, MD, USA).

### Western Blotting

Protein extracts from cerebral cortex tissue were prepared by homogenization in lysis buffer supplemented with phosphatase inhibitors (Sigma–Aldrich, Cat# PHOSS‐RO) and protease inhibitors (Sigma‐Aldrich, Cat# P8340) using a TissueLyser II (Qiagen, Germany). The homogenates were centrifuged at 12,000 × g for 20 min at 4 °C, and supernatants were collected for analysis. Samples from primary cultured neurons (see below) were obtained by first aspirating the culture medium, washing the cells with ice‐cold PBS, and then lysing them in a buffer containing phosphatase and protease inhibitors. Protein concentrations in tissue homogenates and cultured cell lysates were measured using the SMART BCA Protein Assay Kit (iNtRON). Equal amounts of protein (20 µg per sample) were separated by sodium dodecyl sulfate–polyacrylamide gel electrophoresis (SDS–PAGE; Bio‐Rad, CA, USA) and transferred onto polyvinylidene fluoride (PVDF) membranes (0.45 µm pore size; Merck Millipore, Ireland) at 385 mA for 1 h. Membranes were blocked with 5% skim milk in Tris‐buffered saline containing Tween 20 (TBS‐T; 10 mm Tris‐HCl, pH 7.6, 150 mm NaCl, 0.1% Tween 20) for 1 h, followed by incubation with primary and appropriate secondary antibodies. The following primary antibodies were used: CLPP (Abcam, CAT# ab124822), ATF5 (Abcam, CAT# ab184923), phosphorylated‐Eif2α (p‐Eif2α; Cell Signaling Technology, CAT#3398), Eif2α (Cell Signaling Technology, CAT#5324ATF4), ATF4 (Abcam, CAT# ab184909), HSP60 (Abcam, CAT# ab46798), HSP70 (Santa Cruz Biotechnology, CAT#J1216), ATF6 (Abcam, CAT# ab203119), BIP (GRP78) (Abcam, CAT#ab108615), LONP1 (Abcam, CAT#ab103809), p‐IRE1 (S724) (Abcam, CAT# ab124945), IRE1 (Abcam, CAT# ab37037), total PERK (t‐PERK; Cell Signaling Technology, CAT#C33E10), p‐PERK (Thr982, Affinity Biosciences, CAT#DF7576), and β‐actin (Santa Cruz Biotechnology, CAT# sc‐8432). Uncropped, continuous scan images of Western blot membranes are provided as Supporting Information.

### Oxygen Consumption Rate

Mitochondria were isolated from the cerebral cortex as previously described.^[^ ^86^ ^]^ In brief, the cerebral cortex was homogenized in mitochondrial isolation buffer (70 mm sucrose, 210 mm mannitol, 5 mm HEPES, 1 mm EGTA, and 0.5% [w/v] fatty acid‐free BSA [pH 7.2]) using a Teflon‐glass homogenizer (Thomas Scientific, USA). Following centrifugation at 600 × g for 10 min at 4 °C, and at 8,000 × g for 10 min at 4 °C, the mitochondrial fraction was resuspended in mitochondrial isolation buffer. Protein concentration was determined using the Bradford assay (Bio‐Rad). Aliquots containing 2 µg/25 µL or 20 µg/50 µL of protein were diluted with mitochondrial assay solution (70 mm sucrose, 220 mm mannitol, 10 mm KH_2_PO_4_, 5 mm MgCl_2_, 2 mm HEPES, 1 mm EGTA, 0.2% [w/v] fatty acid‐free BSA, 10 mm succinate, and 2 µm rotenone [pH 7.2]) and seeded in Seahorse XFe96/XF Pro Cell Culture Microplates or XF‐24 plates (Seahorse Bioscience, USA). The plates were centrifuged at 2,000 × g for 20 min at 4 °C using a swinging‐bucket microplate adaptor (Eppendorf, Germany). After adding 180 µL (XFe96/XF Pro) or 450 µL (XF‐24) of mitochondrial assay buffer, the plates were equilibrated at 37 °C for 8–10 min and then transferred to the Seahorse XF pro or Seahorse XF‐24 extracellular flux analyzer (Seahorse Bioscience). Mitochondrial respiration was assessed by measuring the oxygen consumption rate (OCR). Oxygen consumption measurements were obtained under five sequentially induced respiratory states: i) basal respiration; ii) State3 (coupled respiration), with addition of ADP (4 mm) for measuring oxygen consumption during ATP production; iii) state4o, with addition of oligomycin (2.5 µg mL^−1^) for measuring oxygen consumption from protein leakage; iv) state3u (uncoupled respiration), with addition of carbonyl cyanide m‐chlorophenyl hydrazine (CCCP, 4 µm) for measuring oxygen consumption during maximal respiration; and v) non‐mitochondrial respiration, with addition of antimycin (4 µm). OCR was automatically calculated and recorded using the Seahorse wave pro or Seahorse XF‐24 software (Seahorse Bioscience).

### Real‐Time Polymerase Chain Reaction

Total RNA was extracted from cortical tissue, and complementary DNA (cDNA) was synthesized using a High‐Capacity cDNA Reverse Transcription Kit (Invitrogen, CA, USA). Quantitative reverse‐transcription polymerase chain reaction (RT–qPCR) was performed on a real‐time ExiCycle system (BIONEER, Daejeon, Korea) using 100 ng of cDNA, 2× SYBR mix, and forward and reverse primers (3 pmol each). Gene expression profiling was performed using the AccuTarget qPCR Screening Kit (BIONEER, Daejeon, Korea) to assess the expression of 84 mitochondrial‐related genes and 83 genes related to mitochondrial energy metabolism. Expression of genes involved in UPR^mt^ were measured using the following primer pairs: *Lonp1*, 5’‐GAC CAT TCC GGG ATA TCA TCG‐3’ (forward) and 5’‐CGATGATATCCCGAATGGTC‐3’ (reverse); *mtDNAj*, 5’‐GGA TAG GCG AGA GGC TGG‐3’ (forward) and 5’‐CCA GCC TCT CGC CTA TCC‐3’ (reverse); *Atf5*, 5’‐CAA GGA TCC TCG GAT CTT‐3’ (forward) and 5’‐AAG GCG AAG GTG GAG GAC‐3’ (reverse); *Hsp60*, 5’‐AAA TGC TTC GAC TAC CCA CAG‐3’ (forward) and 5’‐CTG TGG GTA GTC GAA GCA TTT‐3’ (reverse); *Hsp10*, 5’‐AAG TTT CTT CCG CTC TTT GACA‐3’ (forward) and 5’‐TGT CAA AGA GCG GAA GAA ACT T‐3’ (reverse); *Clpp*, 5’‐GAG TCA GCA ATG GAG AGG GA‐3’ (forward) and 5’‐TCC CTC TCC ATT GCT GAC TC‐3’ (reverse); and *GDF15*, 5’‐AGG ACC TGC TAA CCA GGC TG‐3’ (forward) and 5’‐TCT GGC GTG AGT ATC CGG AC‐3’ (reverse). Because *Atf5* has two splice variants, mRNA levels of total *Atf5*, *Atf5‐α*, and *Atf5‐β* were quantified separately to confirm the loss of *Atf5* expression in *Atf5* cKO mice^[^ ^87^ ^]^: total *Atf5*, 5’‐GTC TTC ACC CAG CTG AAC AAT‐3’ (forward) and 5’‐AAT GGA GGC TGC ACC AAC‐3’ (reverse); *Atf5‐ɑ*, 5’‐GTT GCC TCC TCG CCT TTT‐3’ (forward) and 5’‐GGA GGC TGC ACC AAC AAT‐3’ (reverse); and *Atf5‐β*, 5’‐TTT TAT GAA GAG GAA TAA GAT GAG GTC‐3’ (forward) and 5’‐GGA GGC TGCA CCA ACA AT‐3’ (reverse). mRNA expression levels were normalized using β‐actin as a housekeeping gene: *β‐actin*, 5’‐GGC TGT ATT CCC CTC CAT CG‐3’ (forward), and 5’‐CCA GTT GGT AAC AAT GCC ATG T‐3’ (reverse).

### Immunohistochemistry (IHC)

Mice were transcardially perfused with phosphate‐buffered saline (PBS), followed by 4% paraformaldehyde (PFA) in PBS. Whole brains were dissected and post‐fixed in 4% PFA overnight at 4 °C, then transferred to a 30% sucrose solution for cryoprotection prior to sectioning. Coronal brain sections (30 µm thick) were obtained using a cryostat and washed three times with PBS (5 min each). Brain slices were blocked in a solution containing 3% Tween 20 and 2% bovine serum albumin (BSA) in PBS for 1 h. Slices were rinsed with PBS and incubated overnight at 4 °C with antibodies against TOMM40 (1:500, ThermoFisher), NeuN (1:500, Sigma), CAMKII alpha (1:500, Santa Cruz), and ATF5 (1:500, Abcam), both diluted in the blocking solution. After washing with PBS three times for 5 min each, the sections were incubated with a fluorescently labeled secondary antibody for 2 h at RT in the dark. Subsequently, sections were washed with PBS three times for 5 min each and incubated with DAPI (4′,6‐diamidino‐2‐phenylindole, 1:1000 in PBS) for 15 min. Finally, the sections were washed with PBS three times for 5 min each and mounted using fluorescence‐compatible mounting medium. Fluorescent images were acquired using an LSM 980 confocal microscope (ZEISS, Oberkochen, Germany)

### Primary Neuron Culture

Cortical neurons were harvested from postnatal day 1 *Atf5* ^fl/fl^ and *Emx1^cre/+^*; *Atf5* ^fl/fl^ mice. Cortical tissues were enzymatically dissociated using papain, and the resulting cell suspension was plated onto poly‐D‐lysine–coated, 18‐mm glass coverslips in 60‐mm Petri dishes containing plating medium, consisting of Neurobasal‐A medium supplemented with 2% B‐27, 10% FBS, 1% GlutaMax, and 1 mm sodium pyruvate (all from Thermo Fisher Scientific). After 4 h, the plating medium was replaced with the same medium lacking FBS; thereafter, 50% of the medium was replaced every 7 days.

### Immunocytochemistry

Cells were fixed by aspirating the culture media, washing with ice‐cold PBS, and adding a fixation buffer containing 4% formaldehyde and 4% sucrose in PBS. After incubating at RT for 15 min, the cells were washed three times with ice‐cold PBS for 5 min each. Cells were permeabilized by incubating with 0.2% Triton X‐100 in PBS at RT for 10 min. Cells were then washed three times with ice‐cold PBS and blocked for 1 h at RT with a blocking solution containing donkey serum (GeneTex, Cat No.GTX30972). Primary antibody hybridization was carried out by incubating the primary antibody (diluted in blocking solution) overnight at 4 °C. The following day, the primary antibody solution was removed, and cells were washed three times with ice‐cold PBS for 10 min each. For secondary antibody hybridization, cells were incubated with secondary antibody (diluted in blocking solution containing donkey serum) at 37 °C for 2 h in the dark. After washing three times with ice‐cold PBS (10 min each), cells were coverslip‐mounted with Faramount mounting medium followed by sealing with clear nail polish. Slides were dried for 24 h at RT in the dark prior to fluorescence microscopy analysis and stored at 4 °C in dark chambers for long‐term preservation.

### Serum GDF15 Measurements

Blood samples were collected from mice via cardiac puncture under anesthesia (≈5% sevoflurane). Typically, 0.5 mL of blood was obtained from each mouse. The blood was allowed to clot at RT for 30 min, and the serum was then separated at 2,000 x g for 15 min at 4 °C. The supernatant was carefully transferred to fresh tubes and stored at −80 °C until analysis. Serum GDF15 concentrations were measured using the Mouse GDF15 Quantikine ELISA Kit (R&D Systems, Cat# MGD150) according to the manufacturer's instructions.

### Virus Packaging and Stereotaxic Brain Injection

Full‐length mouse *Atf5* (Cat #MR203843, Origene) was subcloned into pAAV‐hSyn‐DIO‐EGFP (Addgene, plasmid #50457) to produce the pAAV‐hSyn‐DIO‐ATF5 construct. AAVs (serotype PHP.eB) were packaged as previously described.^[^ ^88^ ^]^ The final virus solution was aliquoted and stored at −80 °C. For stereotaxic brain injections, mice were anesthetized with 2.5% sevoflurane and then secured in a stereotaxic apparatus (RWD, Shenzhen, China). During the procedure, a continuous flow of 2.5% sevoflurane was maintained with fresh gas supplied at 4 L min^−1^ (FiO_2_, 0.4). Coordinates relative to Bregma were AP −0.9 mm, ML, ± 3.0 mm, and DV −1.5 mm.^[^ ^89^ ^]^ AAVs were infused at a rate of 0.1 µL min^−1^, with a post‐injection diffusion time of 8–10 min. For systemic delivery, 200 µL of viral solution (≈2 × 10^11^ vg mL^−1^) was administered via tail vein injection. Mice were placed in a recovery chamber for at least 30 min after injection.

### Statistical Analysis

To ensure objectivity and minimize potential bias, MCAO experiments were conducted under blinded conditions. Specifically, the experimenter performing MCAO surgery, behavior, and subsequent tissue processing/analysis was blinded to mouse genotype and treatment (± sevoflurane). However, certain assays, including Western blot, mitochondrial oxygen consumption rate measurements, and RT‐PCR were performed under unblinded conditions to ensure accurate sample handling, maintain proper loading order, and prevent processing errors. To minimize potential bias, standardized protocols were strictly adhered to across all groups and employed automated quantification methods for objective data analysis.

R statistical software (version 4.2.0; R Core Team, Austria) was used for data analysis. All continuous variables were tested for normality and variance homogeneity. Independent t‐test or one‐way analysis of variance (ANOVA) was applied only if these two conditions were met. In cases where normality was not confirmed, the Kruskal–Wallis test was used. The Welch ANOVA was applied when the variation lacked uniformity. P‐values less than 0.05 were considered significant. Complete statistical results are provided in Supporting Information. All data are presented as means ± standard deviation (SD).

## Conflict of Interest

The authors declare no conflict of interest.

## Author contributions

X.J. and T.Z. are first authors and contributed equally to this work. X.J. and T.Z. performed the conceptualization, data curation, formal analysis, investigation, methodology, project administration, validation, visualization, and writing–original draft. J.C., Y.L., S.L., H.M.K., J.P., and C.H.C. contributed to investigation and methodology. B.H. was involved in data curation, formal analysis, and methodology. H.S.Yi, J.Y.H., and W.C. contributed to conceptualization, resources, supervision, funding acquisition, investigation, project administration, and writing–review and editing.

## Supporting information



Supporting Information

Supporting Information

Supporting Information

Supplemental Table 1

Supplemental Table 2

Supplemental Table 3

Supplemental Table 4

Supplemental Table 5

Supplemental Table 6

Supplemental Table 7

## Data Availability

The data that support the findings of this study are available in the supplementary material of this article.
