# Supplementary material for: ATF5‐Dependent GDF15 Expression Mediates Anesthesia‐Induced Neuroprotection Against Stroke
Source: Adv Sci (Weinh). 2025 Nov 26;13(3):e17086. doi: 10.1002/advs.202417086 (PMC12806220; doi:10.1002/advs.202417086)
Supplement: Supplementary file 1 — Supporting Information [file ADVS-13-e17086-s003.pdf]

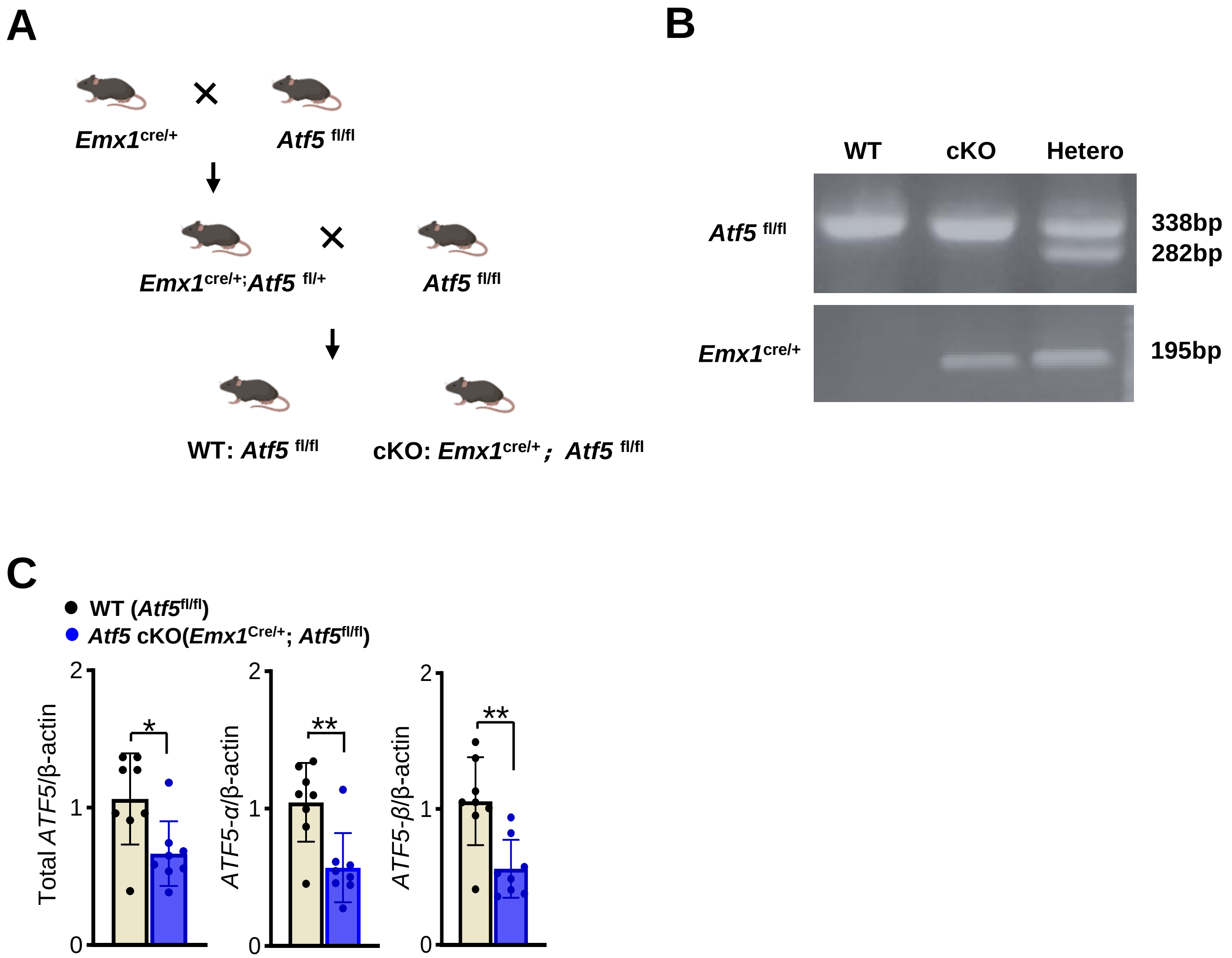

Supplementary Figure 1

(A) Schematic drawing of the generation of *Atf5* conditional knockout mice (*Atf5* cKO). **(B)** Genotyping results with two bands indicating heterozygous mice. **(C)** mRNA expression levels of total *Atf5*, *Atf5-α*, and *Atf5-β* in the cortices of WT and *Atf5* cKO mice, n = 8 per group.

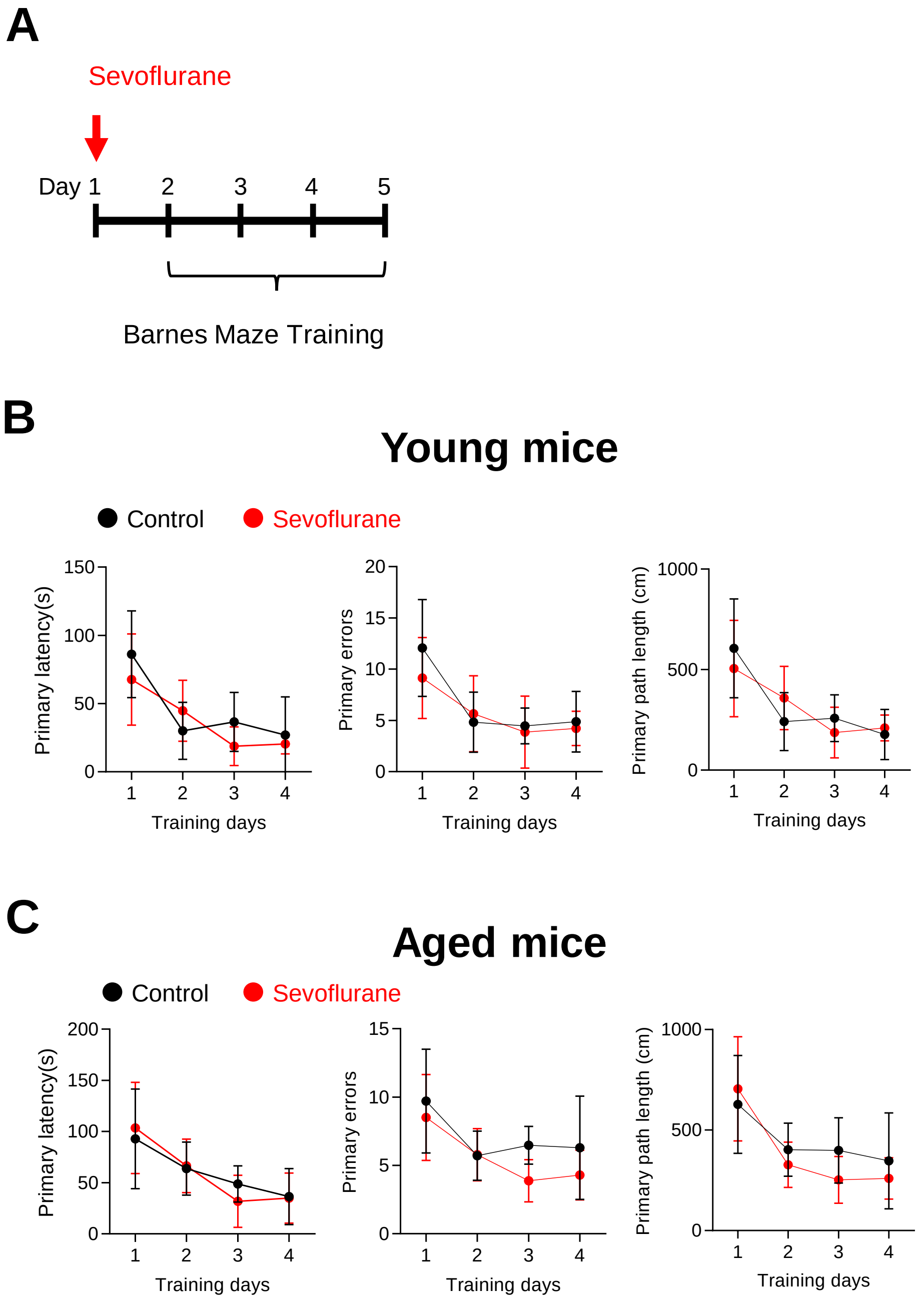

Supplementary Figure 2

(A) Experimental timeline of Barnes Maze Test. **(B-C)** Sevoflurane exposure did not affect primary latency ,errors, or path length during training sessions in both young and aged mice. n =7-10 per group.

A

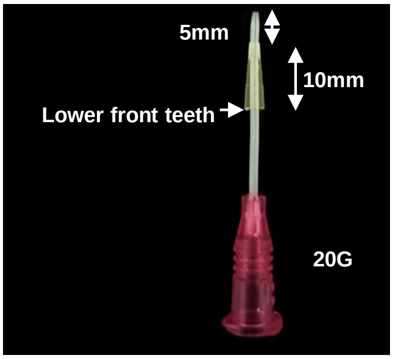

B

	pH	pCO_2 mmHg	pO_2 mmHg	sO_2 %
Animal 1	7.25	46.6	73	92
Animal 2	7.35	42.4	94	97
Animal 3	7.21	46.8	73	91
Animal 4	7.28	48.4	68	91
Mean±SD	7.27±0.05	46.05±0.22	77±10.02	92.75±2.49

Supplementary figure 3

(A) Customized endotracheal tube for mice. **(B)** Blood gas analysis using trunk blood obtained after decapitation with ventilation conditions used for MCAO surgery. pCO2, partial pressure of carbon dioxide; pO2, partial pressure of oxygen; sO2, oxygen saturation.

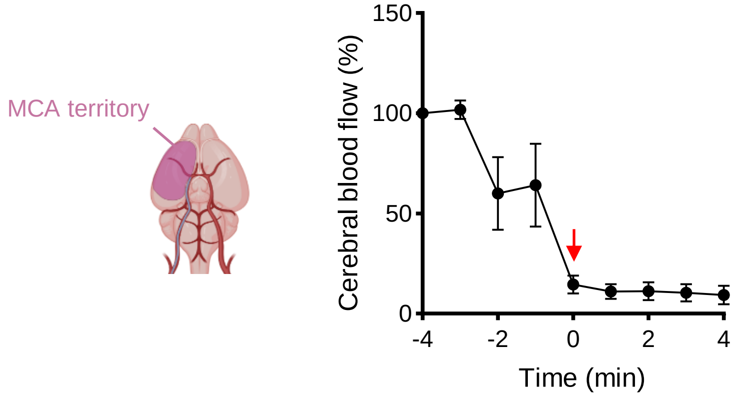

Supplementary figure 4. Reduction in cerebral blood flow in the MCA territory during the ischemic period (n = 6).
