# Supplementary material for: ATF5‐Dependent GDF15 Expression Mediates Anesthesia‐Induced Neuroprotection Against Stroke
Source: Adv Sci (Weinh). 2025 Nov 26;13(3):e17086. doi: 10.1002/advs.202417086 (PMC12806220; doi:10.1002/advs.202417086)
Supplement: Supplementary file 2 — Supporting Information [file ADVS-13-e17086-s002.pdf]

Statistic data-Data analysis using R


Fig1C_cortex infarct volume_B6 1
Fig1C_neurological score_B6 3
Fig1D B6_CLPP 4
Fig1D B6_HSP60 5
Fig1D B6_HSP70 6
Fig1D B6_LONP1 7
Fig1D_B6_ATF5 9
Fig1E B6_OCR_Basal 11
Fig1E B6_OCR_State3 12
Fig1E B6_OCR_State3u 13
Fig1E B6_OCR_State4o 14
Fig 2A_ATF5 intensity_ATF5 cKO 15
Fig 2D_cortex infarct volume_ATF5 cKO 17
Fig 2D_neurological score_ATF5 cKO 19
Fig 2E_ATF5_WT 21
Fig 2E_CLPP_WT 23
Fig 2E_GDF15_WT 24
Fig 2E_HSP10_WT 25
Fig 2E_HSP60_WT 27
Fig 2E_LONP1_WT 29
Fig 2E_mtDNAj_WT 30
Fig 2F_ATF5_ATF5 cKO 32
Fig 2F_CLPP_ATF5 cKO 33
Fig 2F_GDF15_ATF5 cKO 34
Fig 2F_HSP10_ATF5 cKO 35
Fig 2F_HSP60_ATF5 cKO 36
Fig 2F_LONP1_ATF5 cKO 37
Fig 2F_mtDNAj_ATF5 cKO 38
Fig 2H_ATF5 cKO_ATF5 39
Fig 2H_ATF5 cKO_CLPP 40
Fig 2H_ATF5 cKO_HSP60 41
Fig 2H_ATF5 cKO_HSP70 42
Fig 2H_ATF5 cKO_LONP1 43
Fig 2I_ATF5 cKO_OCR_Basal 44
Fig 2I_ATF5 cKO_OCR_state3 46
Fig 2I_ATF5 cKO_OCR_state3u 48
Fig 2I_ATF5 cKO_OCR_state4o 50
Fig 3B_cortex infarct volume_GDF15 KO 52
Fig 3B_neurological score_GDF15 KO 54
Fig3 E_GDF15 KO_OCR_Basal 56
Fig3 E_GDF15 KO_OCR_state3 58
Fig3 E_GDF15 KO_OCR_state3u 60
Fig3 E_GDF15 KO_OCR_state4o 62
Fig3 G_GDF15 KO_ATF5 64
Fig3 G_GDF15 KO_CLPP 66
Fig3 G_GDF15 KO_HSP60 67
Fig3 G_GDF15 KO_HSP70 68
Fig3 G_GDF15 KO_LONP1 69
Fig3 H_serum GDF15_B6 70
Fig3 I_serum GDF15_ATF5 WT 71
Fig3 J_serum GDF15_ATF5 cKO 72
Fig3 K_serum GDF15_B6_WT_ATF5 cKO_post 0H 74
Fig4 B_virus cortex injection_ATF5 75
Fig4 B_virus cortex injection_CLPP 77

Fig4 B_virus cortex injection_HSP60 77
Fig4 B_virus cortex injection_HSP70 79
Fig4 B_virus cortex injection_LONP1 80
Fig4 D_ATF5 virus_OCR_Basal 81
Fig4 D_ATF5 virus_OCR_State3 83
Fig4 D_ATF5 virus_OCR_State3u 84
Fig4 D_ATF5 virus_OCR_State4o 86
Fig5 C_virus iv injection_ATF5 88
Fig5 D_cortex -qPCR_GDF15_Emx1 cre_ATF5 virus IV 90
Fig5 E _serum level of GDF15 virus iv injection-Elisa 92
Fig5 G_cortex infarct volume_Emx1 cre_ATF5 virus IV 93
Fig5 G_neurological score_Emx1 cre_ATF5 virus IV 94
Fig6 A_2,8 mon_ATF5 96
Fig6 A_2,14 mon_ATF5 97
Fig6 A_2,20 mon_ATF5 98
Fig6 B qPCR_GDF15__2,8,14,20mon 99
Fig6 C_2,8,14,20_OCR_Basal 100
Fig6 C_2,8,14,20_OCR_state3 102
Fig6 C_2,8,14,20_OCR_state3u 104
Fig6 C_2,8,14,20_OCR_state4o 106
Fig6 E aging_ATF5 108
Fig6 E aging_CLPP 109
Fig6 E aging_HSP60 110
Fig6 E aging_HSP70 111
Fig6 E aging_LONP1 112
Fig6 G Aging_OCR_Basal 113
Fig6 G Aging_OCR_State3 115
Fig6 G Aging_OCR_State3u 117
Fig6 G Aging_OCR_State4o 119

# Data analysis using R

```
## Present data is ** Fig1C_cortex infarct volume_B6.csv **
##
## **  Data structure  **
## 'data.frame':    16 obs. of  3 variables:
##  $ subject: int  1 2 3 4 5 6 7 8 9 10 ...
##  $ group  : chr  "con" "con" "con" "con" ...
##  $ infarct: num  0.715 0.548 0.591 0.505 0.526 ...
##
## ** Explorative data analysis with graphics**
```

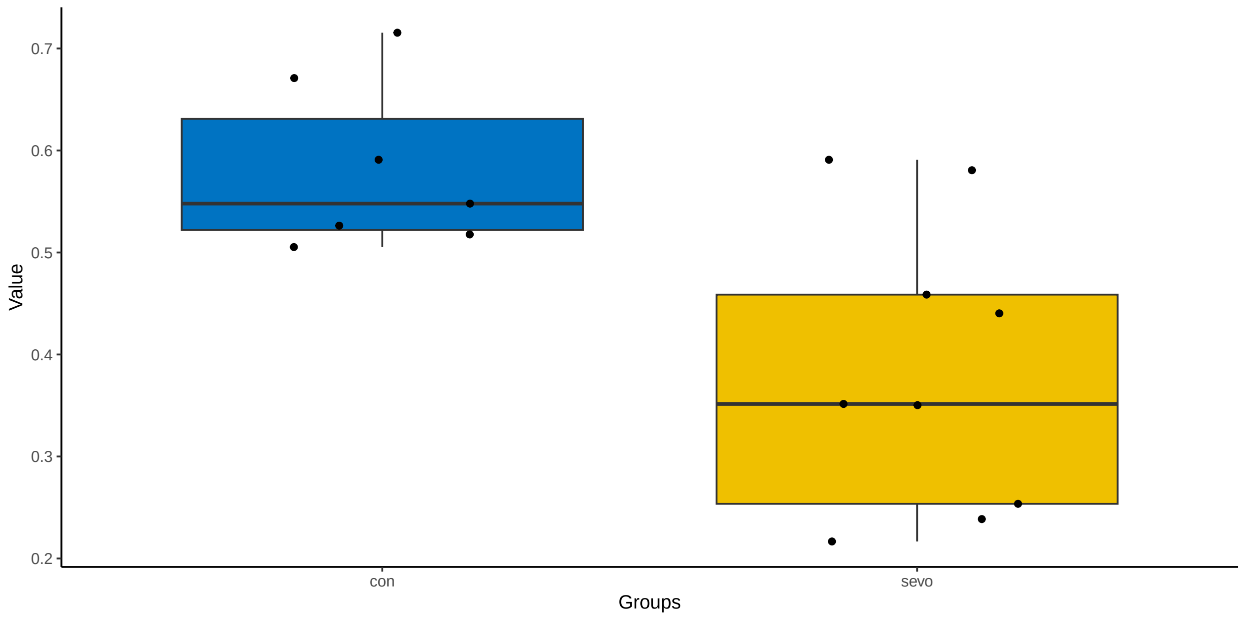


```
## 1. Normality assumption test by Shapiro_Wilk test is
##  p = 0.501
##  Normality assumption was not rejected
## 2. Equal variance test by Bartlett test is
##  p = 0.190
##  Equal variance assumption was not rejected
## 3. The result of anova is
##  p = 0.006
##  A statistically significant difference exist between groups
##
##   Tukey multiple comparisons of means
##     95% family-wise confidence level
##
## Fit: aov(formula = d1[, 3] ~ d1[, 2], data = d1)
##
```

```
## $`d1[, 2]`
##                diff        lwr         upr     p adj
## sevo-con -0.1952463 -0.3239474 -0.06654529 0.0057694
```

# Data analysis using R

```
## Present data is ** Fig1C_neurological score_B6.csv **
##
## **  Data structure  **
## 'data.frame':    16 obs. of  3 variables:
##  $ subject: int  1 2 3 4 5 6 7 8 9 10 ...
##  $ group  : chr  "con" "con" "con" "con" ...
##  $ score  : int  3 3 3 3 3 3 3 3 3 2 ...
##
## ** Explorative data analysis with graphics**
```

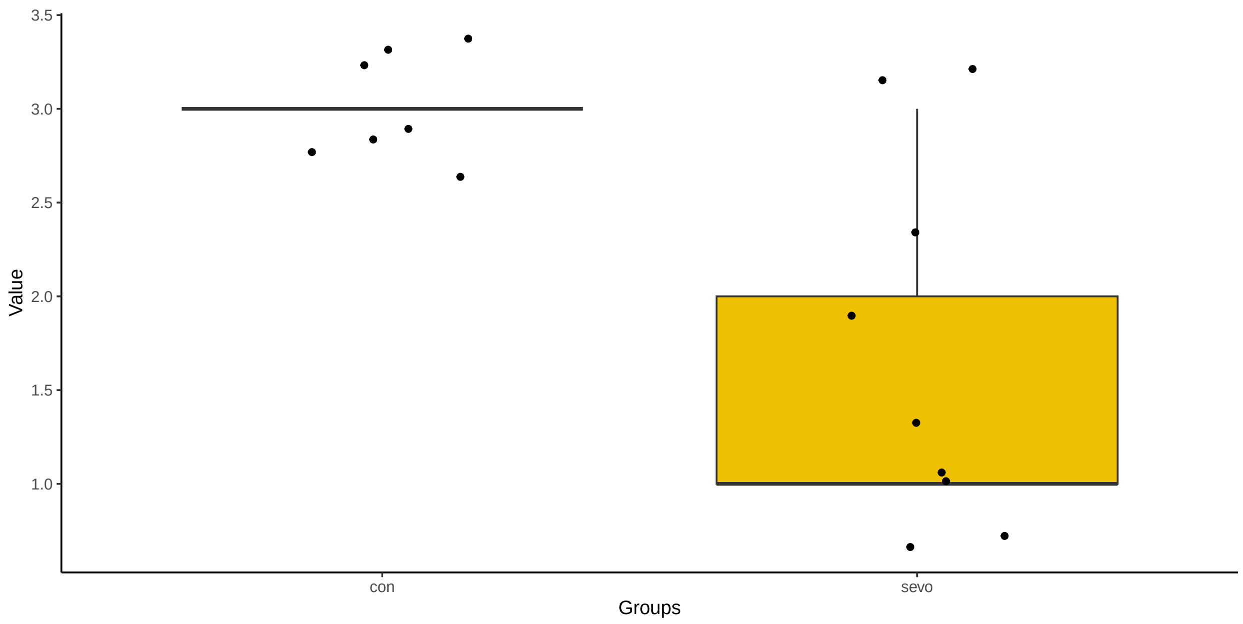


```
## 1. Normality assumption test by Shapiro_Wilk test is
##  p = 0.004
##  Normality assumption was rejected
## 2. The result of Kruskall_Wallis test:
##  p = 0.004
##  A statistically significant difference exist between groups
##
```

# Data analysis using R

```
## Present data is ** Fig1D B6_CLPP.csv **
##
## **  Data structure  **
## 'data.frame':    10 obs. of  3 variables:
##  $ subject: int  1 2 3 4 5 6 7 8 9 10
##  $ group  : chr  "con" "con" "con" "con" ...
##  $ CLPP   : num  0.774 1.098 1.203 1.031 0.893 ...
##
## ** Explorative data analysis with graphics**
```

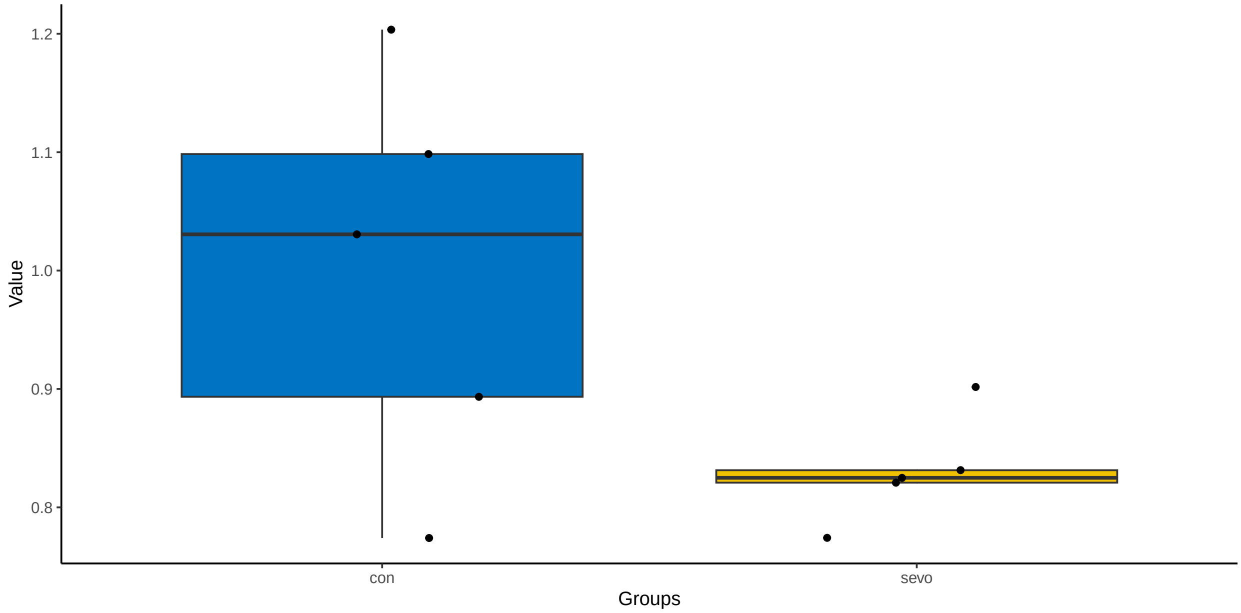


```
## 1. Normality assumption test by Shapiro_Wilk test is
##  p = 0.946
##  Normality assumption was not rejected
## 2. Equal variance test by Bartlett test is
##  p = 0.027
##  Equal variance assumption was rejected
## 3. The result of Welch ANOVA is
##  p = 0.088
##  A statistically significant difference do not exist between groups
##
```

# Data analysis using R

```
## Present data is ** Fig1D B6_HSP60.csv **
##
## **  Data structure  **
## 'data.frame':    10 obs. of  3 variables:
##  $ subject: int  1 2 3 4 5 6 7 8 9 10
##  $ group  : chr  "con" "con" "con" "con" ...
##  $ HSP60  : num  0.773 1.207 0.804 1.038 1.178 ...
##
## ** Explorative data analysis with graphics**
```

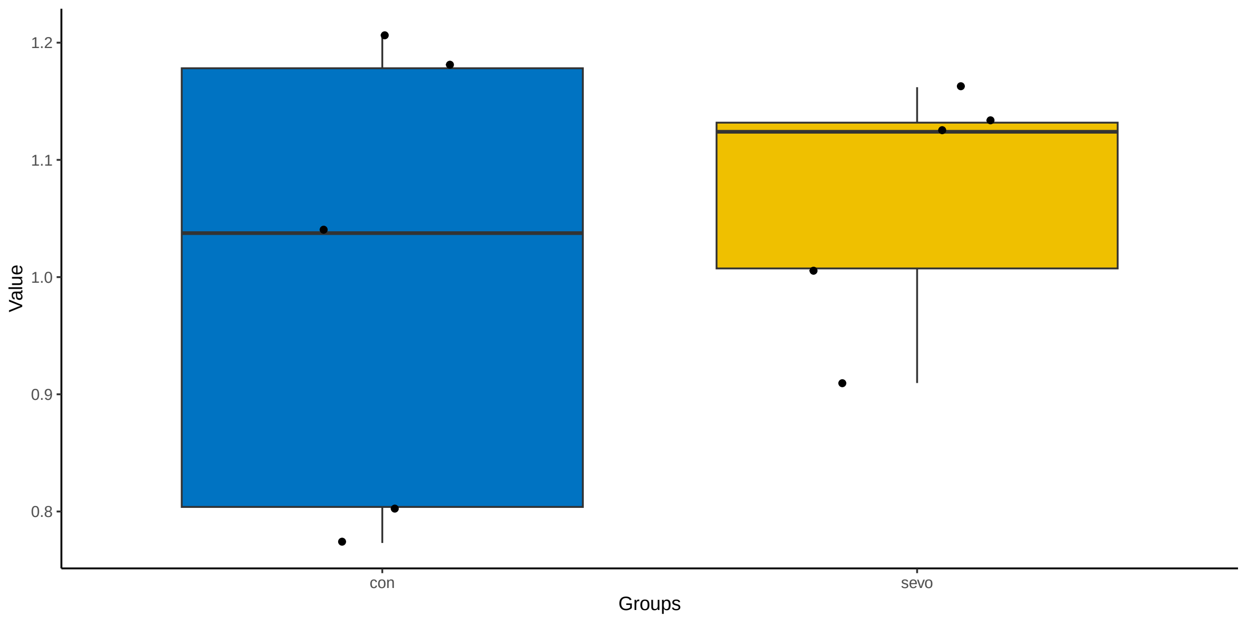


```
## 1. Normality assumption test by Shapiro_Wilk test is
##  p = 0.429
##  Normality assumption was not rejected
## 2. Equal variance test by Bartlett test is
##  p = 0.232
##  Equal variance assumption was not rejected
## 3. The result of anova is
##  p = 0.533
##  A statistically significant difference do not exist between groups
##
```

# Data analysis using R

```
## Present data is ** Fig1D B6_HSP70.csv **
##
## **  Data structure  **
## 'data.frame':    10 obs. of  3 variables:
##  $ subject: int  1 2 3 4 5 6 7 8 9 10
##  $ group  : chr  "con" "con" "con" "con" ...
##  $ HSP70  : num  0.869 1.004 0.92 1.125 1.082 ...
##
## ** Explorative data analysis with graphics**
```

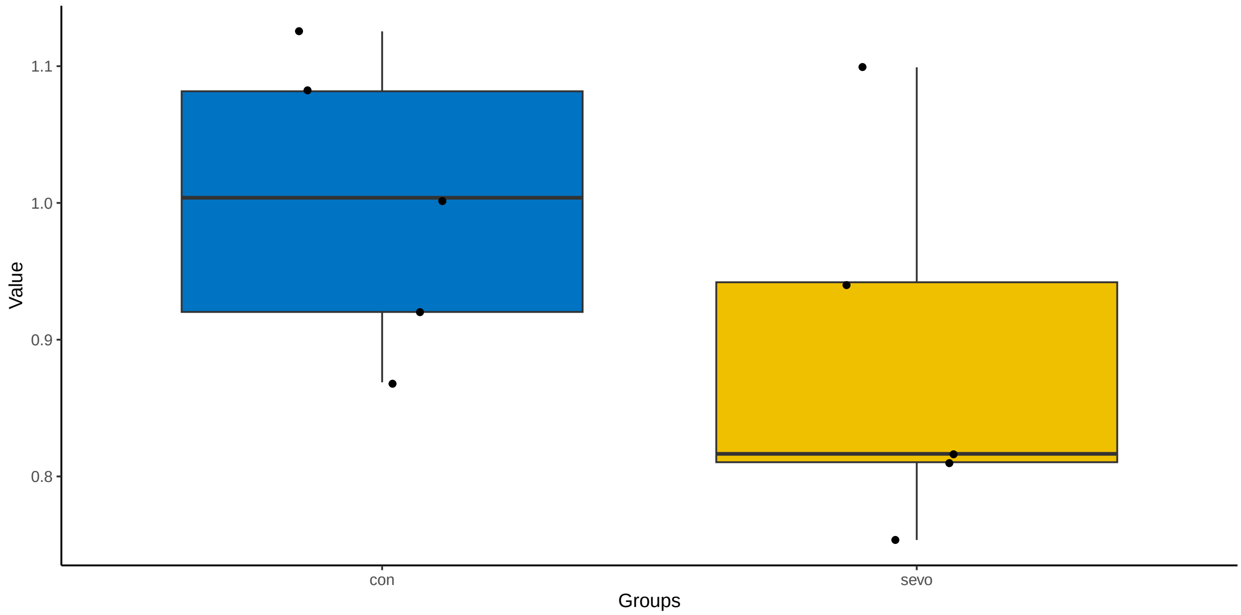


```
## 1. Normality assumption test by Shapiro_Wilk test is
##  p = 0.382
##  Normality assumption was not rejected
## 2. Equal variance test by Bartlett test is
##  p = 0.632
##  Equal variance assumption was not rejected
## 3. The result of anova is
##  p = 0.178
##  A statistically significant difference do not exist between groups
##
```

# Data analysis using R

```
## Present data is ** Fig1D B6_LONP1.csv **
##
## **  Data structure  **
## 'data.frame':    10 obs. of  3 variables:
##  $ subject: int  1 2 3 4 5 6 7 8 9 10
##  $ group  : chr  "con" "con" "con" "con" ...
##  $ LONP1  : num  0.999 0.665 0.981 1.097 1.259 ...
##
## ** Explorative data analysis with graphics**
```

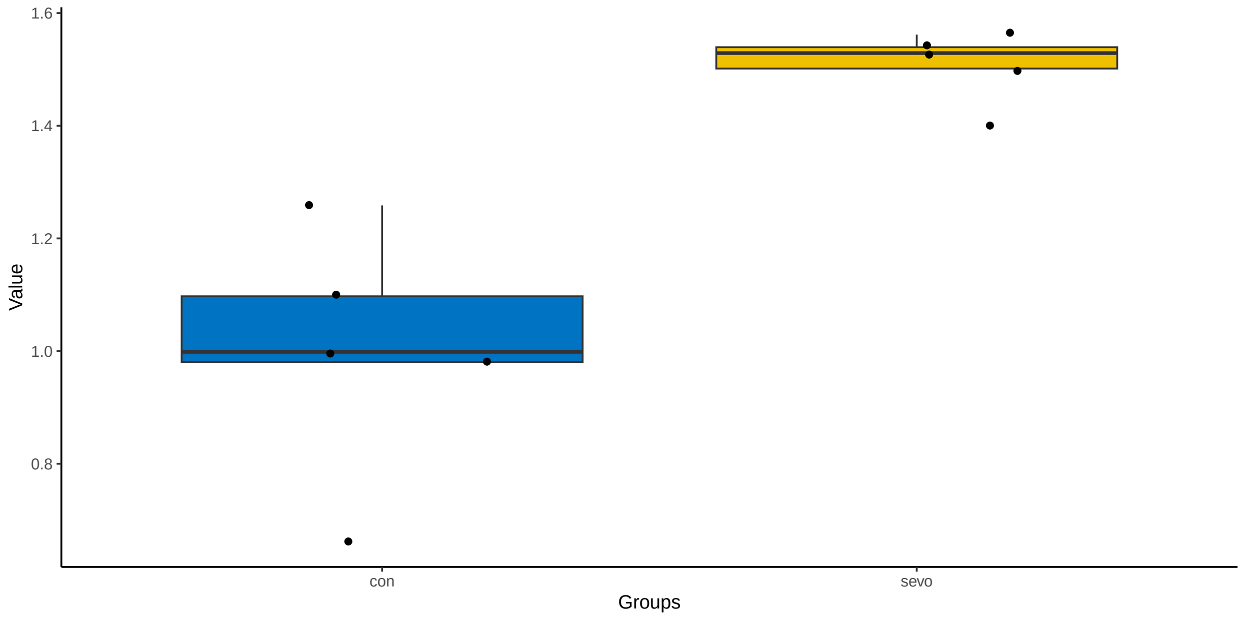


```
## 1. Normality assumption test by Shapiro_Wilk test is
##  p = 0.218
##  Normality assumption was not rejected
## 2. Equal variance test by Bartlett test is
##  p = 0.038
##  Equal variance assumption was rejected
## 3. The result of Welch ANOVA is
##  p = 0.005
##  A statistically significant difference exist between groups
##
##   Tukey multiple comparisons of means
##     95% family-wise confidence level
##
## Fit: aov(formula = d1[, 3] ~ d1[, 2])
##
```

```
## $`d1[, 2]`
##               diff       lwr       upr     p adj
## sevo-con 0.5056593 0.2717981 0.7395205 0.0010713
```

# Data analysis using R

```
## Present data is ** Fig1D_B6_ATF5.csv **
##
## **  Data structure  **
## 'data.frame':    10 obs. of  3 variables:
##  $ subject: int  1 2 3 4 5 6 7 8 9 10
##  $ group  : chr  "con" "con" "con" "con" ...
##  $ ATF5   : num  1.27 0.732 0.578 0.875 1.545 ...
##
## ** Explorative data analysis with graphics**
```

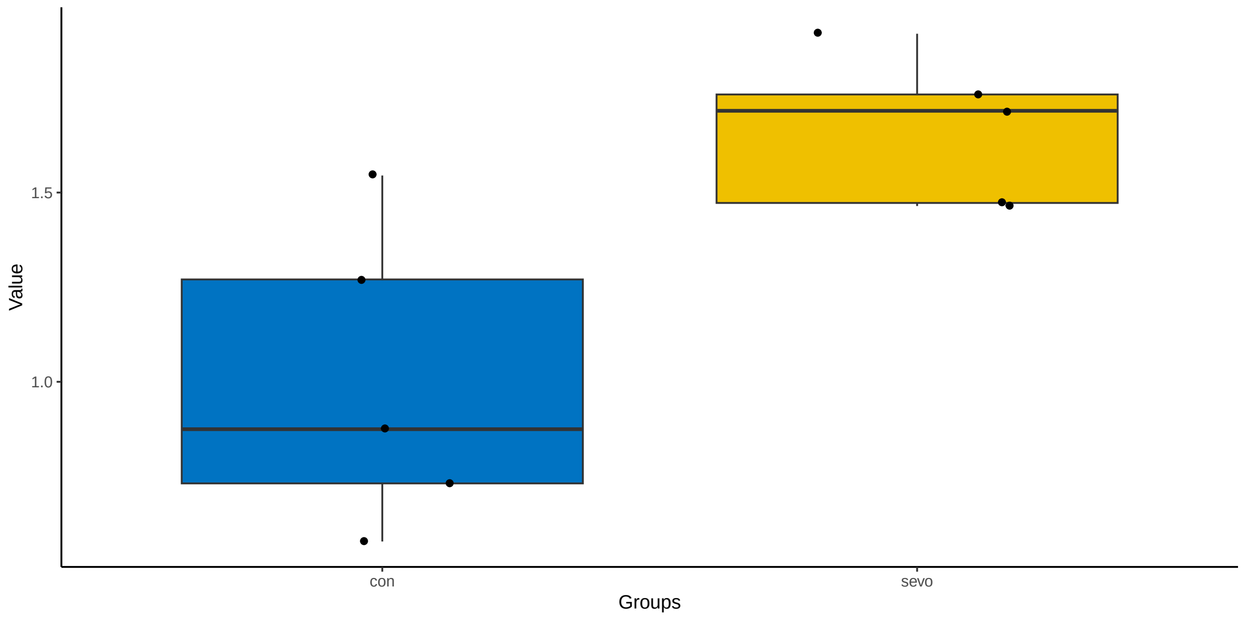


```
## 1. Normality assumption test by Shapiro_Wilk test is
##  p = 0.816
##  Normality assumption was not rejected
## 2. Equal variance test by Bartlett test is
##  p = 0.197
##  Equal variance assumption was not rejected
## 3. The result of anova is
##  p = 0.010
##  A statistically significant difference exist between groups
##
##   Tukey multiple comparisons of means
##     95% family-wise confidence level
##
## Fit: aov(formula = d1[, 3] ~ d1[, 2], data = d1)
##
```

```
## $`d1[, 2]`
##              diff       lwr      upr     p adj
## sevo-con 0.666288 0.2081512 1.124425 0.0100248
```

# Data analysis using R

```
## Present data is ** Fig1 E B6_OCR_Basal.csv **
##
## **  Data structure  **
## 'data.frame':    12 obs. of  3 variables:
##  $ subject: int  1 2 3 4 5 6 7 8 9 10 ...
##  $ group  : chr  "B6" "B6" "B6" "B6" ...
##  $ basal  : num  275 183 269 269 266 ...
##
## ** Explorative data analysis with graphics**
```

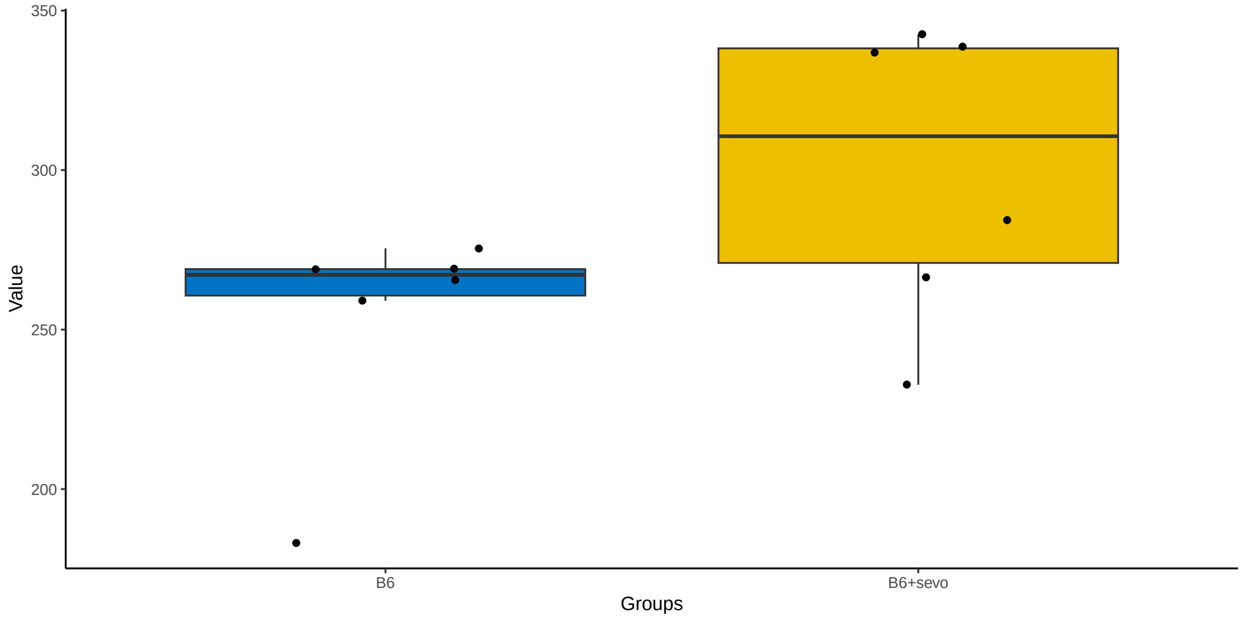


```
## 1. Normality assumption test by Shapiro_Wilk test is
##  p = 0.069
##  Normality assumption was not rejected
## 2. Equal variance test by Bartlett test is
##  p = 0.559
##  Equal variance assumption was not rejected
## 3. The result of anova is
##  p = 0.075
##  A statistically significant difference do not exist between groups
##
```

# Data analysis using R

```
## Present data is ** Fig1E B6_OCR_State3.csv **
##
## **  Data structure  **
## 'data.frame':    12 obs. of  3 variables:
##  $ subject: int  1 2 3 4 5 6 7 8 9 10 ...
##  $ group  : chr  "B6" "B6" "B6" "B6" ...
##  $ state3 : num  522 287 548 423 403 ...
##
## ** Explorative data analysis with graphics**
```

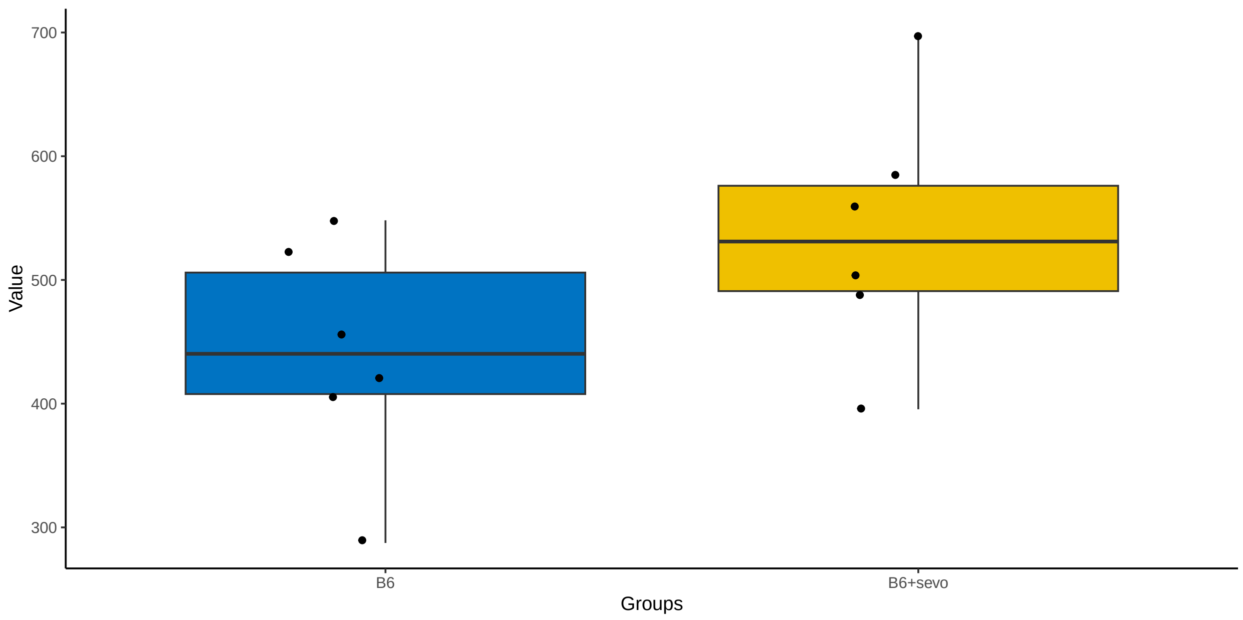


```
## 1. Normality assumption test by Shapiro_Wilk test is
##  p = 0.92
##  Normality assumption was not rejected
## 2. Equal variance test by Bartlett test is
##  p = 0.850
##  Equal variance assumption was not rejected
## 3. The result of anova is
##  p = 0.116
##  A statistically significant difference do not exist between groups
##
```

# Data analysis using R

```
## Present data is ** Fig1 E B6_OCR_State3u.csv **
##
## **  Data structure  **
## 'data.frame':    12 obs. of  3 variables:
##  $ subject: int  1 2 3 4 5 6 7 8 9 10 ...
##  $ group  : chr  "B6" "B6" "B6" "B6" ...
##  $ state3u: num  357 228 449 309 314 ...
##
## ** Explorative data analysis with graphics**
```

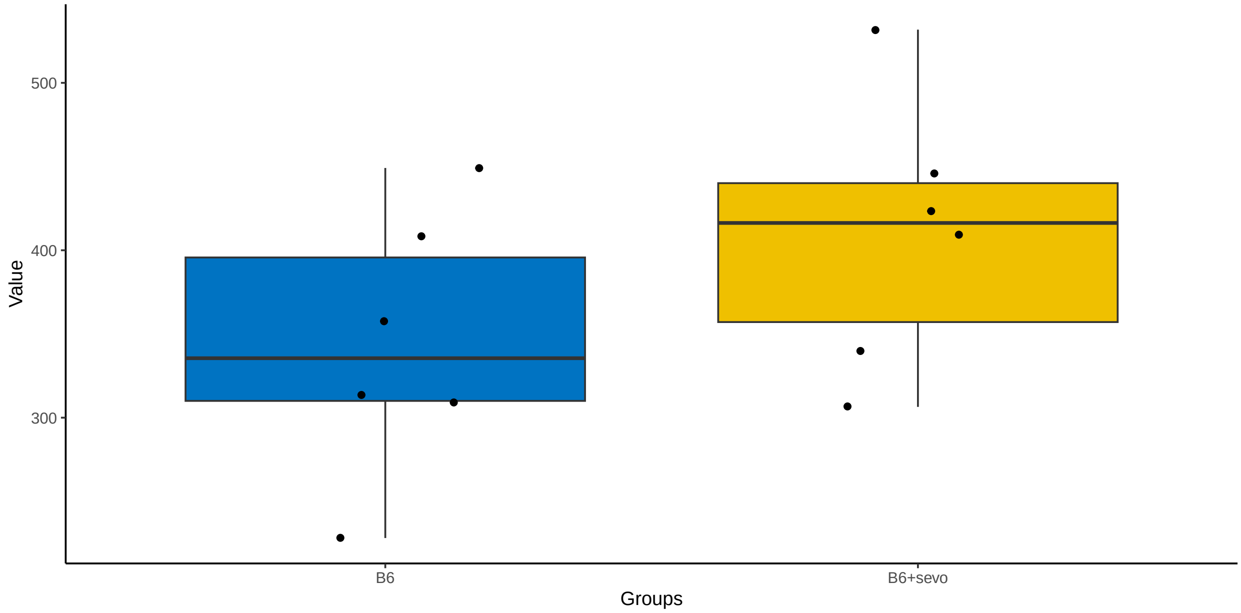


```
## 1. Normality assumption test by Shapiro_Wilk test is
##  p = 0.909
##  Normality assumption was not rejected
## 2. Equal variance test by Bartlett test is
##  p = 0.974
##  Equal variance assumption was not rejected
## 3. The result of anova is
##  p = 0.186
##  A statistically significant difference do not exist between groups
##
```

# Data analysis using R

```
## Present data is ** Fig1E B6_OCR_State4o.csv **
##
## **  Data structure  **
## 'data.frame':    12 obs. of  3 variables:
##  $ subject: int  1 2 3 4 5 6 7 8 9 10 ...
##  $ group  : chr  "B6" "B6" "B6" "B6" ...
##  $ state4o: num  113.1 88.5 123.7 99.4 104.6 ...
##
## ** Explorative data analysis with graphics**
```

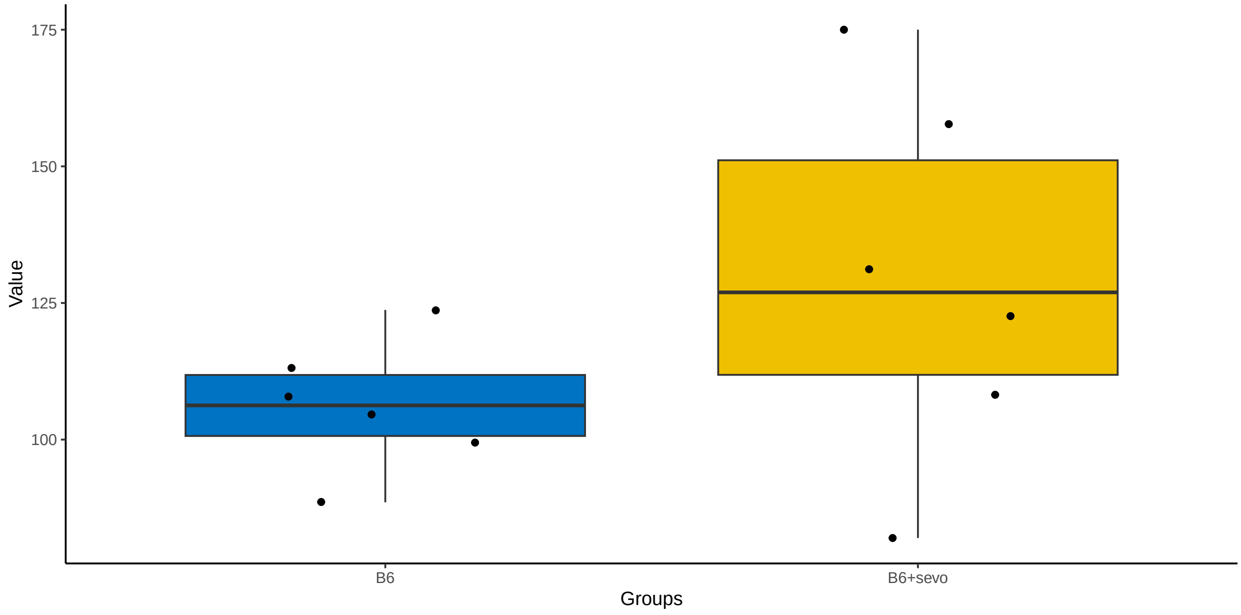


```
## 1. Normality assumption test by Shapiro_Wilk test is
##  p = 0.964
##  Normality assumption was not rejected
## 2. Equal variance test by Bartlett test is
##  p = 0.042
##  Equal variance assumption was rejected
## 3. The result of Welch ANOVA is
##  p = 0.159
##  A statistically significant difference do not exist between groups
##
```

# Data analysis using R

```
## Present data is ** Fig 2A_ATF5 intensity_ATF5 cKO.csv **
##
## **  Data structure  **
## 'data.frame':    36 obs. of  3 variables:
##  $ subject: int  1 2 3 4 5 6 7 8 9 10 ...
##  $ group  : chr  "WT" "WT" "WT" "WT" ...
##  $ ATF5   : num  0.732 0.973 1.121 0.631 0.79 ...
##
## ** Explorative data analysis with graphics**
```

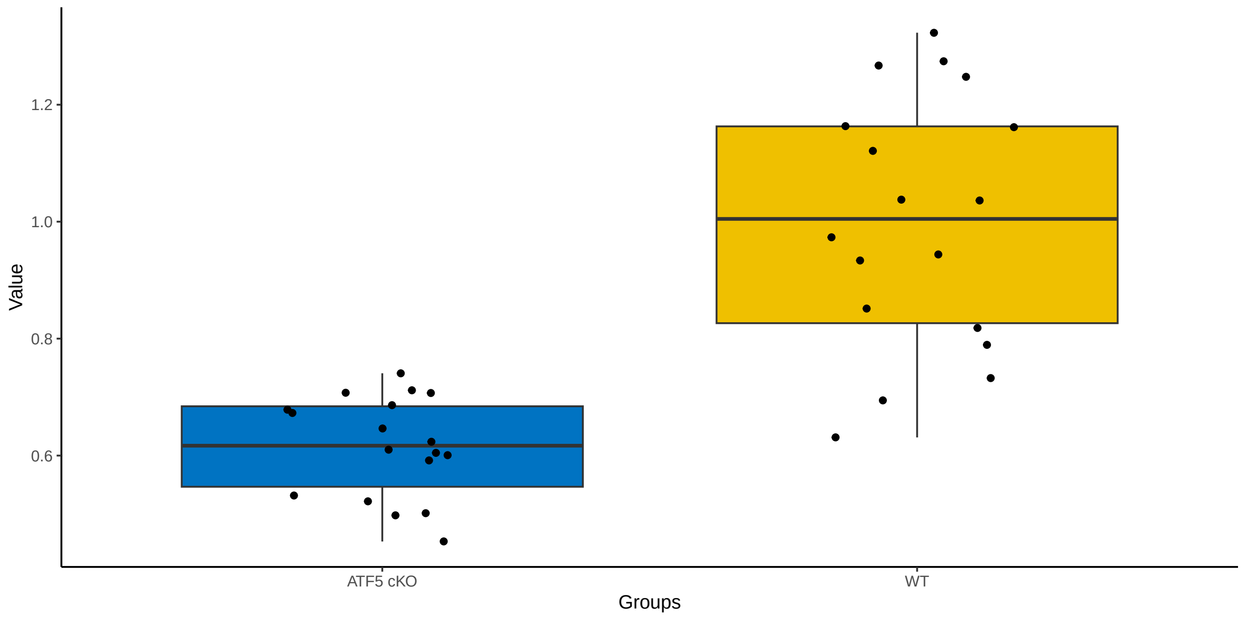


```
## 1. Normality assumption test by Shapiro_Wilk test is
##  p = 0.965
##  Normality assumption was not rejected
## 2. Equal variance test by Bartlett test is
##  p = 0.000
##  Equal variance assumption was rejected
## 3. The result of Welch ANOVA is
##  p = 0.000
##  A statistically significant difference exist between groups
##
##   Tukey multiple comparisons of means
##     95% family-wise confidence level
##
## Fit: aov(formula = d1[, 3] ~ d1[, 2])
##
```

```
## $`d1[, 2]`
##                  diff       lwr       upr p adj
## WT-ATF5 cKO 0.3840011 0.2727891 0.4952131     0
```

# Data analysis using R

```
## Present data is ** Fig 2D_cortex infarct volume_ATF5 cKO.csv **
##
## **  Data structure  **
## 'data.frame':    23 obs. of  3 variables:
##  $ subject: int  1 2 3 4 5 6 7 8 9 10 ...
##  $ group  : chr  "WT" "WT" "WT" "WT" ...
##  $ infarct: num  0.667 0.713 0.653 0.541 0.486 ...
##
## ** Explorative data analysis with graphics**
```

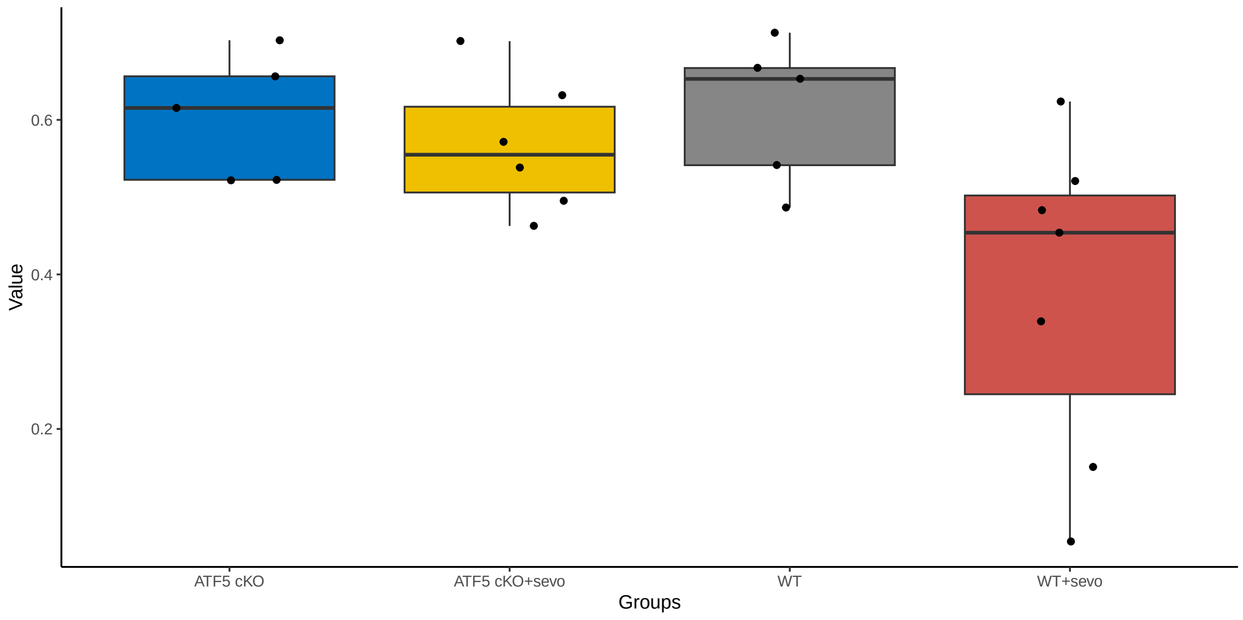


```
## 1. Normality assumption test by Shapiro_Wilk test is
##  p = 0.658
##  Normality assumption was not rejected
## 2. Equal variance test by Bartlett test is
##  p = 0.108
##  Equal variance assumption was not rejected
## 3. The result of anova is
##  p = 0.019
##  A statistically significant difference exist between groups
##
##   Tukey multiple comparisons of means
##     95% family-wise confidence level
##
## Fit: aov(formula = d1[, 3] ~ d1[, 2], data = d1)
##
```

```
## $`d1[, 2]`
##                                  diff        lwr          upr     p adj
## ATF5 cKO+sevo-ATF5 cKO -0.036845994 -0.2700546  0.196362596 0.9699340
## WT-ATF5 cKO             0.008316556 -0.2352619  0.251894984 0.9996711
## WT+sevo-ATF5 cKO       -0.228636469 -0.4541463 -0.003126664 0.0462093
## WT-ATF5 cKO+sevo        0.045162550 -0.1880460  0.278371140 0.9468896
## WT+sevo-ATF5 cKO+sevo  -0.191790474 -0.4060578  0.022476807 0.0891877
## WT+sevo-WT             -0.236953024 -0.4624628 -0.011443219 0.0373813
```

# Data analysis using R

```
## Present data is ** Fig 2D_neurological score_ATF5 cKO.csv **
##
## **  Data structure  **
## 'data.frame':    23 obs. of  3 variables:
##  $ subject: int  1 2 3 4 5 6 7 8 9 10 ...
##  $ group  : chr  "WT" "WT" "WT" "WT" ...
##  $ score  : int  3 3 3 3 3 1 2 1 3 1 ...
##
## ** Explorative data analysis with graphics**
```

```
## 1. Normality assumption test by Shapiro_Wilk test is
##  p = 0.014
##  Normality assumption was rejected
## 2. The result of Kruskall_Wallis test:
##  p = 0.008
##  A statistically significant difference exist between groups
##
```

```
## Dunn (1964) Kruskal-Wallis multiple comparison
```

```
##   p-values adjusted with the Benjamini-Hochberg method.
```

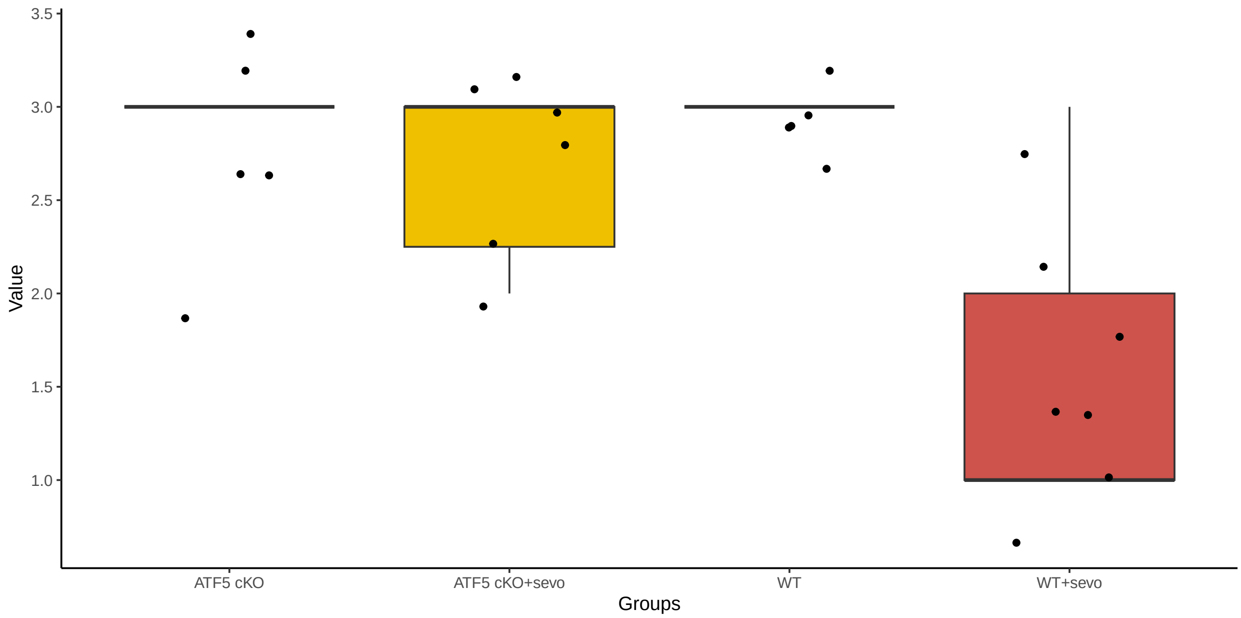


```
##                 Comparison          Z     P.unadj      P.adj
## 1 ATF5 cKO - ATF5 cKO+sevo  0.3536989 0.723564580 0.72356458
```

```
## 2            ATF5 cKO - WT -0.5079613 0.611480477 0.73377657
## 3       ATF5 cKO+sevo - WT -0.8842471 0.376562816 0.56484422
## 4       ATF5 cKO - WT+sevo  2.5452916 0.010918650 0.03275595
## 5  ATF5 cKO+sevo - WT+sevo  2.2938762 0.021797613 0.04359523
## 6             WT - WT+sevo  3.0939526 0.001975091 0.01185054
```

# Data analysis using R

```
## Present data is ** Fig 2E_ATF5_WT.csv **
##
## **  Data structure  **
## 'data.frame':    9 obs. of  3 variables:
##  $ subject: int  1 2 3 4 5 6 7 8 9
##  $ group  : chr  "WT" "WT" "WT" "WT" ...
##  $ ATF5   : num  0.736 0.473 1.578 1.213 1.567 ...
##
## ** Explorative data analysis with graphics**
```

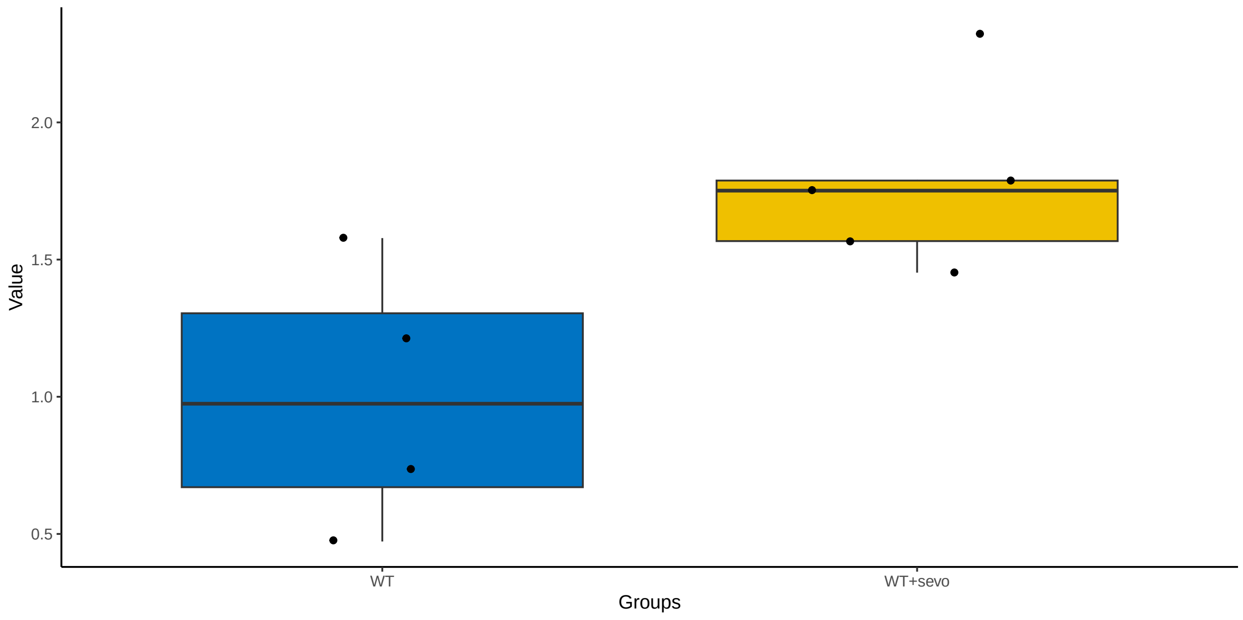


```
## 1. Normality assumption test by Shapiro_Wilk test is
##  p = 0.523
##  Normality assumption was not rejected
## 2. Equal variance test by Bartlett test is
##  p = 0.507
##  Equal variance assumption was not rejected
## 3. The result of anova is
##  p = 0.026
##  A statistically significant difference exist between groups
##
##   Tukey multiple comparisons of means
##     95% family-wise confidence level
##
## Fit: aov(formula = d1[, 3] ~ d1[, 2], data = d1)
##
```

```
## $`d1[, 2]`
##                 diff       lwr      upr     p adj
## WT+sevo-WT 0.777201 0.1259268 1.428475 0.0257055
```

# Data analysis using R

```
## Present data is ** Fig 2E_CLPP_WT.csv **
##
## **  Data structure  **
## 'data.frame':    9 obs. of  3 variables:
##  $ subject: int  1 2 3 4 5 6 7 8 9
##  $ group  : chr  "WT" "WT" "WT" "WT" ...
##  $ CLPP   : num  1.023 1.067 0.929 0.982 0.982 ...
##
## ** Explorative data analysis with graphics**
```

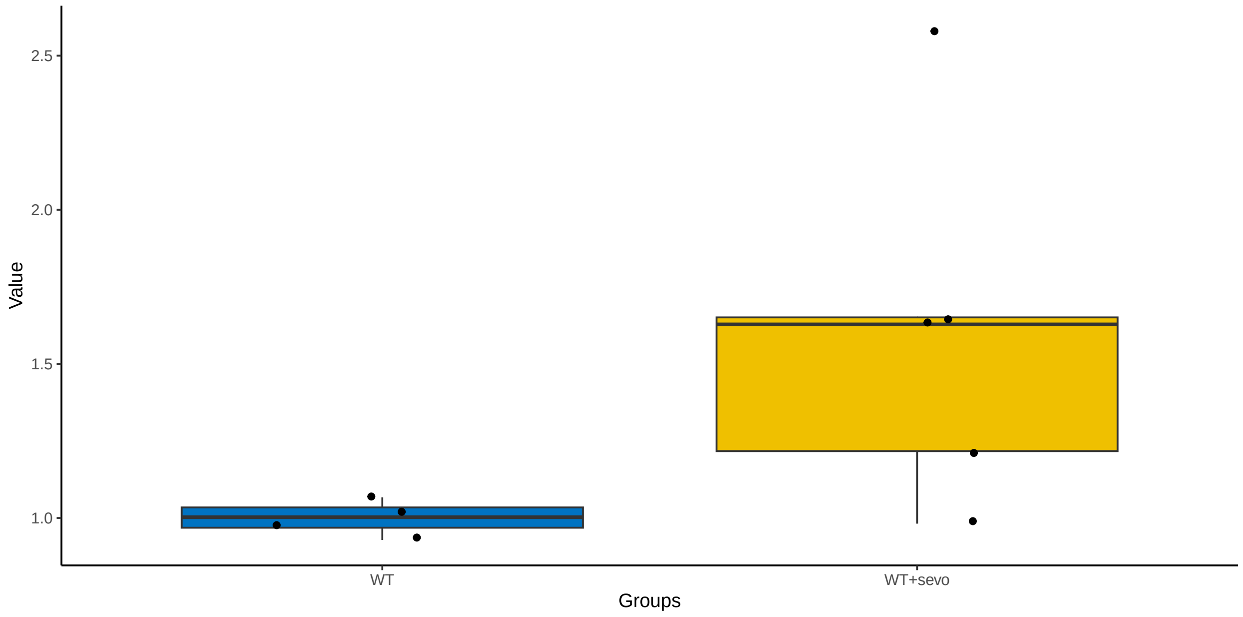


```
## 1. Normality assumption test by Shapiro_Wilk test is
##  p = 0.045
##  Normality assumption was rejected
## 2. The result of Kruskall_Wallis test:
##  p = 0.065
##  A statistically significant difference do not exist between groups
##
```

# Data analysis using R

```
## Present data is ** Fig 2E_GDF15_WT.csv **
##
## **  Data structure  **
## 'data.frame':    17 obs. of  3 variables:
##  $ subject: int  1 2 3 4 5 6 7 8 9 10 ...
##  $ group  : chr  "WT" "WT" "WT" "WT" ...
##  $ GDF15  : num  1.095 1.548 0.927 1.008 0.908 ...
##
## ** Explorative data analysis with graphics**
```

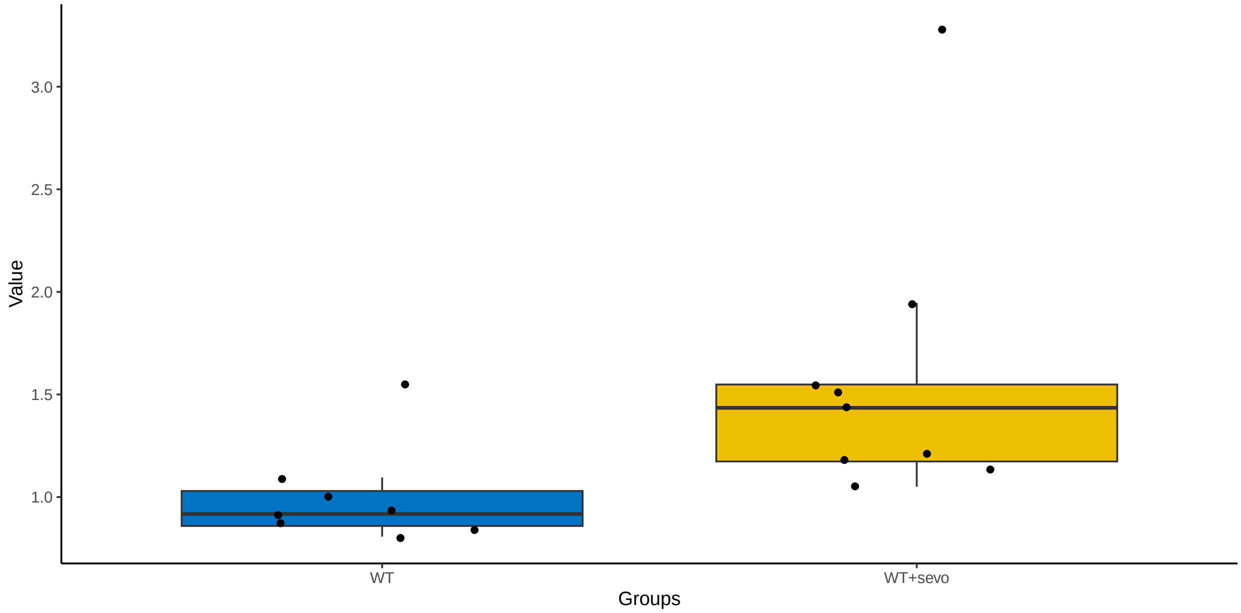


```
## 1. Normality assumption test by Shapiro_Wilk test is
##  p = 0.000
##  Normality assumption was rejected
## 2. The result of Kruskall_Wallis test:
##  p = 0.006
##  A statistically significant difference exist between groups
##
```

# Data analysis using R

```
## Present data is ** Fig 2E_HSP10_WT.csv **
##
## **  Data structure  **
## 'data.frame':    9 obs. of  3 variables:
##  $ subject: int  1 2 3 4 5 6 7 8 9
##  $ group  : chr  "WT" "WT" "WT" "WT" ...
##  $ HSP10  : num  0.807 0.624 1.249 1.32 1.506 ...
##
## ** Explorative data analysis with graphics**
```

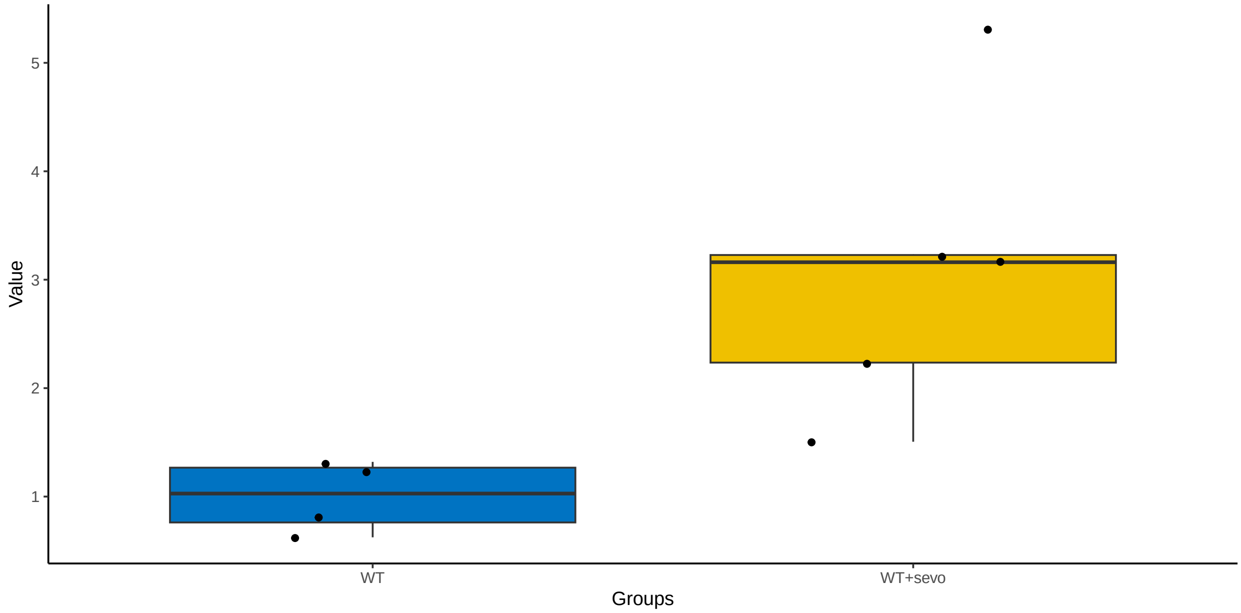


```
## 1. Normality assumption test by Shapiro_Wilk test is
##  p = 0.276
##  Normality assumption was not rejected
## 2. Equal variance test by Bartlett test is
##  p = 0.037
##  Equal variance assumption was rejected
## 3. The result of Welch ANOVA is
##  p = 0.028
##  A statistically significant difference exist between groups
##
##   Tukey multiple comparisons of means
##     95% family-wise confidence level
##
## Fit: aov(formula = d1[, 3] ~ d1[, 2])
##
```

```
## $`d1[, 2]`
##                 diff       lwr      upr    p adj
## WT+sevo-WT 2.081961 0.3441252 3.819796 0.025302
```

# Data analysis using R

```
## Present data is ** Fig 2E_HSP60_WT.csv **
##
## **  Data structure  **
## 'data.frame':    9 obs. of  3 variables:
##  $ subject: int  1 2 3 4 5 6 7 8 9
##  $ group  : chr  "WT" "WT" "WT" "WT" ...
##  $ HSP60  : num  0.787 0.541 1.542 1.129 1.608 ...
##
## ** Explorative data analysis with graphics**
```

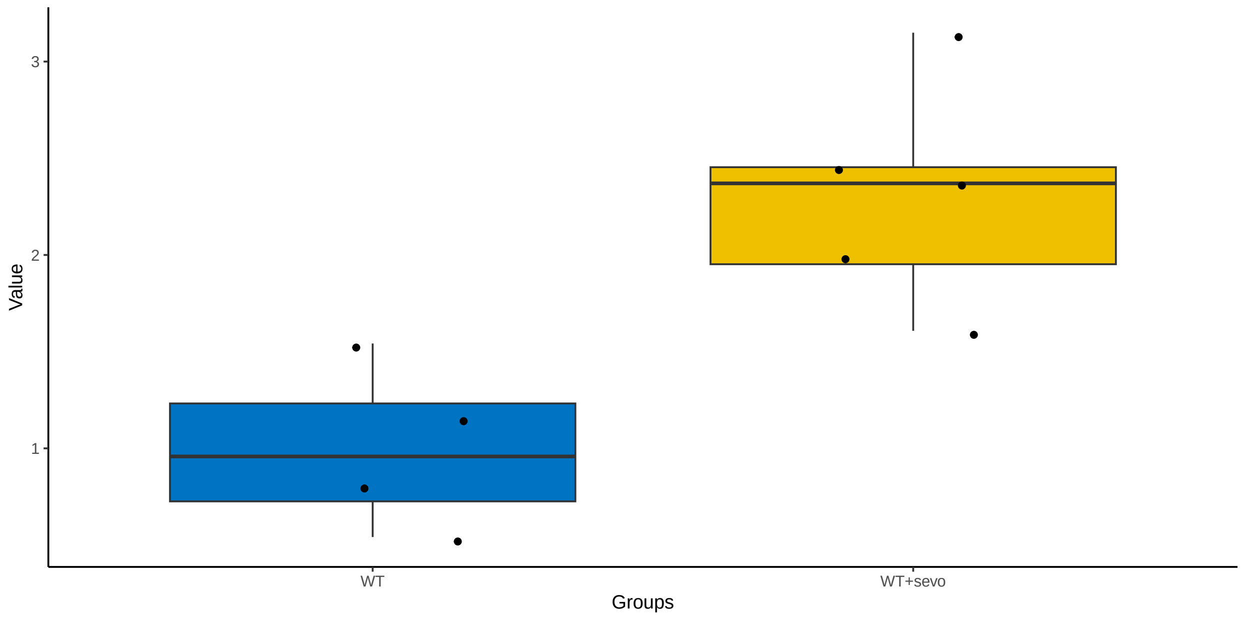


```
## 1. Normality assumption test by Shapiro_Wilk test is
##  p = 0.902
##  Normality assumption was not rejected
## 2. Equal variance test by Bartlett test is
##  p = 0.622
##  Equal variance assumption was not rejected
## 3. The result of anova is
##  p = 0.007
##  A statistically significant difference exist between groups
##
##   Tukey multiple comparisons of means
##     95% family-wise confidence level
##
## Fit: aov(formula = d1[, 3] ~ d1[, 2], data = d1)
##
```

```
## $`d1[, 2]`
##                 diff       lwr      upr     p adj
## WT+sevo-WT 1.306608 0.4767221 2.136493 0.0074271
```

# Data analysis using R

```
## Present data is ** Fig 2E_LONP1_WT.csv **
##
## **  Data structure  **
## 'data.frame':    9 obs. of  3 variables:
##  $ subject: int  1 2 3 4 5 6 7 8 9
##  $ group  : chr  "WT" "WT" "WT" "WT" ...
##  $ LONP1  : num  1.2 0.613 0.961 1.225 1.551 ...
##
## ** Explorative data analysis with graphics**
```

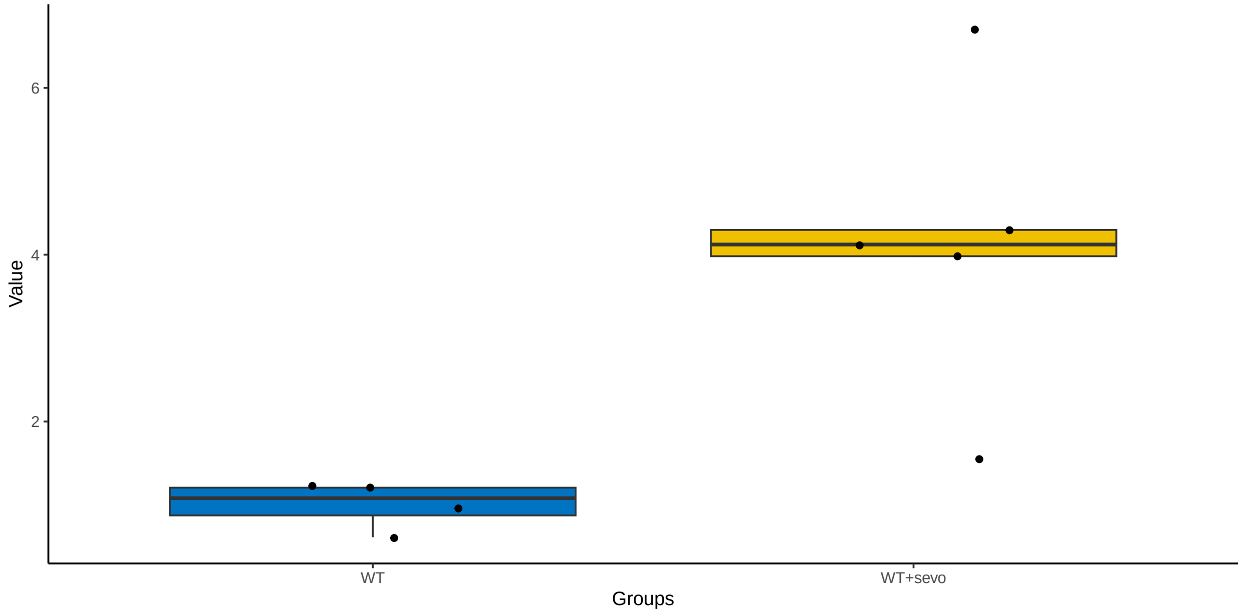


```
## 1. Normality assumption test by Shapiro_Wilk test is
##  p = 0.029
##  Normality assumption was rejected
## 2. The result of Kruskall_Wallis test:
##  p = 0.014
##  A statistically significant difference exist between groups
##
```

# Data analysis using R

```
## Present data is ** Fig 2E_mtDNAj_WT.csv **
##
## **  Data structure  **
## 'data.frame':    9 obs. of  3 variables:
##  $ subject: int  1 2 3 4 5 6 7 8 9
##  $ group  : chr  "WT" "WT" "WT" "WT" ...
##  $ mtDNAj : num  1.133 0.325 1.24 1.302 1.181 ...
##
## ** Explorative data analysis with graphics**
```

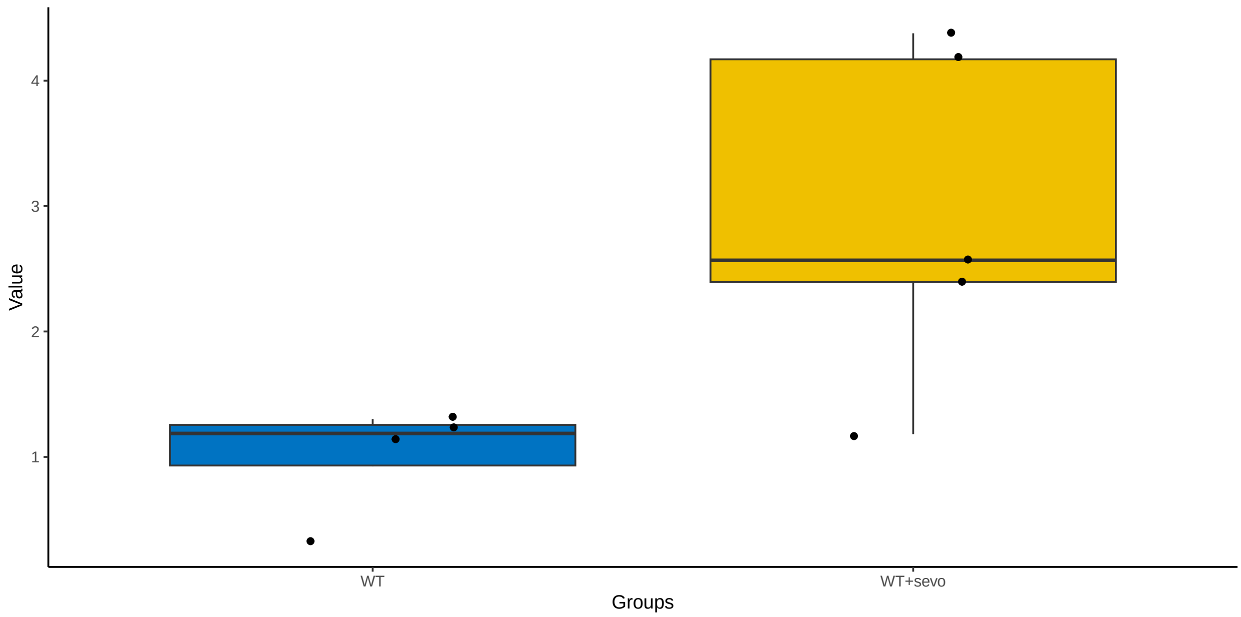


```
## 1. Normality assumption test by Shapiro_Wilk test is
##  p = 0.807
##  Normality assumption was not rejected
## 2. Equal variance test by Bartlett test is
##  p = 0.099
##  Equal variance assumption was not rejected
## 3. The result of anova is
##  p = 0.029
##  A statistically significant difference exist between groups
##
##   Tukey multiple comparisons of means
##     95% family-wise confidence level
##
## Fit: aov(formula = d1[, 3] ~ d1[, 2], data = d1)
##
```

```
## $`d1[, 2]`
##                 diff      lwr      upr     p adj
## WT+sevo-WT 1.938505 0.271353 3.605657 0.0285245
```

# Data analysis using R

```
## Present data is ** Fig 2F_ATF5_ATF5 cKO.csv **
##
## **  Data structure  **
## 'data.frame':    10 obs. of  3 variables:
##  $ subject: int  1 2 3 4 5 6 7 8 9 10
##  $ group  : chr  "ATF5 cKO+con" "ATF5 cKO+con" "ATF5 cKO+con" "ATF5 cKO+con" ...
##  $ ATF5   : num  1.375 1.292 0.717 0.858 0.758 ...
##
## ** Explorative data analysis with graphics**
```

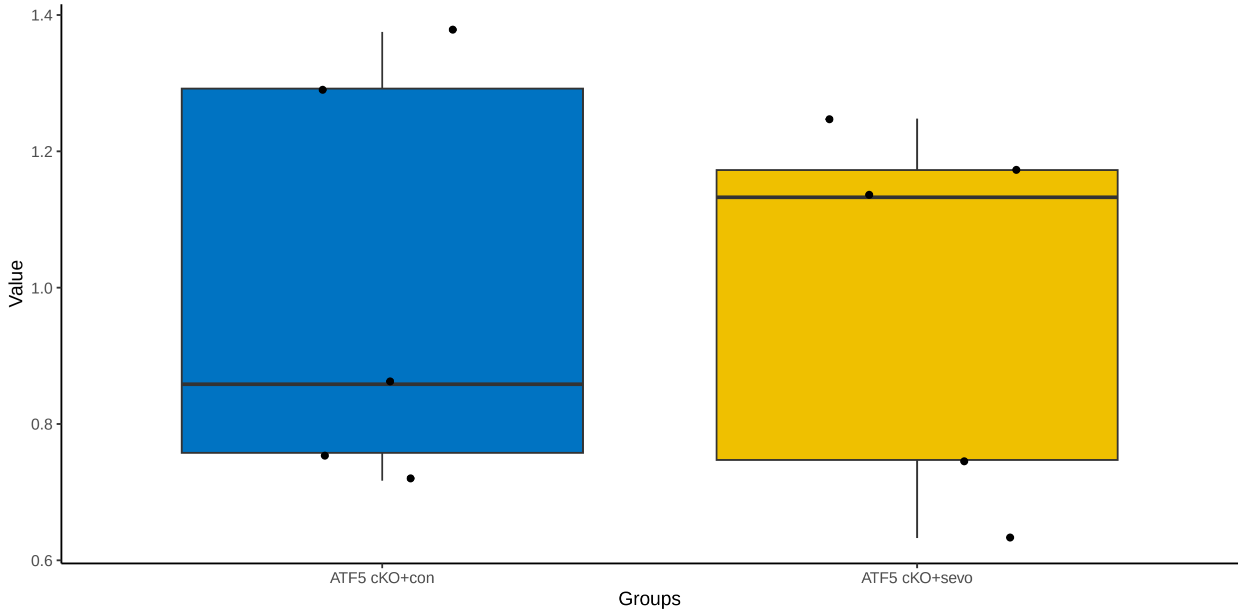


```
## 1. Normality assumption test by Shapiro_Wilk test is
##  p = 0.123
##  Normality assumption was not rejected
## 2. Equal variance test by Bartlett test is
##  p = 0.830
##  Equal variance assumption was not rejected
## 3. The result of anova is
##  p = 0.944
##  A statistically significant difference do not exist between groups
##
```

# Data analysis using R

```
## Present data is ** Fig 2F_CLPP_ATF5 cKO.csv **
##
## **  Data structure  **
## 'data.frame':    10 obs. of  3 variables:
##  $ subject: int  1 2 3 4 5 6 7 8 9 10
##  $ group  : chr  "ATF5 cKO+con" "ATF5 cKO+con" "ATF5 cKO+con" "ATF5 cKO+con" ...
##  $ CLPP   : num  1.302 0.732 0.824 1.464 0.678 ...
##
## ** Explorative data analysis with graphics**
```

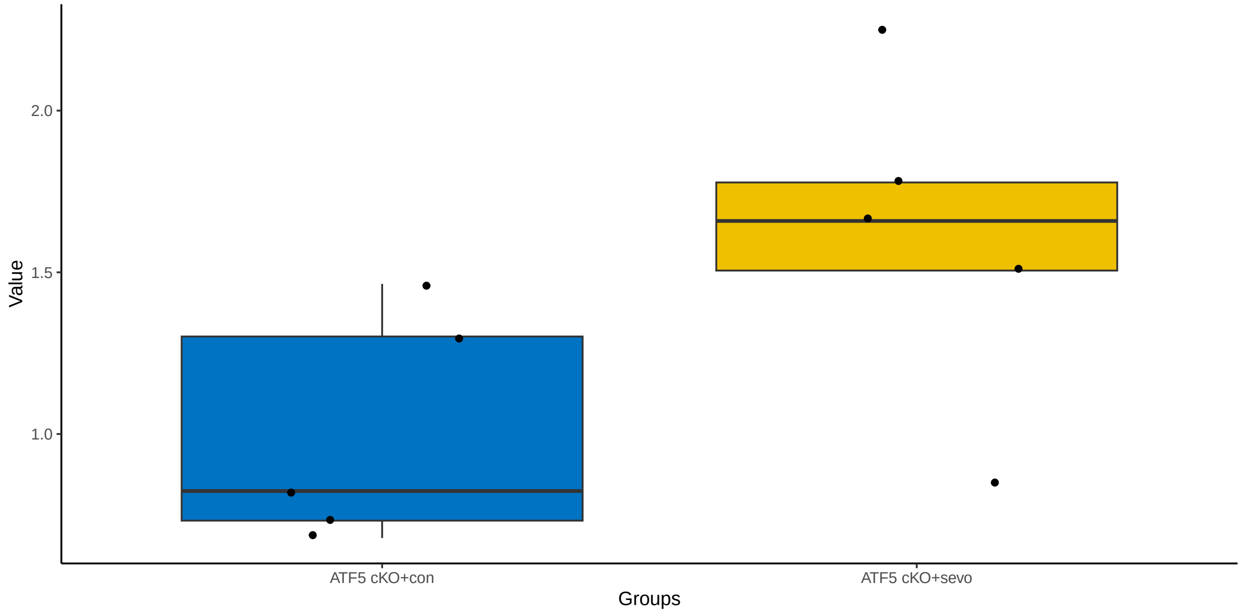


```
## 1. Normality assumption test by Shapiro_Wilk test is
##  p = 0.988
##  Normality assumption was not rejected
## 2. Equal variance test by Bartlett test is
##  p = 0.523
##  Equal variance assumption was not rejected
## 3. The result of anova is
##  p = 0.058
##  A statistically significant difference do not exist between groups
##
```

# Data analysis using R

```
## Present data is ** Fig 2F_GDF15_ATF5 cKO.csv **
##
## **  Data structure  **
## 'data.frame':    10 obs. of  3 variables:
##  $ subject: int  1 2 3 4 5 6 7 8 9 10
##  $ group  : chr  "ATF5 cKO+con" "ATF5 cKO+con" "ATF5 cKO+con" "ATF5 cKO+con" ...
##  $ GDF15  : num  1.155 0.752 0.828 1.633 0.632 ...
##
## ** Explorative data analysis with graphics**
```

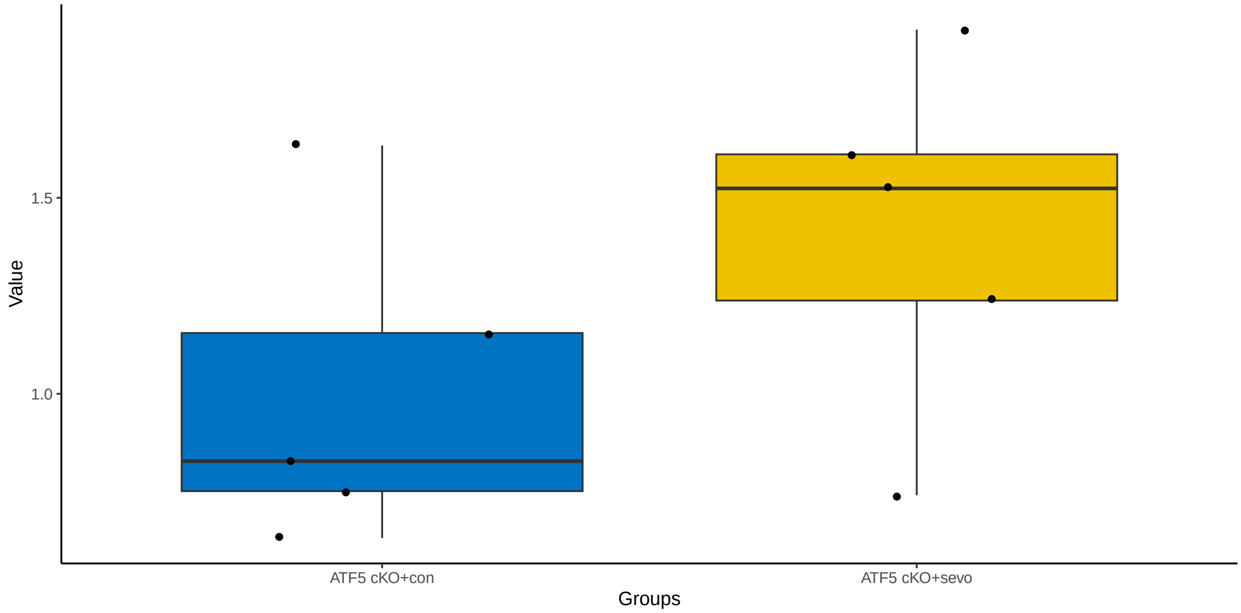


```
## 1. Normality assumption test by Shapiro_Wilk test is
##  p = 0.894
##  Normality assumption was not rejected
## 2. Equal variance test by Bartlett test is
##  p = 0.847
##  Equal variance assumption was not rejected
## 3. The result of anova is
##  p = 0.168
##  A statistically significant difference do not exist between groups
##
```

# Data analysis using R

```
## Present data is ** Fig 2F_HSP10_ATF5 cKO.csv **
##
## **  Data structure  **
## 'data.frame':    10 obs. of  3 variables:
##  $ subject: int  1 2 3 4 5 6 7 8 9 10
##  $ group  : chr  "ATF5 cKO+con" "ATF5 cKO+con" "ATF5 cKO+con" "ATF5 cKO+con" ...
##  $ HSP10  : num  1.778 0.633 0.853 1.301 0.435 ...
##
## ** Explorative data analysis with graphics**
```

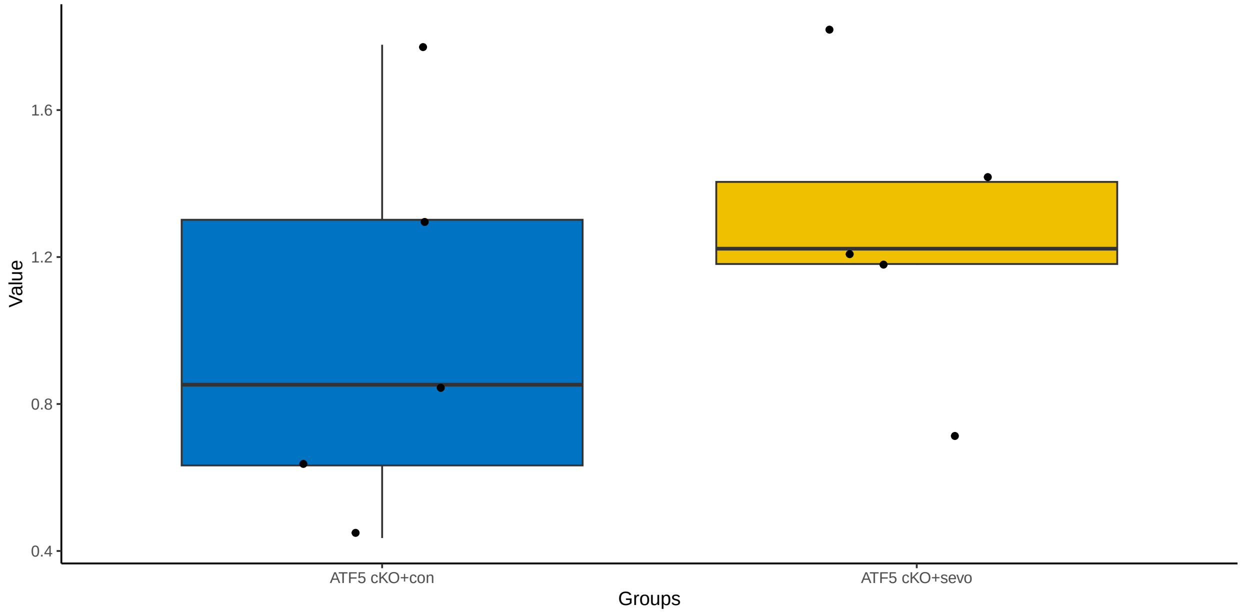


```
## 1. Normality assumption test by Shapiro_Wilk test is
##  p = 0.724
##  Normality assumption was not rejected
## 2. Equal variance test by Bartlett test is
##  p = 0.552
##  Equal variance assumption was not rejected
## 3. The result of anova is
##  p = 0.393
##  A statistically significant difference do not exist between groups
##
```

# Data analysis using R

```
## Present data is ** Fig 2F_HSP60_ATF5 cKO.csv **
##
## **  Data structure  **
## 'data.frame':    10 obs. of  3 variables:
##  $ subject: int  1 2 3 4 5 6 7 8 9 10
##  $ group  : chr  "ATF5 cKO+con" "ATF5 cKO+con" "ATF5 cKO+con" "ATF5 cKO+con" ...
##  $ HSP60  : num  1.347 0.758 0.768 1.516 0.611 ...
##
## ** Explorative data analysis with graphics**
```

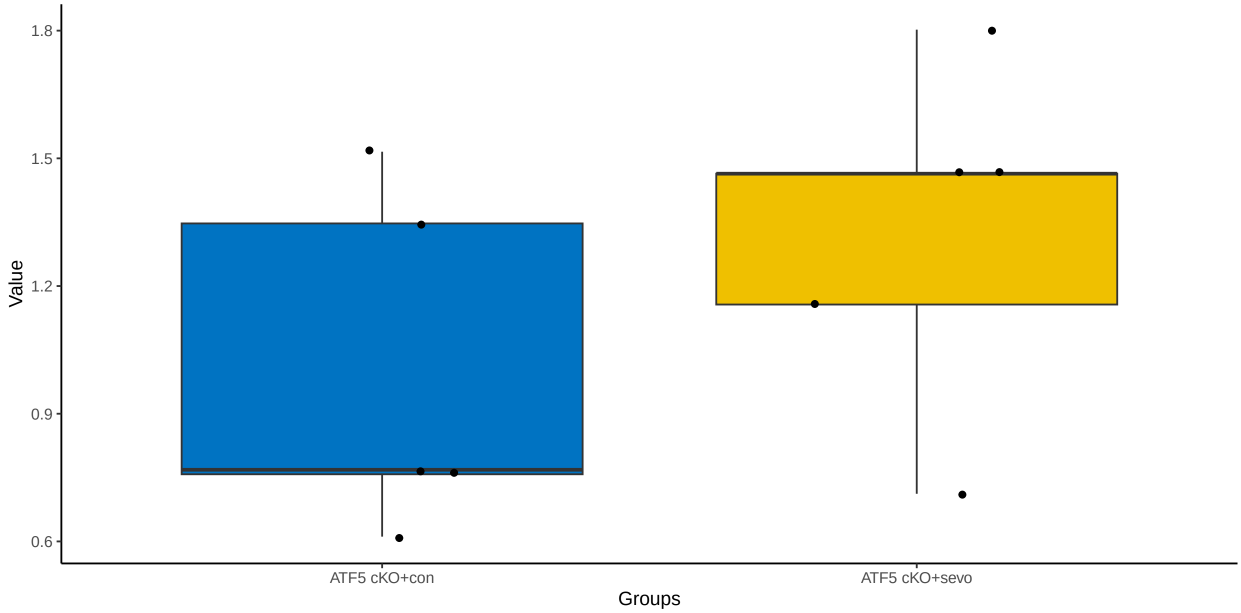


```
## 1. Normality assumption test by Shapiro_Wilk test is
##  p = 0.589
##  Normality assumption was not rejected
## 2. Equal variance test by Bartlett test is
##  p = 0.976
##  Equal variance assumption was not rejected
## 3. The result of anova is
##  p = 0.249
##  A statistically significant difference do not exist between groups
##
```

# Data analysis using R

```
## Present data is ** Fig 2F_LONP1_ATF5 cKO.csv **
##
## **  Data structure  **
## 'data.frame':    10 obs. of  3 variables:
##  $ subject: int  1 2 3 4 5 6 7 8 9 10
##  $ group  : chr  "ATF5 cKO+con" "ATF5 cKO+con" "ATF5 cKO+con" "ATF5 cKO+con" ...
##  $ LONP1  : num  1.172 0.702 1.013 1.494 0.619 ...
##
## ** Explorative data analysis with graphics**
```

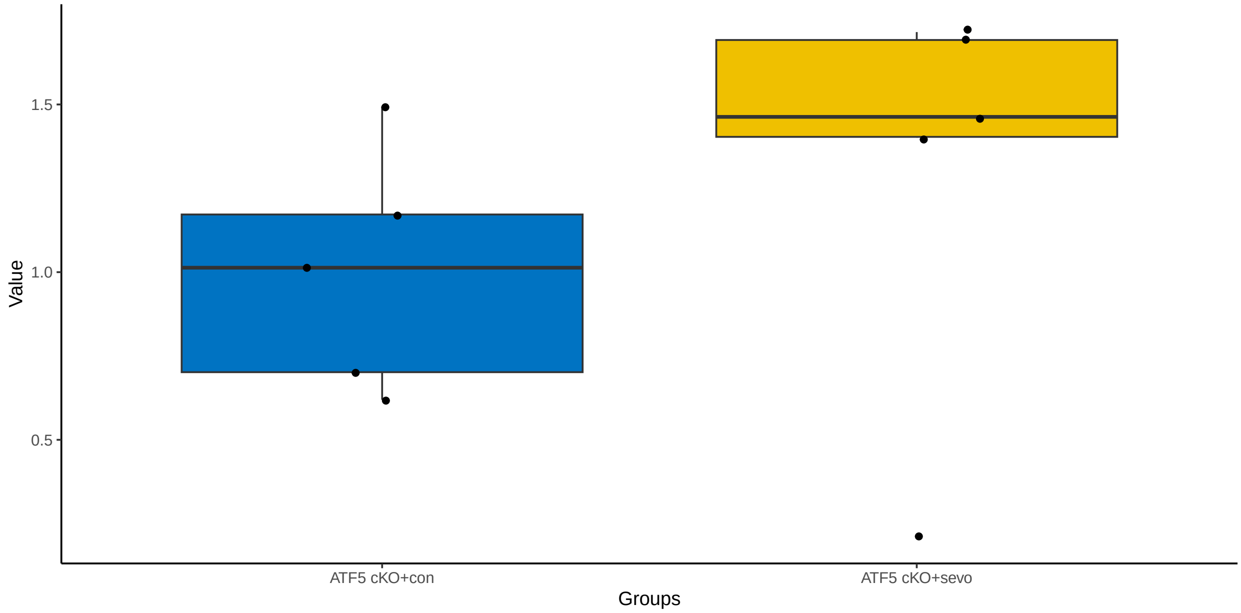


```
## 1. Normality assumption test by Shapiro_Wilk test is
##  p = 0.105
##  Normality assumption was not rejected
## 2. Equal variance test by Bartlett test is
##  p = 0.302
##  Equal variance assumption was not rejected
## 3. The result of anova is
##  p = 0.383
##  A statistically significant difference do not exist between groups
##
```

# Data analysis using R

```
## Present data is ** Fig 2F_mtDNAj_ATF5 cKO.csv **
##
## **  Data structure  **
## 'data.frame':    10 obs. of  3 variables:
##  $ subject: int  1 2 3 4 5 6 7 8 9 10
##  $ group  : chr  "ATF5 cKO+con" "ATF5 cKO+con" "ATF5 cKO+con" "ATF5 cKO+con" ...
##  $ mtDNAj : num  1.236 0.677 0.793 1.598 0.696 ...
##
## ** Explorative data analysis with graphics**
```

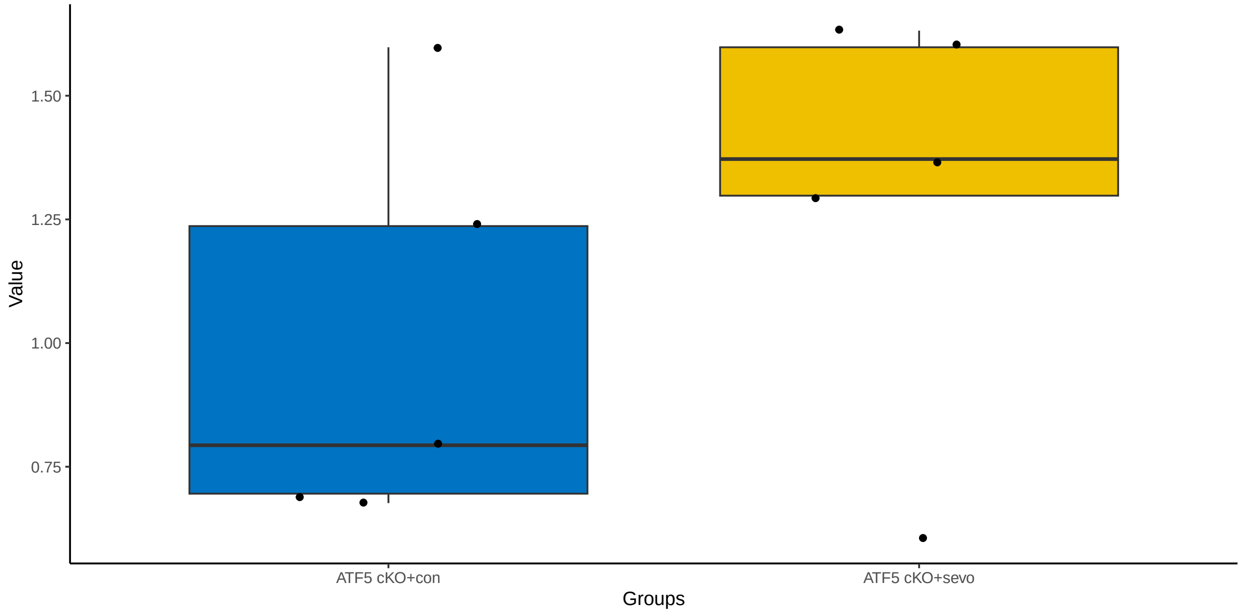


```
## 1. Normality assumption test by Shapiro_Wilk test is
##  p = 0.942
##  Normality assumption was not rejected
## 2. Equal variance test by Bartlett test is
##  p = 0.970
##  Equal variance assumption was not rejected
## 3. The result of anova is
##  p = 0.276
##  A statistically significant difference do not exist between groups
##
```

# Data analysis using R

```
## Present data is ** Figure2H_ATF5 cKO_ATF5.csv **
##
## **  Data structure  **
## 'data.frame':    14 obs. of  3 variables:
##  $ subject: int  1 2 3 4 5 6 7 8 9 10 ...
##  $ group  : chr  "WT" "WT" "WT" "WT" ...
##  $ ATF5   : num  1.093 0.945 1.049 0.913 0.866 ...
##
## ** Explorative data analysis with graphics**
```

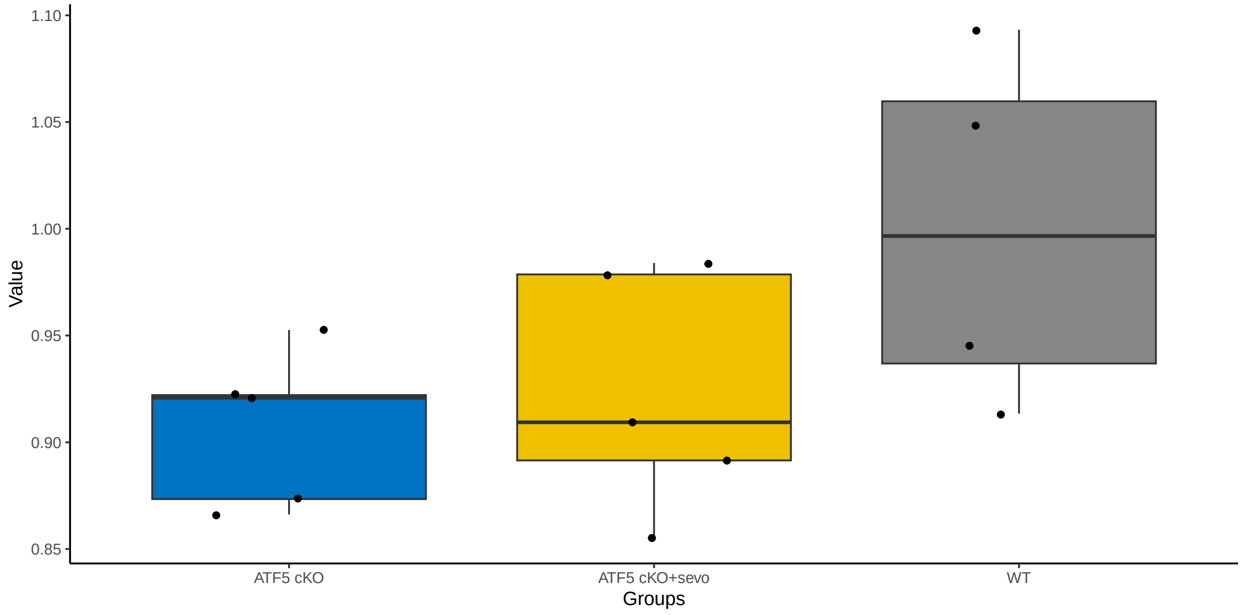


```
## 1. Normality assumption test by Shapiro_Wilk test is
##  p = 0.658
##  Normality assumption was not rejected
## 2. Equal variance test by Bartlett test is
##  p = 0.339
##  Equal variance assumption was not rejected
## 3. The result of anova is
##  p = 0.094
##  A statistically significant difference do not exist between groups
##
```

# Data analysis using R

```
## Present data is ** Figure2H_ATF5 cKO_CLPP.csv **
##
## **  Data structure  **
## 'data.frame':    14 obs. of  3 variables:
##  $ subject: int  1 2 3 4 5 6 7 8 9 10 ...
##  $ group  : chr  "WT" "WT" "WT" "WT" ...
##  $ CLPP   : num  0.933 0.981 0.965 1.121 0.978 ...
##
## ** Explorative data analysis with graphics**
```

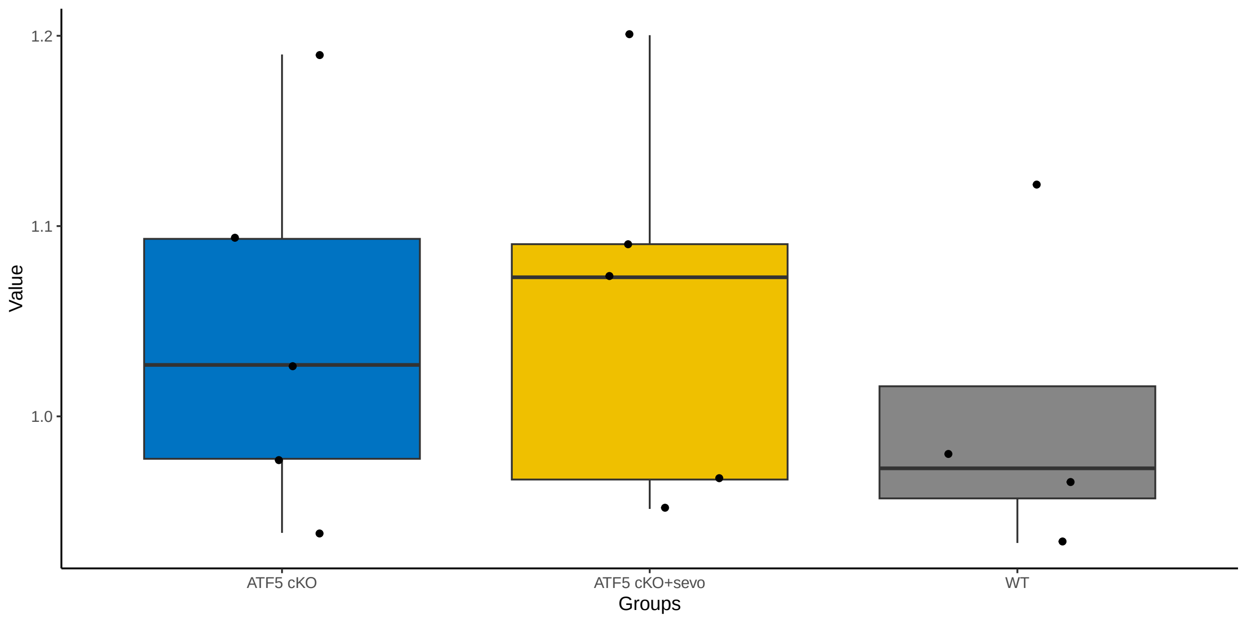


```
## 1. Normality assumption test by Shapiro_Wilk test is
##  p = 0.147
##  Normality assumption was not rejected
## 2. Equal variance test by Bartlett test is
##  p = 0.936
##  Equal variance assumption was not rejected
## 3. The result of anova is
##  p = 0.668
##  A statistically significant difference do not exist between groups
##
```

# Data analysis using R

```
## Present data is ** Figure2H_ATF5 cKO_HSP60.csv **
##
## **  Data structure  **
## 'data.frame':    14 obs. of  3 variables:
##  $ subject: int  1 2 3 4 5 6 7 8 9 10 ...
##  $ group  : chr  "WT" "WT" "WT" "WT" ...
##  $ HSP60  : num  1.017 1.061 0.93 0.992 0.85 ...
##
## ** Explorative data analysis with graphics**
```

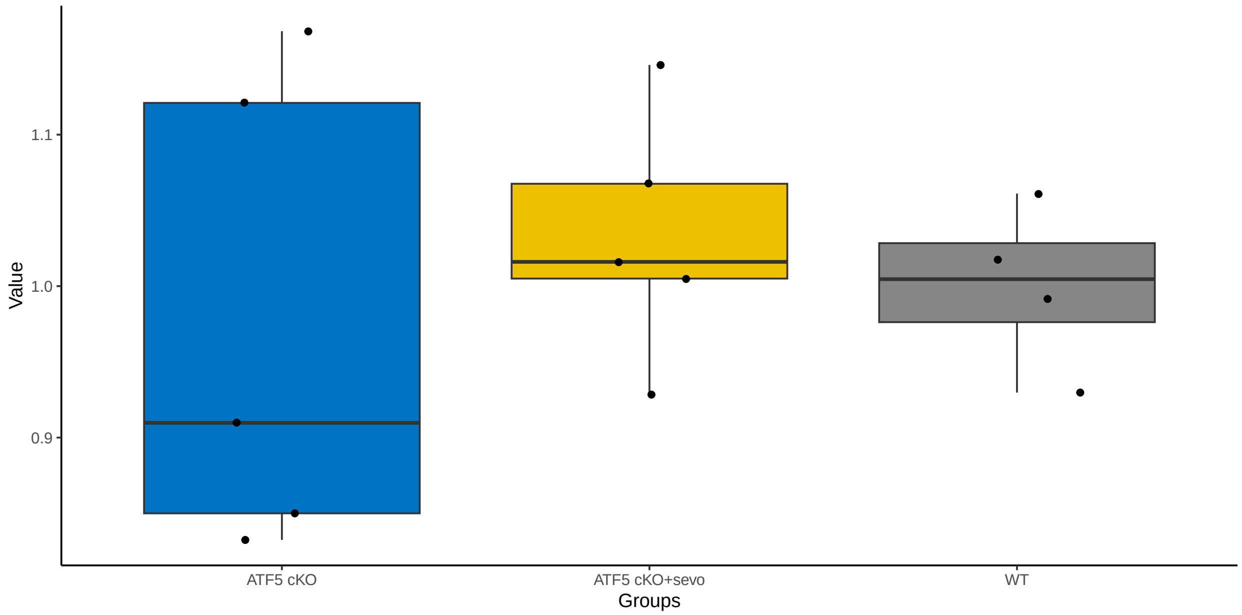


```
## 1. Normality assumption test by Shapiro_Wilk test is
##  p = 0.809
##  Normality assumption was not rejected
## 2. Equal variance test by Bartlett test is
##  p = 0.179
##  Equal variance assumption was not rejected
## 3. The result of anova is
##  p = 0.727
##  A statistically significant difference do not exist between groups
##
```

# Data analysis using R

```
## Present data is ** Figure2H_ATF5 cKO_HSP70.csv **
##
## **  Data structure  **
## 'data.frame':    14 obs. of  3 variables:
##  $ subject: int  1 2 3 4 5 6 7 8 9 10 ...
##  $ group  : chr  "WT" "WT" "WT" "WT" ...
##  $ HSP70  : num  0.923 1.004 0.971 1.102 1.384 ...
##
## ** Explorative data analysis with graphics**
```

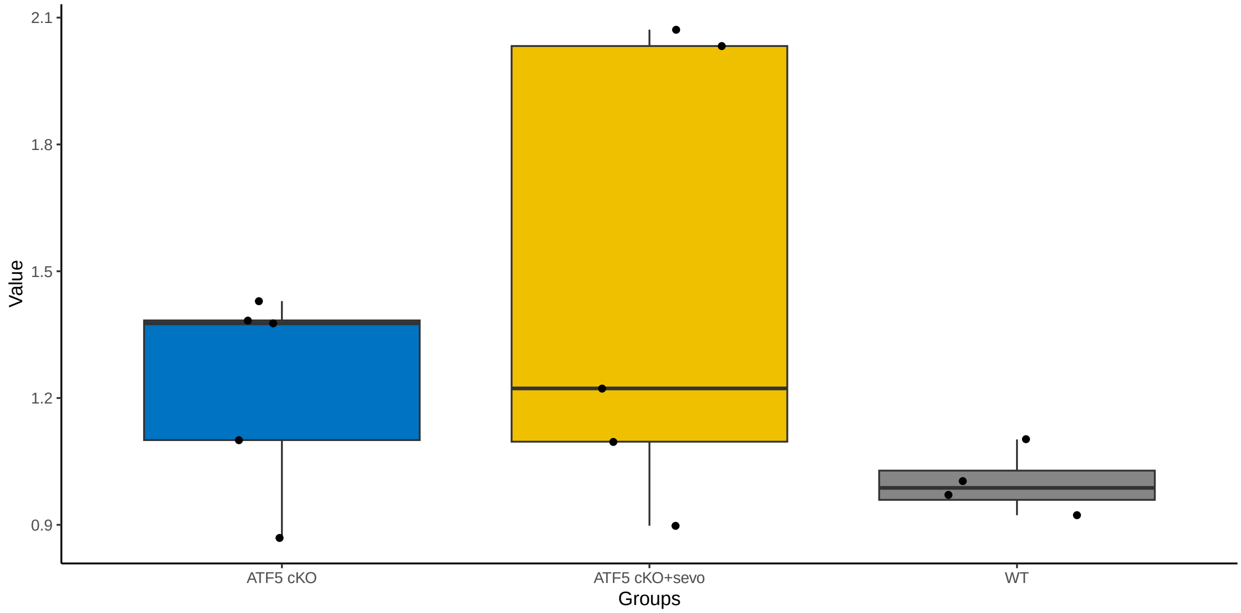


```
## 1. Normality assumption test by Shapiro_Wilk test is
##  p = 0.767
##  Normality assumption was not rejected
## 2. Equal variance test by Bartlett test is
##  p = 0.015
##  Equal variance assumption was rejected
## 3. The result of Welch ANOVA is
##  p = 0.110
##  A statistically significant difference do not exist between groups
##
```

# Data analysis using R

```
## Present data is ** Figure2H_ATF5 cKO_LONP1.csv **
##
## **  Data structure  **
## 'data.frame':    14 obs. of  3 variables:
##  $ subject: int  1 2 3 4 5 6 7 8 9 10 ...
##  $ group  : chr  "WT" "WT" "WT" "WT" ...
##  $ LONP1  : num  1.014 0.916 1.047 1.023 1.224 ...
##
## ** Explorative data analysis with graphics**
```

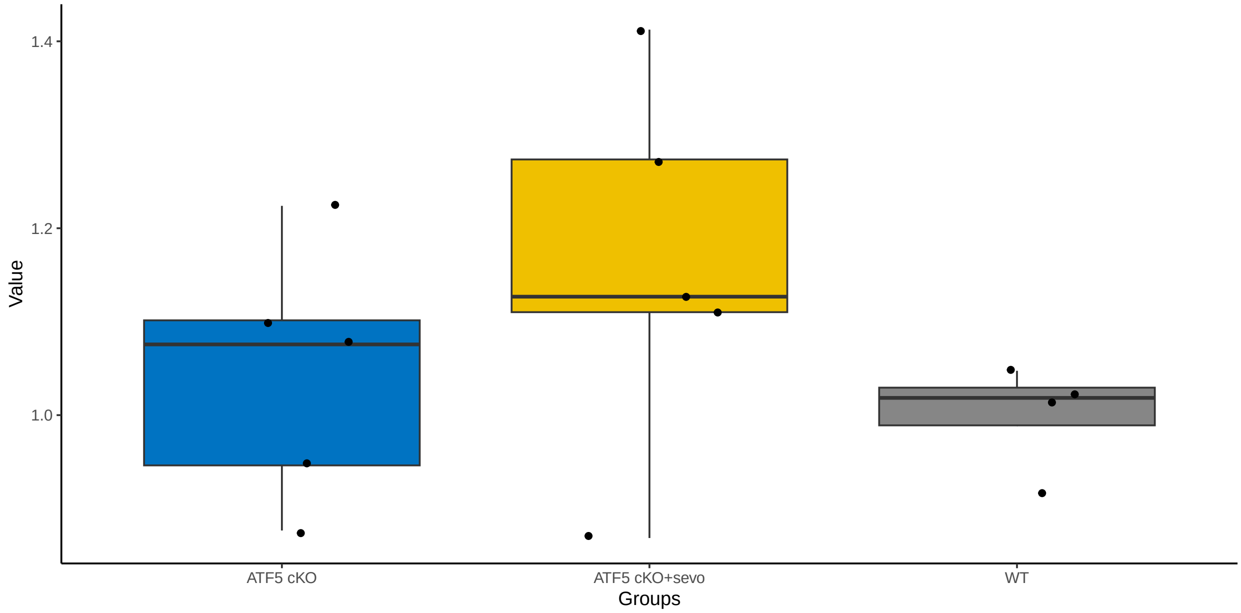


```
## 1. Normality assumption test by Shapiro_Wilk test is
##  p = 0.991
##  Normality assumption was not rejected
## 2. Equal variance test by Bartlett test is
##  p = 0.159
##  Equal variance assumption was not rejected
## 3. The result of anova is
##  p = 0.297
##  A statistically significant difference do not exist between groups
##
```

# Data analysis using R

```
## Present data is ** Fig2Ⅰ_ATF5 cKO_OCR_Basal.csv **
##
## **  Data structure  **
## 'data.frame':    20 obs. of  3 variables:
##  $ subject: int  1 2 3 4 5 6 7 8 9 10 ...
##  $ group  : chr  "WT" "WT" "WT" "WT" ...
##  $ basal  : num  53.5 54.2 64.7 74.7 60.5 ...
##
## ** Explorative data analysis with graphics**
```

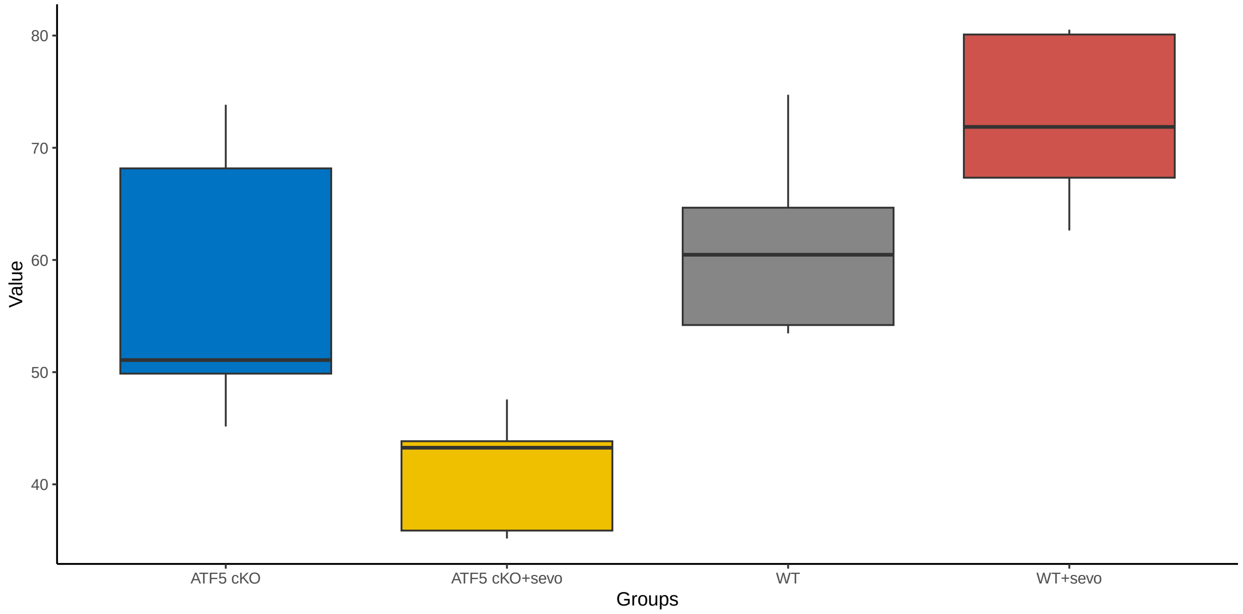


```
## 1. Normality assumption test by Shapiro_Wilk test is
##  p = 0.338
##  Normality assumption was not rejected
## 2. Equal variance test by Bartlett test is
##  p = 0.468
##  Equal variance assumption was not rejected
## 3. The result of anova is
##  p = 0.000
##  A statistically significant difference exist between groups
##
##   Tukey multiple comparisons of means
##     95% family-wise confidence level
##
## Fit: aov(formula = d1[, 3] ~ d1[, 2], data = d1)
##
```

```
## $`d1[, 2]`
##                                diff        lwr        upr     p adj
## ATF5 cKO+sevo-ATF5 cKO -16.475027 -32.781057 -0.1689972 0.0472320
## WT-ATF5 cKO               3.880908 -12.425122 20.1869376 0.9028224
## WT+sevo-ATF5 cKO         14.867512  -1.438518 31.1735419 0.0804123
## WT-ATF5 cKO+sevo         20.355935   4.049905 36.6619647 0.0122370
## WT+sevo-ATF5 cKO+sevo    31.342539  15.036509 47.6485690 0.0002566
## WT+sevo-WT               10.986604  -5.319426 27.2926341 0.2558855
```

# Data analysis using R

```
## Present data is ** Fig2 I_ATF5 cKO_OCR_state3.csv **
##
## **  Data structure  **
## 'data.frame':    20 obs. of  3 variables:
##  $ subject: int  1 2 3 4 5 6 7 8 9 10 ...
##  $ group  : chr  "WT" "WT" "WT" "WT" ...
##  $ state3 : num  151 152 172 184 158 ...
##
## ** Explorative data analysis with graphics**
```

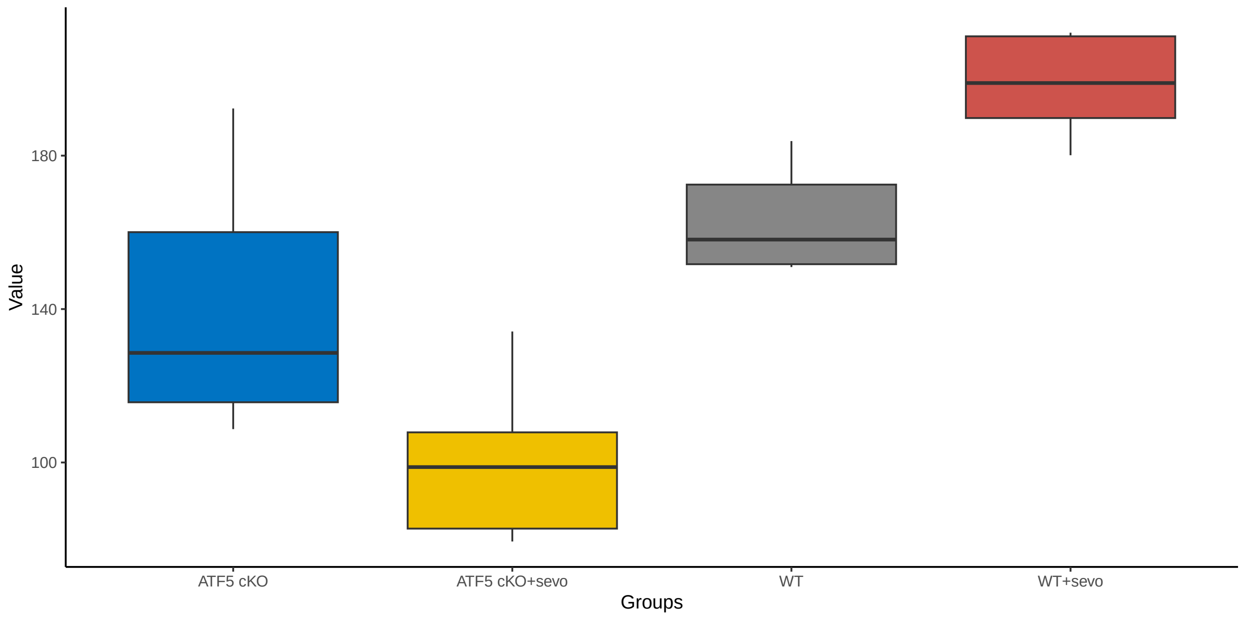


```
## 1. Normality assumption test by Shapiro_Wilk test is
##  p = 0.548
##  Normality assumption was not rejected
## 2. Equal variance test by Bartlett test is
##  p = 0.232
##  Equal variance assumption was not rejected
## 3. The result of anova is
##  p = 0.000
##  A statistically significant difference exist between groups
##
##   Tukey multiple comparisons of means
##     95% family-wise confidence level
##
## Fit: aov(formula = d1[, 3] ~ d1[, 2], data = d1)
##
```

```
## $`d1[, 2]`
##                              diff        lwr         upr     p adj
## ATF5 cKO+sevo-ATF5 cKO -40.46933 -81.819365   0.8807142 0.0561696
## WT-ATF5 cKO             22.33671 -19.013334  63.6867454 0.4352908
## WT+sevo-ATF5 cKO        57.33180  15.981757  98.6818365 0.0054813
## WT-ATF5 cKO+sevo        62.80603  21.455992 104.1560709 0.0025369
## WT+sevo-ATF5 cKO+sevo   97.80112  56.451083 139.1511620 0.0000245
## WT+sevo-WT              34.99509  -6.354949  76.3451308 0.1128836
```

# Data analysis using R

```
## Present data is ** Fig2 l_ATF5 cKO_OCR_state3u.csv **
##
## **  Data structure  **
## 'data.frame':    20 obs. of  3 variables:
##  $ subject: int  1 2 3 4 5 6 7 8 9 10 ...
##  $ group  : chr  "WT" "WT" "WT" "WT" ...
##  $ state3u: num  115 108 132 115 107 ...
##
## ** Explorative data analysis with graphics**
```

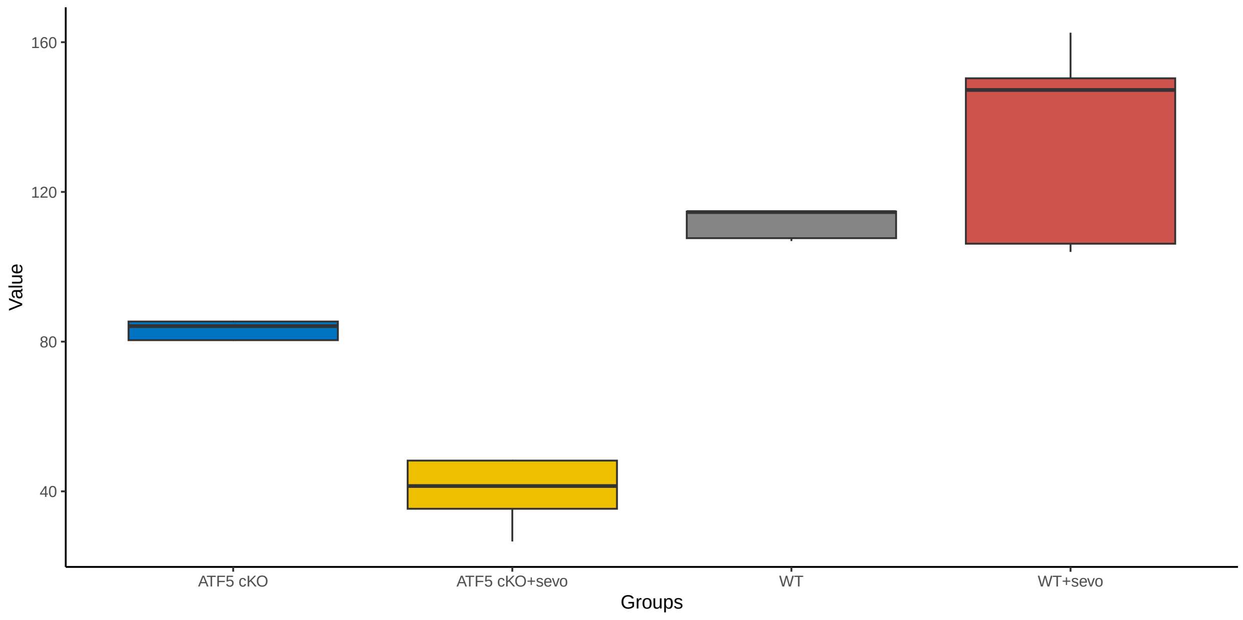


```
## 1. Normality assumption test by Shapiro_Wilk test is
##  p = 0.379
##  Normality assumption was not rejected
## 2. Equal variance test by Bartlett test is
##  p = 0.324
##  Equal variance assumption was not rejected
## 3. The result of anova is
##  p = 0.000
##  A statistically significant difference exist between groups
##
##   Tukey multiple comparisons of means
##     95% family-wise confidence level
##
## Fit: aov(formula = d1[, 3] ~ d1[, 2], data = d1)
##
```

```
## $`d1[, 2]`
##                                diff        lwr        upr     p adj
## ATF5 cKO+sevo-ATF5 cKO -39.41085 -74.449704  -4.371991 0.0248838
## WT-ATF5 cKO              31.92195  -3.116909  66.960804 0.0807250
## WT+sevo-ATF5 cKO         50.81388  15.775022  85.852735 0.0037817
## WT-ATF5 cKO+sevo         71.33280  36.293939 106.371651 0.0001378
## WT+sevo-ATF5 cKO+sevo    90.22473  55.185870 125.263582 0.0000087
## WT+sevo-WT               18.89193 -16.146926  53.930787 0.4368671
```

# Data analysis using R

```
## Present data is ** Fig2 I_ATF5 cKO_OCR_state4o.csv **
##
## **  Data structure  **
## 'data.frame':    20 obs. of  3 variables:
##  $ subject: int  1 2 3 4 5 6 7 8 9 10 ...
##  $ group  : chr  "WT" "WT" "WT" "WT" ...
##  $ state4o: num  28.5 28.1 28.6 30.2 30.2 ...
##
## ** Explorative data analysis with graphics**
```

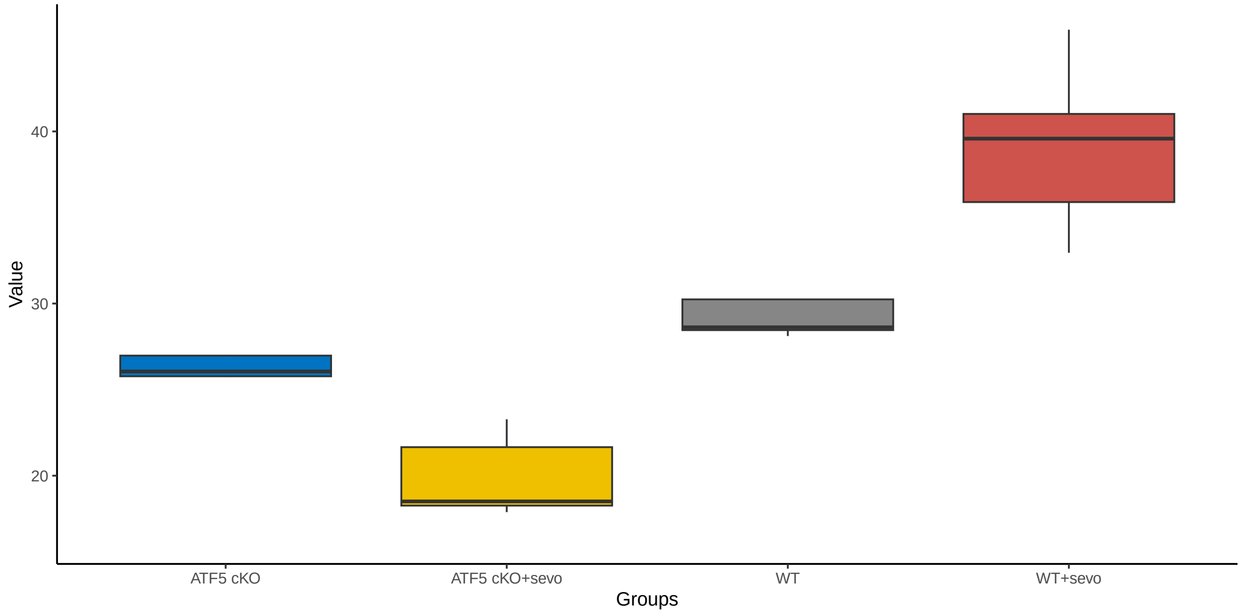


```
## 1. Normality assumption test by Shapiro_Wilk test is
##  p = 0.196
##  Normality assumption was not rejected
## 2. Equal variance test by Bartlett test is
##  p = 0.013
##  Equal variance assumption was rejected
## 3. The result of Welch ANOVA is
##  p = 0.000
##  A statistically significant difference exist between groups
##
##   Tukey multiple comparisons of means
##     95% family-wise confidence level
##
## Fit: aov(formula = d1[, 3] ~ d1[, 2])
##
```

```
## $`d1[, 2]`
##                                diff        lwr       upr     p adj
## ATF5 cKO+sevo-ATF5 cKO -6.155884 -14.050518  1.738751 0.1570546
## WT-ATF5 cKO              3.062434  -4.832200 10.957069 0.6888638
## WT+sevo-ATF5 cKO        12.997682   5.103048 20.892317 0.0012148
## WT-ATF5 cKO+sevo         9.218318   1.323684 17.112953 0.0194809
## WT+sevo-ATF5 cKO+sevo  19.153566  11.258932 27.048201 0.0000180
## WT+sevo-WT               9.935248   2.040613 17.829882 0.0115409
```

# Data analysis using R

```
## Present data is ** Fig 3B_cortex infarct volume_GDF15 KO.csv **
##
## **  Data structure  **
## 'data.frame':    18 obs. of  3 variables:
##  $ subject: int  1 2 3 4 5 6 7 8 9 10 ...
##  $ group  : chr  "WT" "WT" "WT" "WT" ...
##  $ infarct: num  0.629 0.445 0.715 0.526 0.341 ...
##
## ** Explorative data analysis with graphics**
```

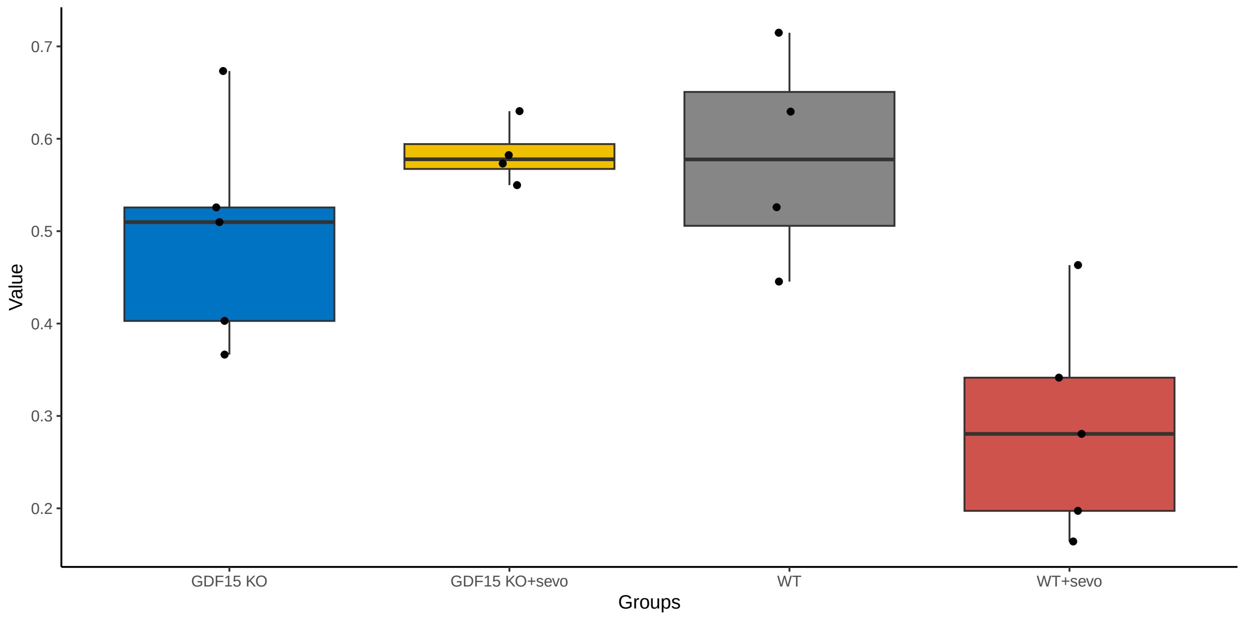


```
## 1. Normality assumption test by Shapiro_Wilk test is
##  p = 0.277
##  Normality assumption was not rejected
## 2. Equal variance test by Bartlett test is
##  p = 0.257
##  Equal variance assumption was not rejected
## 3. The result of anova is
##  p = 0.003
##  A statistically significant difference exist between groups
##
##   Tukey multiple comparisons of means
##     95% family-wise confidence level
##
## Fit: aov(formula = d1[, 3] ~ d1[, 2], data = d1)
##
```

```
## $`d1[, 2]`
##                                diff        lwr         upr     p adj
## GDF15 KO+sevo-GDF15 KO  0.0882294 -0.1203228  0.29678159 0.6192683
## WT-GDF15 KO             0.0832464 -0.1253058  0.29179859 0.6602096
## WT+sevo-GDF15 KO       -0.2062716 -0.4028965 -0.00964671 0.0384362
## WT-GDF15 KO+sevo       -0.0049830 -0.2248163  0.21485031 0.9998921
## WT+sevo-GDF15 KO+sevo  -0.2945010 -0.5030532 -0.08594881 0.0052301
## WT+sevo-WT             -0.2895180 -0.4980702 -0.08096581 0.0059652
```

# Data analysis using R

```
## Present data is ** Fig 3B_neurological score_GDF15 KO.csv **
##
## **  Data structure  **
## 'data.frame':    18 obs. of  3 variables:
##  $ subject: int  1 2 3 4 5 6 7 8 9 10 ...
##  $ group  : chr  "WT" "WT" "WT" "WT" ...
##  $ score  : int  3 3 2 3 1 2 1 1 1 3 ...
##
## ** Explorative data analysis with graphics**
```

```
## 1. Normality assumption test by Shapiro_Wilk test is
##  p = 0.007
##  Normality assumption was rejected
## 2. The result of Kruskall_Wallis test:
##  p = 0.009
##  A statistically significant difference exist between groups
##
```

```
## Dunn (1964) Kruskal-Wallis multiple comparison
```

```
##   p-values adjusted with the Benjamini-Hochberg method.
```

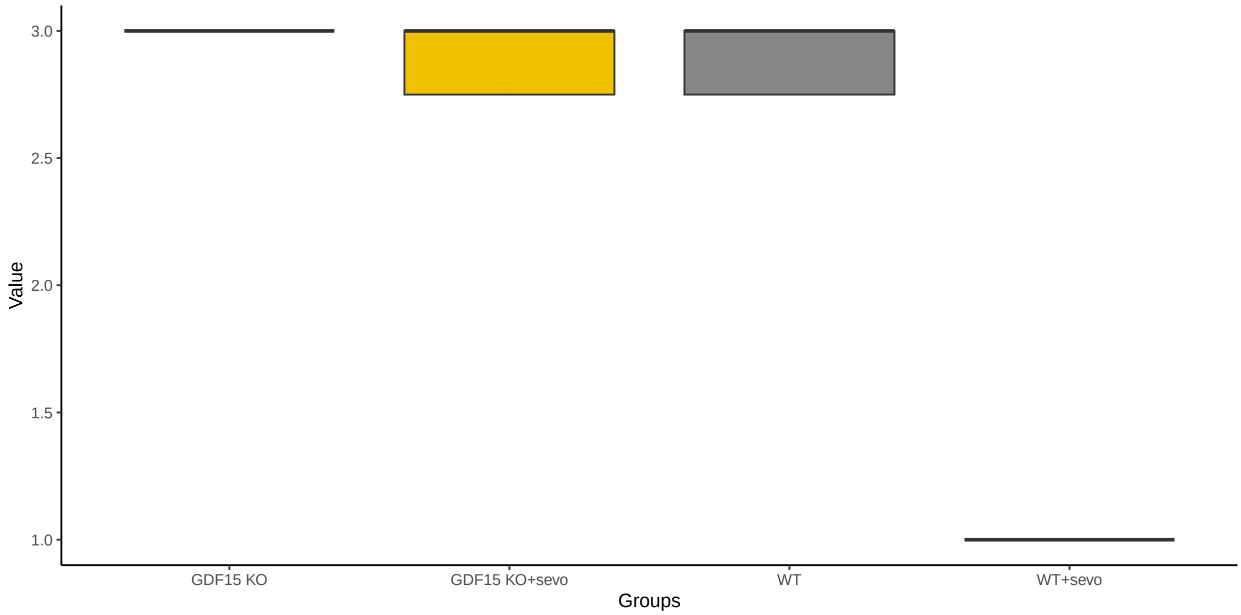


```
##                  Comparison         Z     P.unadj      P.adj
## 1 GDF15 KO - GDF15 KO+sevo 0.1086534 0.913477425 1.00000000
```

```
## 2            GDF15 KO - WT 0.1086534 0.913477425 1.00000000
## 3       GDF15 KO+sevo - WT 0.0000000 1.000000000 1.00000000
## 4       GDF15 KO - WT+sevo 2.8975711 0.003760645 0.02256387
## 5  GDF15 KO+sevo - WT+sevo 2.6232029 0.008710739 0.01742148
## 6             WT - WT+sevo 2.6232029 0.008710739 0.02613222
```

# Data analysis using R

```
## Present data is ** Fig3 E_GDF15 KO_OCR_Basal.csv **
##
## **  Data structure  **
## 'data.frame':    17 obs. of  3 variables:
##  $ subject: int  1 2 3 4 5 6 7 8 9 10 ...
##  $ group  : chr  "WT" "WT" "WT" "WT+sevo" ...
##  $ basal  : num  333 423 307 383 499 ...
##
## ** Explorative data analysis with graphics**
```

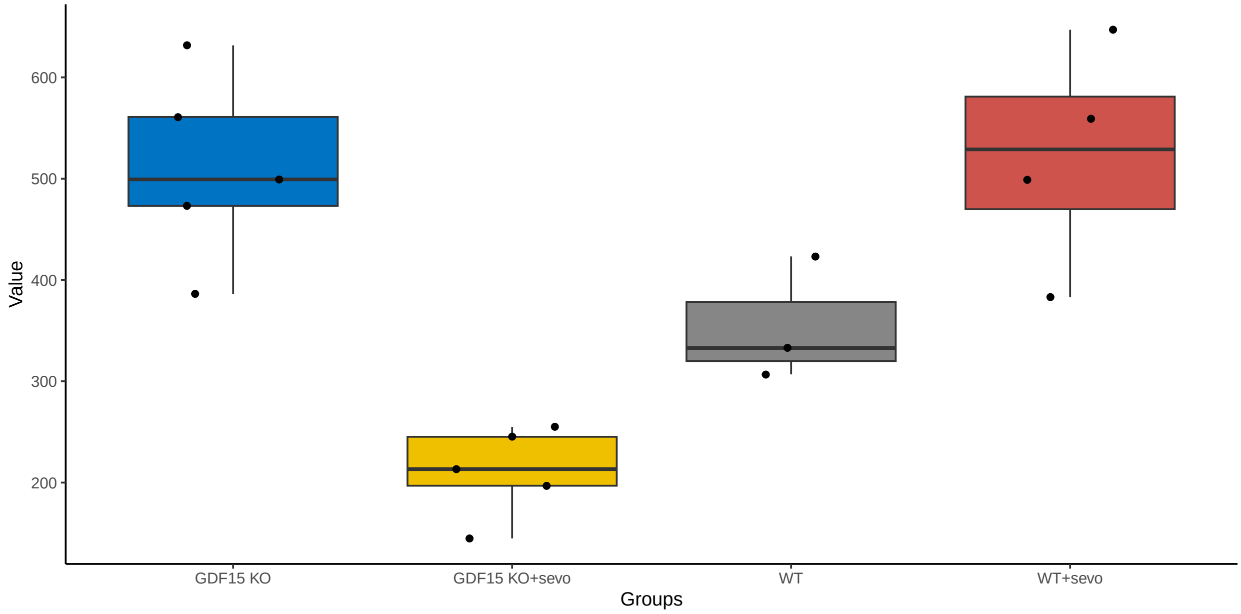


```
## 1. Normality assumption test by Shapiro_Wilk test is
##  p = 0.802
##  Normality assumption was not rejected
## 2. Equal variance test by Bartlett test is
##  p = 0.417
##  Equal variance assumption was not rejected
## 3. The result of anova is
##  p = 0.000
##  A statistically significant difference exist between groups
##
##   Tukey multiple comparisons of means
##     95% family-wise confidence level
##
## Fit: aov(formula = d1[, 3] ~ d1[, 2], data = d1)
##
```

```
## $`d1[, 2]`
##                                diff        lwr        upr     p adj
## GDF15 KO+sevo-GDF15 KO -299.15538 -450.27734 -148.03342 0.0003106
## WT-GDF15 KO            -155.88736 -330.38796   18.61325 0.0866110
## WT+sevo-GDF15 KO         11.70166 -148.58738  171.99070 0.9963451
## WT-GDF15 KO+sevo        143.26803  -31.23258  317.76863 0.1240728
## WT+sevo-GDF15 KO+sevo   310.85704  150.56800  471.14608 0.0003770
## WT+sevo-WT              167.58901  -14.90809  350.08612 0.0762909
```

# Data analysis using R

```
## Present data is ** Fig3 E_GDF15 KO_OCR_state3.csv **
##
## **  Data structure  **
## 'data.frame':    17 obs. of  3 variables:
##  $ subject: int  1 2 3 4 5 6 7 8 9 10 ...
##  $ group  : chr  "WT" "WT" "WT" "WT+sevo" ...
##  $ state3 : num  786 872 548 754 922 ...
##
## ** Explorative data analysis with graphics**
```

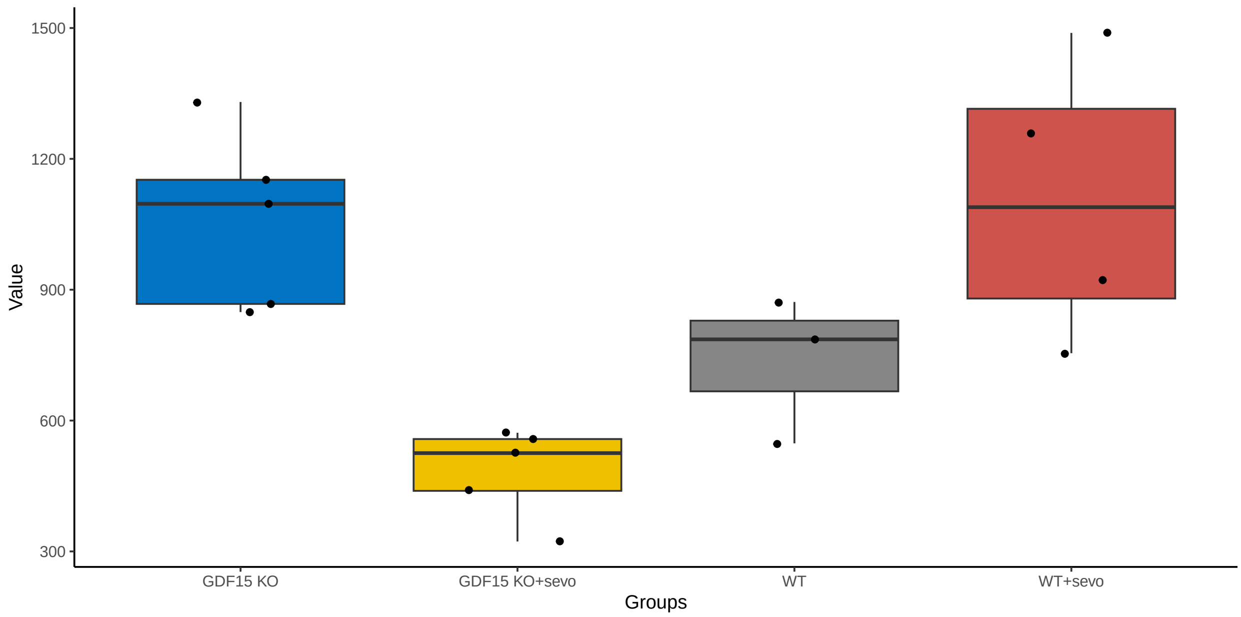


```
## 1. Normality assumption test by Shapiro_Wilk test is
##  p = 0.623
##  Normality assumption was not rejected
## 2. Equal variance test by Bartlett test is
##  p = 0.267
##  Equal variance assumption was not rejected
## 3. The result of anova is
##  p = 0.002
##  A statistically significant difference exist between groups
##
##   Tukey multiple comparisons of means
##     95% family-wise confidence level
##
## Fit: aov(formula = d1[, 3] ~ d1[, 2], data = d1)
##
```

```
## $`d1[, 2]`
##                               diff       lwr       upr     p adj
## GDF15 KO+sevo-GDF15 KO -575.8333 -971.5919 -180.0746 0.0044191
## WT-GDF15 KO            -323.8541 -780.8369  133.1286 0.2103491
## WT+sevo-GDF15 KO         46.2637 -373.5018  466.0292 0.9877559
## WT-GDF15 KO+sevo        251.9791 -205.0036  708.9619 0.4025845
## WT+sevo-GDF15 KO+sevo   622.0970  202.3315 1041.8624 0.0038317
## WT+sevo-WT              370.1178 -107.8062  848.0418 0.1552344
```

# Data analysis using R

```
## Present data is ** Fig3 E_GDF15 KO_OCR_state3u.csv **
##
## **  Data structure  **
## 'data.frame':    17 obs. of  3 variables:
##  $ subject: int  1 2 3 4 5 6 7 8 9 10 ...
##  $ group  : chr  "WT" "WT" "WT" "WT+sevo" ...
##  $ state3u: num  67.6 69.4 94.4 126.4 119.4 ...
##
## ** Explorative data analysis with graphics**
```

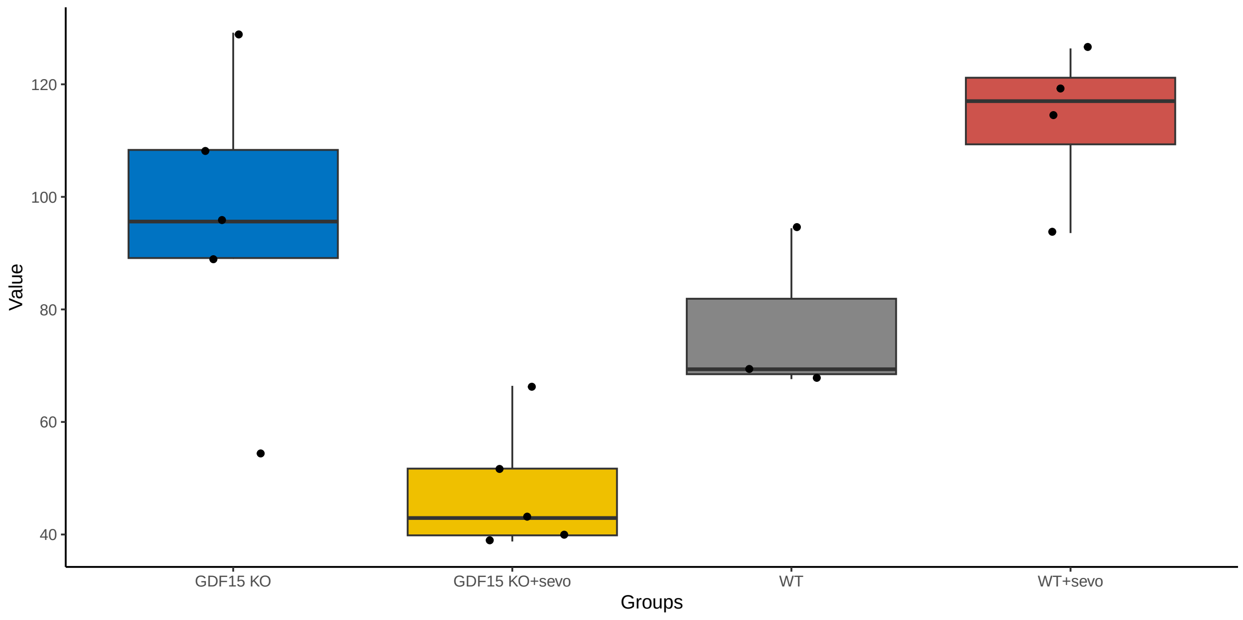


```
## 1. Normality assumption test by Shapiro_Wilk test is
##  p = 0.67
##  Normality assumption was not rejected
## 2. Equal variance test by Bartlett test is
##  p = 0.374
##  Equal variance assumption was not rejected
## 3. The result of anova is
##  p = 0.001
##  A statistically significant difference exist between groups
##
##   Tukey multiple comparisons of means
##     95% family-wise confidence level
##
## Fit: aov(formula = d1[, 3] ~ d1[, 2], data = d1)
##
```

```
## $`d1[, 2]`
##                                diff        lwr       upr     p adj
## GDF15 KO+sevo-GDF15 KO -47.35620 -82.368174 -12.34423 0.0076204
## WT-GDF15 KO            -18.15626 -58.584604  22.27208 0.5682227
## WT+sevo-GDF15 KO        18.20214 -18.933662  55.33794 0.4990946
## WT-GDF15 KO+sevo        29.19994 -11.228402  69.62828 0.1978108
## WT+sevo-GDF15 KO+sevo   65.55834  28.422541 102.69415 0.0008890
## WT+sevo-WT              36.35840  -5.922568  78.63938 0.1024185
```

# Data analysis using R

```
## Present data is ** Fig3 E_GDF15 KO_OCR_state4o.csv **
##
## **  Data structure  **
## 'data.frame':    17 obs. of  3 variables:
##  $ subject: int  1 2 3 4 5 6 7 8 9 10 ...
##  $ group  : chr  "WT" "WT" "WT" "WT+sevo" ...
##  $ state4o: num  198 239 158 193 276 ...
##
## ** Explorative data analysis with graphics**
```

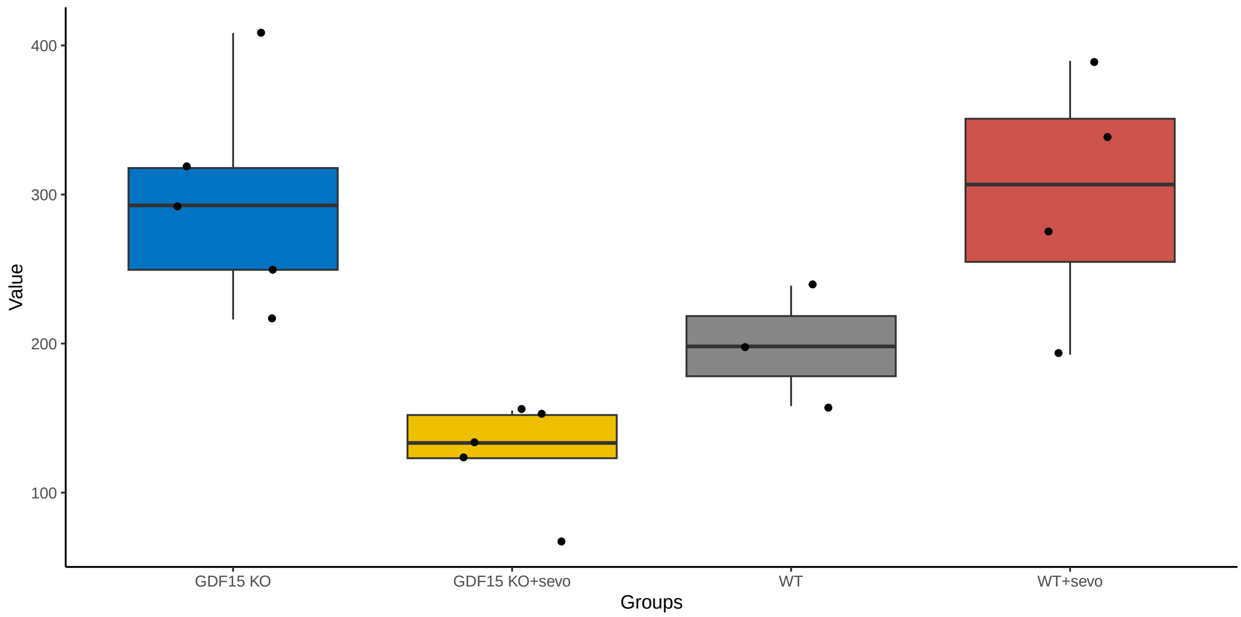


```
## 1. Normality assumption test by Shapiro_Wilk test is
##  p = 0.971
##  Normality assumption was not rejected
## 2. Equal variance test by Bartlett test is
##  p = 0.400
##  Equal variance assumption was not rejected
## 3. The result of anova is
##  p = 0.002
##  A statistically significant difference exist between groups
##
##   Tukey multiple comparisons of means
##     95% family-wise confidence level
##
## Fit: aov(formula = d1[, 3] ~ d1[, 2], data = d1)
##
```

```
## $`d1[, 2]`
##                                diff        lwr       upr     p adj
## GDF15 KO+sevo-GDF15 KO -170.656709 -287.51871 -53.79471 0.0042966
## WT-GDF15 KO             -98.550709 -233.49133  36.38991 0.1906611
## WT+sevo-GDF15 KO          2.012851 -121.93802 125.96372 0.9999590
## WT-GDF15 KO+sevo         72.106000  -62.83462 207.04662 0.4284322
## WT+sevo-GDF15 KO+sevo   172.669561   48.71869 296.62043 0.0061404
## WT+sevo-WT              100.563561  -40.56072 241.68784 0.2066769
```

# Data analysis using R

```
## Present data is ** Figure 3G_GDF15 KO_ATF5.csv **
##
## **  Data structure  **
## 'data.frame':    14 obs. of  3 variables:
##  $ subject: int  1 2 3 4 5 6 7 8 9 10 ...
##  $ group  : chr  "WT" "WT" "WT" "WT" ...
##  $ ATF5   : num  0.955 1.148 0.988 0.909 1.185 ...
##
## ** Explorative data analysis with graphics**
```

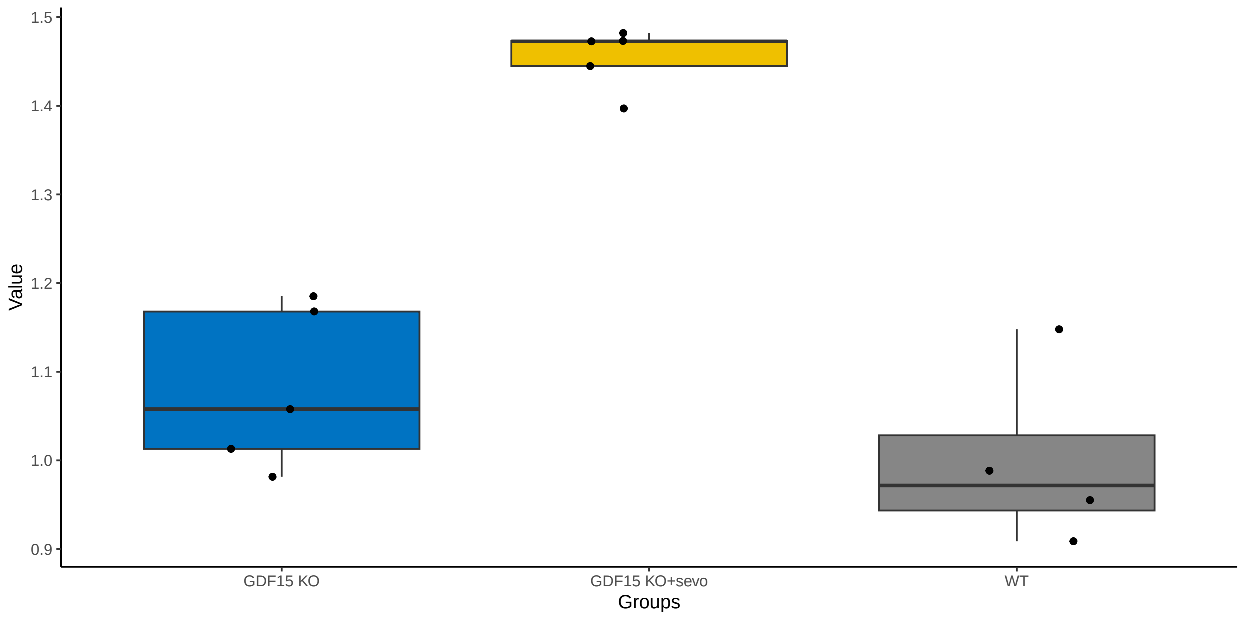


```
## 1. Normality assumption test by Shapiro_Wilk test is
##  p = 0.579
##  Normality assumption was not rejected
## 2. Equal variance test by Bartlett test is
##  p = 0.165
##  Equal variance assumption was not rejected
## 3. The result of anova is
##  p = 0.000
##  A statistically significant difference exist between groups
##
##   Tukey multiple comparisons of means
##     95% family-wise confidence level
##
## Fit: aov(formula = d1[, 3] ~ d1[, 2], data = d1)
##
```

```
## $`d1[, 2]`
##                               diff        lwr        upr     p adj
## GDF15 KO+sevo-GDF15 KO  0.3728038  0.2359159  0.5096918 0.0000393
## WT-GDF15 KO            -0.0811135 -0.2263051  0.0640781 0.3243194
## WT-GDF15 KO+sevo       -0.4539173 -0.5991089 -0.3087257 0.0000107
```

# Data analysis using R

```
## Present data is ** Fig 3 G_GDF15 KO_CLPP.csv **
##
## **  Data structure  **
## 'data.frame':    14 obs. of  3 variables:
##  $ subject: int  1 2 3 4 5 6 7 8 9 10 ...
##  $ group  : chr  "WT" "WT" "WT" "WT" ...
##  $ CLPP   : num  0.753 0.82 1.224 1.202 1.39 ...
##
## ** Explorative data analysis with graphics**
```

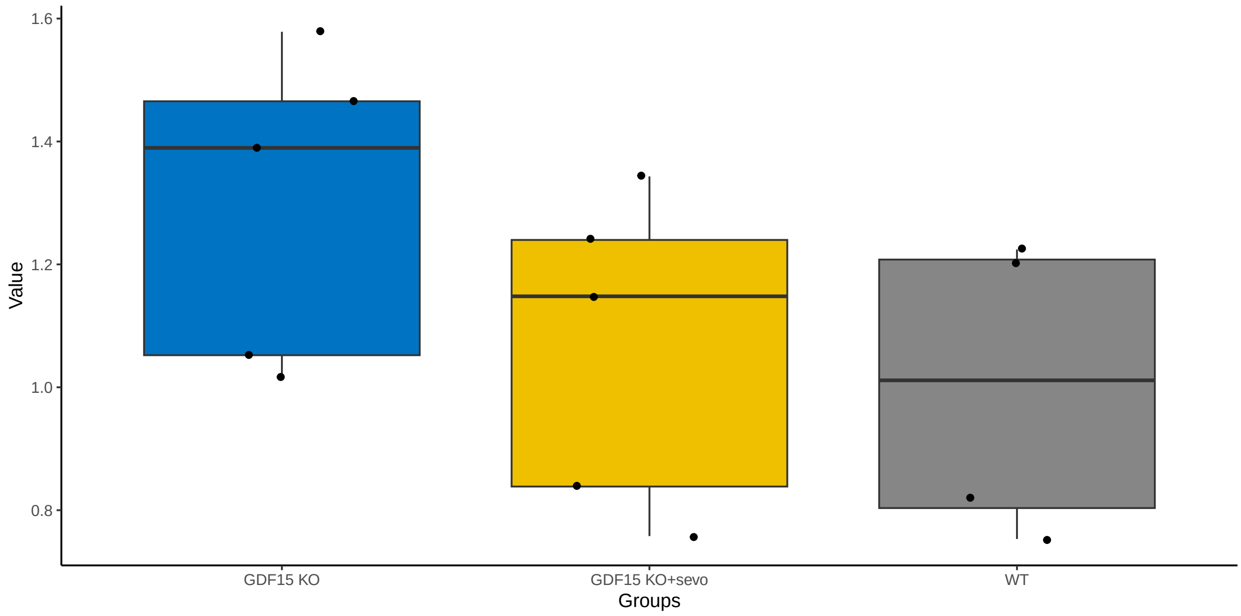


```
## 1. Normality assumption test by Shapiro_Wilk test is
##  p = 0.016
##  Normality assumption was rejected
## 2. The result of Kruskall_Wallis test:
##  p = 0.214
##  A statistically significant difference do not exist between groups
##
```

# Data analysis using R

```
## Present data is ** Fig 3 G_GDF15 KO_HSP60. csv *
*
##
## **  Data structure  **
## 'data.frame':    14 obs. of  3 variables:
##  $ subject: int  1 2 3 4 5 6 7 8 9 10 ...
##  $ group  : chr  "WT" "WT" "WT" "WT" ...
##  $ HSP60  : num  0.964 1.289 1.059 0.688 0.944 ...
##
## ** Explorative data analysis with graphics**
```

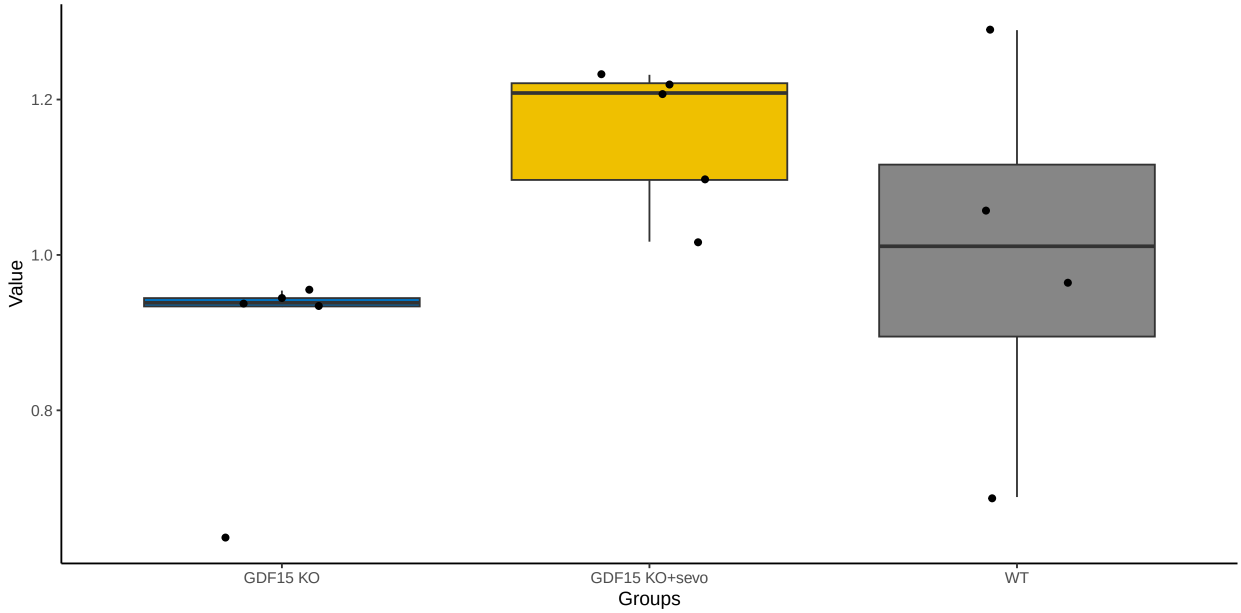


```
## 1. Normality assumption test by Shapiro_Wilk test is
##  p = 0.050
##  Normality assumption was rejected
## 2. The result of Kruskall_Wallis test:
##  p = 0.029
##  A statistically significant difference exist between groups
##
```

# Data analysis using R

```
## Present data is ** Fig 3 G_GDF15 KO_HSP70. csv *
*
##
## **  Data structure  **
## 'data.frame':    14 obs. of  3 variables:
##  $ subject: int  1 2 3 4 5 6 7 8 9 10 ...
##  $ group  : chr  "WT" "WT" "WT" "WT" ...
##  $ HSP70  : num  1.029 1.449 0.744 0.778 1.195 ...
##
## ** Explorative data analysis with graphics**
```

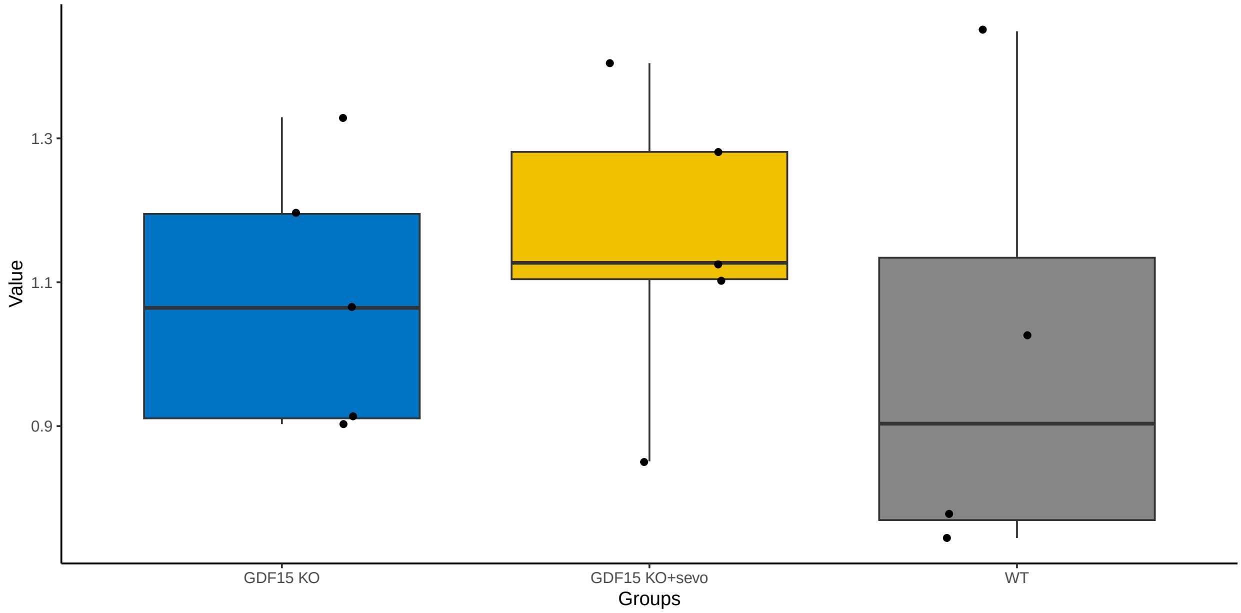


```
## 1. Normality assumption test by Shapiro_Wilk test is
##  p = 0.685
##  Normality assumption was not rejected
## 2. Equal variance test by Bartlett test is
##  p = 0.560
##  Equal variance assumption was not rejected
## 3. The result of anova is
##  p = 0.642
##  A statistically significant difference do not exist between groups
##
```

# Data analysis using R

```
## Present data is ** Fig 3G_GDF15 KO_LONP1. csv **
##
## **  Data structure  **
## 'data.frame':    14 obs. of  3 variables:
##  $ subject: int  1 2 3 4 5 6 7 8 9 10 ...
##  $ group  : chr  "WT" "WT" "WT" "WT" ...
## $ LONP1 : num 0.986 0.991 1.065 0.958 1.201 ...
##
## ** Explorative data analysis with graphics**
```

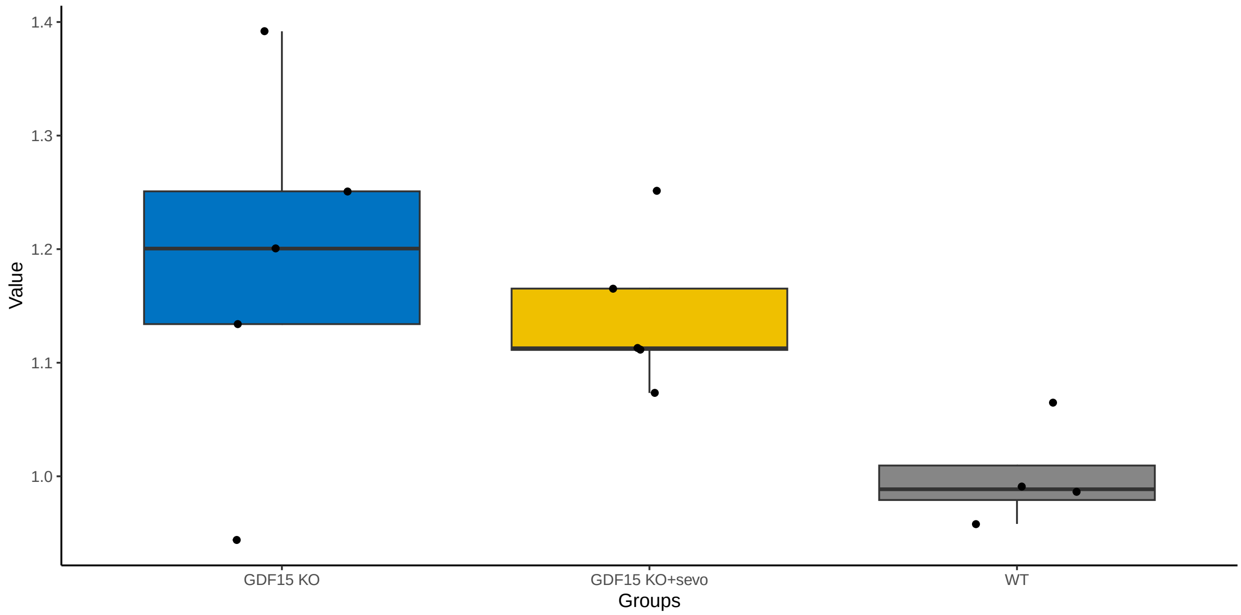


```
## 1. Normality assumption test by Shapiro_Wilk test is
##  p = 0.323
##  Normality assumption was not rejected
## 2. Equal variance test by Bartlett test is
##  p = 0.075
##  Equal variance assumption was not rejected
## 3. The result of anova is
##  p = 0.074
##  A statistically significant difference do not exist between groups
##
```

# Data analysis using R

```
## Present data is ** Fig3 H_serum GDF15_B6.csv **
##
## **  Data structure  **
## 'data.frame':    25 obs. of  3 variables:
##  $ subject: int  1 2 3 4 5 6 7 8 9 10 ...
##  $ group  : chr  "base" "base" "base" "base" ...
##  $ GDF15  : num  47.9 43.3 45.6 53.9 47.2 ...
##
## ** Explorative data analysis with graphics**
```

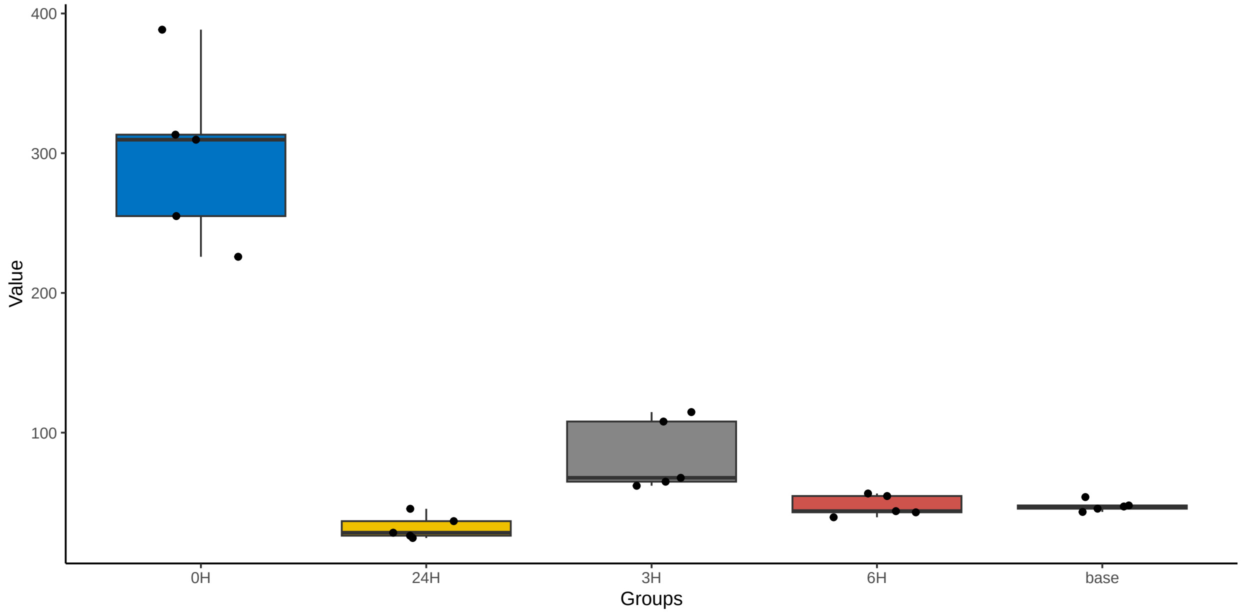


```
## 1. Normality assumption test by Shapiro_Wilk test is
##  p = 0.003
##  Normality assumption was rejected
## 2. The result of Kruskall_Wallis test:
##  p = 0.000
##  A statistically significant difference exist between groups
##
```

# Data analysis using R

```
## Present data is ** Fig3 I_serum GDF15_ATF5 WT.csv *
*
##
## **  Data structure  **
## 'data.frame':    17 obs. of  3 variables:
##  $ subject: int  1 2 3 4 5 6 7 8 9 10 ...
##  $ group  : chr  "base" "base" "base" "0H" ...
##  $ GDF15  : num  33.3 26.5 40.5 658.1 230.4 ...
##
## ** Explorative data analysis with graphics**
```

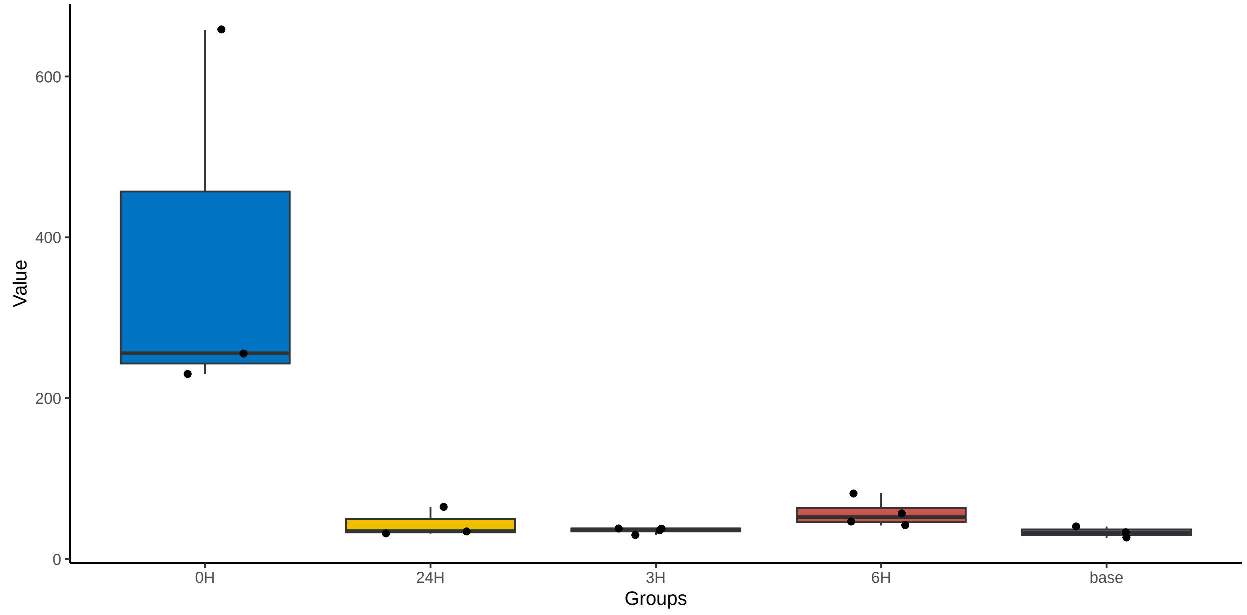


```
## 1. Normality assumption test by Shapiro_Wilk test is
##  p = 0.000
##  Normality assumption was rejected
## 2. The result of Kruskall_Wallis test:
##  p = 0.023
##  A statistically significant difference exist between groups
##
```

# Data analysis using R

```
## Present data is ** Fig3 J_serum GDF15_ATF5 cKO.csv *
*
##
## **  Data structure  **
## 'data.frame':    15 obs. of  3 variables:
##  $ subject: int  1 2 3 4 5 6 7 8 9 10 ...
##  $ group  : chr  "base" "base" "base" "0H" ...
##  $ GDF15  : num  38.1 45.8 43.2 207.7 284.4 ...
##
## ** Explorative data analysis with graphics**
```

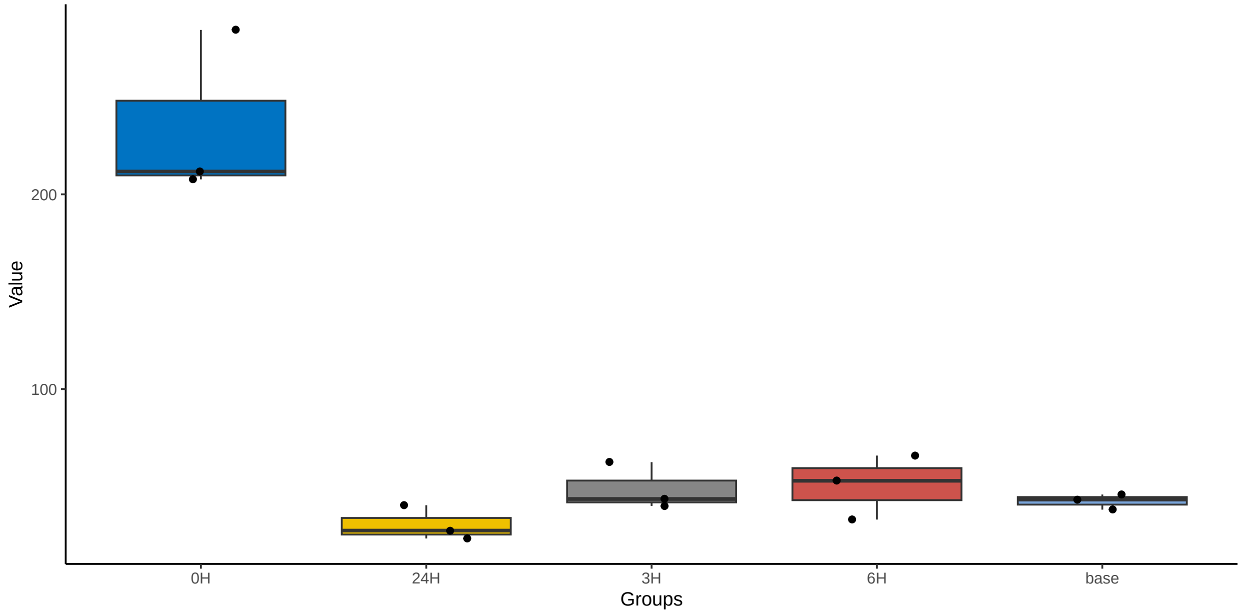


```
## 1. Normality assumption test by Shapiro_Wilk test is
##  p = 0.13
##  Normality assumption was not rejected
## 2. Equal variance test by Bartlett test is
##  p = 0.054
##  Equal variance assumption was not rejected
## 3. The result of anova is
##  p = 0.000
##  A statistically significant difference exist between groups
##
##   Tukey multiple comparisons of means
##     95% family-wise confidence level
##
## Fit: aov(formula = d1[, 3] ~ d1[, 2], data = d1)
##
```

```
## $`d1[, 2]`
##                diff        lwr        upr     p adj
## 24H-0H   -204.31924 -262.93854 -145.69994 0.0000035
## 3H-0H    -185.94795 -244.56724 -127.32865 0.0000083
## 6H-0H    -184.04393 -242.66322 -125.42463 0.0000091
## base-0H  -192.23530 -250.85459 -133.61600 0.0000061
## 3H-24H     18.37129  -40.24800   76.99059 0.8355421
## 6H-24H     20.27531  -38.34398   78.89461 0.7837931
## base-24H   12.08394  -46.53535   70.70324 0.9567712
## 6H-3H       1.90402  -56.71528   60.52332 0.9999648
## base-3H    -6.28735  -64.90665   52.33195 0.9961048
## base-6H    -8.19137  -66.81067   50.42793 0.9893763
```

# Data analysis using R

```
## Present data is ** Fig3 K_serum GDF15_B6_WT_ATF5 cKO_post OH.csv * *
##
## **  Data structure  **
## 'data.frame':    11 obs. of  3 variables:
##  $ subject: int  1 2 3 4 5 6 7 8 9 10 ...
##  $ group  : chr  "B6" "B6" "B6" "B6" ...
##  $ GDF15  : num  388 313 310 255 226 ...
##
## ** Explorative data analysis with graphics**
```

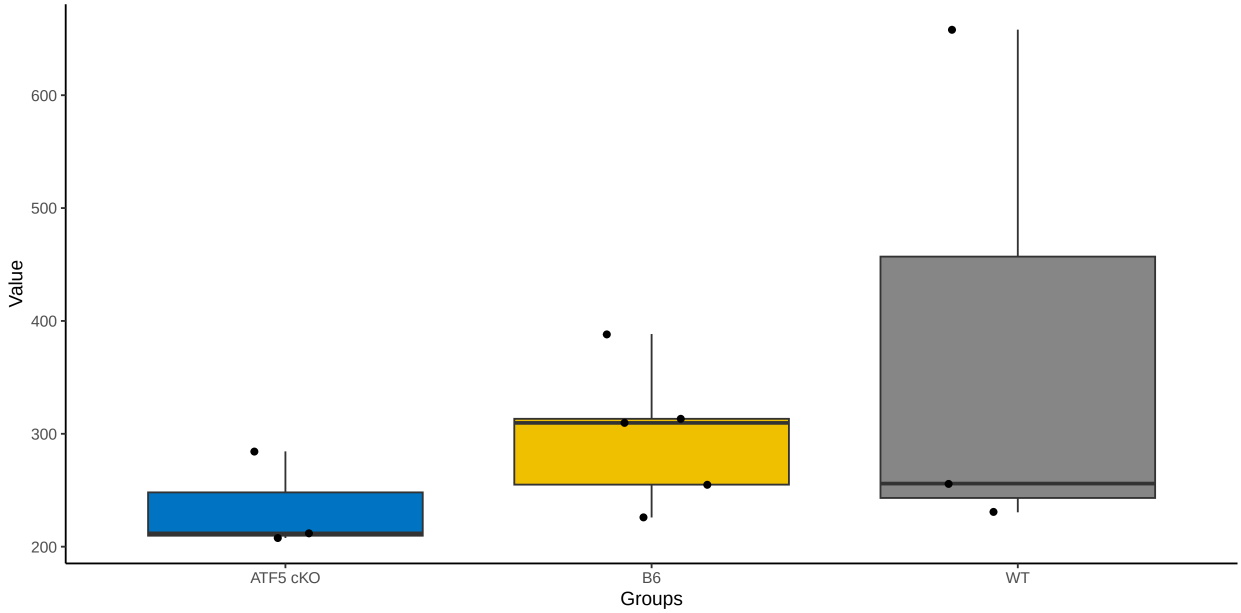


```
## 1. Normality assumption test by Shapiro_Wilk test is
##  p = 0.24
##  Normality assumption was not rejected
## 2. Equal variance test by Bartlett test is
##  p = 0.038
##  Equal variance assumption was rejected
## 3. The result of Welch ANOVA is
##  p = 0.316
##  A statistically significant difference do not exist between groups
##
```

# Data analysis using R

```
## Present data is ** Fig4B_virus cortex injection_ATF5.csv **
##
## **  Data structure  **
## 'data.frame':    10 obs. of  3 variables:
##  $ subject: int  1 2 3 4 5 6 7 8 9 10
##  $ group  : chr  "con virus" "con virus" "con virus" "con virus" ...
##  $ ATF5   : num  1.144 0.963 0.969 0.684 1.24 ...
##
## ** Explorative data analysis with graphics**
```

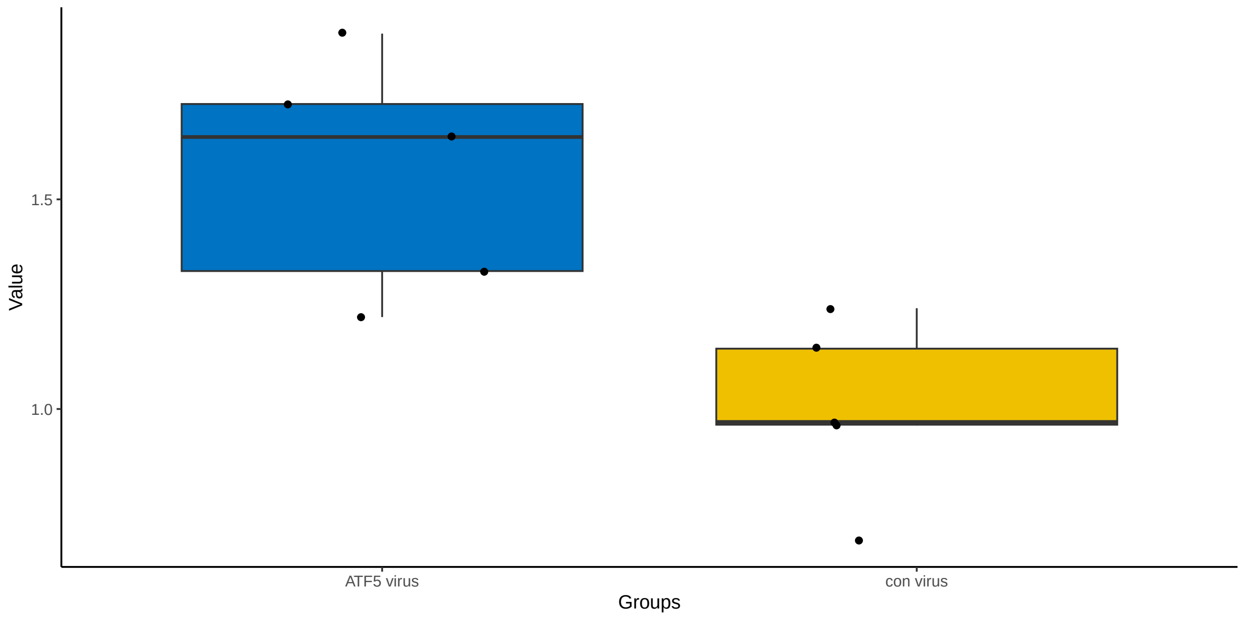


```
## 1. Normality assumption test by Shapiro_Wilk test is
##  p = 0.514
##  Normality assumption was not rejected
## 2. Equal variance test by Bartlett test is
##  p = 0.597
##  Equal variance assumption was not rejected
## 3. The result of anova is
##  p = 0.007
##  A statistically significant difference exist between groups
##
##   Tukey multiple comparisons of means
##     95% family-wise confidence level
##
## Fit: aov(formula = d1[, 3] ~ d1[, 2], data = d1)
##
```

```
## $`d1[, 2]`
##                             diff        lwr        upr     p adj
## con virus-ATF5 virus -0.563929 -0.9279141 -0.1999438 0.0072647
```

# Data analysis using R

```
## Present data is ** Fig4B_virus cortex injection_CLPP.csv **
##
## **  Data structure  **
## 'data.frame':    10 obs. of  3 variables:
##  $ subject: int  1 2 3 4 5 6 7 8 9 10
##  $ group  : chr  "con virus" "con virus" "con virus" "con virus" ...
##  $ CLPP   : num  0.895 1.498 0.865 0.854 0.887 ...
##
## ** Explorative data analysis with graphics**
```

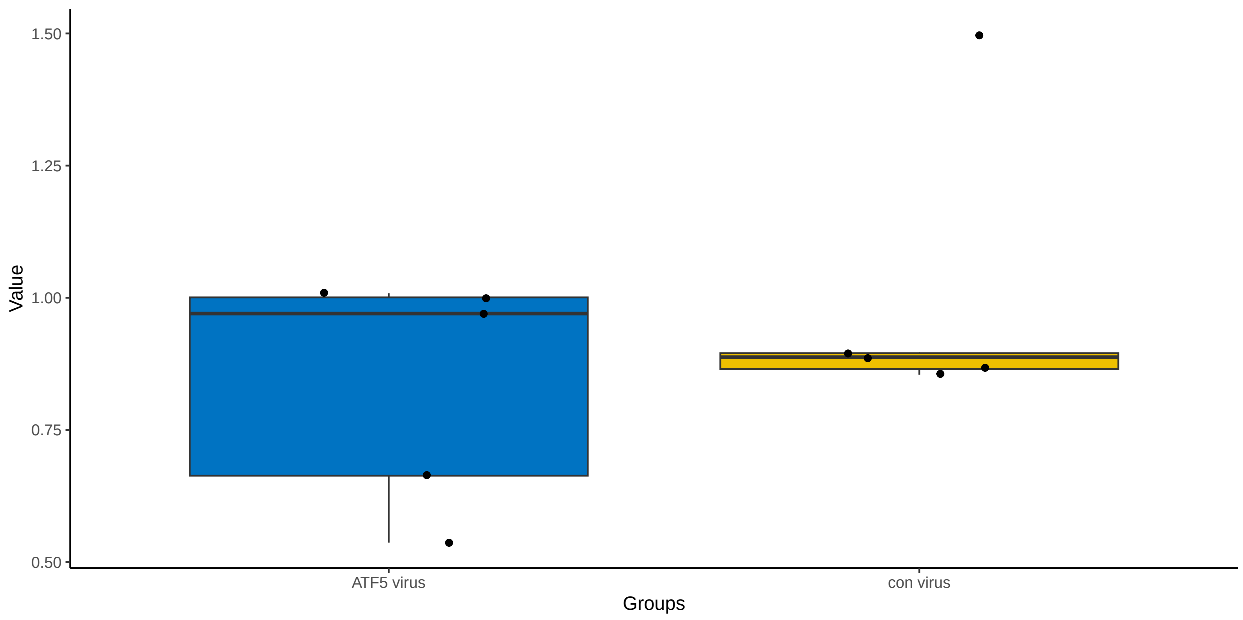


```
## 1. Normality assumption test by Shapiro_Wilk test is
##  p = 0.171
##  Normality assumption was not rejected
## 2. Equal variance test by Bartlett test is
##  p = 0.658
##  Equal variance assumption was not rejected
## 3. The result of anova is
##  p = 0.332
##  A statistically significant difference do not exist between groups
##
```

# Data analysis using R

```
## Present data is ** Fig4B_virus cortex injection_HSP60.csv **
##
## **  Data structure  **
## 'data.frame':    10 obs. of  3 variables:
##  $ subject: int  1 2 3 4 5 6 7 8 9 10
##  $ group  : chr  "con virus" "con virus" "con virus" "con virus" ...
##  $ HSP60  : num  0.888 1.133 0.895 1.152 0.932 ...
##
## ** Explorative data analysis with graphics**
```

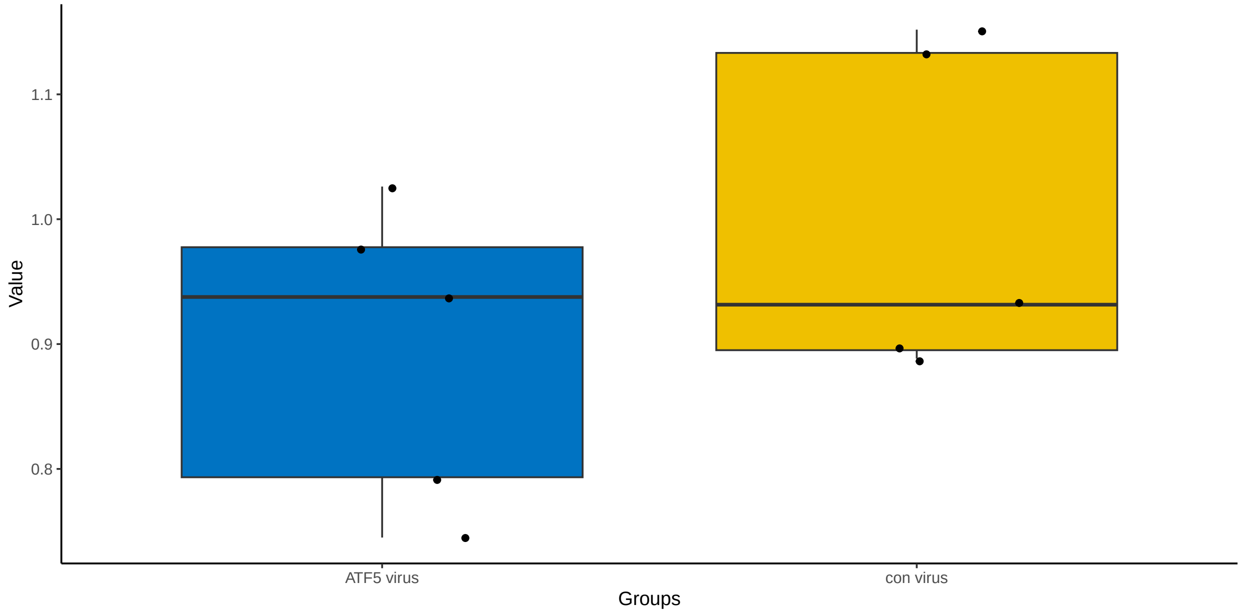


```
## 1. Normality assumption test by Shapiro_Wilk test is
##  p = 0.081
##  Normality assumption was not rejected
## 2. Equal variance test by Bartlett test is
##  p = 0.879
##  Equal variance assumption was not rejected
## 3. The result of anova is
##  p = 0.229
##  A statistically significant difference do not exist between groups
##
```

# Data analysis using R

```
## Present data is ** Fig4B_virus cortex injection_HSP70.csv **
##
## **  Data structure  **
## 'data.frame':    10 obs. of  3 variables:
##  $ subject: int  1 2 3 4 5 6 7 8 9 10
##  $ group  : chr  "con virus" "con virus" "con virus" "con virus" ...
##  $ HSP70  : num  0.746 1.478 1.021 0.968 0.787 ...
##
## ** Explorative data analysis with graphics**
```

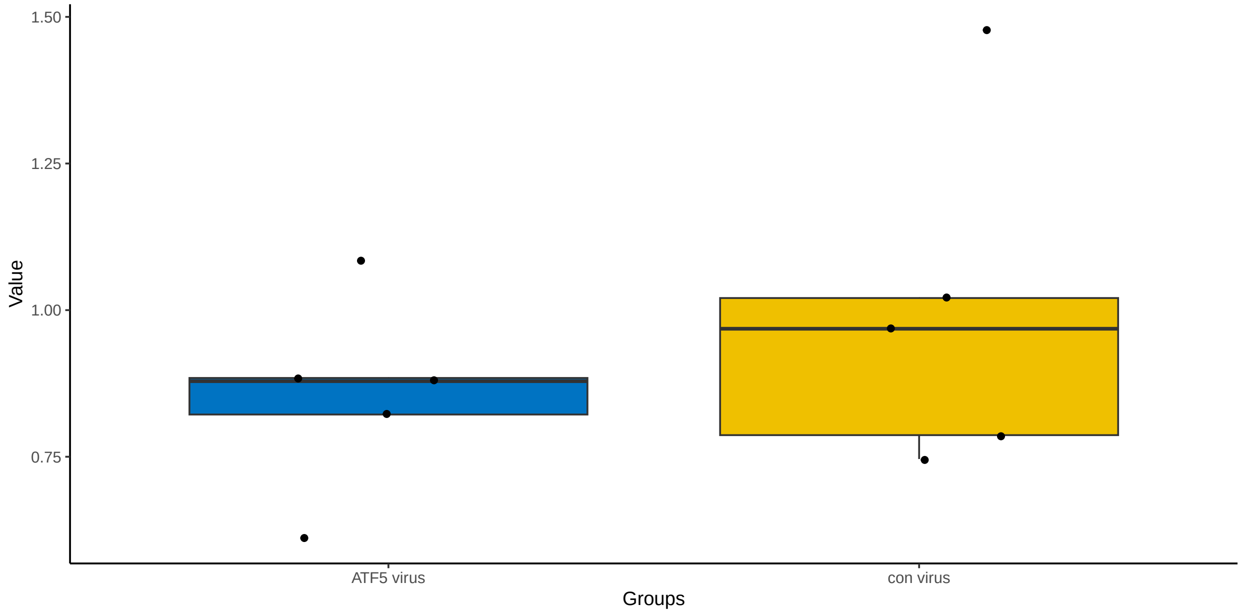


```
## 1. Normality assumption test by Shapiro_Wilk test is
##  p = 0.175
##  Normality assumption was not rejected
## 2. Equal variance test by Bartlett test is
##  p = 0.310
##  Equal variance assumption was not rejected
## 3. The result of anova is
##  p = 0.367
##  A statistically significant difference do not exist between groups
##
```

# Data analysis using R

```
## Present data is ** Fig4B_virus cortex injection_LONP1.csv **
##
## **  Data structure  **
## 'data.frame':    10 obs. of  3 variables:
##  $ subject: int  1 2 3 4 5 6 7 8 9 10
##  $ group  : chr  "con virus" "con virus" "con virus" "con virus" ...
##  $ LONP1  : num  0.597 1.092 1.128 1.087 1.095 ...
##
## ** Explorative data analysis with graphics**
```

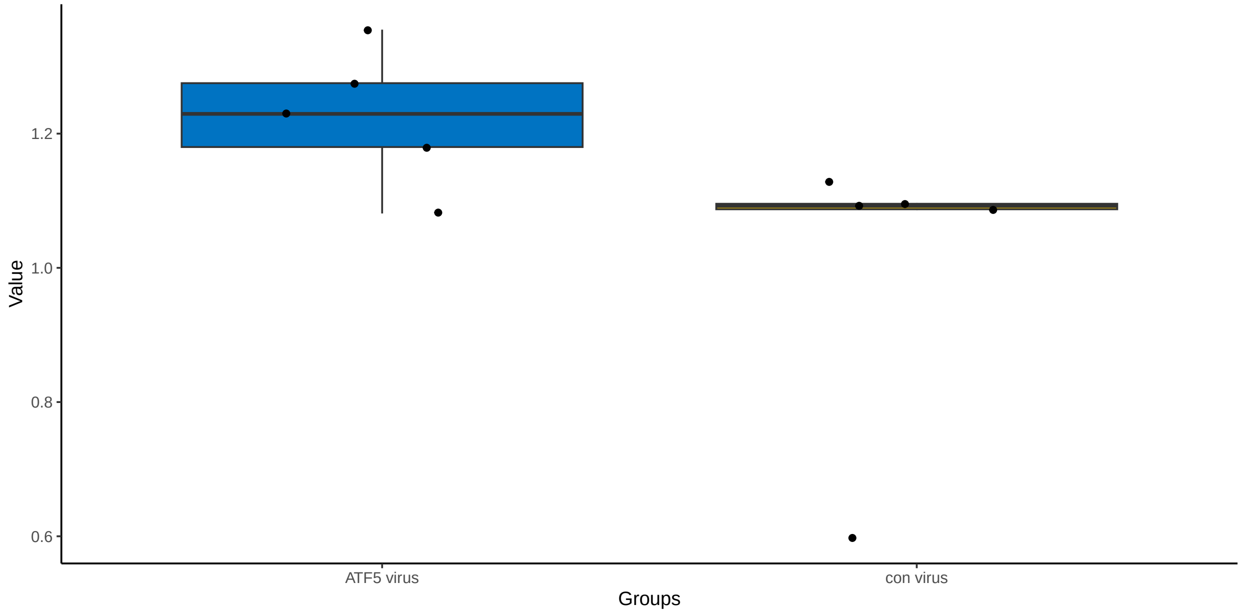


```
## 1. Normality assumption test by Shapiro_Wilk test is
##  p = 0.008
##  Normality assumption was rejected
## 2. The result of Kruskall_Wallis test:
##  p = 0.076
##  A statistically significant difference do not exist between groups
##
```

# Data analysis using R

```
## Present data is ** Fig4 D_ATF5 virus_OCR_Basal.csv **
##
## **  Data structure  **
## 'data.frame':    10 obs. of  3 variables:
##  $ subject: int  1 2 3 4 5 6 7 8 9 10
##  $ group  : chr  "CON virus" "CON virus" "CON virus" "CON virus" ...
##  $ basal  : num  187 114 104 138 114 ...
##
## ** Explorative data analysis with graphics**
```

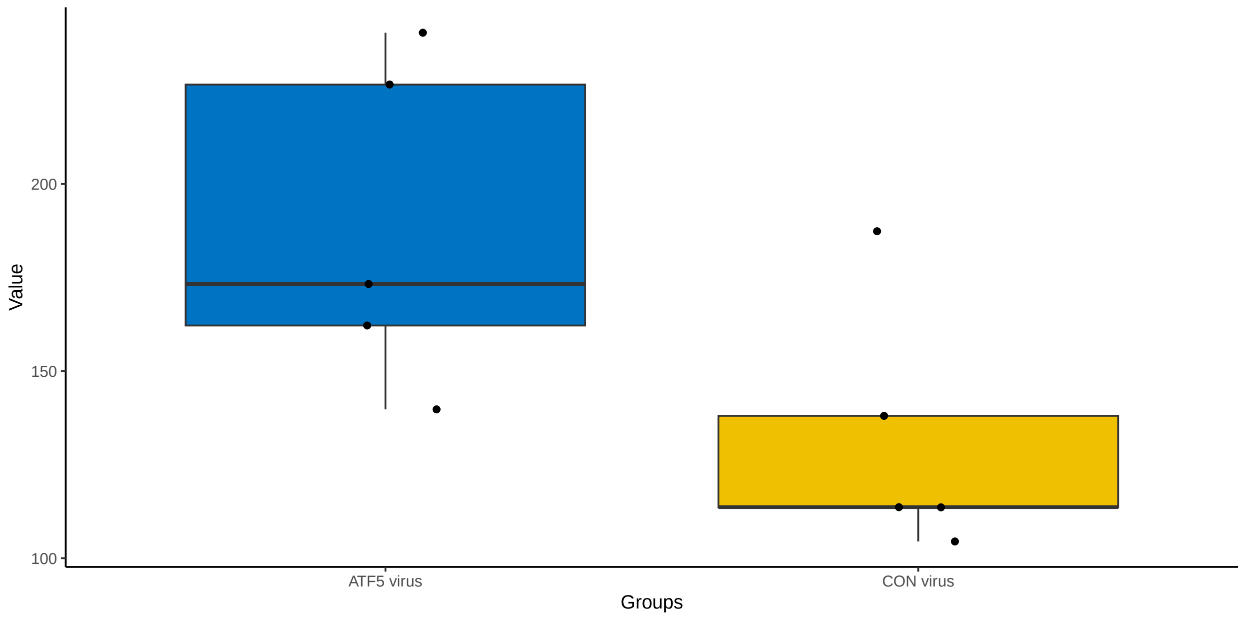


```
## 1. Normality assumption test by Shapiro_Wilk test is
##  p = 0.145
##  Normality assumption was not rejected
## 2. Equal variance test by Bartlett test is
##  p = 0.642
##  Equal variance assumption was not rejected
## 3. The result of anova is
##  p = 0.048
##  A statistically significant difference exist between groups
##
##   Tukey multiple comparisons of means
##     95% family-wise confidence level
##
## Fit: aov(formula = d1[, 3] ~ d1[, 2], data = d1)
##
```

```
## $`d1[, 2]`
##                              diff       lwr        upr     p adj
## CON virus-ATF5 virus -57.01848 -113.4592 -0.5777531 0.0481911
```

# Data analysis using R

```
## Present data is ** Fig4 D_ATF5 virus_OCR_State3.csv **
##
## **  Data structure  **
## 'data.frame':    10 obs. of  3 variables:
##  $ subject: int  1 2 3 4 5 6 7 8 9 10
##  $ group  : chr  "CON virus" "CON virus" "CON virus" "CON virus" ...
##  $ state3 : num  434 308 327 372 239 ...
##
## ** Explorative data analysis with graphics**
```

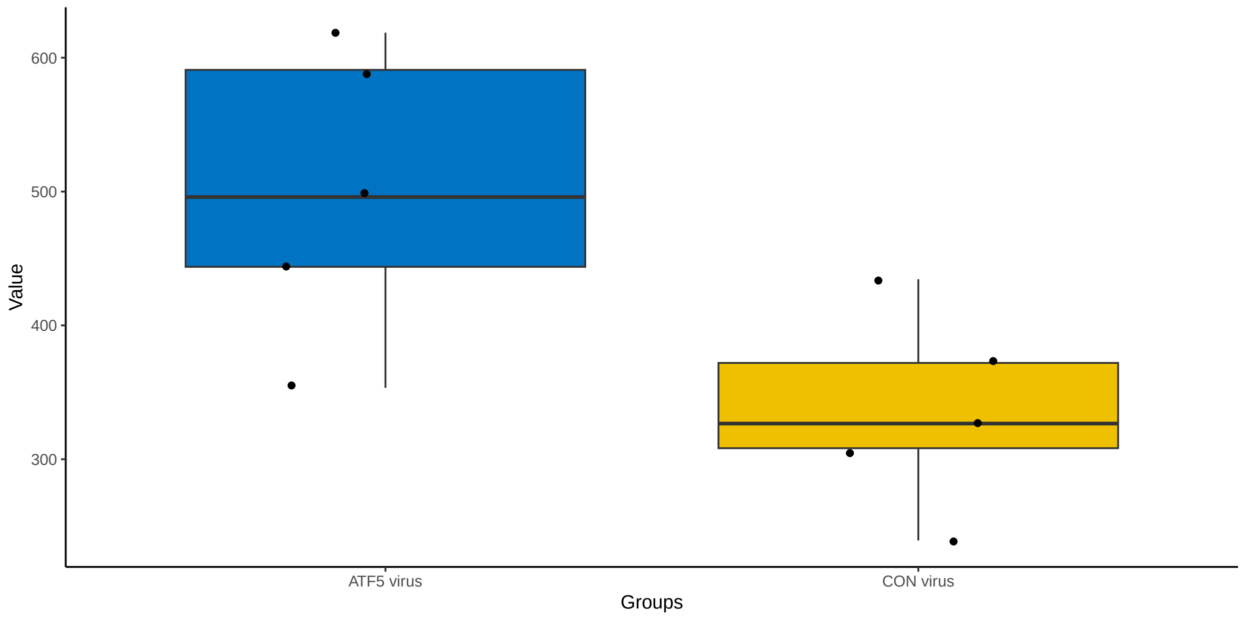


```
## 1. Normality assumption test by Shapiro_Wilk test is
##  p = 0.791
##  Normality assumption was not rejected
## 2. Equal variance test by Bartlett test is
##  p = 0.458
##  Equal variance assumption was not rejected
## 3. The result of anova is
##  p = 0.023
##  A statistically significant difference exist between groups
##
##   Tukey multiple comparisons of means
##     95% family-wise confidence level
##
## Fit: aov(formula = d1[, 3] ~ d1[, 2], data = d1)
##
```

# Data analysis using R

```
## Present data is ** Fig4 D_ATF5 virus_OCR_State3u.csv **
##
## **  Data structure  **
## 'data.frame':    10 obs. of  3 variables:
##  $ subject: int  1 2 3 4 5 6 7 8 9 10
##  $ group  : chr  "CON virus" "CON virus" "CON virus" "CON virus" ...
##  $ state3u: num  415 293 265 339 232 ...
##
## ** Explorative data analysis with graphics**
```

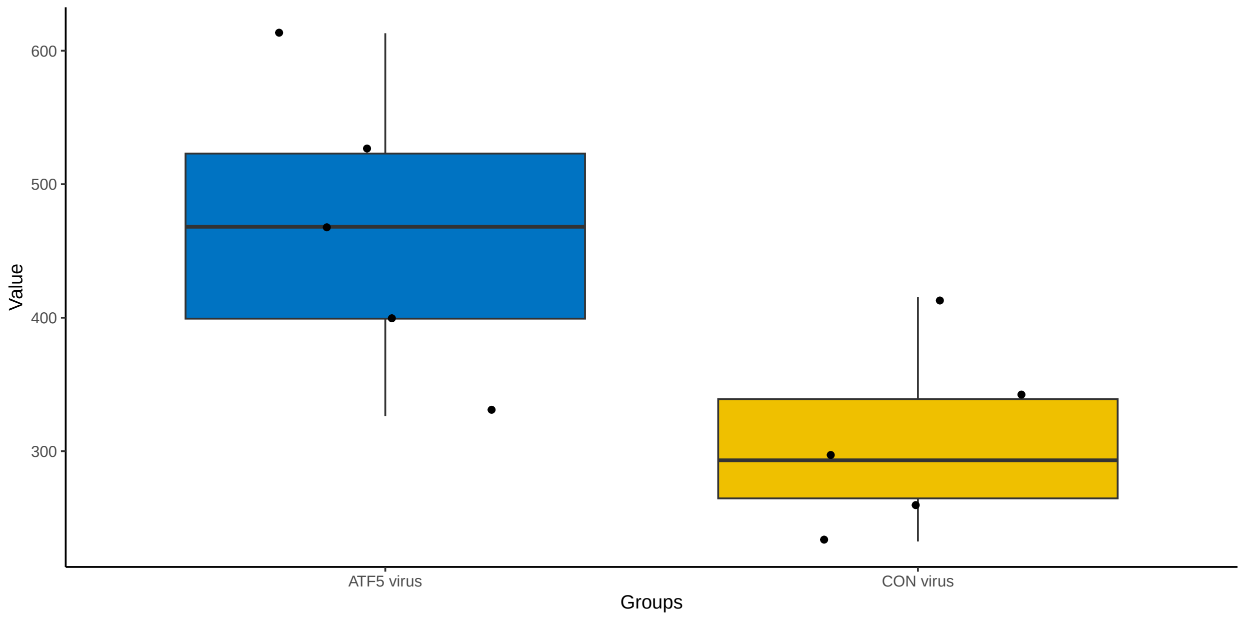


```
## 1. Normality assumption test by Shapiro_Wilk test is
##  p = 0.988
##  Normality assumption was not rejected
## 2. Equal variance test by Bartlett test is
##  p = 0.415
##  Equal variance assumption was not rejected
## 3. The result of anova is
##  p = 0.028
##  A statistically significant difference exist between groups
##
##   Tukey multiple comparisons of means
##     95% family-wise confidence level
##
## Fit: aov(formula = d1[, 3] ~ d1[, 2], data = d1)
##
```

```
## $`d1[, 2]`
##                              diff       lwr       upr     p adj
## CON virus-ATF5 virus -157.0807 -292.6647 -21.49682 0.0282911
```

# Data analysis using R

```
## Present data is ** Fig4 D_ATF5 virus_OCR_State4o.csv **
##
## **  Data structure  **
## 'data.frame':    10 obs. of  3 variables:
##  $ subject: int  1 2 3 4 5 6 7 8 9 10
##  $ group  : chr  "CON virus" "CON virus" "CON virus" "CON virus" ...
##  $ state4o: num  94.1 78.5 76.9 93.8 47.7 ...
##
## ** Explorative data analysis with graphics**
```

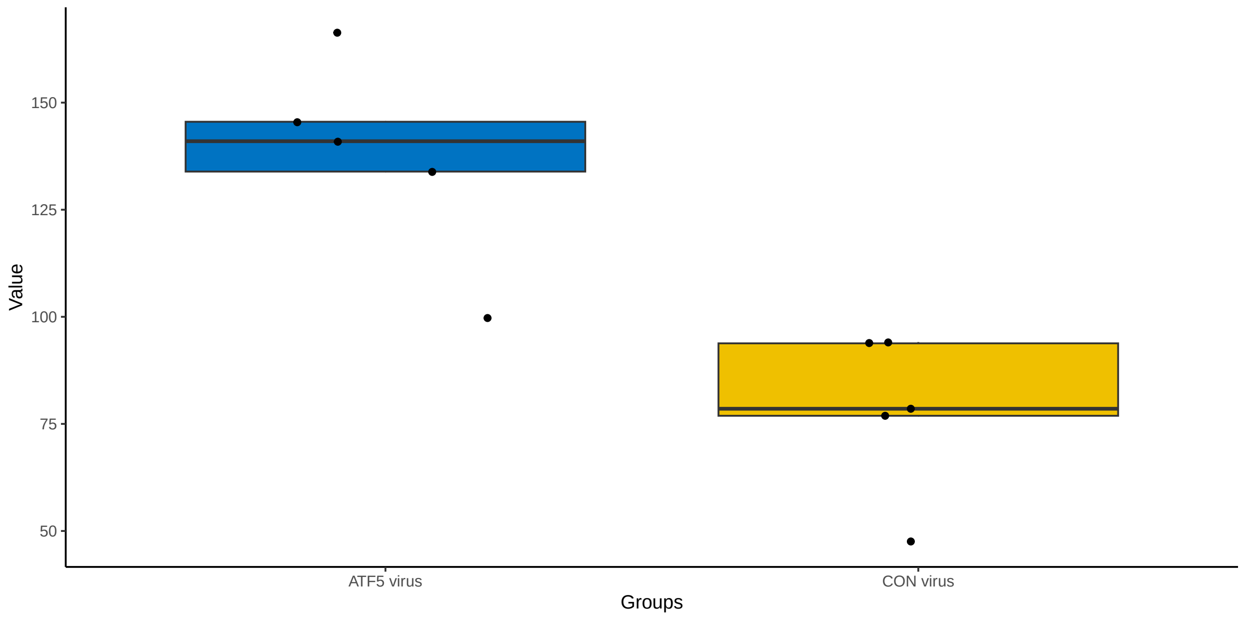


```
## 1. Normality assumption test by Shapiro_Wilk test is
##  p = 0.333
##  Normality assumption was not rejected
## 2. Equal variance test by Bartlett test is
##  p = 0.644
##  Equal variance assumption was not rejected
## 3. The result of anova is
##  p = 0.003
##  A statistically significant difference exist between groups
##
##   Tukey multiple comparisons of means
##     95% family-wise confidence level
##
## Fit: aov(formula = d1[, 3] ~ d1[, 2], data = d1)
##
```

```
## $`d1[, 2]`
##                            diff      lwr      upr     p adj
## CON virus-ATF5 virus -59.10145 -90.7804 -27.4225 0.0026081
```

# Data analysis using R

```
## Present data is ** Fig5C_virus iv injection_ATF5.csv **
##
## **  Data structure  **
## 'data.frame':    10 obs. of  3 variables:
##  $ subject: int  1 2 3 4 5 6 7 8 9 10
##  $ group  : chr  "con virus" "con virus" "con virus" "con virus" ...
##  $ ATF5   : num  1.083 0.949 0.653 0.857 1.457 ...
##
## ** Explorative data analysis with graphics**
```

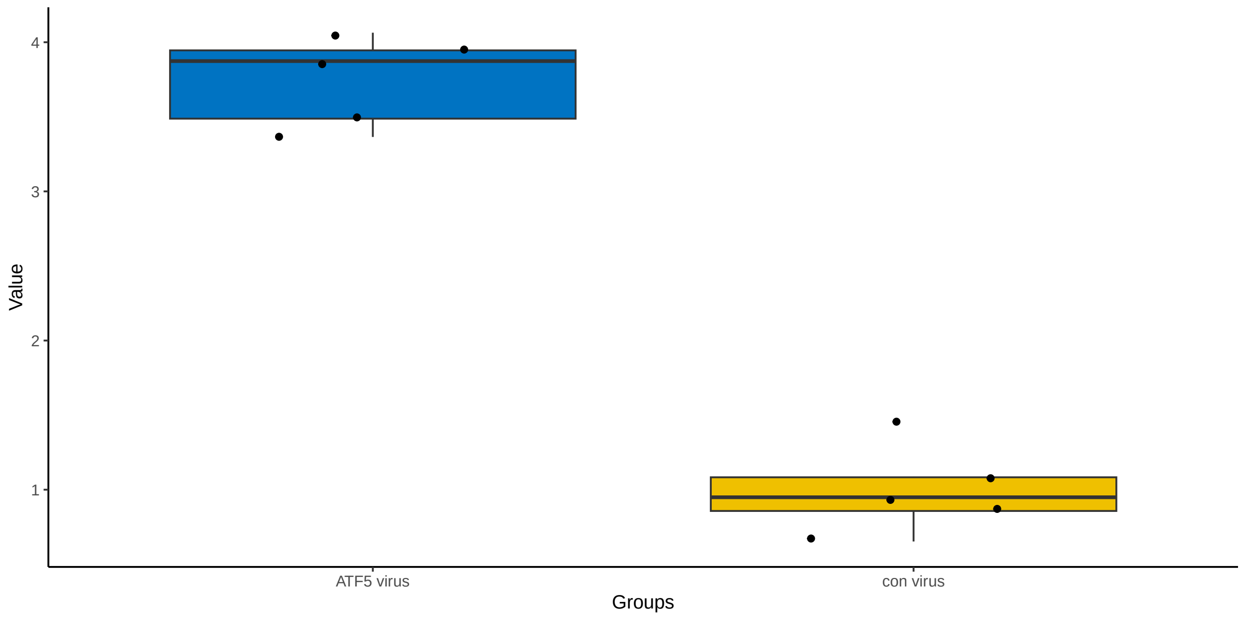


```
## 1. Normality assumption test by Shapiro_Wilk test is
##  p = 0.793
##  Normality assumption was not rejected
## 2. Equal variance test by Bartlett test is
##  p = 0.981
##  Equal variance assumption was not rejected
## 3. The result of anova is
##  p = 0.000
##  A statistically significant difference exist between groups
##
##   Tukey multiple comparisons of means
##     95% family-wise confidence level
##
## Fit: aov(formula = d1[, 3] ~ d1[, 2], data = d1)
##
```

```
## $`d1[, 2]`
##                             diff       lwr       upr p adj
## con virus-ATF5 virus -2.747173 -3.187309 -2.307037 5e-07
```

# Data analysis using R

```
## Present data is ** Fig5D qPCR_GDF15_Emx1 cre_ATF5 virus IV.csv **
##
## **  Data structure  **
## 'data.frame':    10 obs. of  3 variables:
##  $ subject         : int  1 2 3 4 5 6 7 8 9 10
##  $ group           : chr  "con virus" "con virus" "con virus" "con virus" ...
##  $ GDF15.beta.actin: num  1.057 1.092 1.04 0.977 0.835 ...
##
## ** Explorative data analysis with graphics**
```

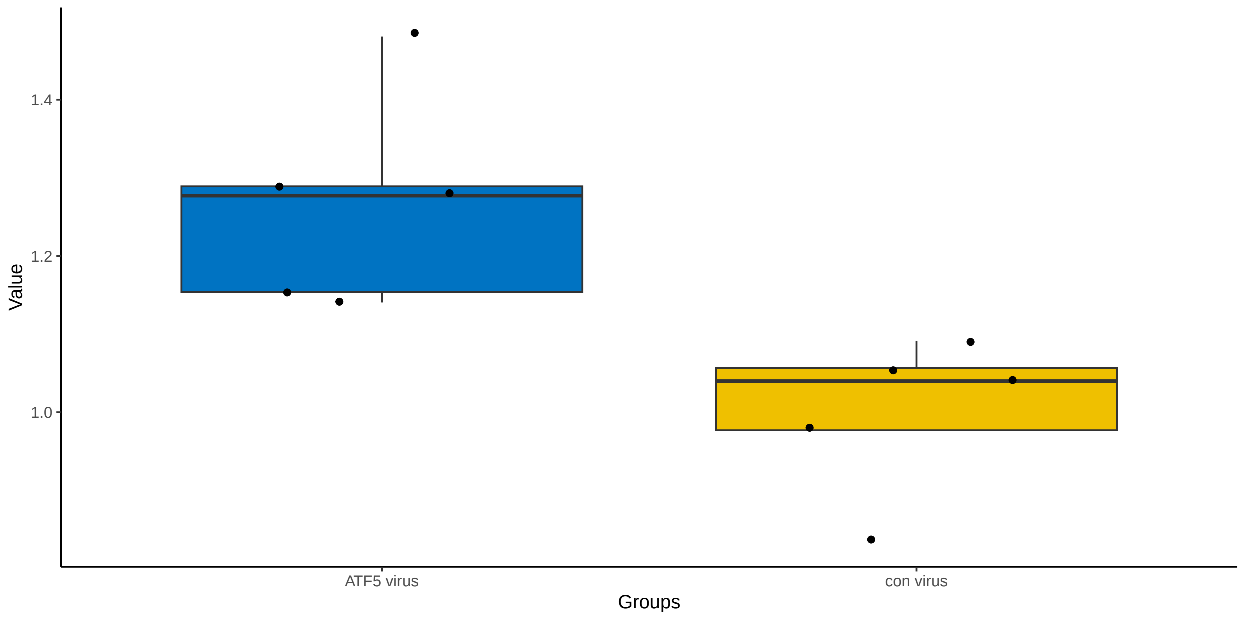


```
## 1. Normality assumption test by Shapiro_Wilk test is
##  p = 0.777
##  Normality assumption was not rejected
## 2. Equal variance test by Bartlett test is
##  p = 0.571
##  Equal variance assumption was not rejected
## 3. The result of anova is
##  p = 0.008
##  A statistically significant difference exist between groups
##
##   Tukey multiple comparisons of means
##     95% family-wise confidence level
##
## Fit: aov(formula = d1[, 3] ~ d1[, 2], data = d1)
##
```

```
## $`d1[, 2]`
##                             diff        lwr         upr   p adj
## con virus-ATF5 virus -0.2682744 -0.4439558 -0.09259296 0.00783
```

# Data analysis using R

```
## Present data is ** Figure 5 E serum level of GDF15 virus iv injection_csv.csv **
##
## **  Data structure  **
## 'data.frame':    10 obs. of  3 variables:
##  $ subject: int  1 2 3 4 5 6 7 8 9 10
##  $ group  : chr  "con virus" "con virus" "con virus" "con virus" ...
##  $ GDF15  : num  135.7 127.7 80.5 63.3 234.7 ...
##
## ** Explorative data analysis with graphics**
```

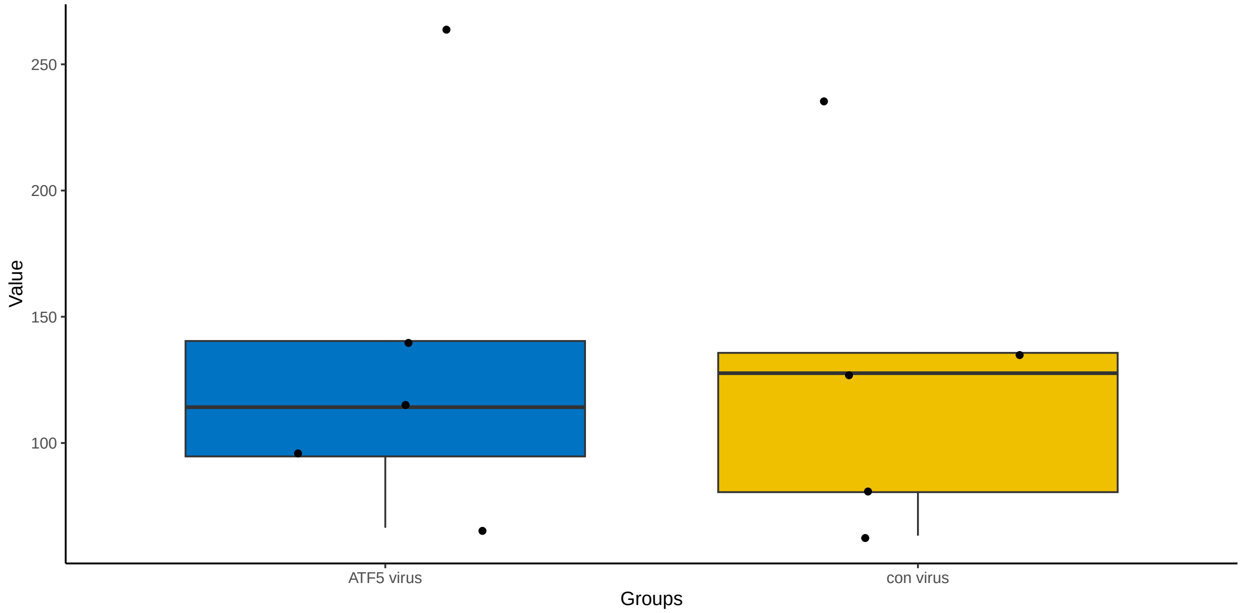


```
## 1. Normality assumption test by Shapiro_Wilk test is
##  p = 0.068
##  Normality assumption was not rejected
## 2. Equal variance test by Bartlett test is
##  p = 0.805
##  Equal variance assumption was not rejected
## 3. The result of anova is
##  p = 0.874
##  A statistically significant difference do not exist between groups
##
```

# Data analysis using R

```
## Present data is ** Fig5 G_cortex infarct volume_Emx1 cre_ATF5 virus IV.csv *
*
##
## **  Data structure  **
## 'data.frame':    9 obs. of  3 variables:
##  $ subject: int  1 2 3 4 5 6 7 8 9
##  $ group  : chr  "con virus" "con virus" "con virus" "con virus" ...
##  $ infarct: num  0.516 0.361 0.512 0.433 0.528 ...
##
## ** Explorative data analysis with graphics**
```

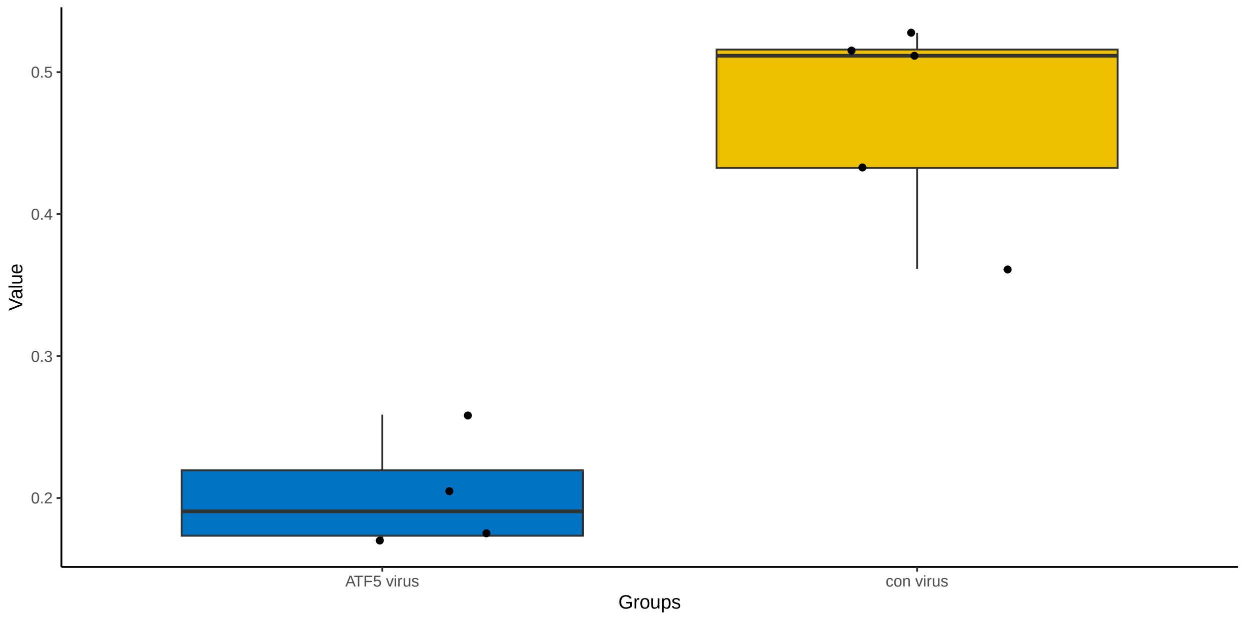


```
## 1. Normality assumption test by Shapiro_Wilk test is
##  p = 0.208
##  Normality assumption was not rejected
## 2. Equal variance test by Bartlett test is
##  p = 0.361
##  Equal variance assumption was not rejected
## 3. The result of anova is
##  p = 0.000
##  A statistically significant difference exist between groups
##
##   Tukey multiple comparisons of means
##     95% family-wise confidence level
##
## Fit: aov(formula = d1[, 3] ~ d1[, 2], data = d1)
##
```

# Data analysis using R

```
## Present data is ** Fig5 G_neurological score_Emx1 cre_ATF5 virus IV.csv *
*
##
## **  Data structure  **
## 'data.frame':    9 obs. of  3 variables:
##  $ subject: int  1 2 3 4 5 6 7 8 9
##  $ group  : chr  "con virus" "con virus" "con virus" "con virus" ...
##  $ score  : int  3 2 3 3 3 1 1 1 2
##
## ** Explorative data analysis with graphics**
```

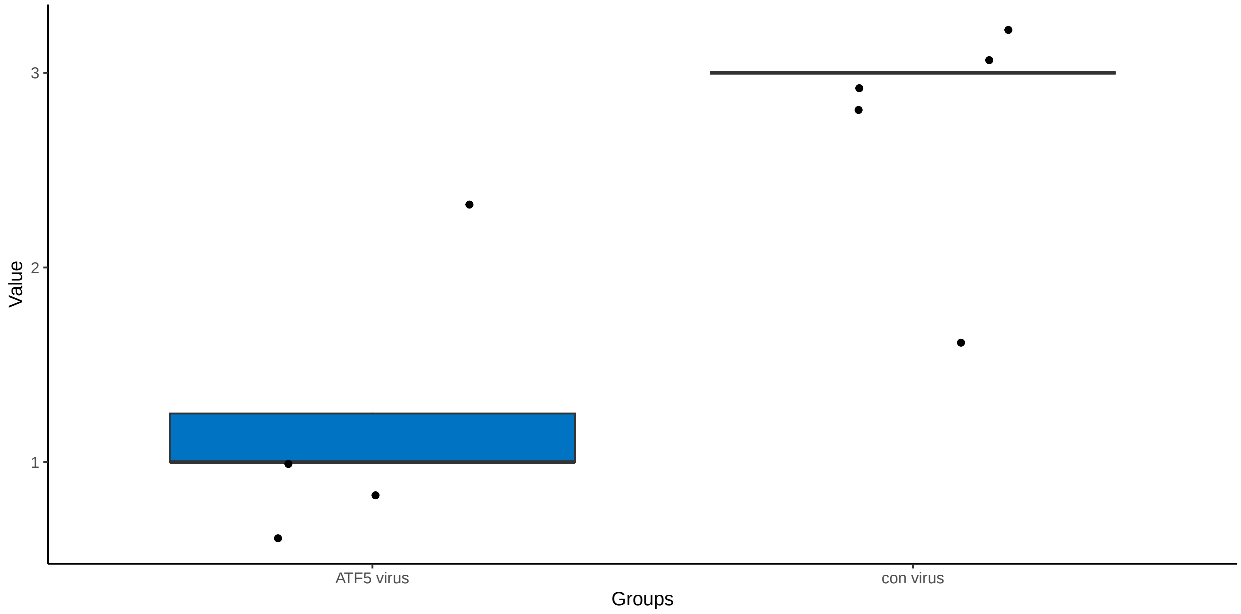


```
## 1. Normality assumption test by Shapiro_Wilk test is
##  p = 0.346
##  Normality assumption was not rejected
## 2. Equal variance test by Bartlett test is
##  p = 0.846
##  Equal variance assumption was not rejected
## 3. The result of anova is
##  p = 0.002
##  A statistically significant difference exist between groups
##
##   Tukey multiple comparisons of means
##     95% family-wise confidence level
##
## Fit: aov(formula = d1[, 3] ~ d1[, 2], data = d1)
##
```

```
## $`d1[, 2]`
##                        diff       lwr      upr     p adj
## con virus-ATF5 virus 1.55 0.8035766 2.296423 0.0017327
```

# Data analysis using R

```
## Present data is ** Fig6 A_2,8 mon_ATF5.csv **
##
## **  Data structure  **
## 'data.frame':    10 obs. of  3 variables:
##  $ subject: int  1 2 3 4 5 6 7 8 9 10
##  $ group  : chr  "2M" "2M" "2M" "2M" ...
##  $ ATF5   : num  0.913 1.148 1.117 1.089 0.733 ...
##
## ** Explorative data analysis with graphics**
```

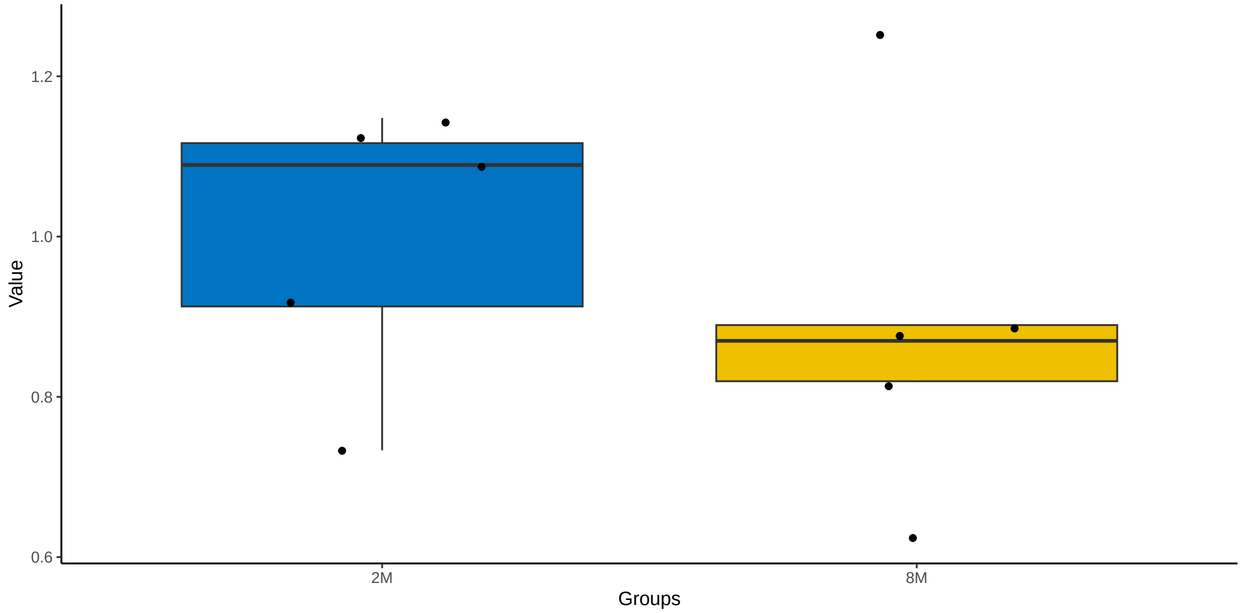


```
## 1. Normality assumption test by Shapiro_Wilk test is
##  p = 0.722
##  Normality assumption was not rejected
## 2. Equal variance test by Bartlett test is
##  p = 0.609
##  Equal variance assumption was not rejected
## 3. The result of anova is
##  p = 0.429
##  A statistically significant difference do not exist between groups
##
```

# Data analysis using R

```
## Present data is ** Fig6 A_2,14 mon_ATF5.csv **
##
## **  Data structure  **
## 'data.frame':    10 obs. of  3 variables:
##  $ subject: int  1 2 3 4 5 6 7 8 9 10
##  $ group  : chr  "2M" "2M" "2M" "2M" ...
##  $ ATF5   : num  1.076 1.204 1.023 1.098 0.599 ...
##
## ** Explorative data analysis with graphics**
```

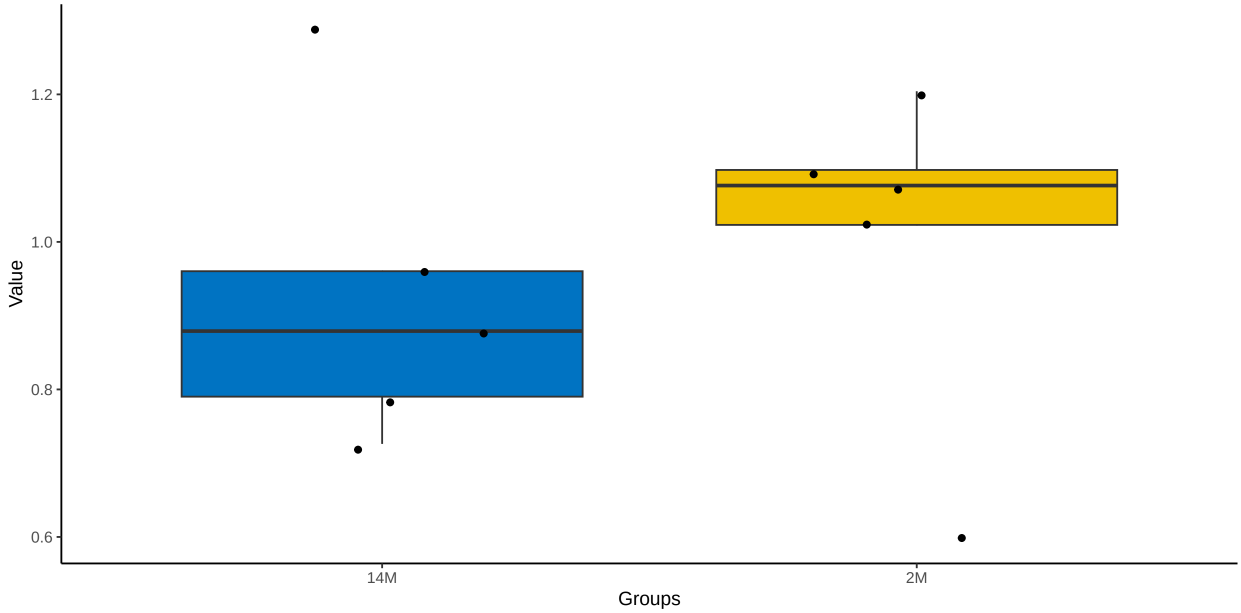


```
## 1. Normality assumption test by Shapiro_Wilk test is
##  p = 0.986
##  Normality assumption was not rejected
## 2. Equal variance test by Bartlett test is
##  p = 0.897
##  Equal variance assumption was not rejected
## 3. The result of anova is
##  p = 0.629
##  A statistically significant difference do not exist between groups
##
```

# Data analysis using R

```
## Present data is ** Fig6 A_2,20 mon_ATF5.csv **
##
## **  Data structure  **
## 'data.frame':    10 obs. of  3 variables:
##  $ subject: int  1 2 3 4 5 6 7 8 9 10
##  $ group  : chr  "2M" "2M" "2M" "2M" ...
##  $ ATF5   : num  0.722 1.324 1.189 1.141 0.625 ...
##
## ** Explorative data analysis with graphics**
```

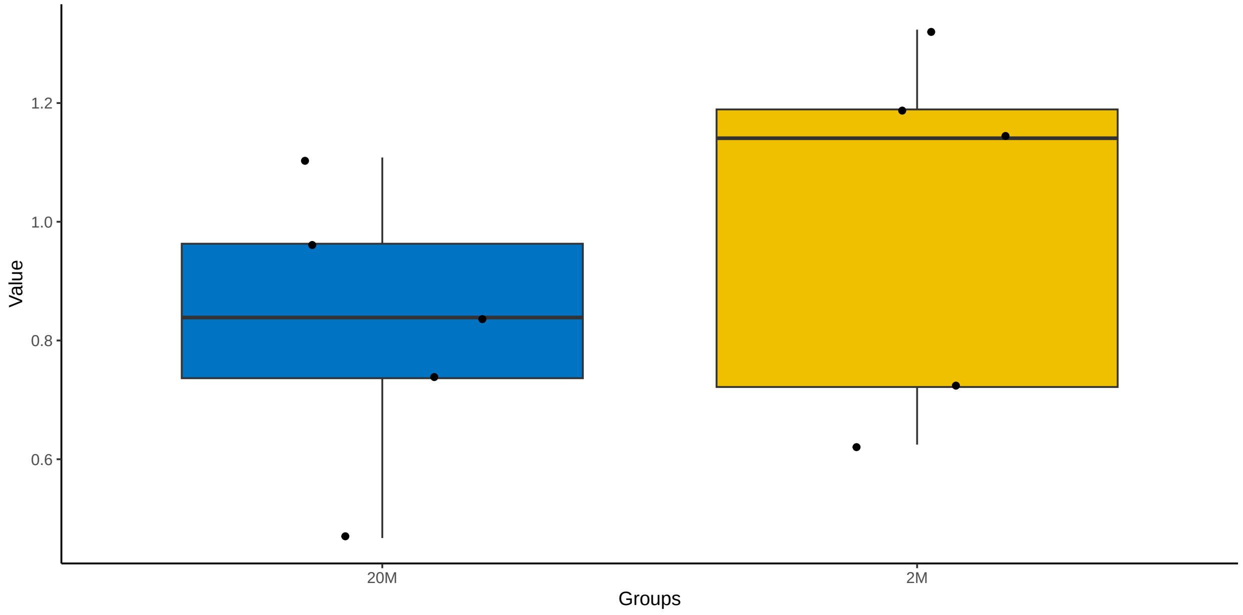


```
## 1. Normality assumption test by Shapiro_Wilk test is
##  p = 0.242
##  Normality assumption was not rejected
## 2. Equal variance test by Bartlett test is
##  p = 0.655
##  Equal variance assumption was not rejected
## 3. The result of anova is
##  p = 0.341
##  A statistically significant difference do not exist between groups
##
```

# Data analysis using R

```
## Present data is ** Fig6 B qPCR_GDF15__2,8,14,20mon.csv *
* ##
## **  Data structure  **
## 'data.frame':    15 obs. of  3 variables:
##  $ subject         : int  1 2 3 4 5 6 7 8 9 10 ...
##  $ group           : chr  "2mon" "2mon" "2mon" "8mon" ...
##  $ GDF15.beta.actin: num  0.945 1.197 0.858 0.888 1.675 ...
##
## ** Explorative data analysis with graphics**
```

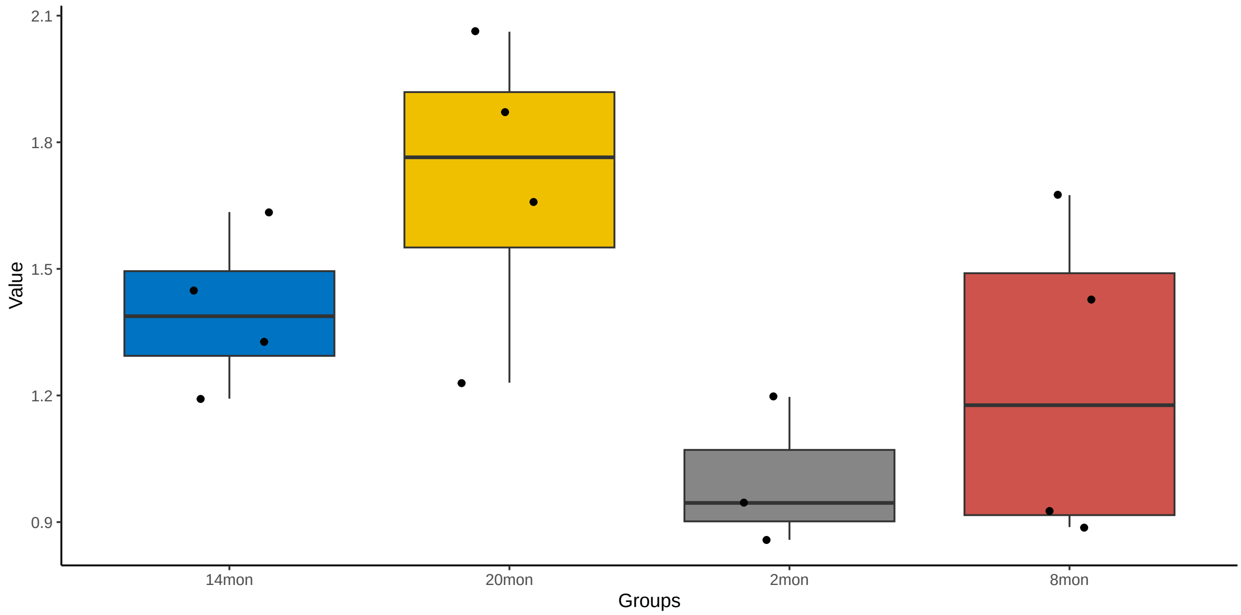


```
## 1. Normality assumption test by Shapiro_Wilk test is
##  p = 0.942
##  Normality assumption was not rejected
## 2. Equal variance test by Bartlett test is
##  p = 0.545
##  Equal variance assumption was not rejected
## 3. The result of anova is
##  p = 0.055
##  A statistically significant difference do not exist between groups
##
```

# Data analysis using R

```
## Present data is ** Fig6 C_2,8,14,20_OCR_Basal.csv **
##
## **  Data structure  **
## 'data.frame':    20 obs. of  20 variables:
##  $ subject: int  1 2 3 4 5 6 7 8 9 10 ...
##  $ group  : chr  "2mon" "2mon" "2mon" "2mon" ...
##  $ basal  : num  119 121 134 191 212 ...
##  $ X      : logi  NA NA NA NA NA NA ...
##  $ X.1    : logi  NA NA NA NA NA NA ...
##  $ X.2    : logi  NA NA NA NA NA NA ...
##  $ X.3    : logi  NA NA NA NA NA NA ...
##  $ X.4    : logi  NA NA NA NA NA NA ...
##  $ X.5    : logi  NA NA NA NA NA NA ...
##  $ X.6    : logi  NA NA NA NA NA NA ...
##  $ X.7    : logi  NA NA NA NA NA NA ...
##  $ X.8    : logi  NA NA NA NA NA NA ...
##  $ X.9    : logi  NA NA NA NA NA NA ...
##  $ X.10   : logi  NA NA NA NA NA NA ...
##  $ X.11   : logi  NA NA NA NA NA NA ...
##  $ X.12   : logi  NA NA NA NA NA NA ...
##  $ X.13   : logi  NA NA NA NA NA NA ...
##  $ X.14   : logi  NA NA NA NA NA NA ...
##  $ X.15   : logi  NA NA NA NA NA NA ...
##  $ X.16   : logi  NA NA NA NA NA NA ...
##
## ** Explorative data analysis with graphics**
```

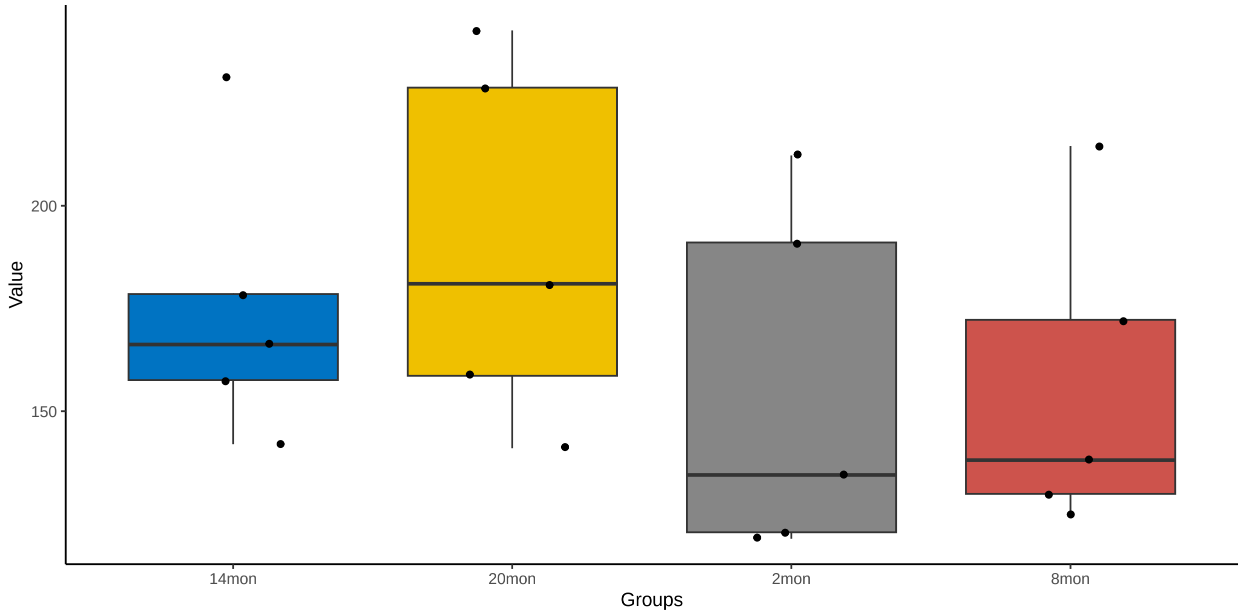

```
## 1. Normality assumption test by Shapiro_Wilk test is
##  p = 0.015
##  Normality assumption was rejected
## 2. The result of Kruskall_Wallis test:
##  p = 0.315
##  A statistically significant difference do not exist between groups
##
```

# Data analysis using R

```
## Present data is ** Fig6 C_2,8,14,20_OCR_state3.csv **
##
## **  Data structure  **
## 'data.frame':    20 obs. of  23 variables:
##  $ subject: int  1 2 3 4 5 6 7 8 9 10 ...
##  $ group  : chr  "2mon" "2mon" "2mon" "2mon" ...
##  $ state3 : num  234 223 252 306 400 ...
##  $ X      : logi  NA NA NA NA NA NA ...
##  $ X.1    : logi  NA NA NA NA NA NA ...
##  $ X.2    : logi  NA NA NA NA NA NA ...
##  $ X.3    : logi  NA NA NA NA NA NA ...
##  $ X.4    : logi  NA NA NA NA NA NA ...
##  $ X.5    : logi  NA NA NA NA NA NA ...
##  $ X.6    : logi  NA NA NA NA NA NA ...
##  $ X.7    : logi  NA NA NA NA NA NA ...
##  $ X.8    : logi  NA NA NA NA NA NA ...
##  $ X.9    : logi  NA NA NA NA NA NA ...
##  $ X.10   : logi  NA NA NA NA NA NA ...
##  $ X.11   : logi  NA NA NA NA NA NA ...
##  $ X.12   : logi  NA NA NA NA NA NA ...
##  $ X.13   : logi  NA NA NA NA NA NA ...
##  $ X.14   : logi  NA NA NA NA NA NA ...
##  $ X.15   : logi  NA NA NA NA NA NA ...
##  $ X.16   : logi  NA NA NA NA NA NA ...
##  $ X.17   : logi  NA NA NA NA NA NA ...
##  $ X.18   : logi  NA NA NA NA NA NA ...
##  $ X.19   : logi  NA NA NA NA NA NA ...
##
## ** Explorative data analysis with graphics**
```

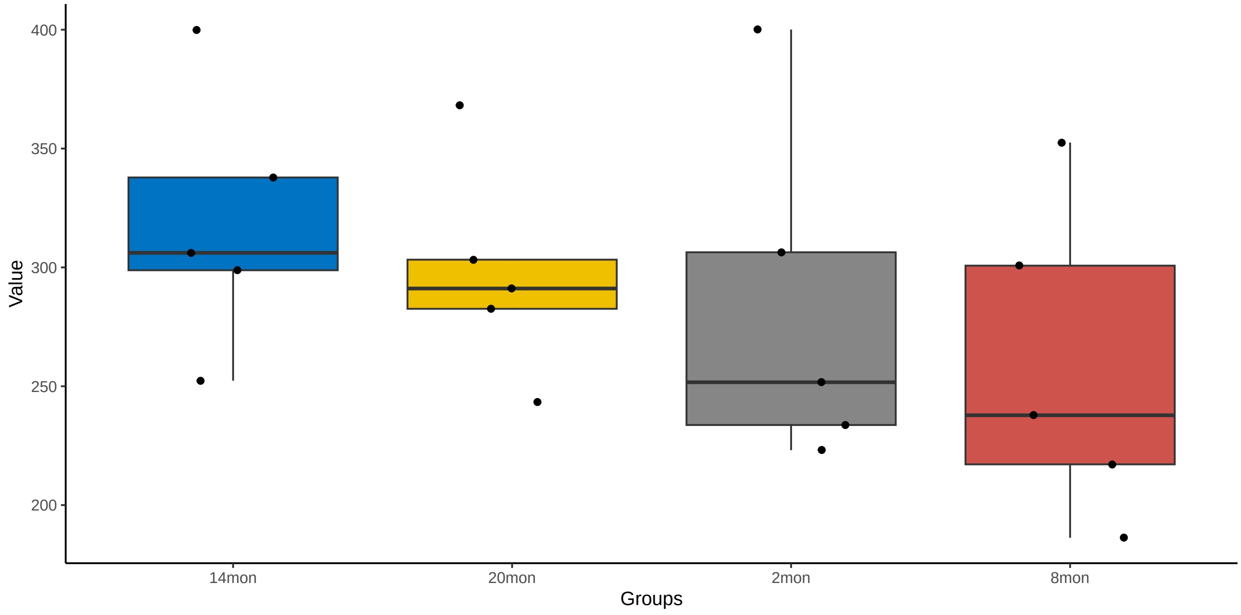


```
## 1. Normality assumption test by Shapiro_Wilk test is
##  p = 0.157
##  Normality assumption was not rejected
## 2. Equal variance test by Bartlett test is
##  p = 0.816
##  Equal variance assumption was not rejected
## 3. The result of anova is
##  p = 0.482
##  A statistically significant difference do not exist between groups
##
```

# Data analysis using R

```
## Present data is ** Fig6 C_2,8,14,20_OCR_state3u.csv **
##
## **  Data structure  **
## 'data.frame':    20 obs. of  26 variables:
##  $ subject: int  1 2 3 4 5 6 7 8 9 10 ...
##  $ group  : chr  "2mon" "2mon" "2mon" "2mon" ...
##  $ state3u: num  153 123 175 238 326 ...
##  $ X      : logi  NA NA NA NA NA NA ...
##  $ X.1    : logi  NA NA NA NA NA NA ...
##  $ X.2    : logi  NA NA NA NA NA NA ...
##  $ X.3    : logi  NA NA NA NA NA NA ...
##  $ X.4    : logi  NA NA NA NA NA NA ...
##  $ X.5    : logi  NA NA NA NA NA NA ...
##  $ X.6    : logi  NA NA NA NA NA NA ...
##  $ X.7    : logi  NA NA NA NA NA NA ...
##  $ X.8    : logi  NA NA NA NA NA NA ...
##  $ X.9    : logi  NA NA NA NA NA NA ...
##  $ X.10   : logi  NA NA NA NA NA NA ...
##  $ X.11   : logi  NA NA NA NA NA NA ...
##  $ X.12   : logi  NA NA NA NA NA NA ...
##  $ X.13   : logi  NA NA NA NA NA NA ...
##  $ X.14   : logi  NA NA NA NA NA NA ...
##  $ X.15   : logi  NA NA NA NA NA NA ...
##  $ X.16   : logi  NA NA NA NA NA NA ...
##  $ X.17   : logi  NA NA NA NA NA NA ...
##  $ X.18   : logi  NA NA NA NA NA NA ...
##  $ X.19   : logi  NA NA NA NA NA NA ...
##  $ X.20   : logi  NA NA NA NA NA NA ...
##  $ X.21   : logi  NA NA NA NA NA NA ...
##  $ X.22   : logi  NA NA NA NA NA NA ...
##
## ** Explorative data analysis with graphics**
```

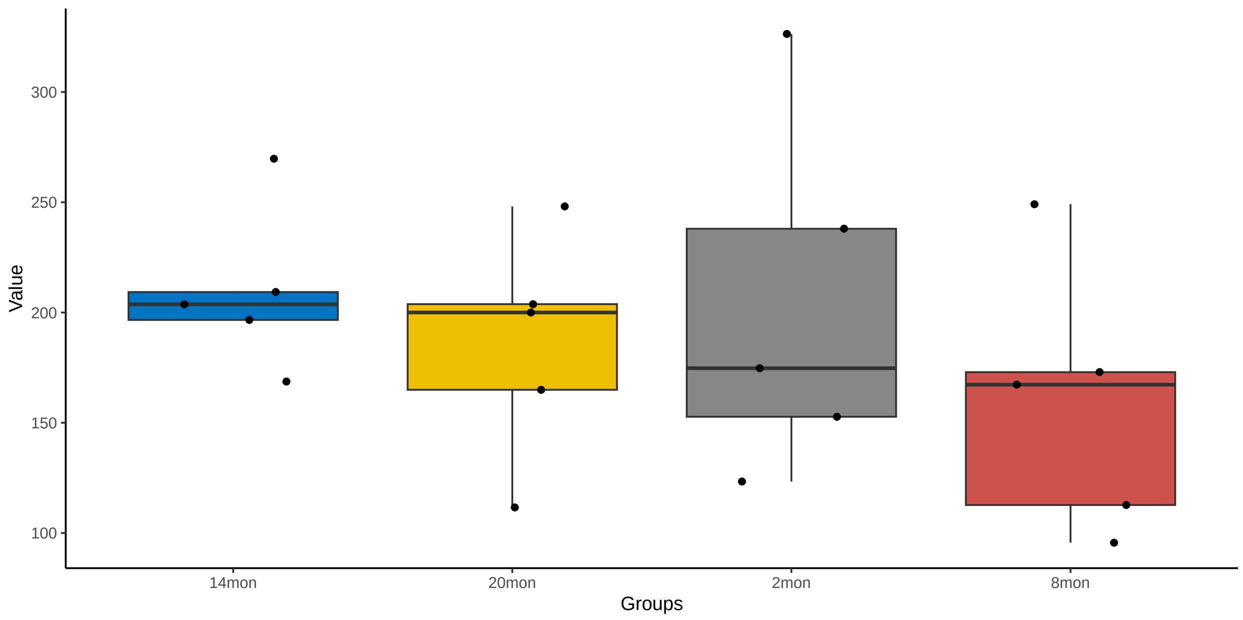


```
## 1. Normality assumption test by Shapiro_Wilk test is
##  p = 0.641
##  Normality assumption was not rejected
## 2. Equal variance test by Bartlett test is
##  p = 0.529
##  Equal variance assumption was not rejected
## 3. The result of anova is
##  p = 0.561
##  A statistically significant difference do not exist between groups
##
```

# Data analysis using R

```
## Present data is ** Fig6 C_2,8,14,20_OCR_state4o.csv **
##
## **  Data structure  **
## 'data.frame':    20 obs. of  26 variables:
##  $ subject: int  1 2 3 4 5 6 7 8 9 10 ...
##  $ group  : chr  "2mon" "2mon" "2mon" "2mon" ...
##  $ state4o: num  66.8 55 71.2 80.2 101.7 ...
##  $ X      : logi  NA NA NA NA NA NA ...
##  $ X.1    : logi  NA NA NA NA NA NA ...
##  $ X.2    : logi  NA NA NA NA NA NA ...
##  $ X.3    : logi  NA NA NA NA NA NA ...
##  $ X.4    : logi  NA NA NA NA NA NA ...
##  $ X.5    : logi  NA NA NA NA NA NA ...
##  $ X.6    : logi  NA NA NA NA NA NA ...
##  $ X.7    : logi  NA NA NA NA NA NA ...
##  $ X.8    : logi  NA NA NA NA NA NA ...
##  $ X.9    : logi  NA NA NA NA NA NA ...
##  $ X.10   : logi  NA NA NA NA NA NA ...
##  $ X.11   : logi  NA NA NA NA NA NA ...
##  $ X.12   : logi  NA NA NA NA NA NA ...
##  $ X.13   : logi  NA NA NA NA NA NA ...
##  $ X.14   : logi  NA NA NA NA NA NA ...
##  $ X.15   : logi  NA NA NA NA NA NA ...
##  $ X.16   : logi  NA NA NA NA NA NA ...
##  $ X.17   : logi  NA NA NA NA NA NA ...
##  $ X.18   : logi  NA NA NA NA NA NA ...
##  $ X.19   : logi  NA NA NA NA NA NA ...
##  $ X.20   : logi  NA NA NA NA NA NA ...
##  $ X.21   : logi  NA NA NA NA NA NA ...
##  $ X.22   : logi  NA NA NA NA NA NA ...
##
## ** Explorative data analysis with graphics**
```

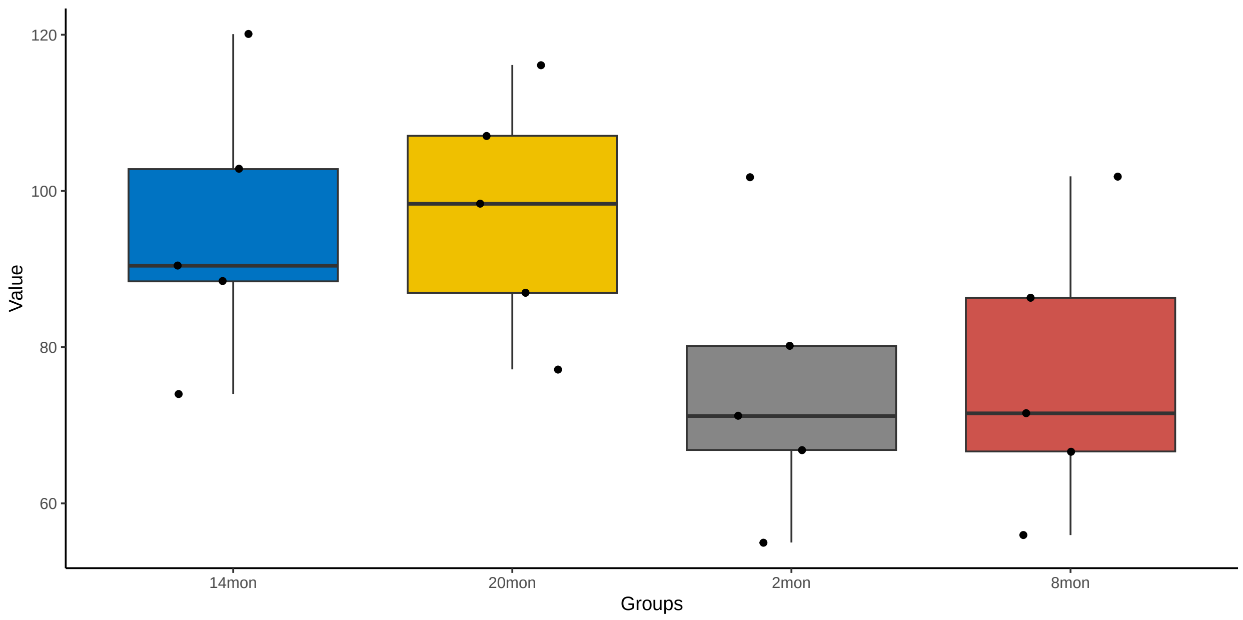


```
## 1. Normality assumption test by Shapiro_Wilk test is
##  p = 0.14
##  Normality assumption was not rejected
## 2. Equal variance test by Bartlett test is
##  p = 0.994
##  Equal variance assumption was not rejected
## 3. The result of anova is
##  p = 0.106
##  A statistically significant difference do not exist between groups
##
```

# Data analysis using R

```
## Present data is ** Fig6E aging_ATF5.csv **
##
## **  Data structure  **
## 'data.frame':    10 obs. of  3 variables:
##  $ subject: int  1 2 3 4 5 6 7 8 9 10
##  $ group  : chr  "con" "con" "con" "con" ...
##  $ ATF5   : num  0.767 0.995 0.981 1.219 1.039 ...
##
## ** Explorative data analysis with graphics**
```

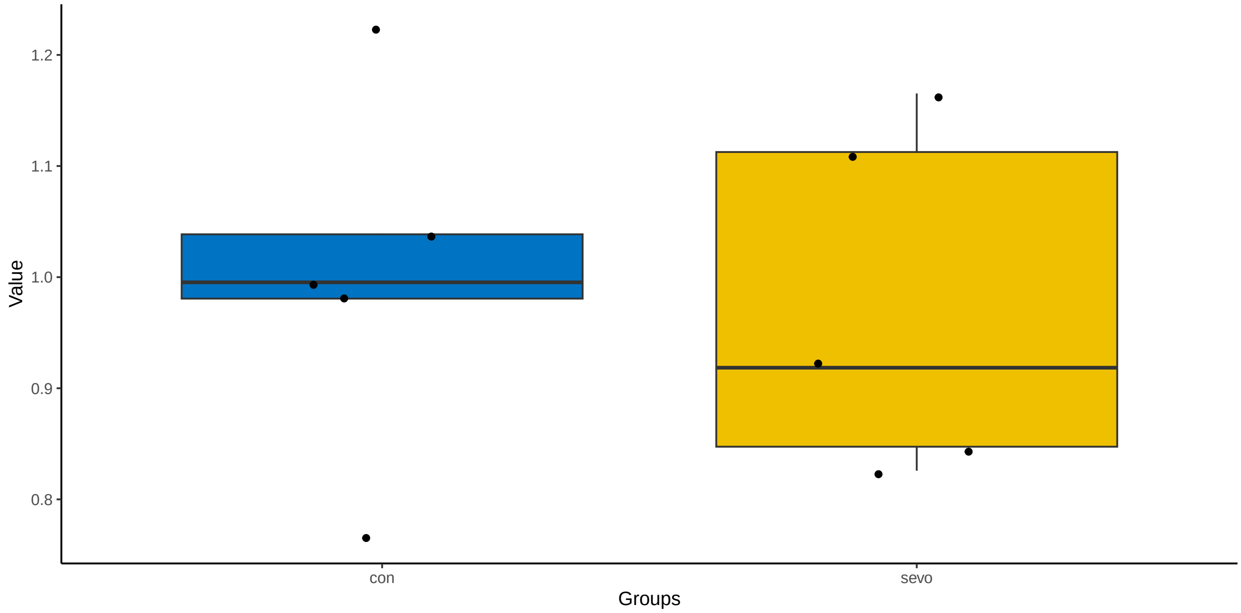


```
## 1. Normality assumption test by Shapiro_Wilk test is
##  p = 0.818
##  Normality assumption was not rejected
## 2. Equal variance test by Bartlett test is
##  p = 0.947
##  Equal variance assumption was not rejected
## 3. The result of anova is
##  p = 0.801
##  A statistically significant difference do not exist between groups
##
```

# Data analysis using R

```
## Present data is ** Fig6E aging_CLPP.csv **
##
## **  Data structure  **
## 'data.frame':    10 obs. of  3 variables:
##  $ subject: int  1 2 3 4 5 6 7 8 9 10
##  $ group  : chr  "con" "con" "con" "con" ...
##  $ CLPP   : num  1.141 0.928 0.997 0.898 1.035 ...
##
## ** Explorative data analysis with graphics**
```

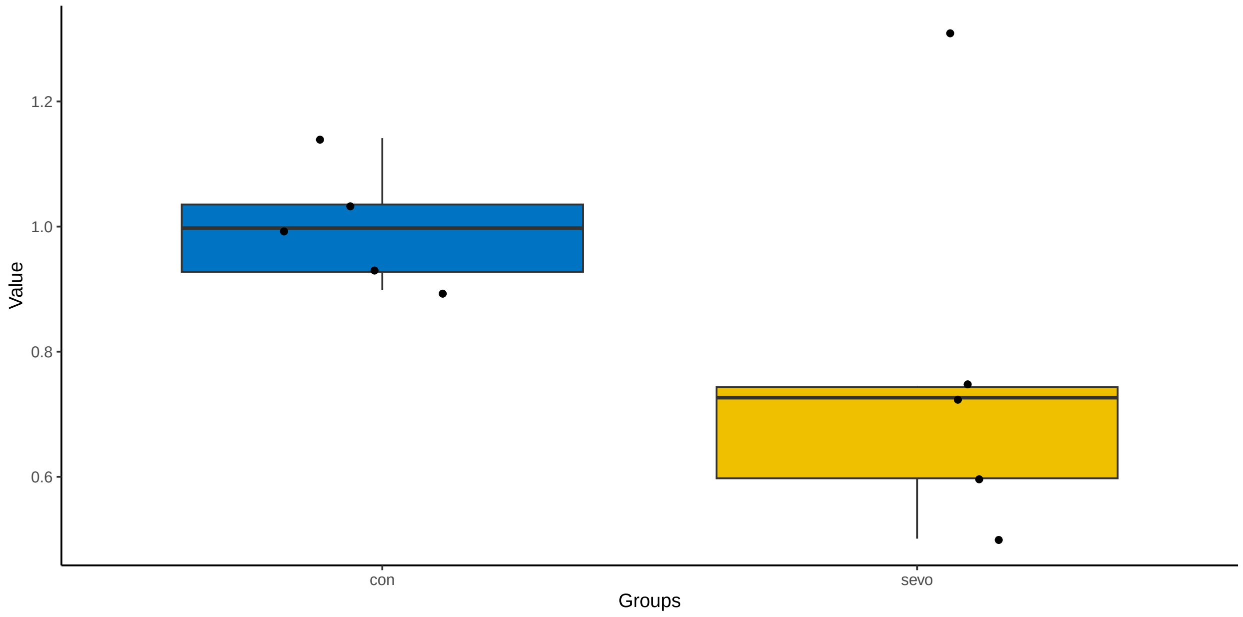


```
## 1. Normality assumption test by Shapiro_Wilk test is
##  p = 0.062
##  Normality assumption was not rejected
## 2. Equal variance test by Bartlett test is
##  p = 0.041
##  Equal variance assumption was rejected
## 3. The result of Welch ANOVA is
##  p = 0.193
##  A statistically significant difference do not exist between groups
##
```

# Data analysis using R

```
## Present data is ** Fig6E aging_HSP60.csv **
##
## **  Data structure  **
## 'data.frame':    10 obs. of  3 variables:
##  $ subject: int  1 2 3 4 5 6 7 8 9 10
##  $ group  : chr  "con" "con" "con" "con" ...
##  $ HSP60  : num  0.493 0.907 0.987 1.295 1.317 ...
##
## ** Explorative data analysis with graphics**
```

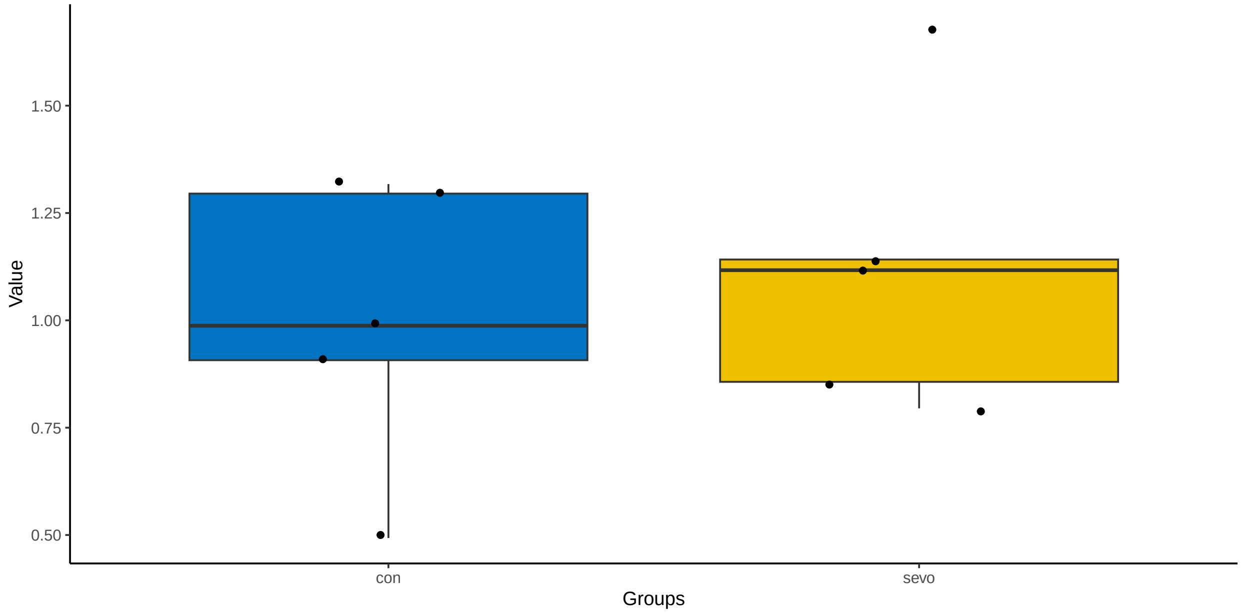


```
## 1. Normality assumption test by Shapiro_Wilk test is
##  p = 0.926
##  Normality assumption was not rejected
## 2. Equal variance test by Bartlett test is
##  p = 0.957
##  Equal variance assumption was not rejected
## 3. The result of anova is
##  p = 0.605
##  A statistically significant difference do not exist between groups
##
```

# Data analysis using R

```
## Present data is ** Fig6E aging_HSP70.csv **
##
## **  Data structure  **
## 'data.frame':    10 obs. of  3 variables:
##  $ subject: int  1 2 3 4 5 6 7 8 9 10
##  $ group  : chr  "con" "con" "con" "con" ...
##  $ HSP70  : num  0.891 1.206 0.943 1.052 0.909 ...
##
## ** Explorative data analysis with graphics**
```

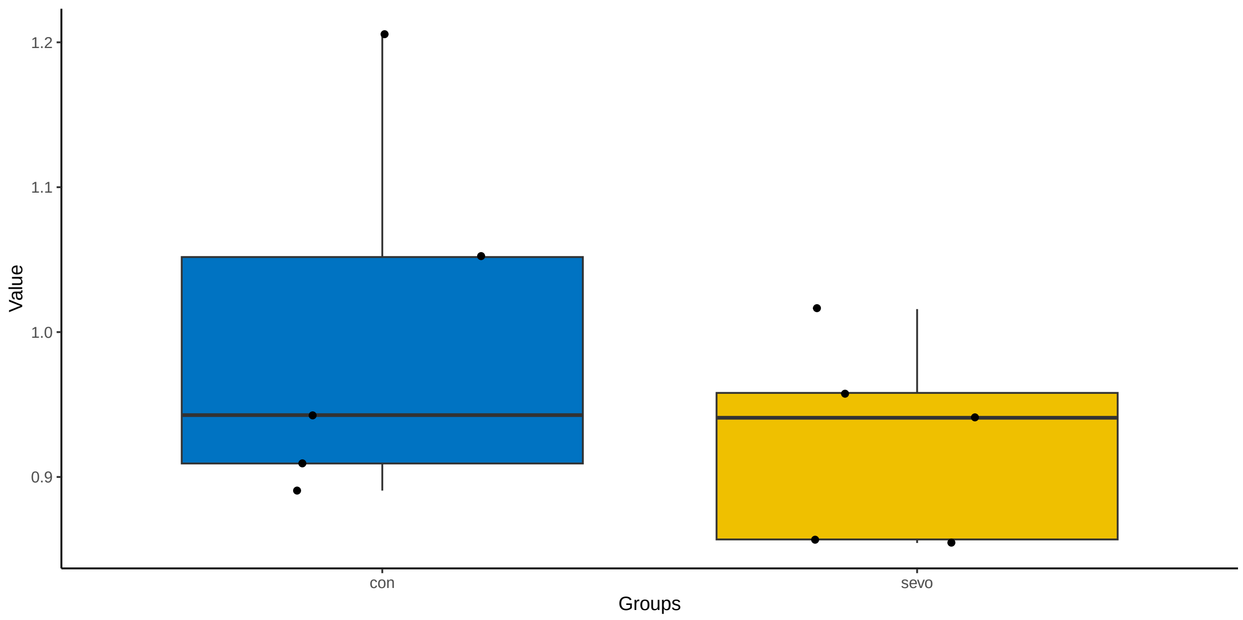


```
## 1. Normality assumption test by Shapiro_Wilk test is
##  p = 0.263
##  Normality assumption was not rejected
## 2. Equal variance test by Bartlett test is
##  p = 0.246
##  Equal variance assumption was not rejected
## 3. The result of anova is
##  p = 0.291
##  A statistically significant difference do not exist between groups
##
```

# Data analysis using R

```
## Present data is ** Fig6E aging_LONP1.csv **
##
## **  Data structure  **
## 'data.frame':    10 obs. of  3 variables:
##  $ subject: int  1 2 3 4 5 6 7 8 9 10
##  $ group  : chr  "con" "con" "con" "con" ...
##  $ LONP1  : num  1.272 1.041 1.12 0.558 1.007 ...
##
## ** Explorative data analysis with graphics**
```

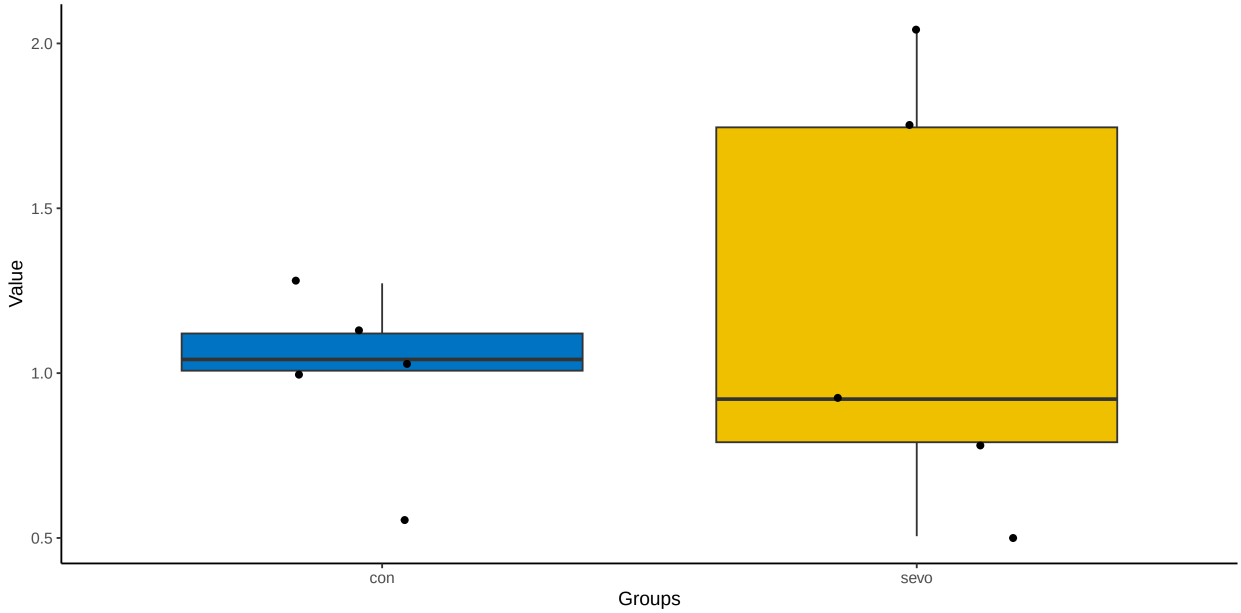


```
## 1. Normality assumption test by Shapiro_Wilk test is
##  p = 0.927
##  Normality assumption was not rejected
## 2. Equal variance test by Bartlett test is
##  p = 0.109
##  Equal variance assumption was not rejected
## 3. The result of anova is
##  p = 0.545
##  A statistically significant difference do not exist between groups
##
```

# Data analysis using R

```
## Present data is ** Fig6 G Aging_OCR_Basal.csv **
##
## **  Data structure  **
## 'data.frame':    9 obs. of  3 variables:
##  $ subject: int  1 2 3 4 5 6 7 8 9
##  $ group  : chr  "Aging" "Aging" "Aging" "Aging" ...
##  $ basal  : num  623 436 501 502 453 ...
##
## ** Explorative data analysis with graphics**
```

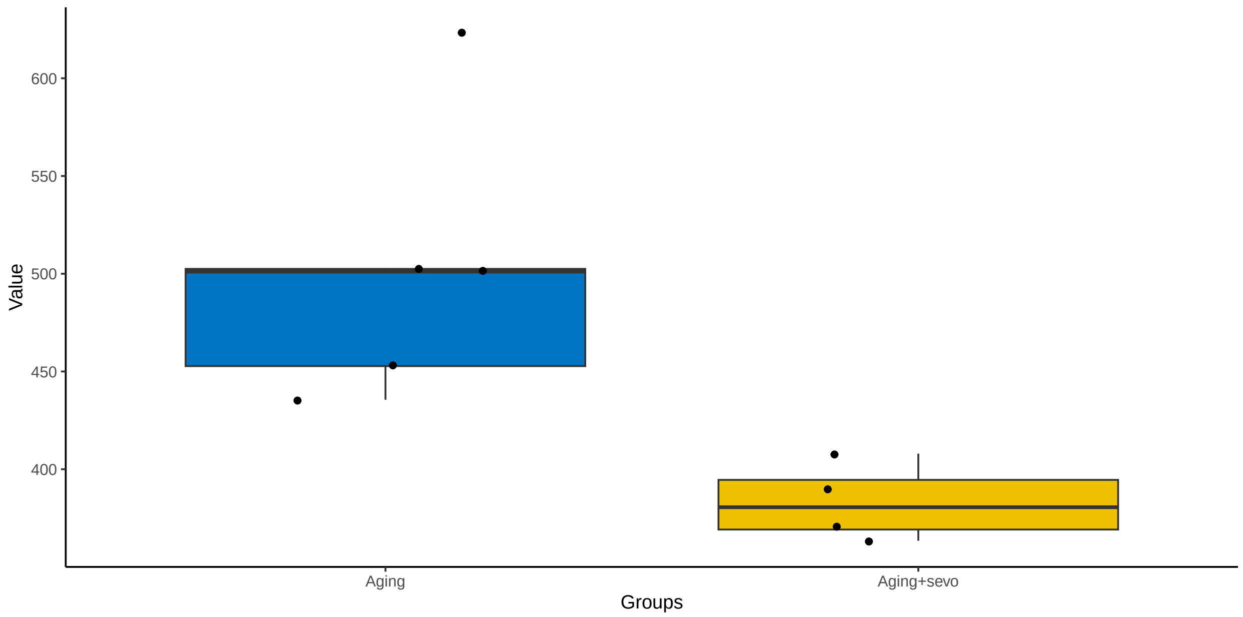


```
## 1. Normality assumption test by Shapiro_Wilk test is
##  p = 0.148
##  Normality assumption was not rejected
## 2. Equal variance test by Bartlett test is
##  p = 0.054
##  Equal variance assumption was not rejected
## 3. The result of anova is
##  p = 0.016
##  A statistically significant difference exist between groups
##
##   Tukey multiple comparisons of means
##     95% family-wise confidence level
##
## Fit: aov(formula = d1[, 3] ~ d1[, 2], data = d1)
##
```

```
## $`d1[, 2]`
##                       diff       lwr       upr    p adj
## Aging+sevo-Aging -119.8672 -210.1712 -29.56325 0.016408
```

# Data analysis using R

```
## Present data is ** Fig6G Aging_OCR_State3.csv **
##
## **  Data structure  **
## 'data.frame':    9 obs. of  3 variables:
##  $ subject: int  1 2 3 4 5 6 7 8 9
##  $ group  : chr  "Aging" "Aging" "Aging" "Aging" ...
##  $ state3 : num  842 662 683 1032 657 ...
##
## ** Explorative data analysis with graphics**
```

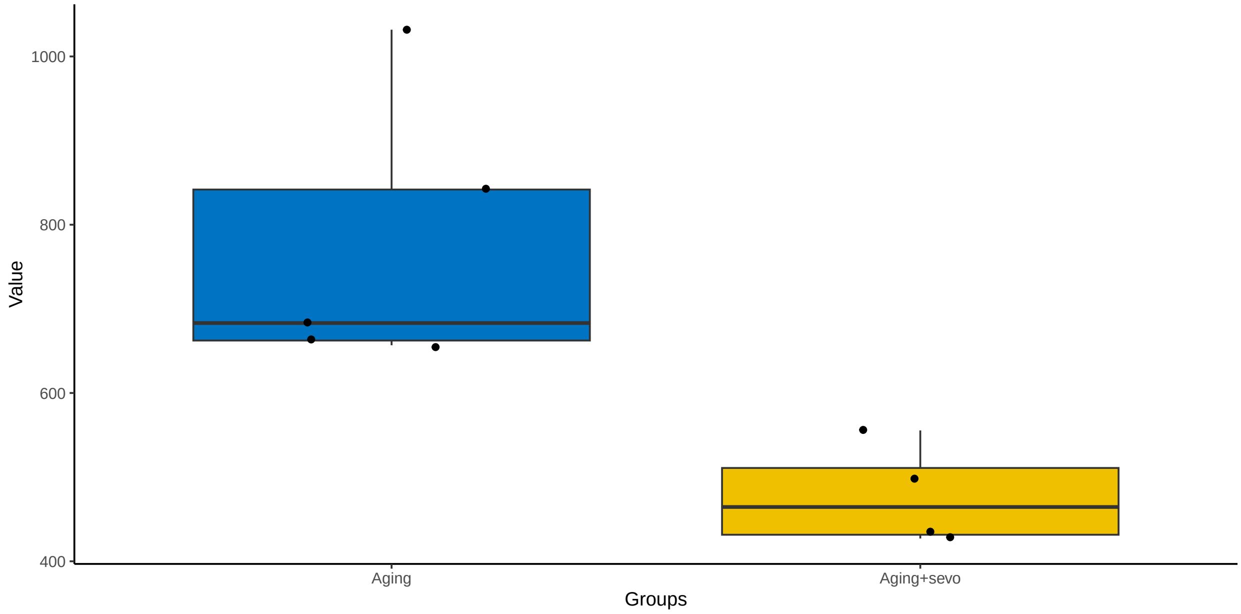


```
## 1. Normality assumption test by Shapiro_Wilk test is
##  p = 0.165
##  Normality assumption was not rejected
## 2. Equal variance test by Bartlett test is
##  p = 0.124
##  Equal variance assumption was not rejected
## 3. The result of anova is
##  p = 0.011
##  A statistically significant difference exist between groups
##
##   Tukey multiple comparisons of means
##     95% family-wise confidence level
##
## Fit: aov(formula = d1[, 3] ~ d1[, 2], data = d1)
##
```

```
## $`d1[, 2]`
##                        diff       lwr       upr     p adj
## Aging+sevo-Aging -297.2767 -501.8583 -92.69511 0.0108958
```

# Data analysis using R

```
## Present data is ** Fig6G Aging_OCR_State3u.csv **
##
## **  Data structure  **
## 'data.frame':    9 obs. of  3 variables:
##  $ subject: int  1 2 3 4 5 6 7 8 9
##  $ group  : chr  "Aging" "Aging" "Aging" "Aging" ...
##  $ state3u: num  649 461 463 860 426 ...
##
## ** Explorative data analysis with graphics**
```

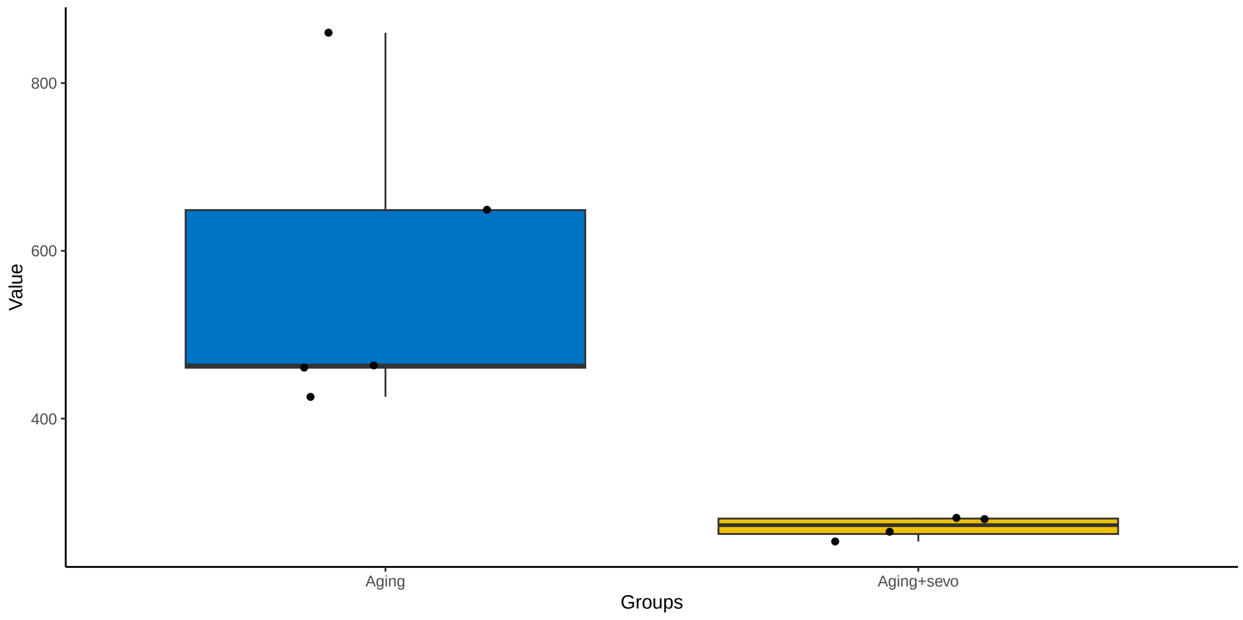


```
## 1. Normality assumption test by Shapiro_Wilk test is
##  p = 0.125
##  Normality assumption was not rejected
## 2. Equal variance test by Bartlett test is
##  p = 0.001
##  Equal variance assumption was rejected
## 3. The result of Welch ANOVA is
##  p = 0.021
##  A statistically significant difference exist between groups
##
##   Tukey multiple comparisons of means
##     95% family-wise confidence level
##
## Fit: aov(formula = d1[, 3] ~ d1[, 2])
##
```

```
## $`d1[, 2]`
##                        diff       lwr       upr     p adj
## Aging+sevo-Aging -301.4471 -521.6027 -81.29142 0.0142975
```

# Data analysis using R

```
## Present data is ** Fig6G Aging_OCR_State4o.csv **
##
## **  Data structure  **
## 'data.frame':    9 obs. of  3 variables:
##  $ subject: int  1 2 3 4 5 6 7 8 9
##  $ group  : chr  "Aging" "Aging" "Aging" "Aging" ...
##  $ state4o: num  245 172 208 275 181 ...
##
## ** Explorative data analysis with graphics**
```

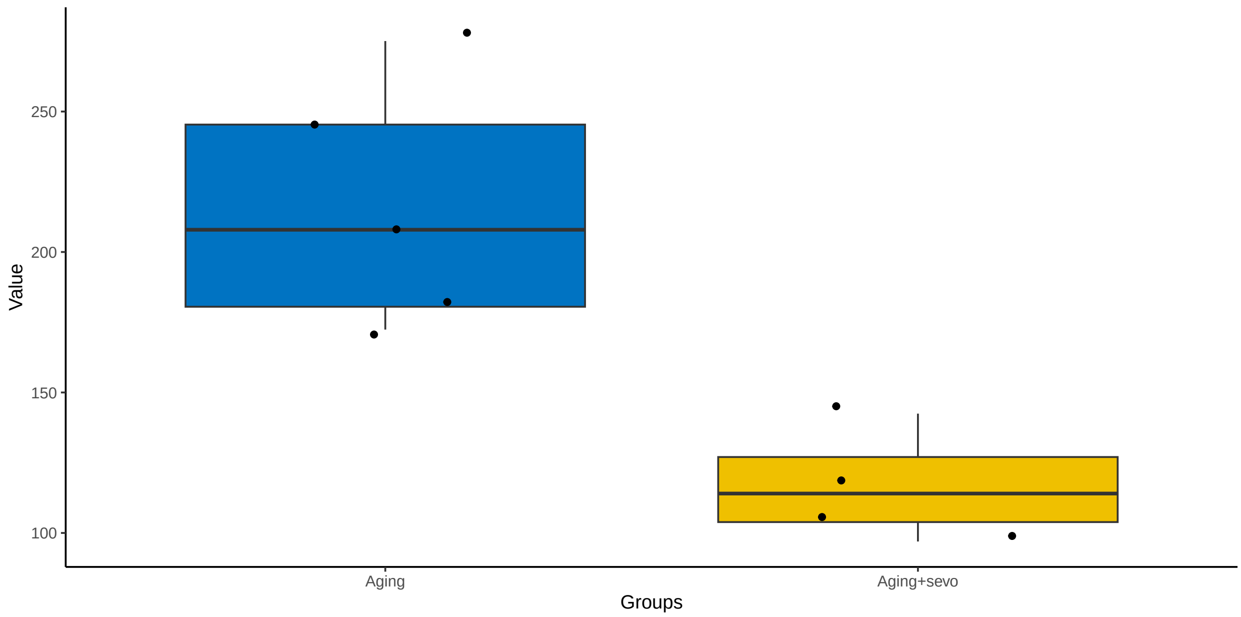


```
## 1. Normality assumption test by Shapiro_Wilk test is
##  p = 0.848
##  Normality assumption was not rejected
## 2. Equal variance test by Bartlett test is
##  p = 0.211
##  Equal variance assumption was not rejected
## 3. The result of anova is
##  p = 0.004
##  A statistically significant difference exist between groups
##
##   Tukey multiple comparisons of means
##     95% family-wise confidence level
##
## Fit: aov(formula = d1[, 3] ~ d1[, 2], data = d1)
##
```

```
## $`d1[, 2]`
##                      diff       lwr      upr    p adj
## Aging+sevo-Aging -99.38237 -155.5291 -43.2356 0.004109
```
