# Supplementary material for: ATF5‐Dependent GDF15 Expression Mediates Anesthesia‐Induced Neuroprotection Against Stroke
Source: Adv Sci (Weinh). 2025 Nov 26;13(3):e17086. doi: 10.1002/advs.202417086 (PMC12806220; doi:10.1002/advs.202417086)

Western Blot -raw data

Figure 1D_B6_WB_raw data 1
Figure 2G ATF5 cKO _WB_raw data 11
Figure 3F_GDF15_WB_raw data 21
Figure 4B_WB_raw data(virus cortex injection) 31
Figure 5B_WB_raw data(virus iv) 41
Figure 6E_aging_WB raw data 43

Figure1 D →LONP1

Control (n=5) Sevoflurane (n=5)

106 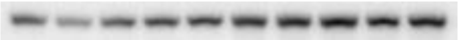 LONP1

Sample bind

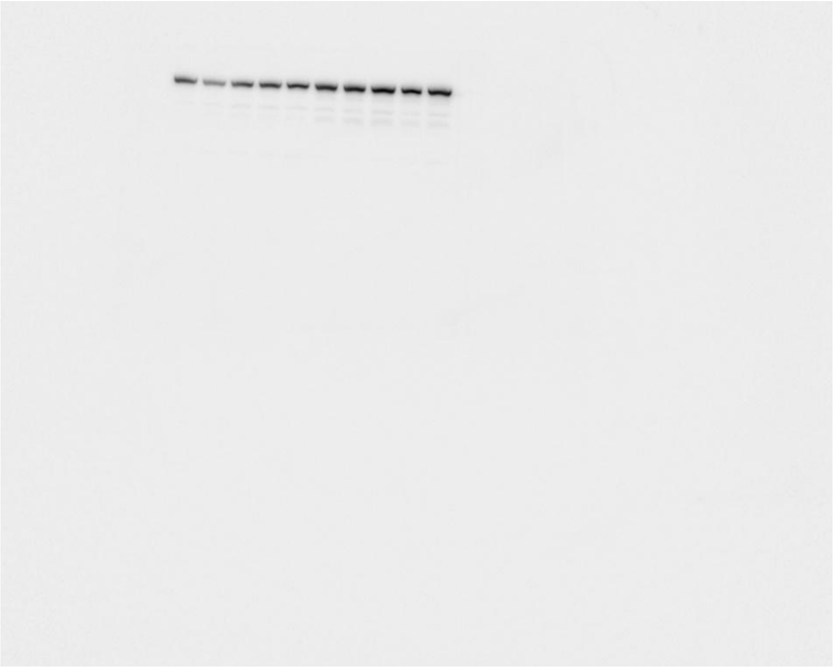

Protein marker

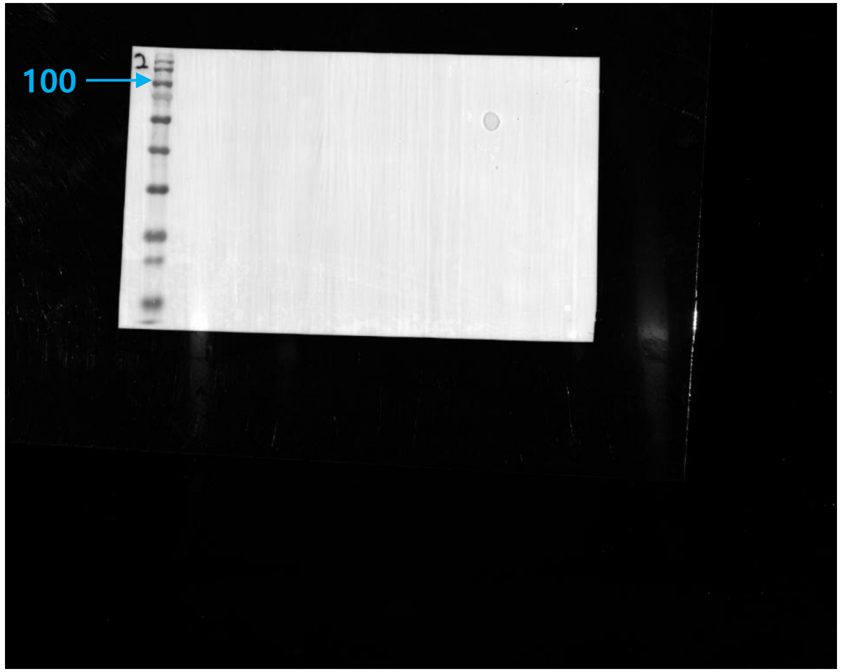

Merge

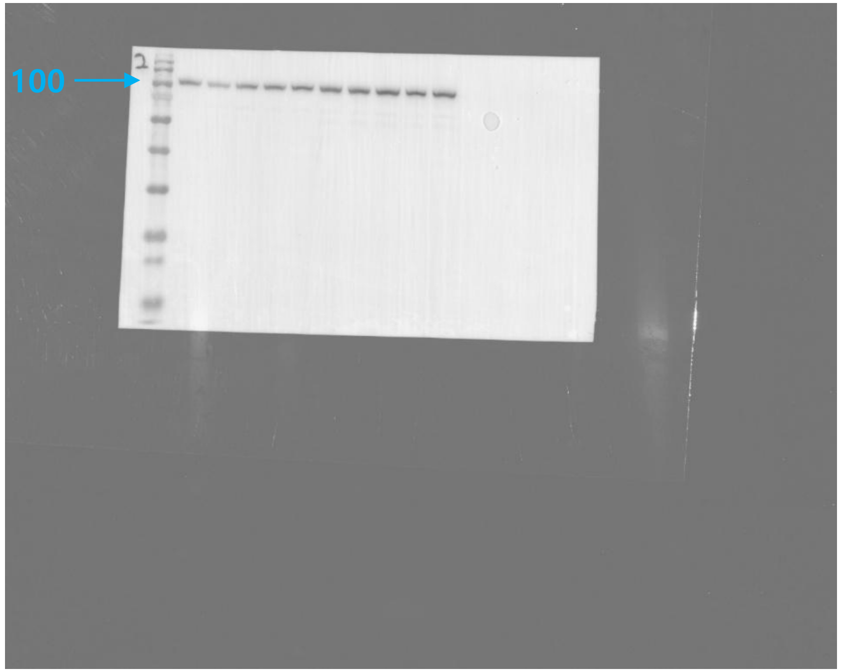

Figure1 D → β-actin of LONP1

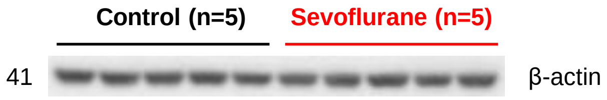

Sample bind

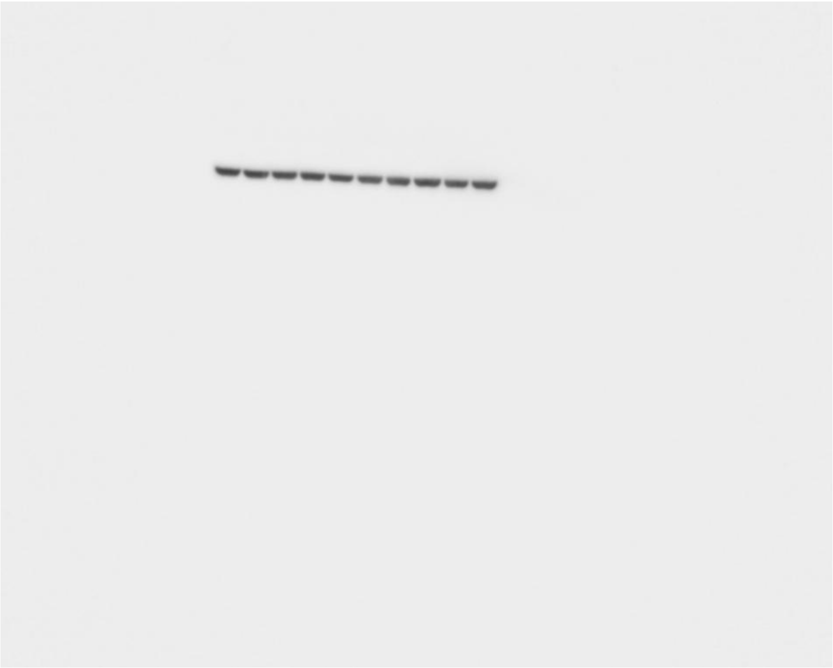

Protein marker

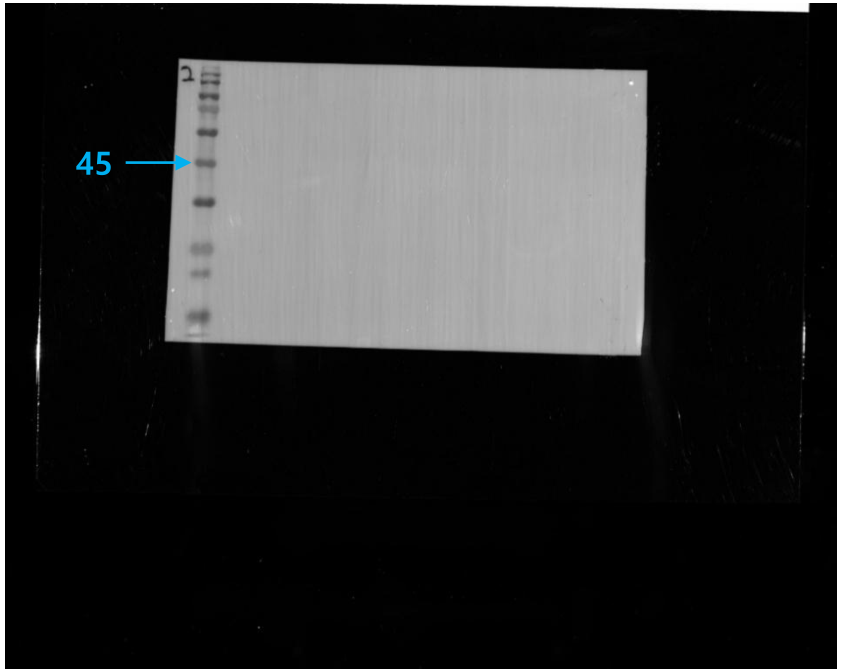

Merge

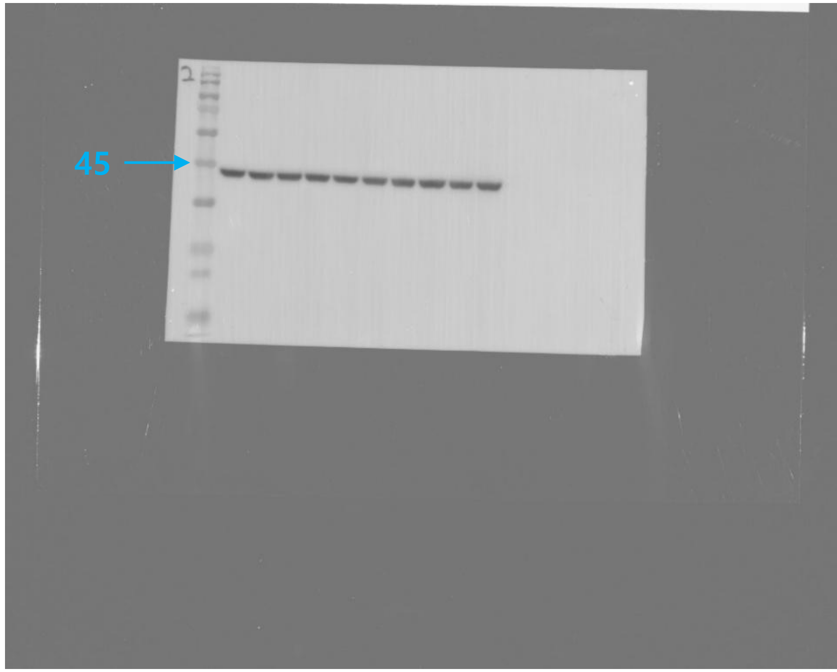

Figure1 D → HSP70

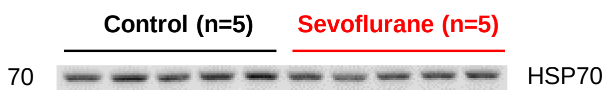

Sample bind

Protein marker

Merge

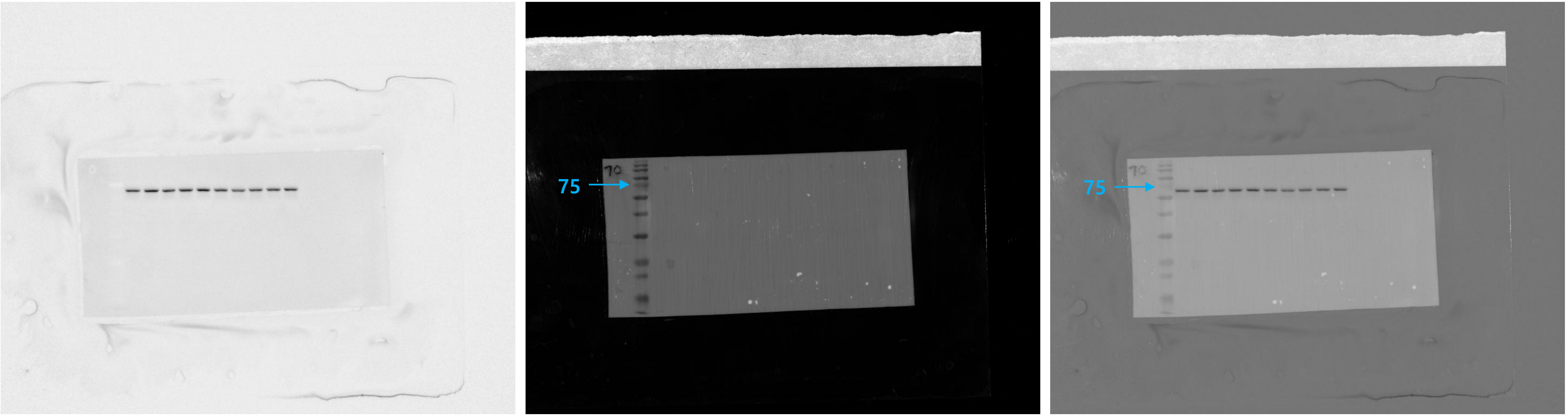

Figure1 D → β-actin of HSP70

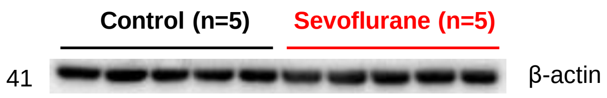

Sample bind

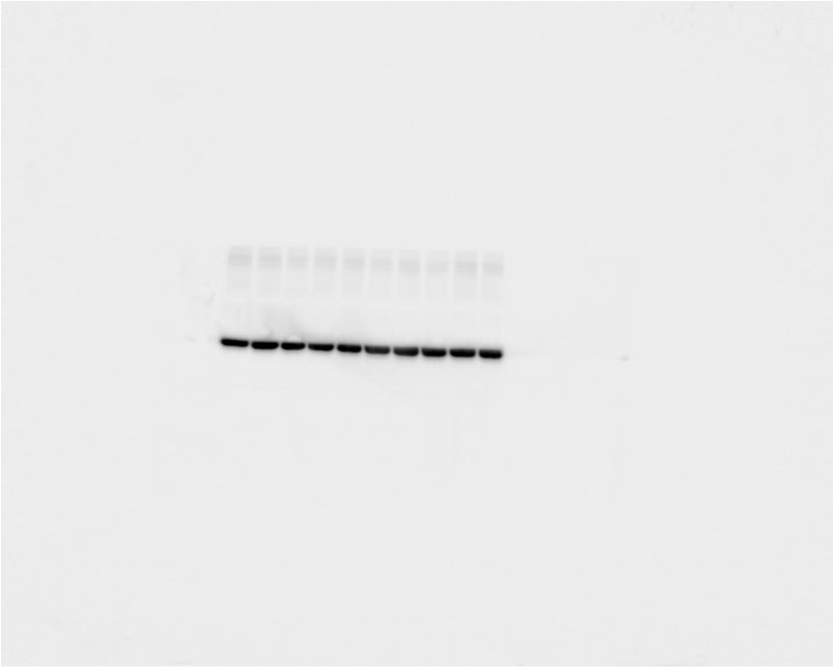

Protein marker

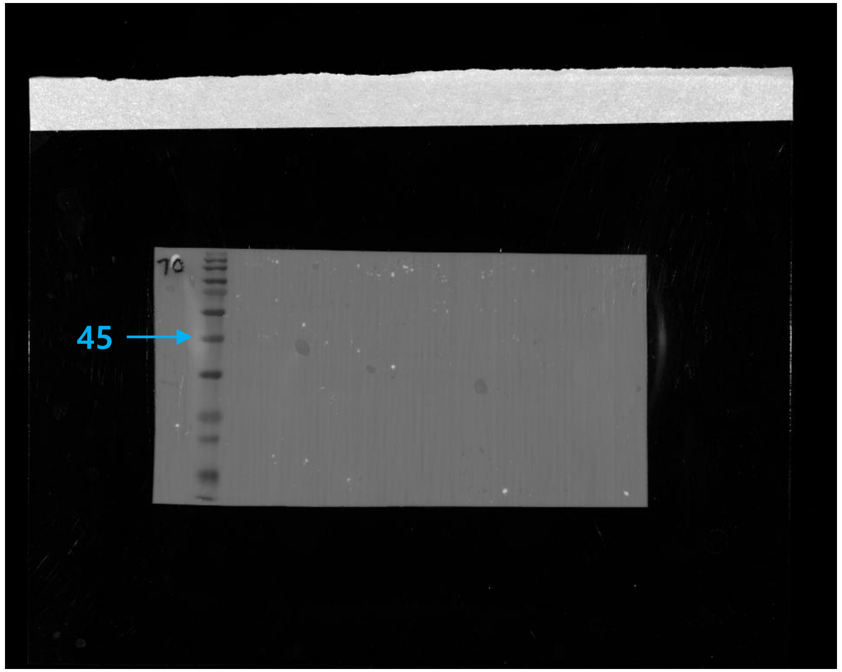

Merge

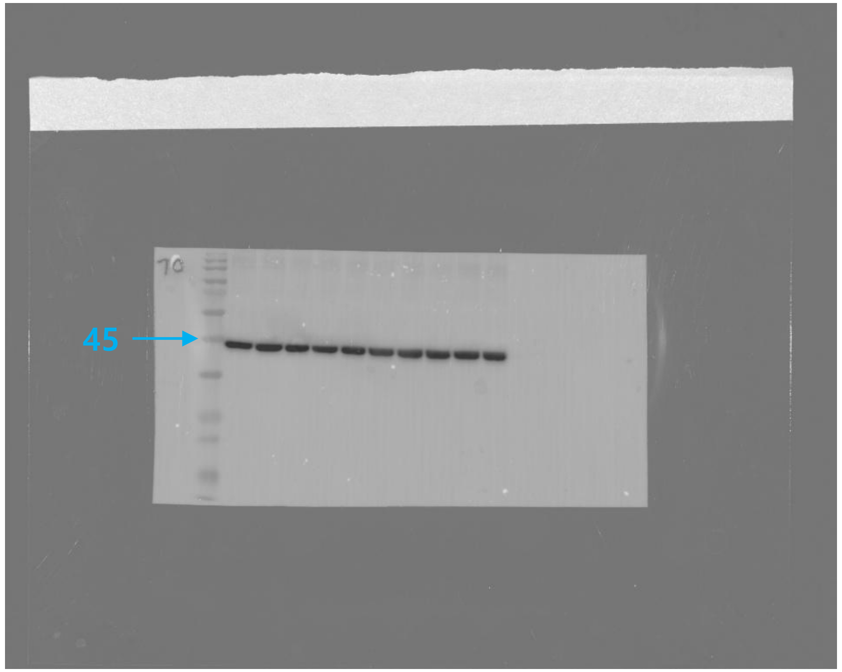

Figure1 D → HSP60

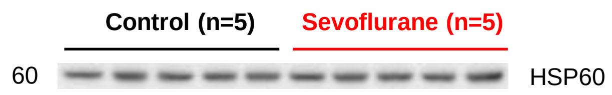

Sample bind

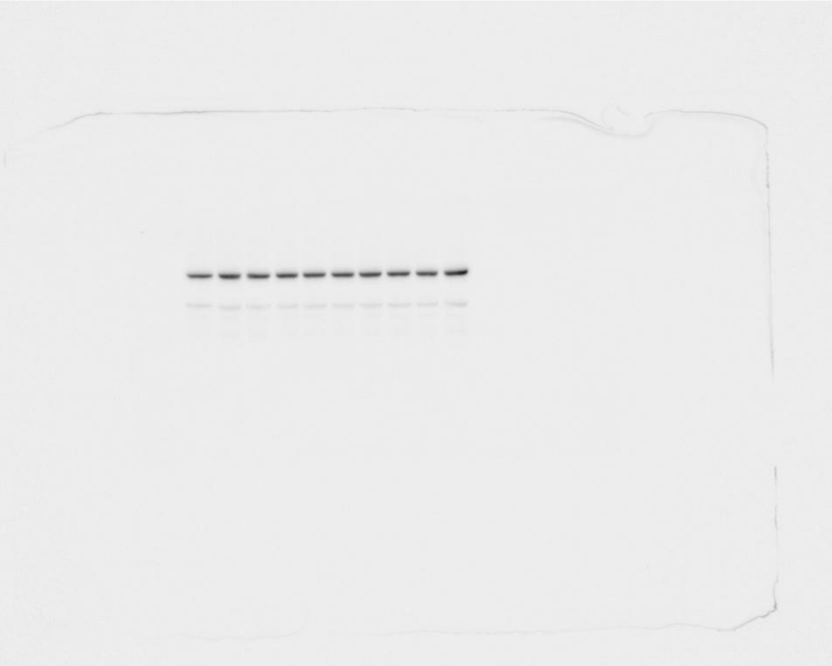

Protein marker

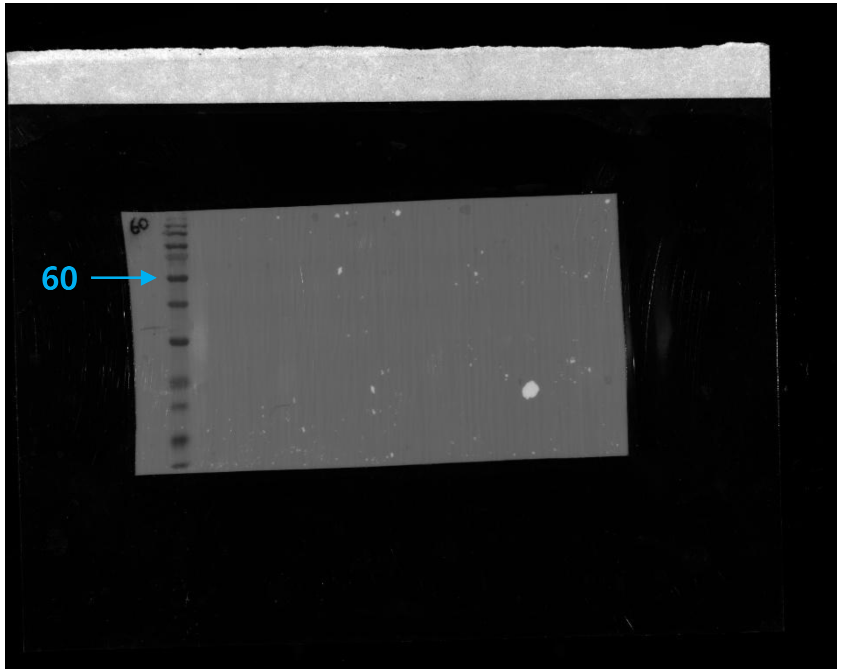

Merge

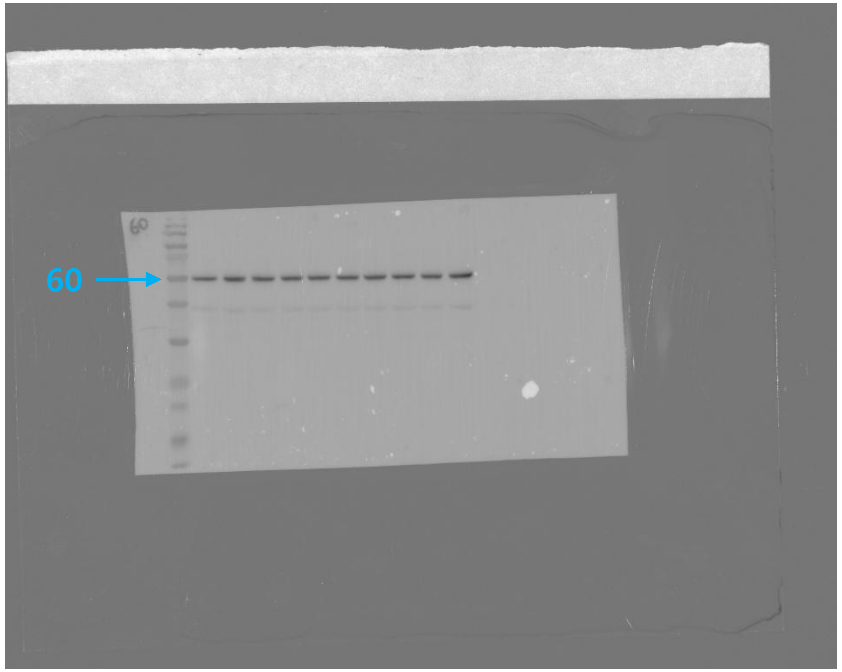

Figure1 D → β-actin of HSP60

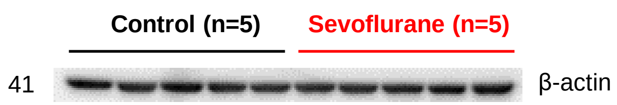

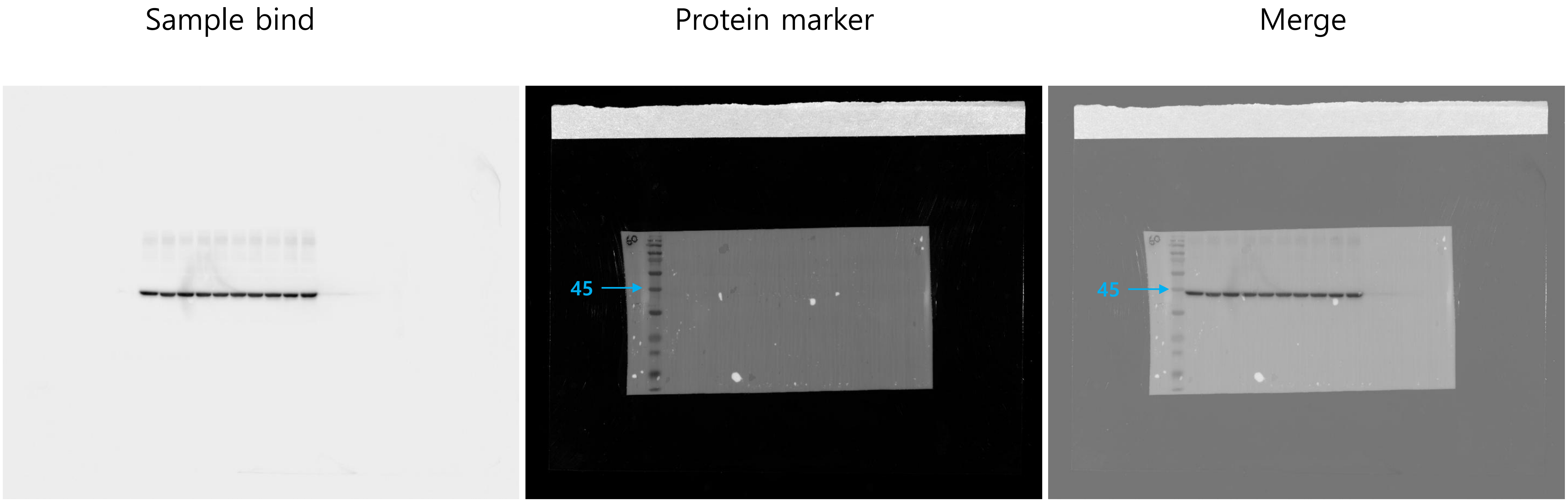

Figure1 D → ATF5

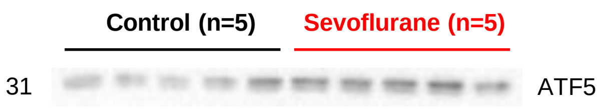

Sample bind

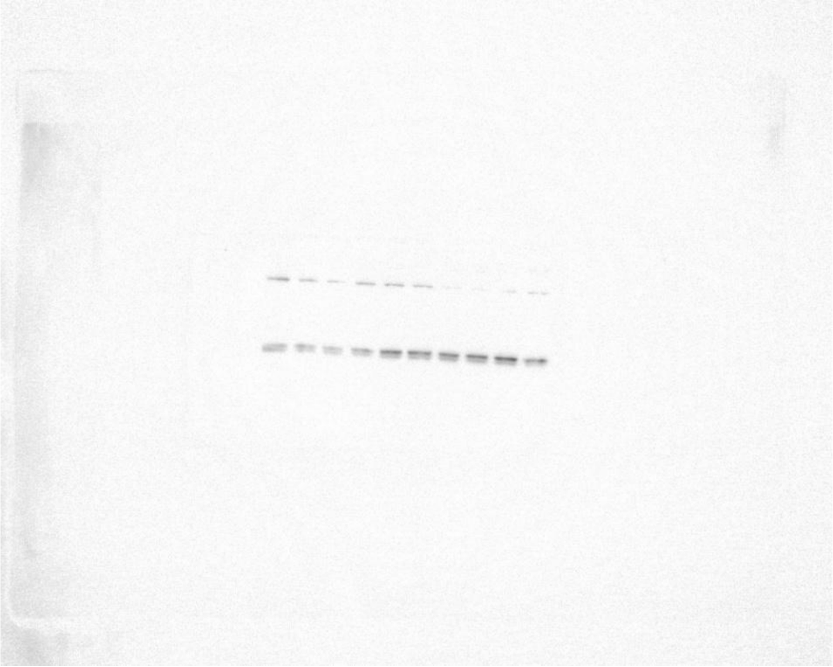

Protein marker

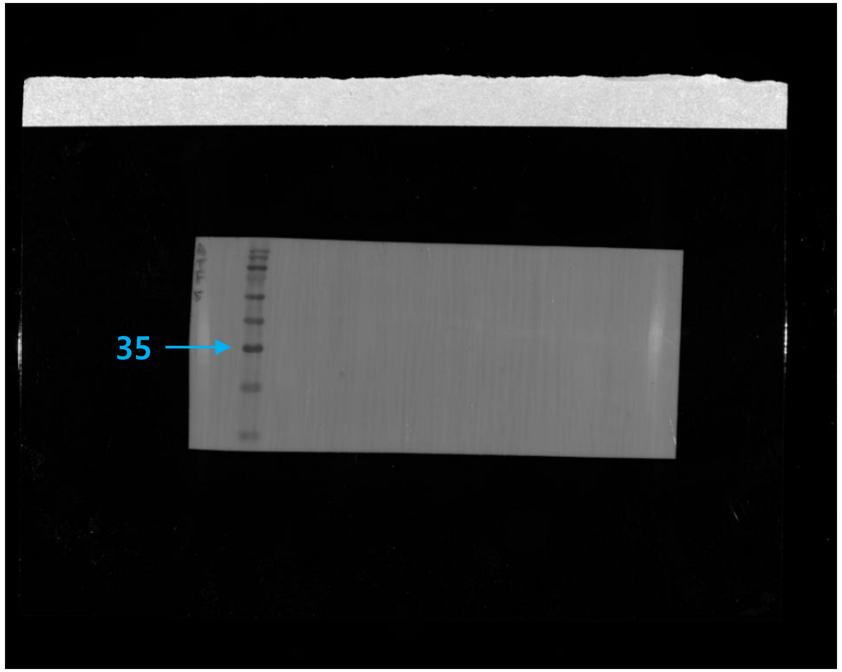

Merge

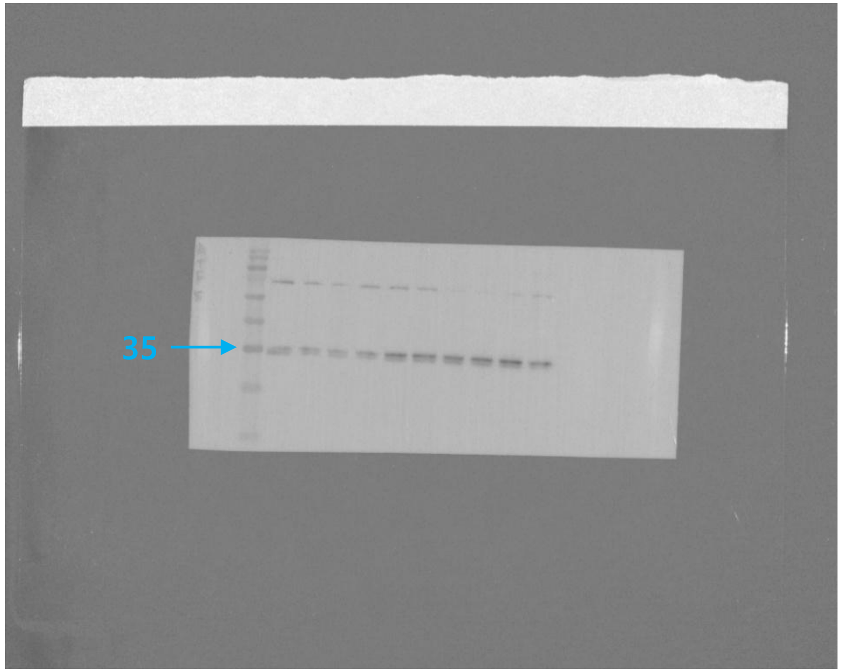

Figure1 D → β-actin of ATF5

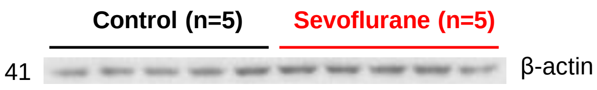

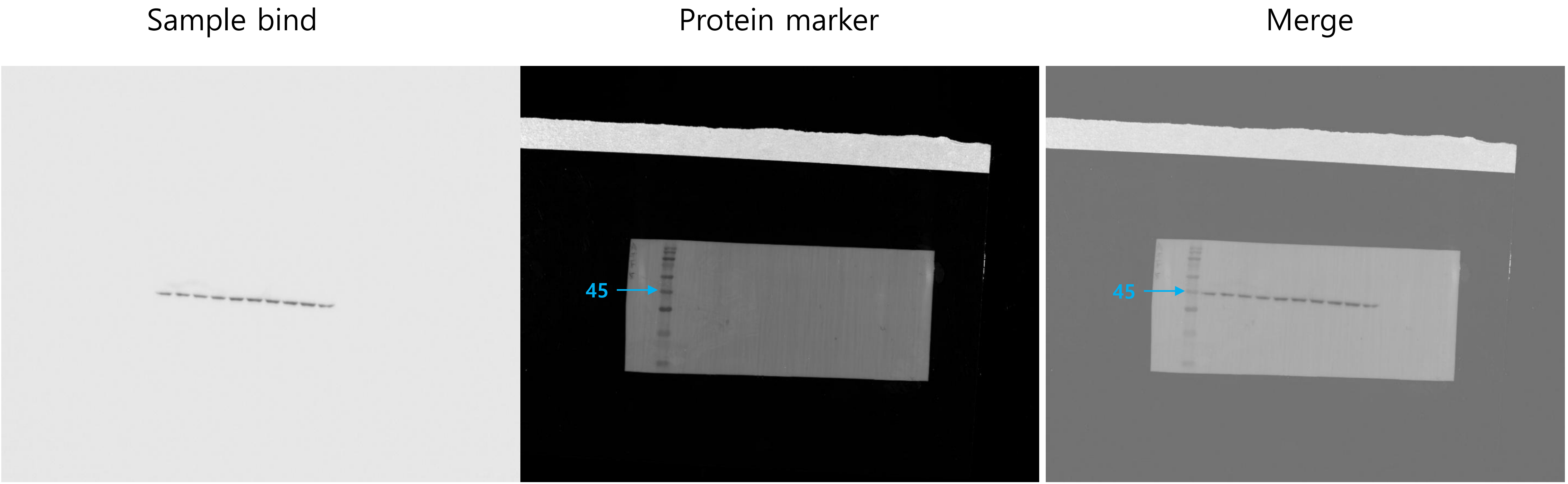

Figure1 D → CLPP

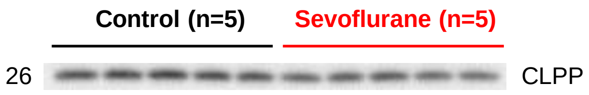

Sample bind

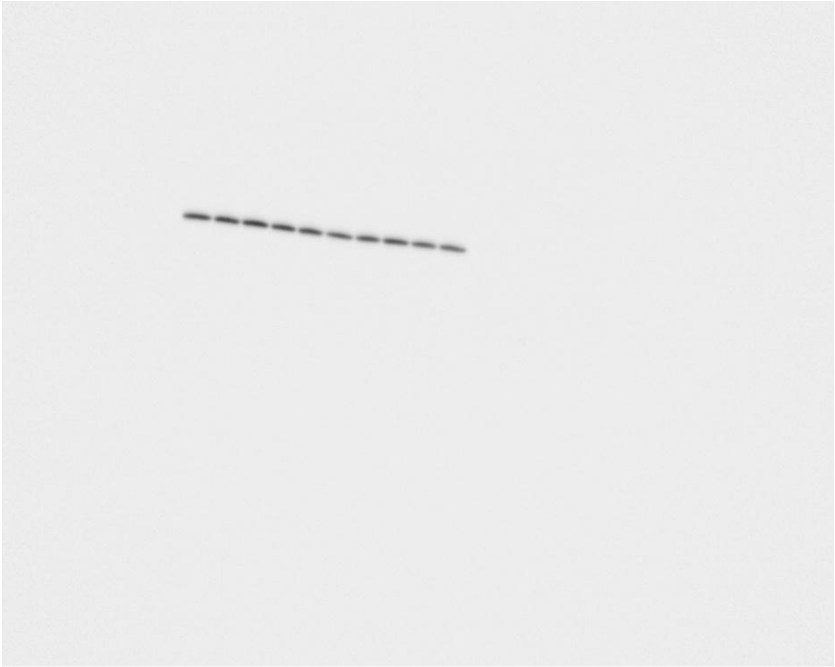

Protein marker

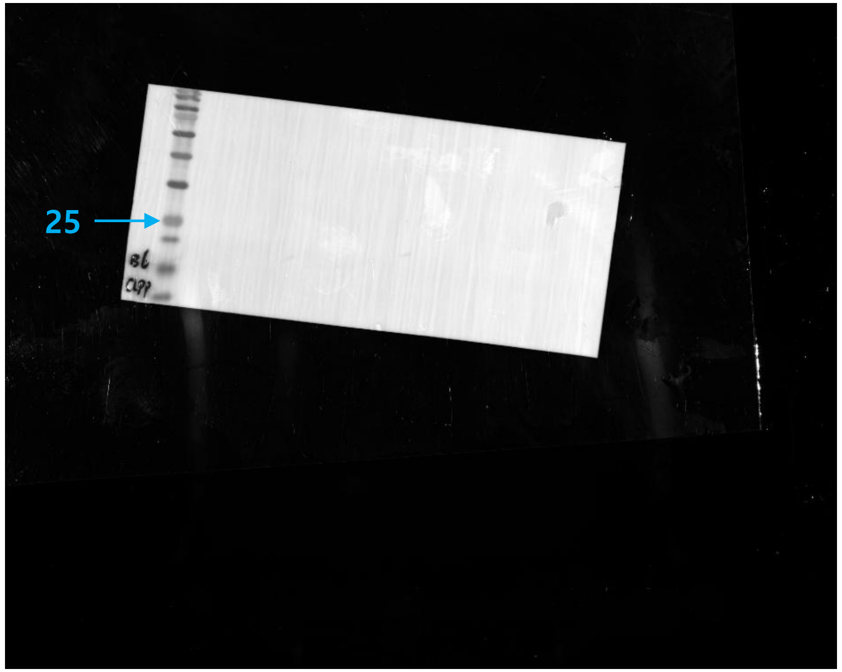

Merge

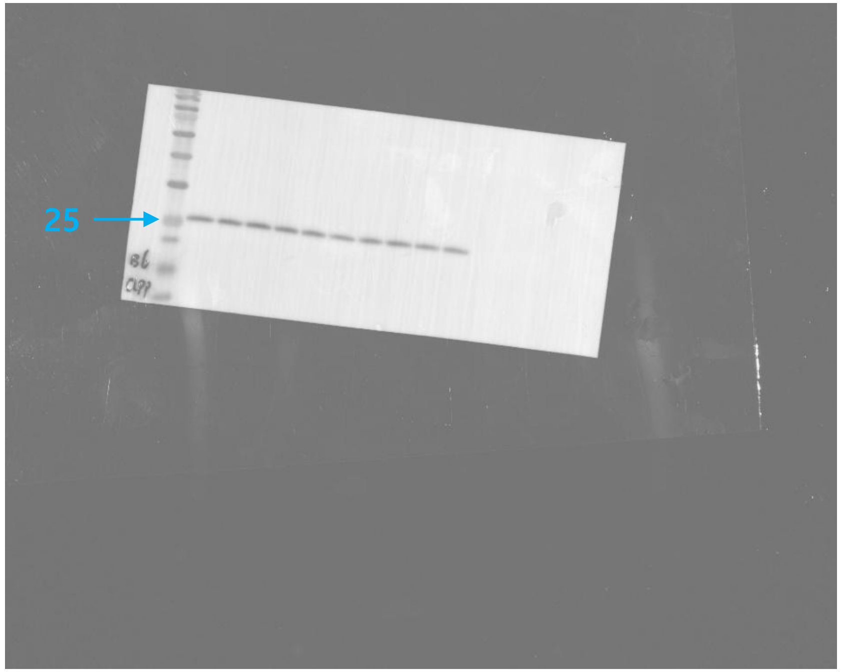

Figure1 D → β-actin of CLPP

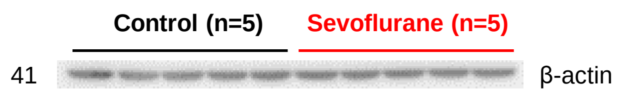

Sample bind | Protein marker | Merge

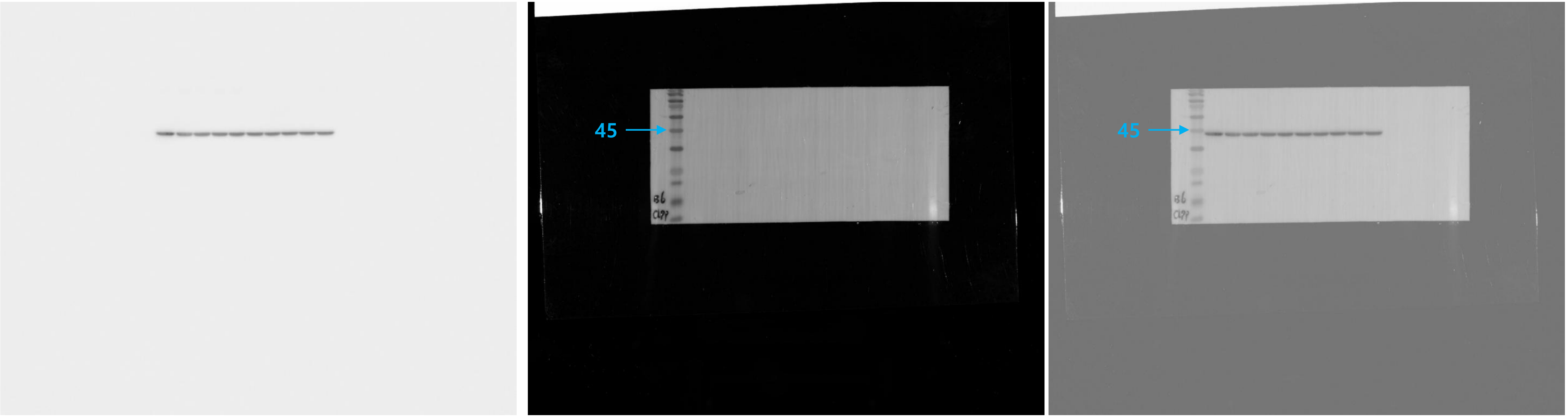

Figure2 G→ LONP1

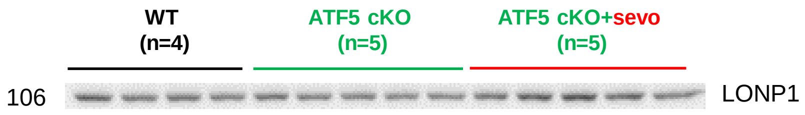

Sample bind Protein marker Merge

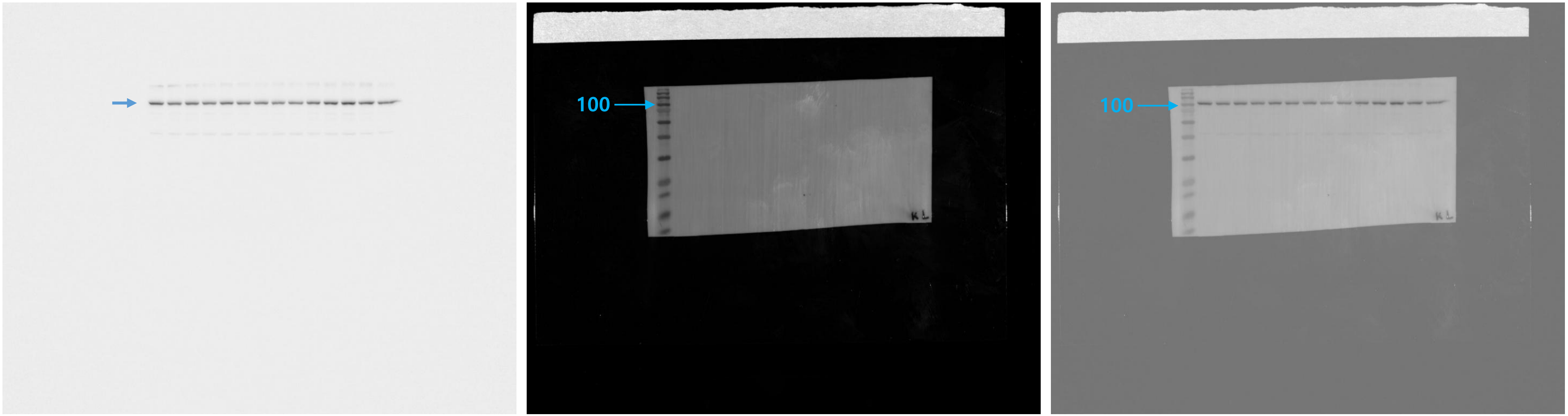

Figure2 G→ β-actin of LONP1

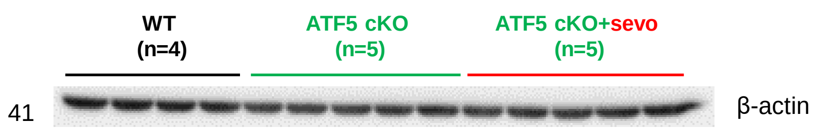

Sample bind

Protein marker

Merge

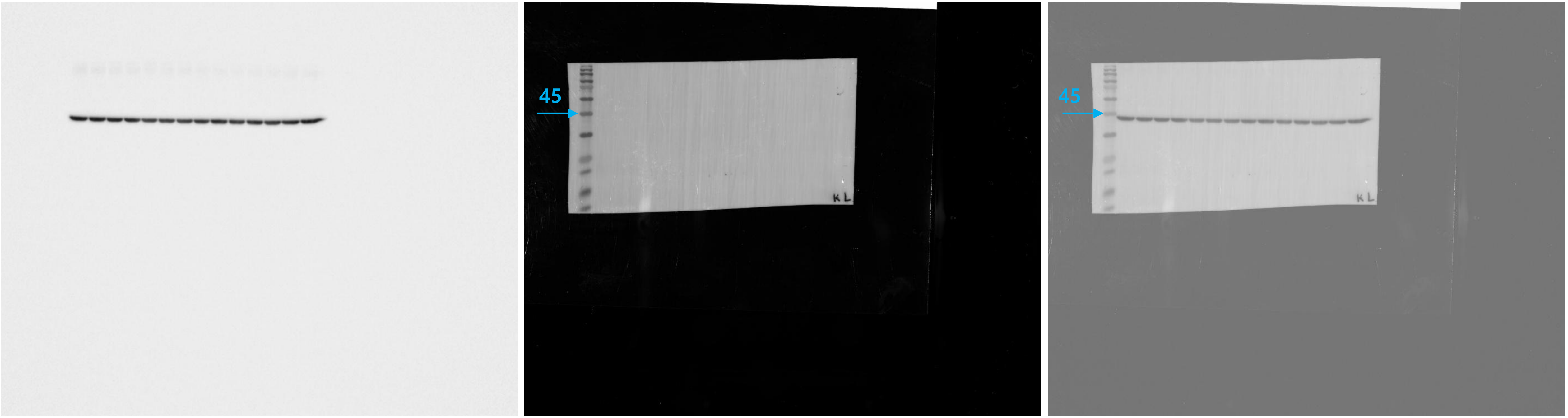

Figure2 G→ HSP70

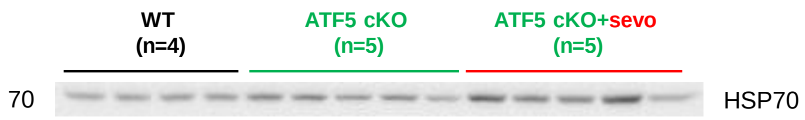

Sample bind

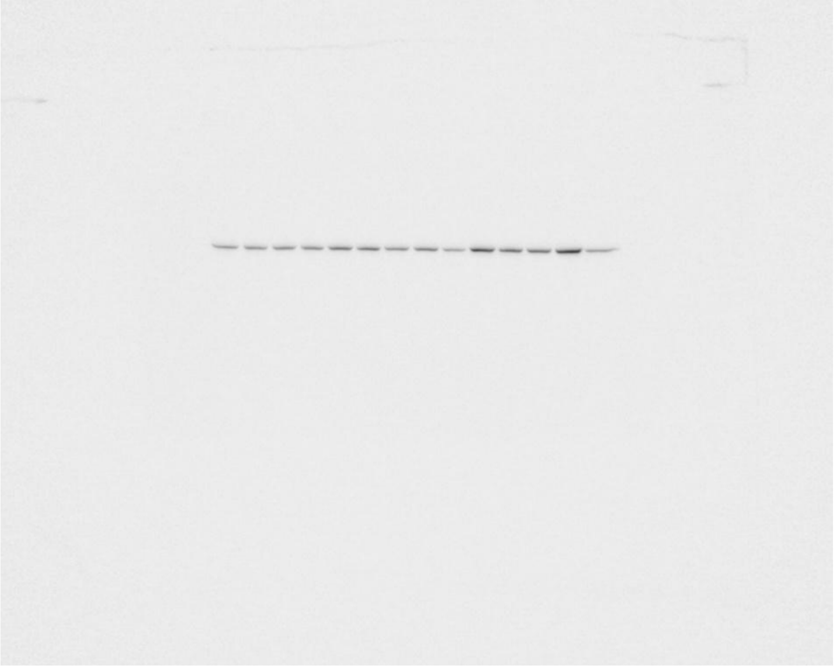

Protein marker

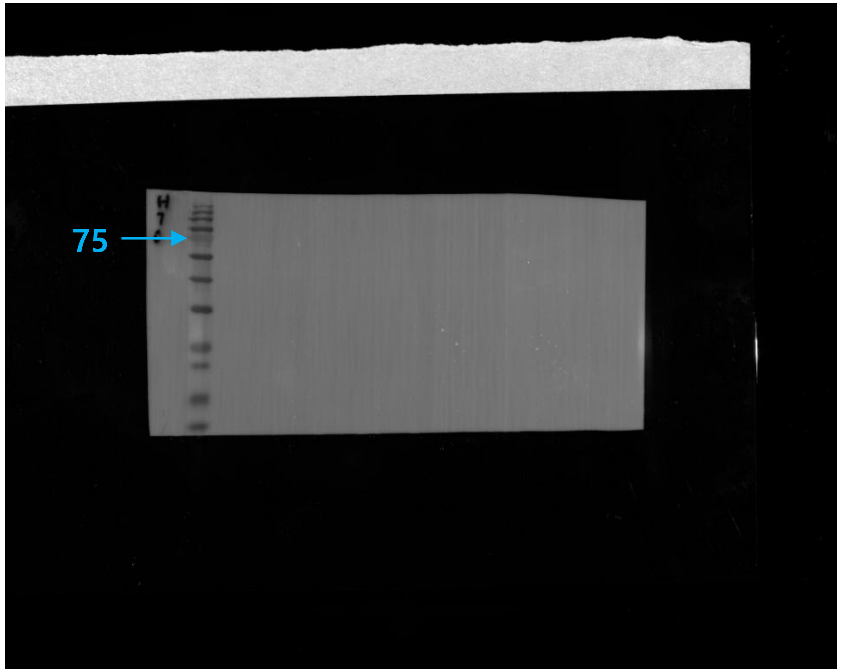

Merge

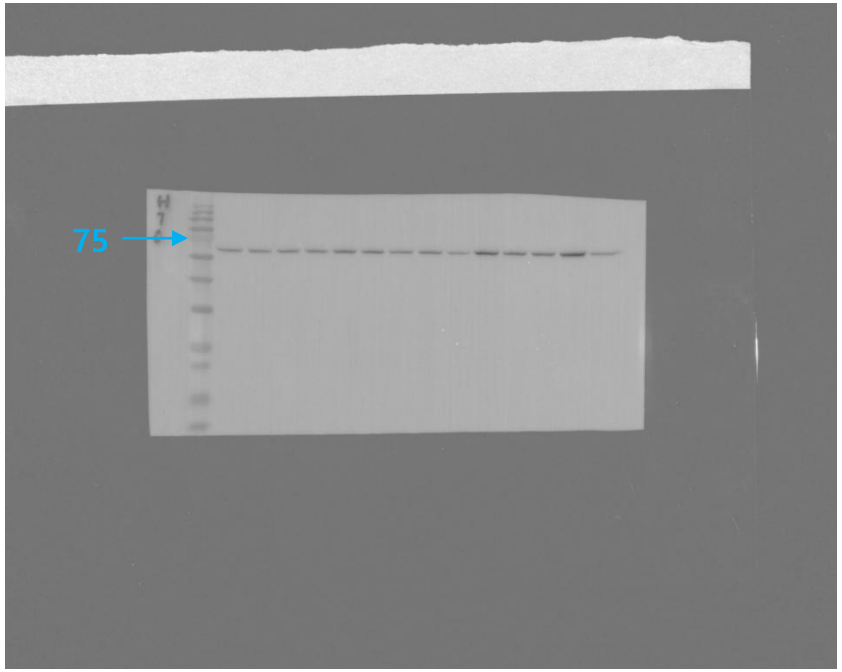

Figure2 G→ β-actin of HSP70

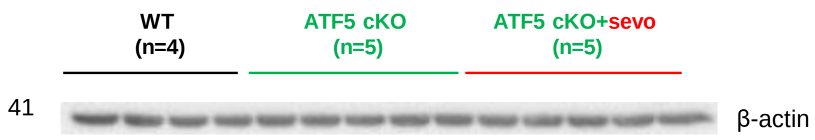

Sample bind

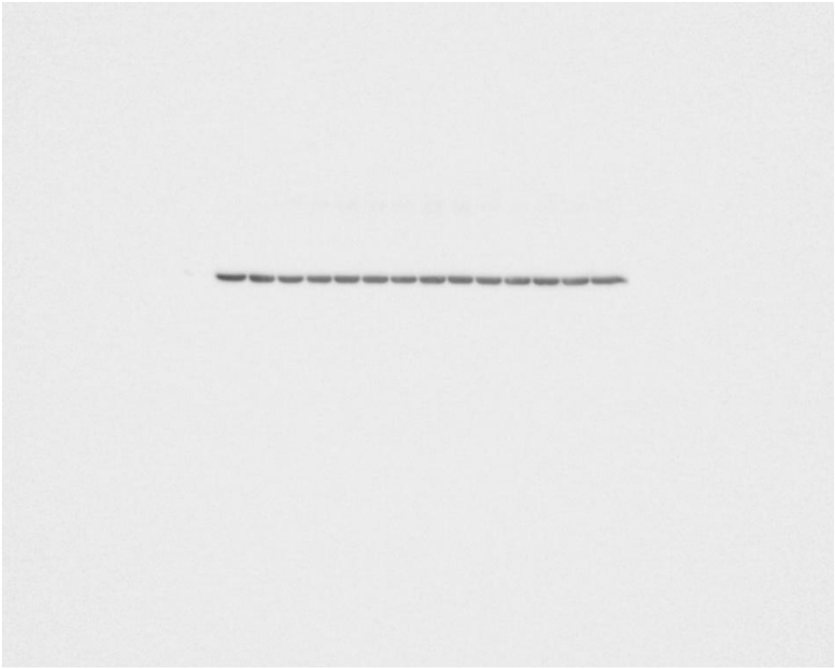

Protein marker

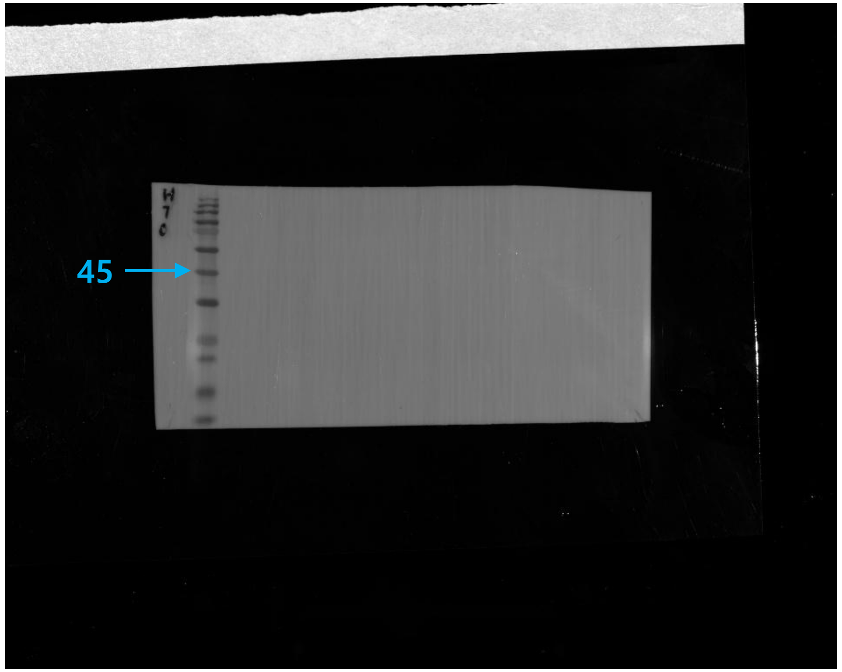

Merge

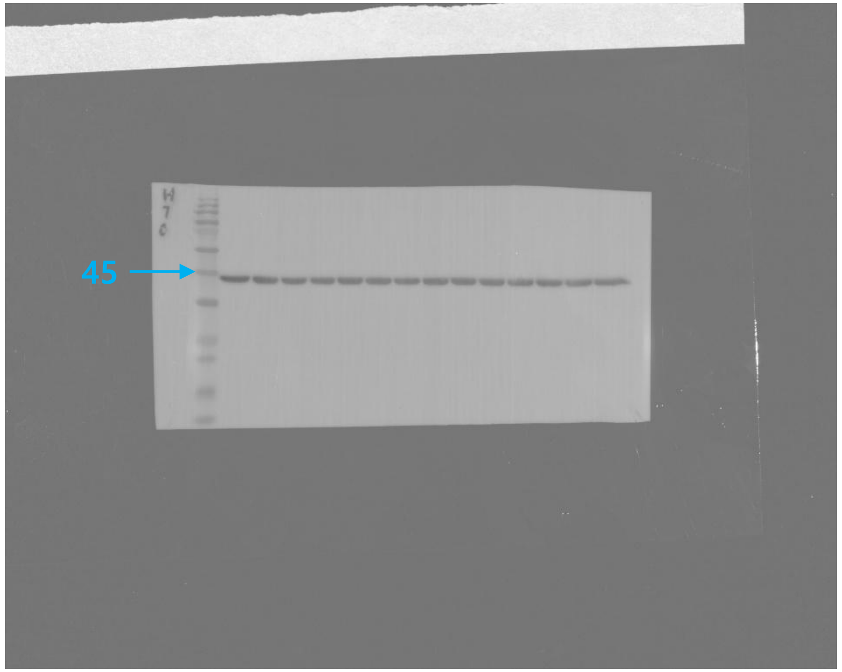

Figure2 G→ HSP60

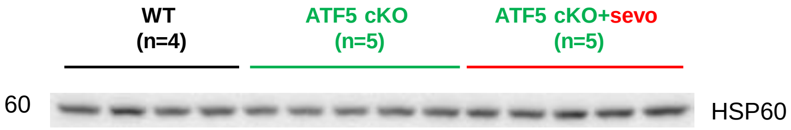

Sample bind

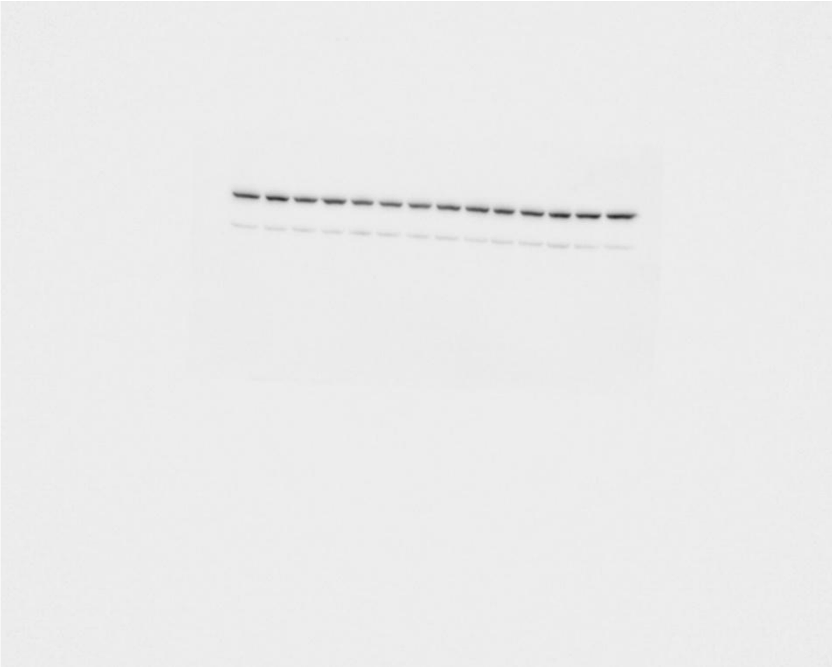

Protein marker

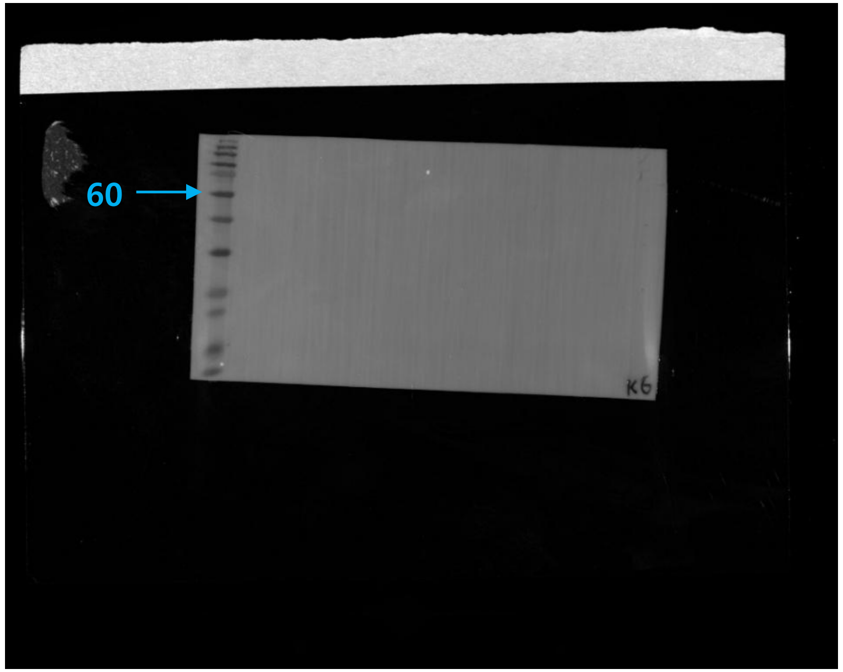

Merge

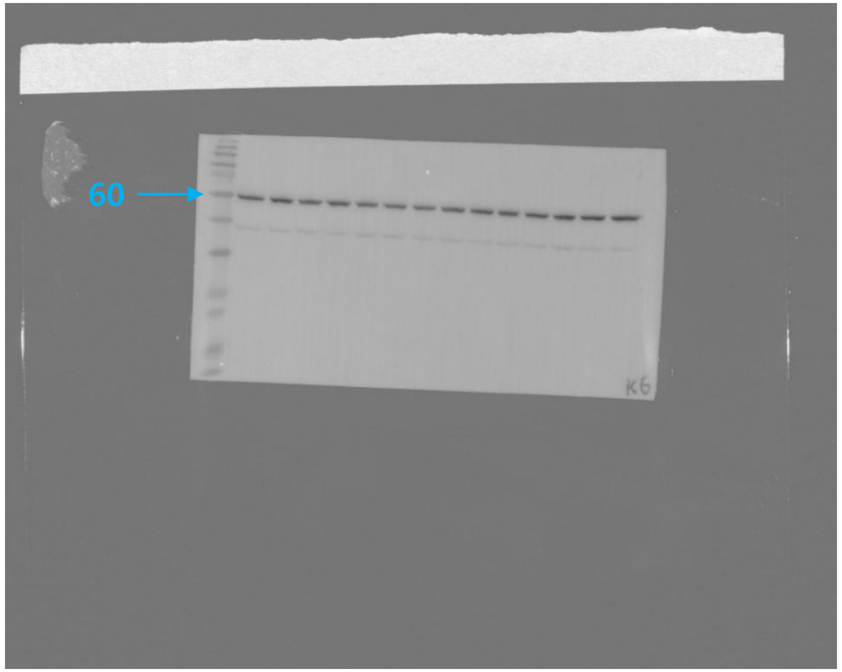

Figure2 G→ β-actin of HSP60

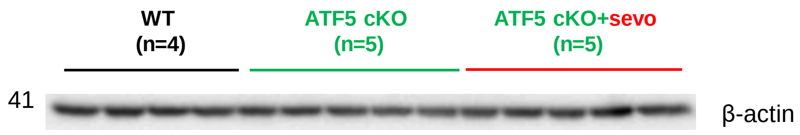

Sample bind

Protein marker

Merge

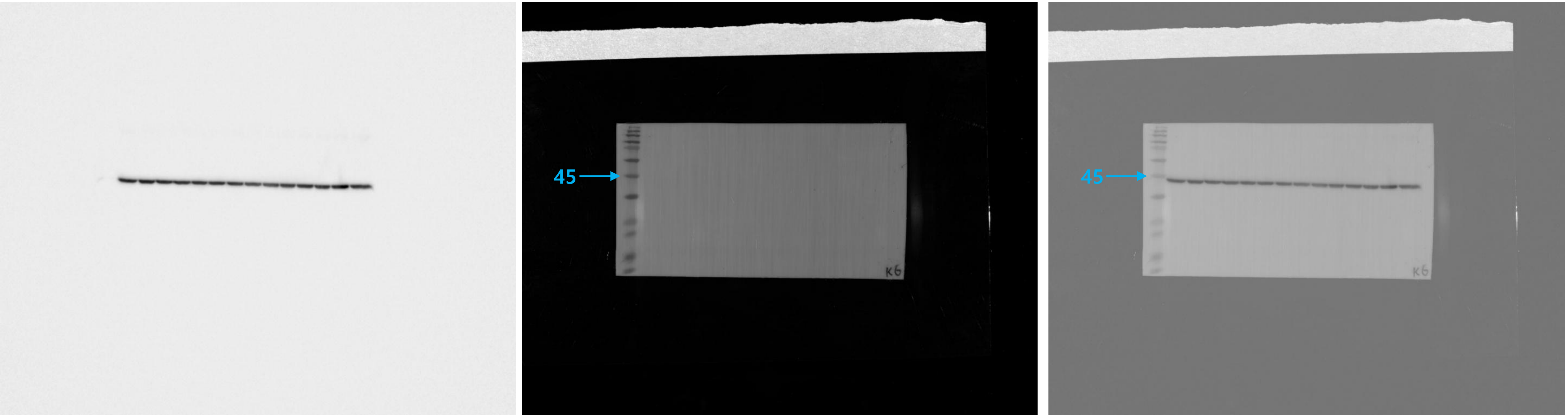

Figure2 G→ ATF5

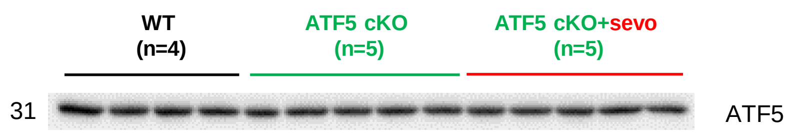

Sample bind

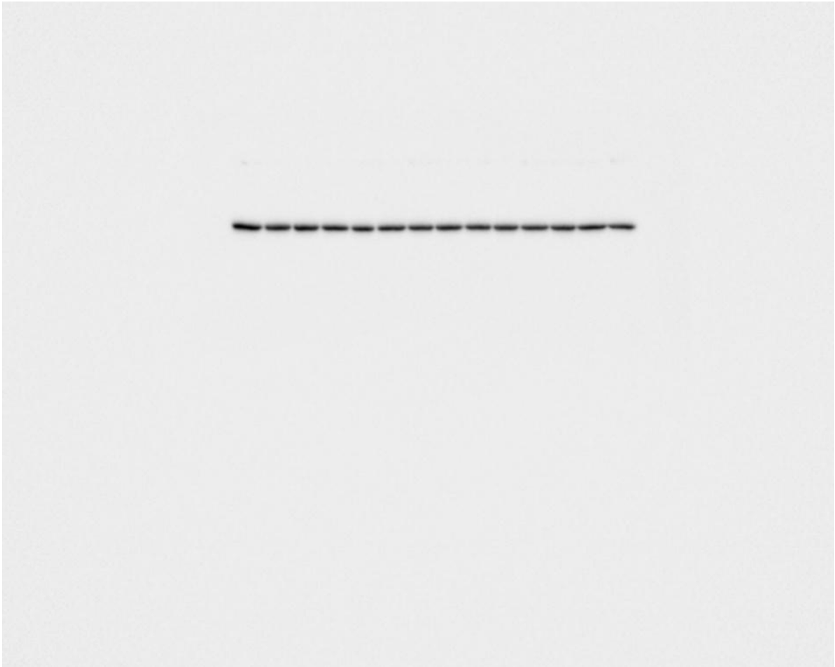

Protein marker

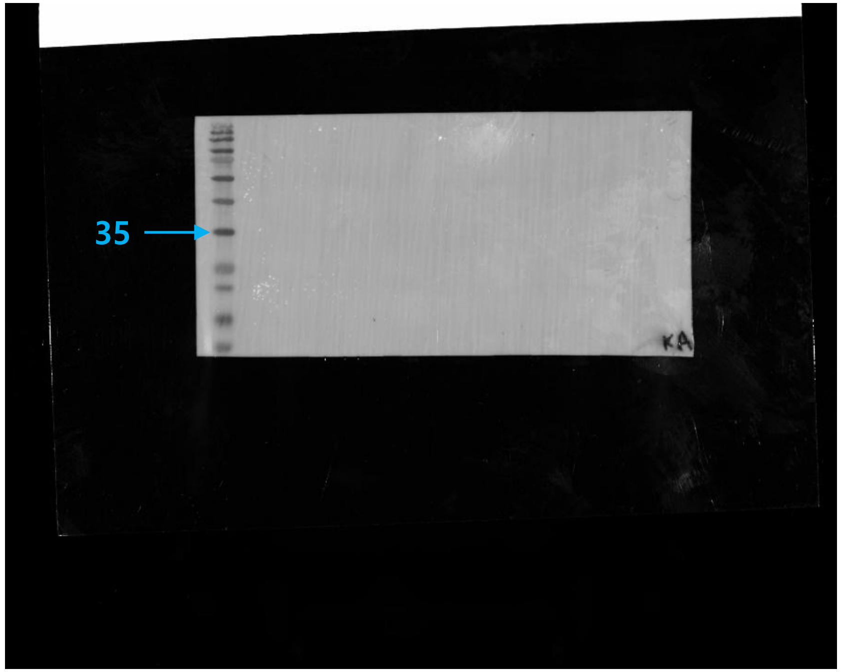

Merge

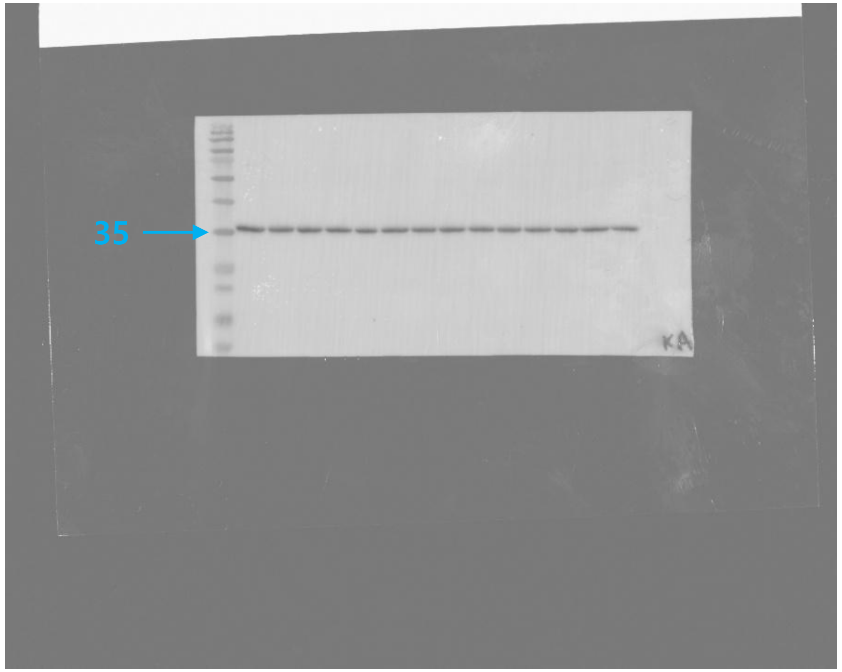

Figure2 G→ β-actin of ATF5

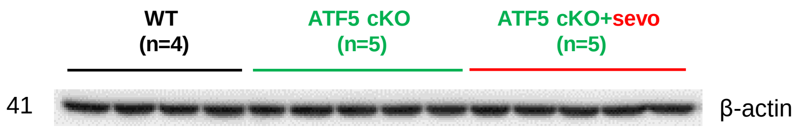

Sample bind Protein marker Merge

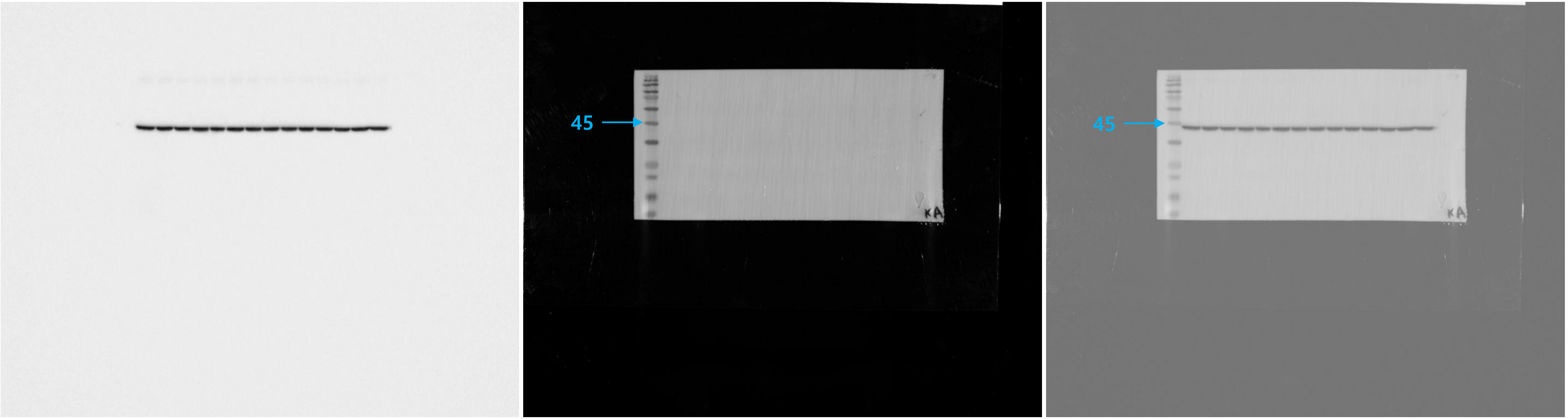

Figure2 G→ CLPP

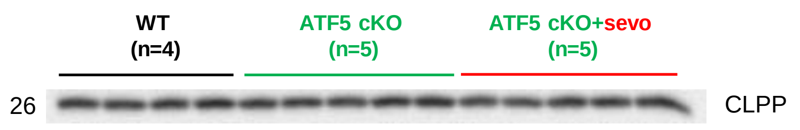

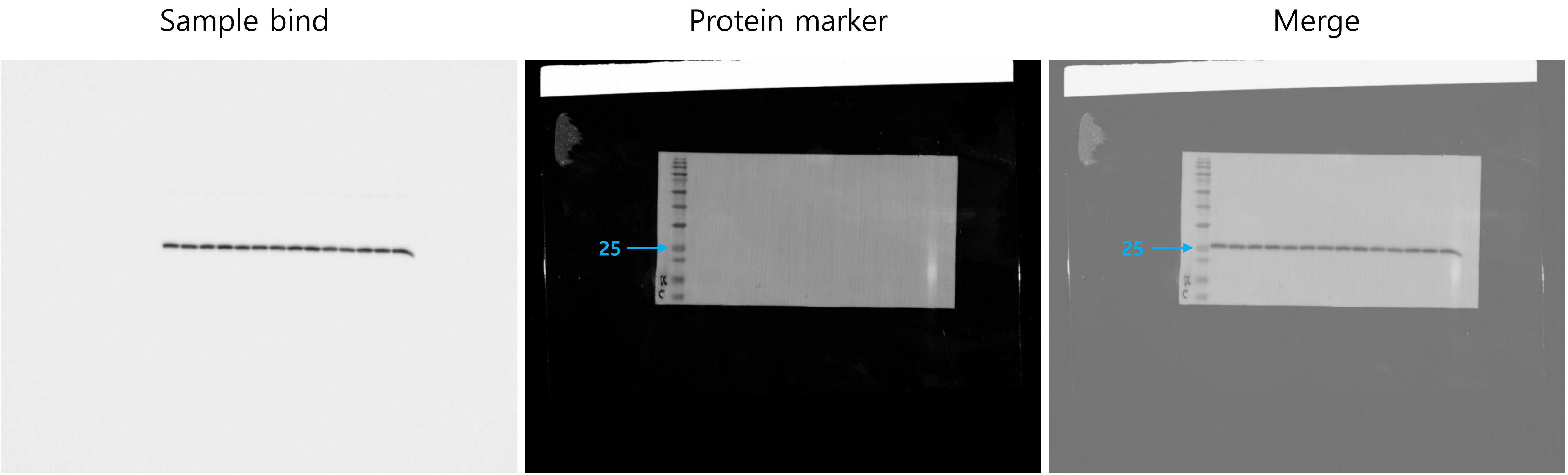

Figure2 G→ β-actin of CLPP

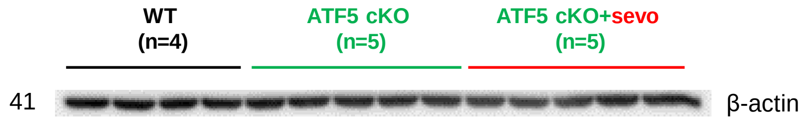

Sample bind

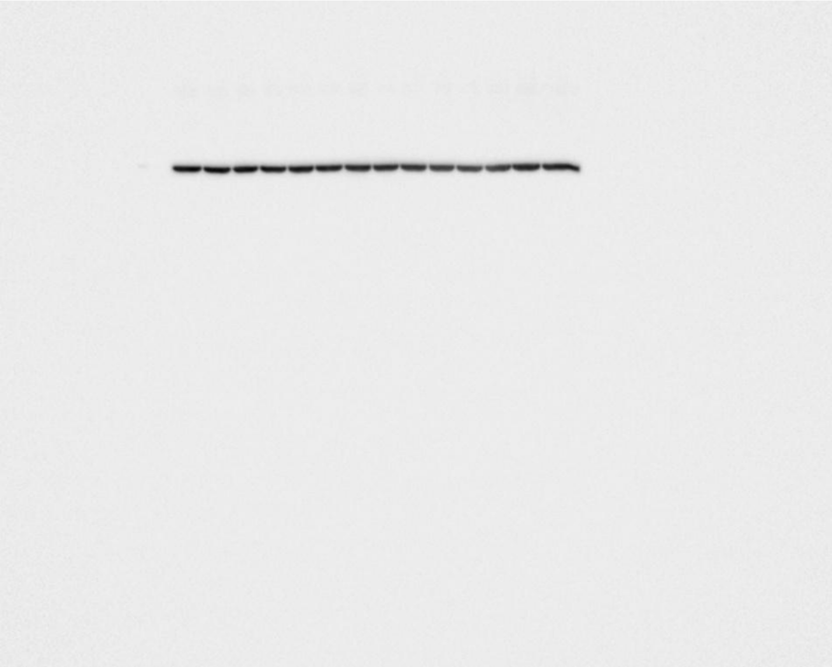

Protein marker

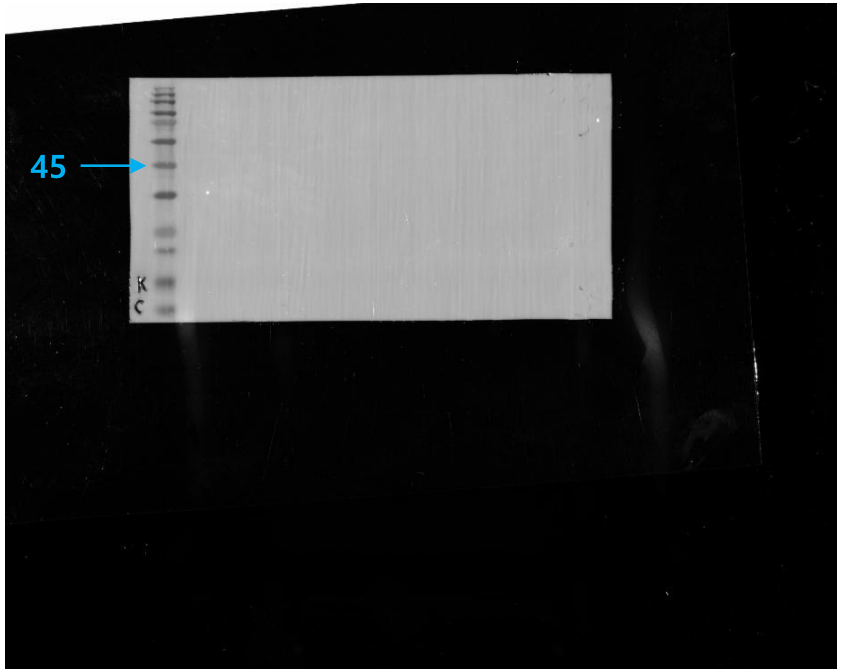

Merge

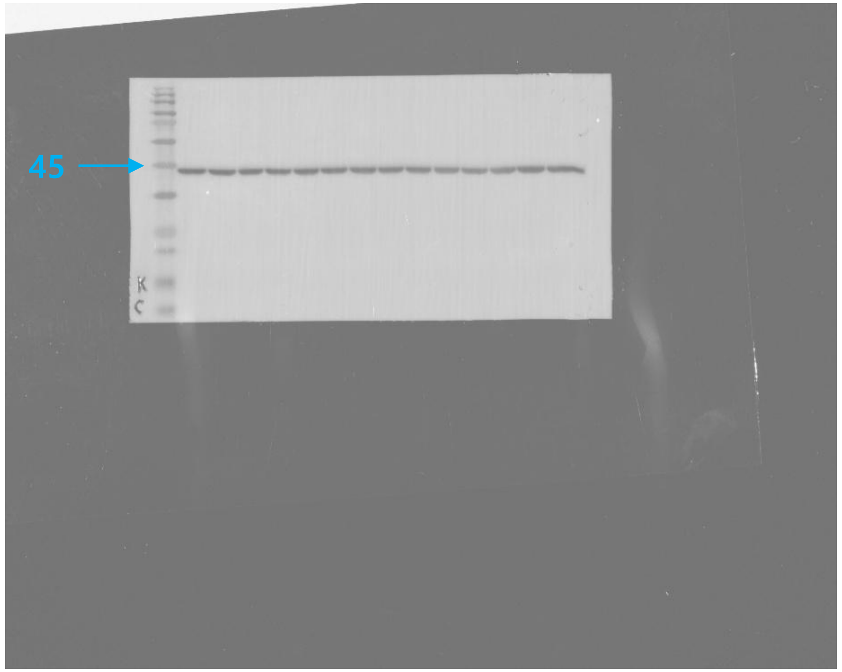

Figure3 F→ LONP1

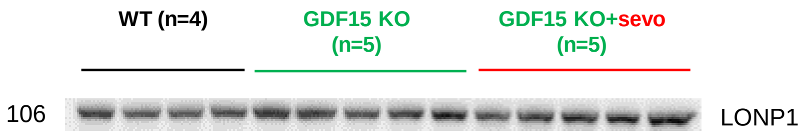

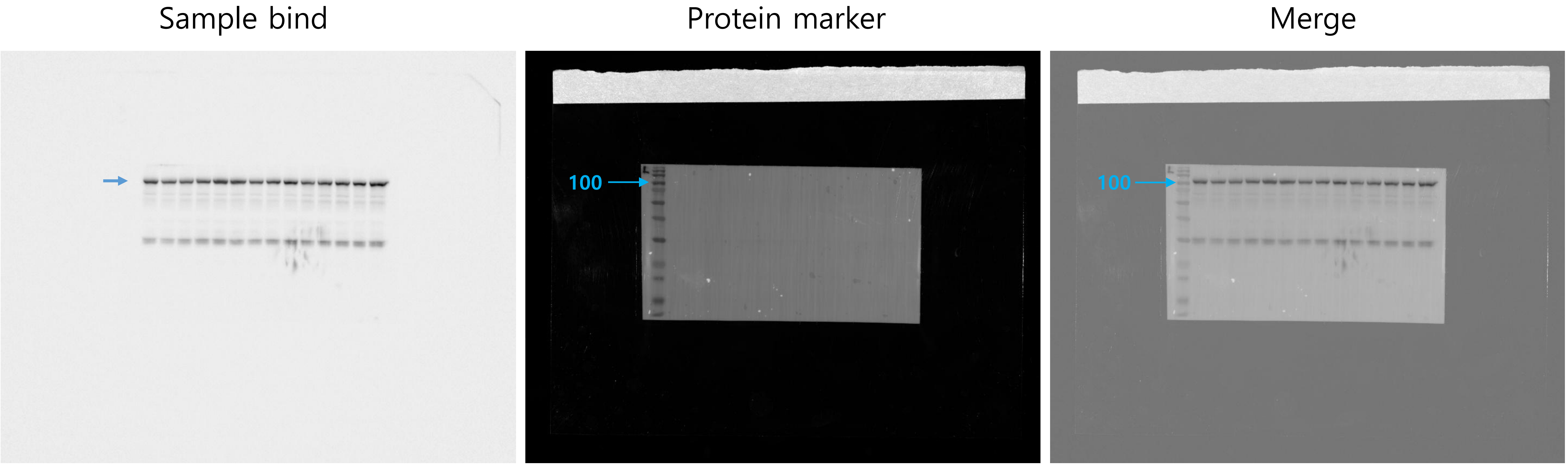

Figure3 F→ β-actin of LONP1

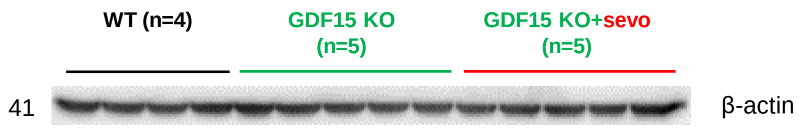

Sample bind

Protein marker

Merge

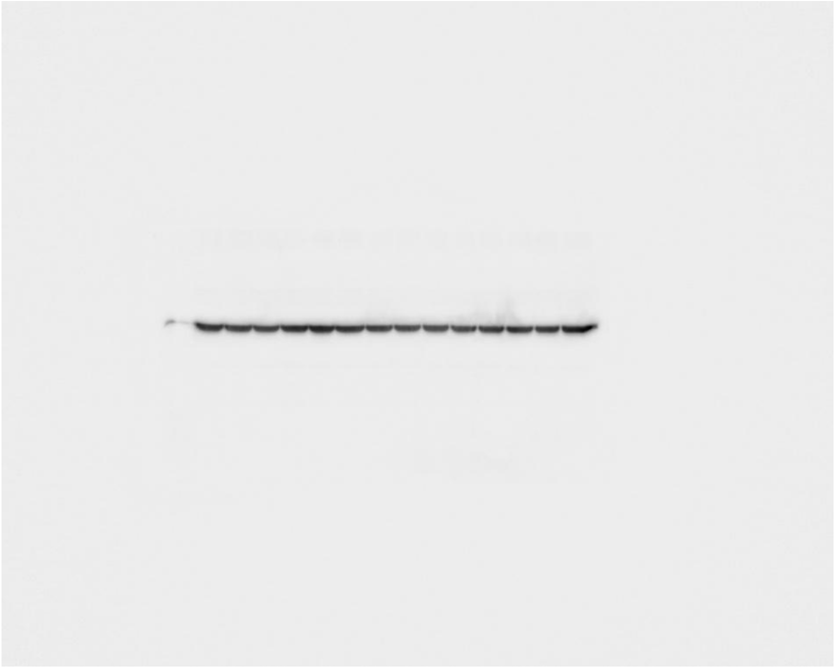

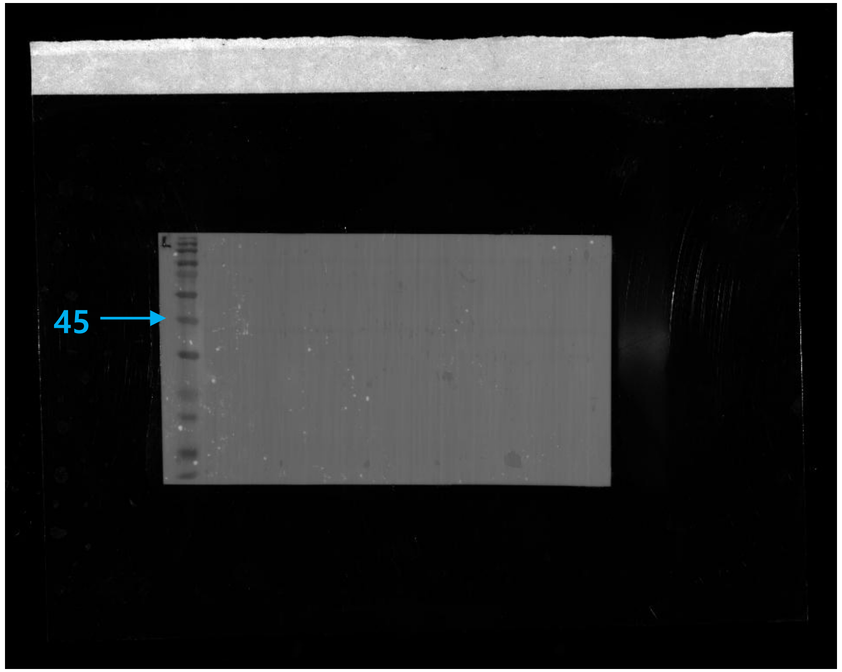

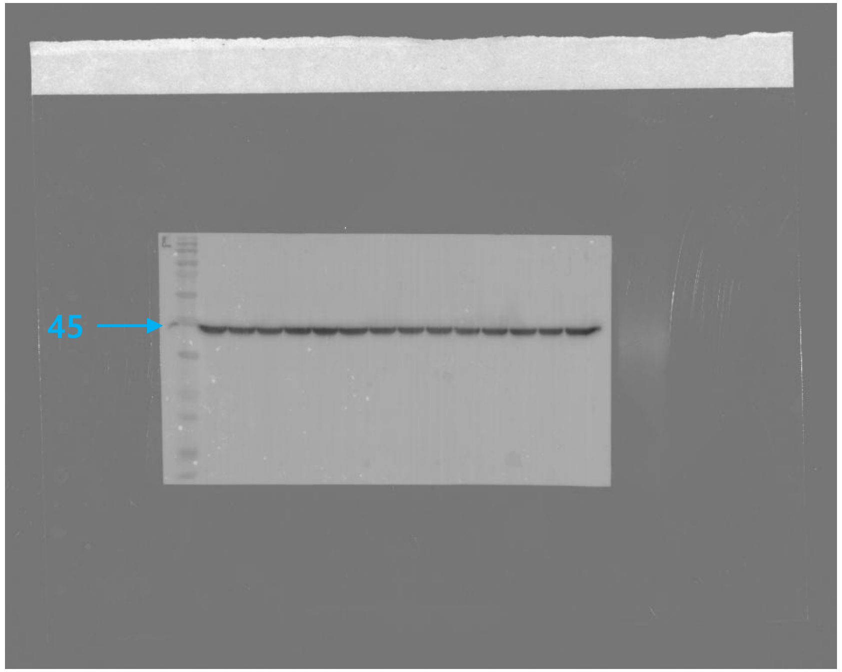

Figure3 F→ HSP70

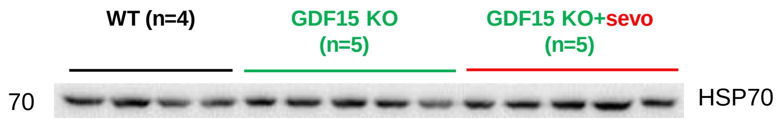

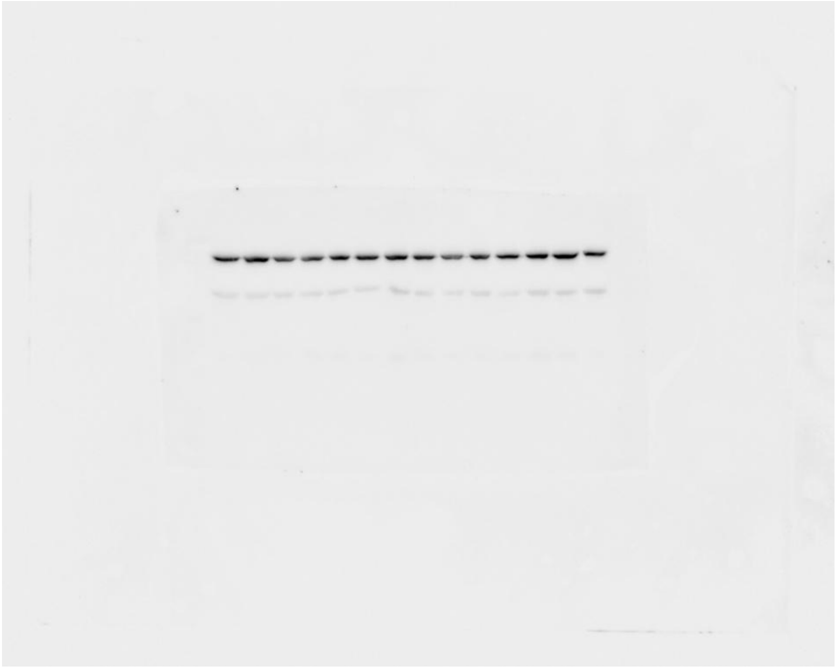

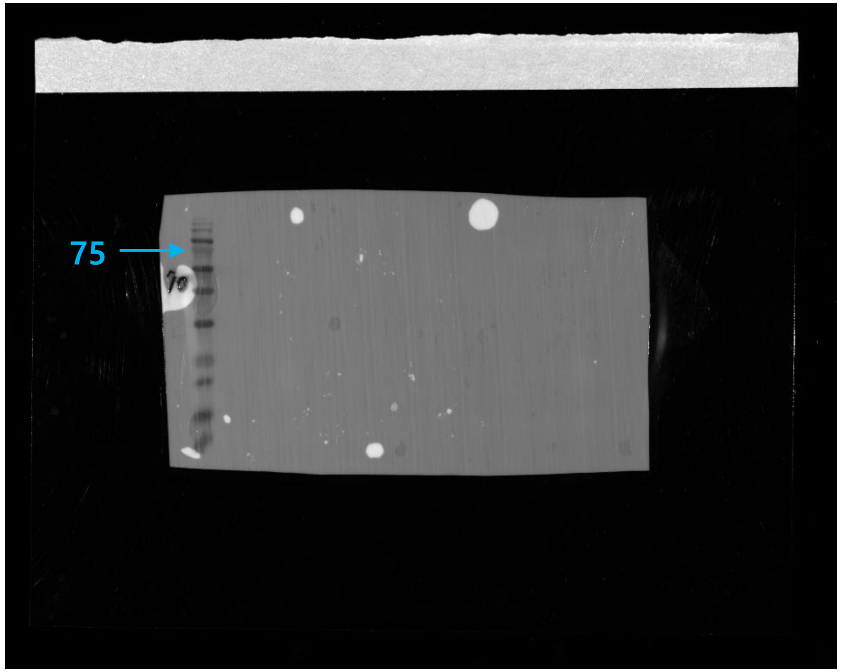

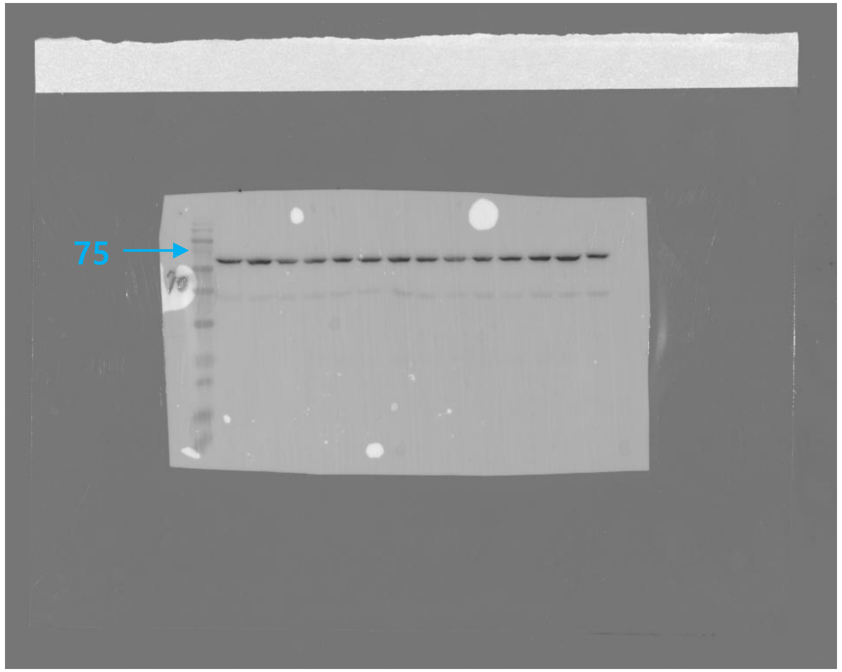

Figure3 F→ β-actin of HSP70

2025.08.07

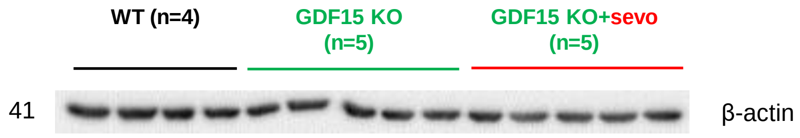

Sample bind

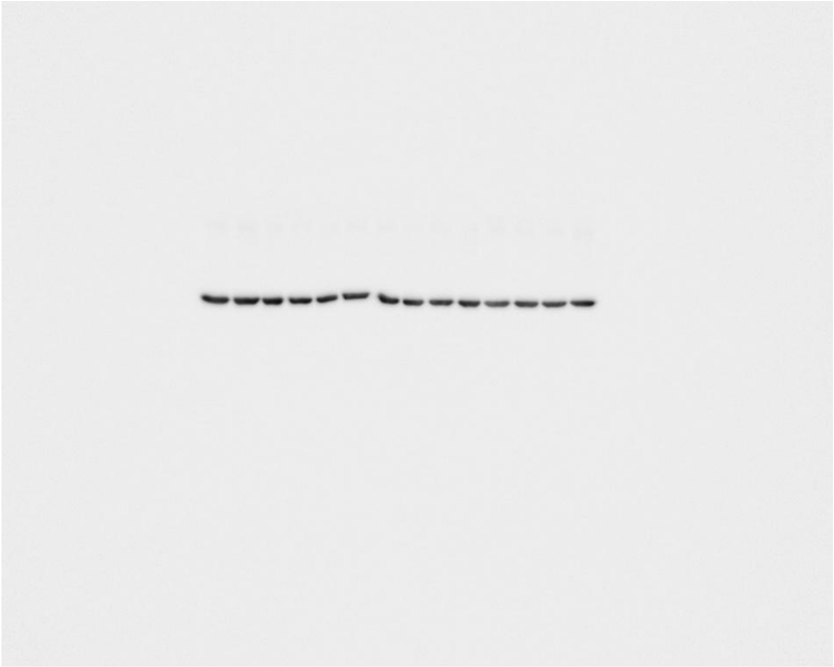

Protein marker

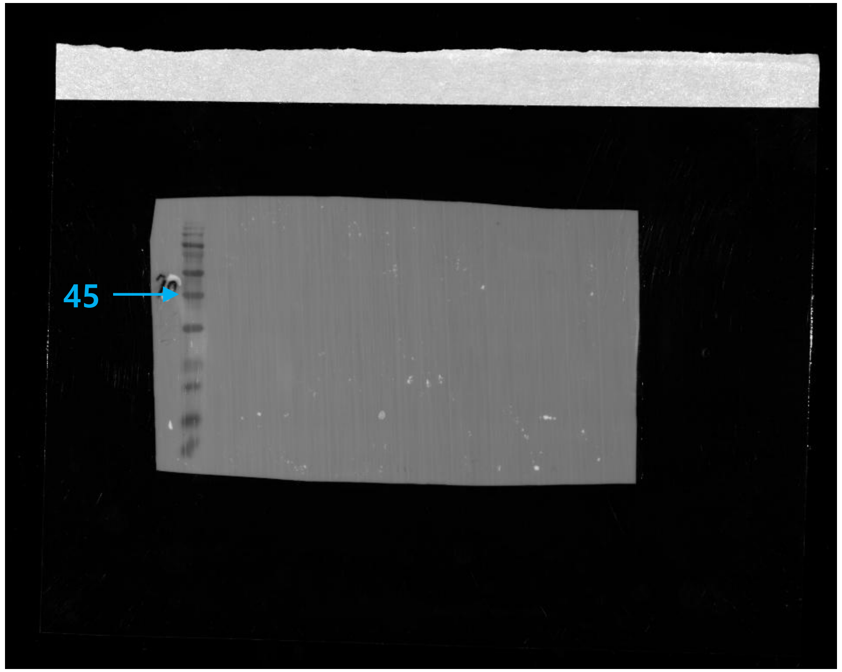

Merge

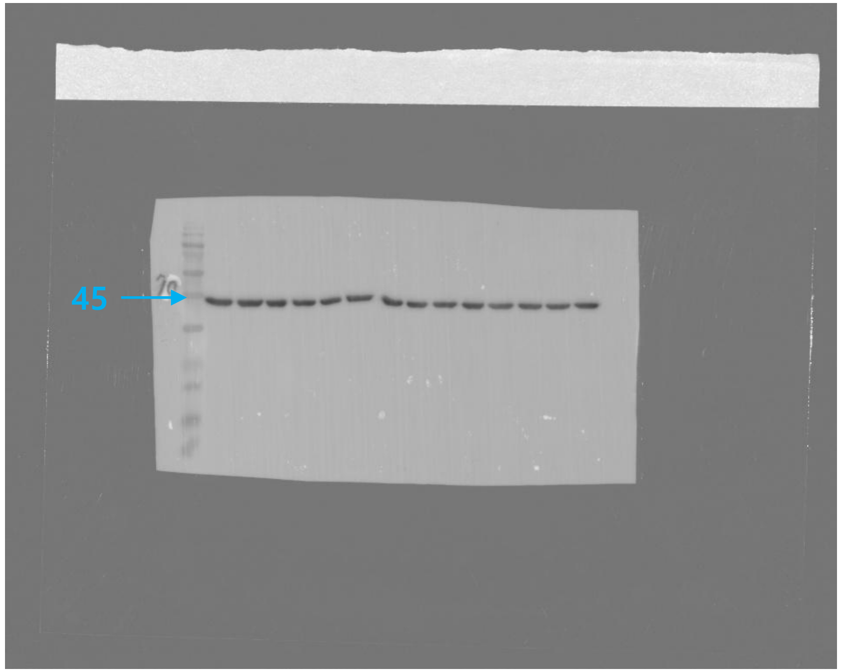

Figure3 F → HSP60

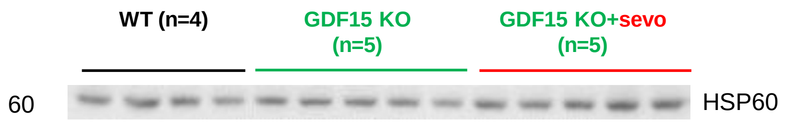

Sample bind

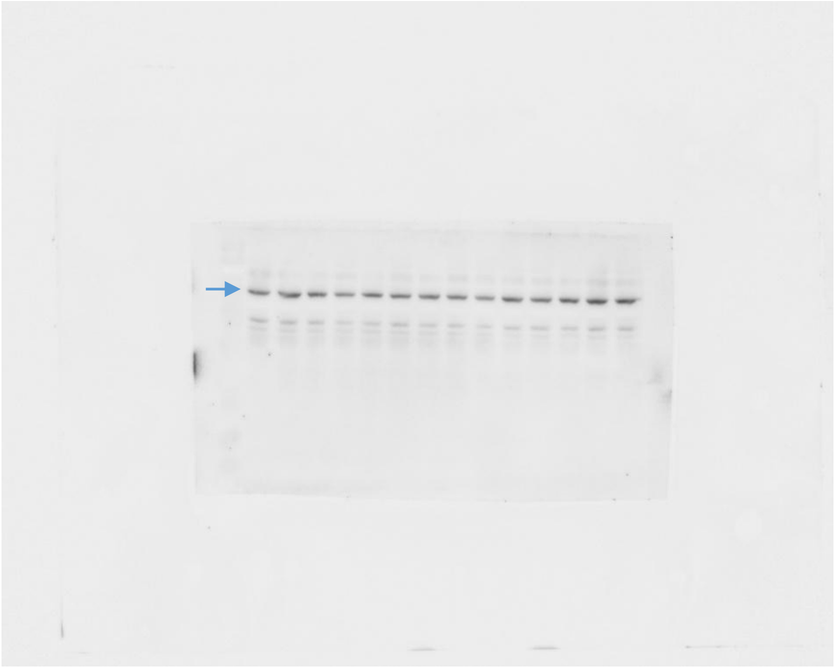

Protein marker

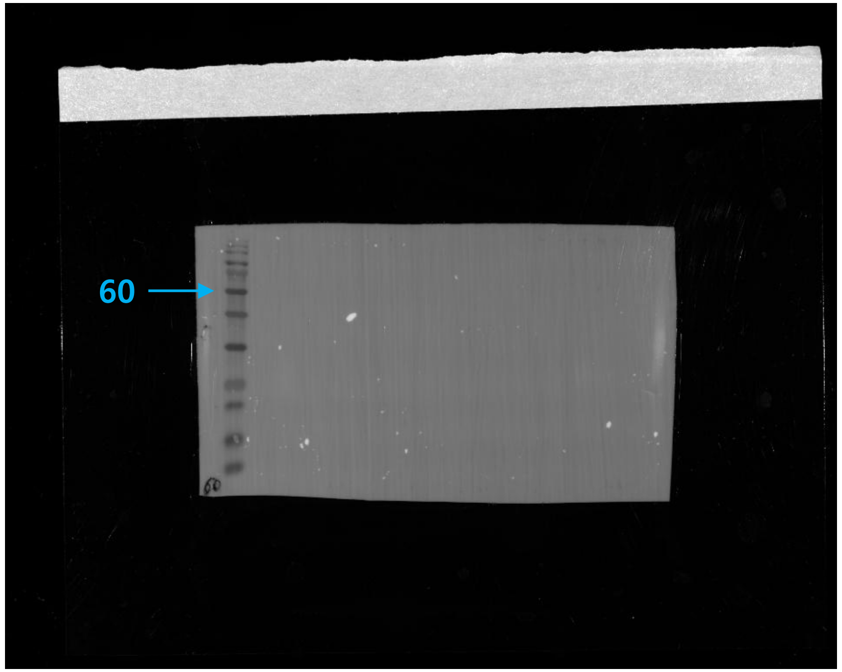

Merge

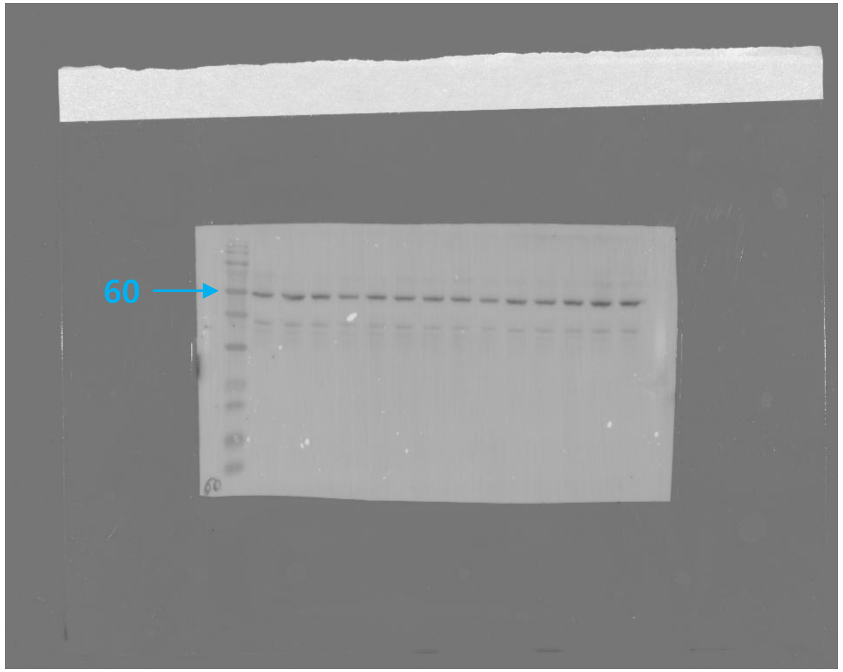

Figure3 F → β-actin of HSP60

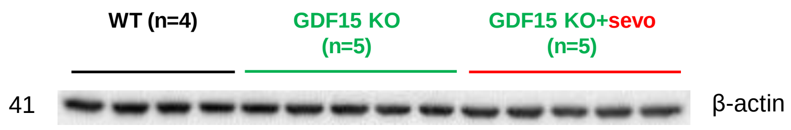

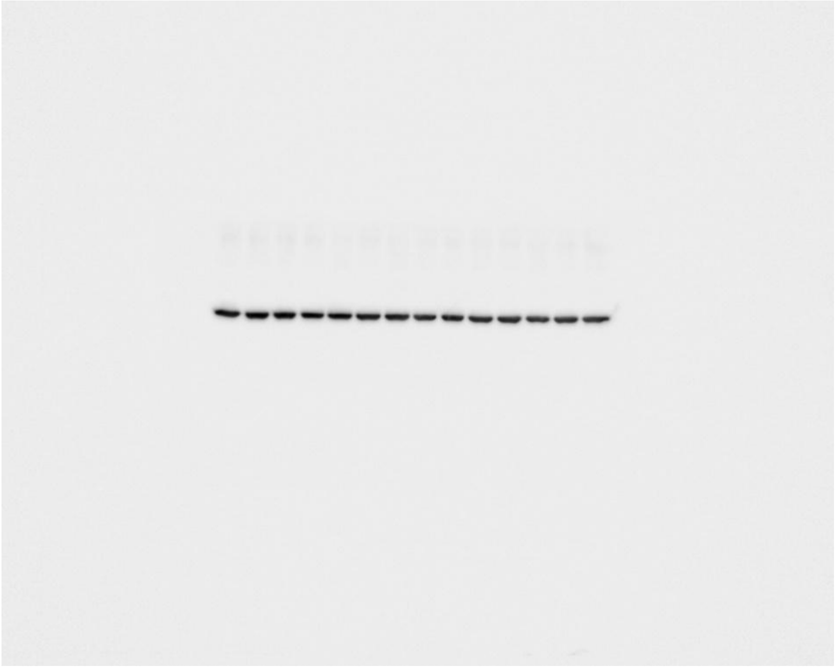

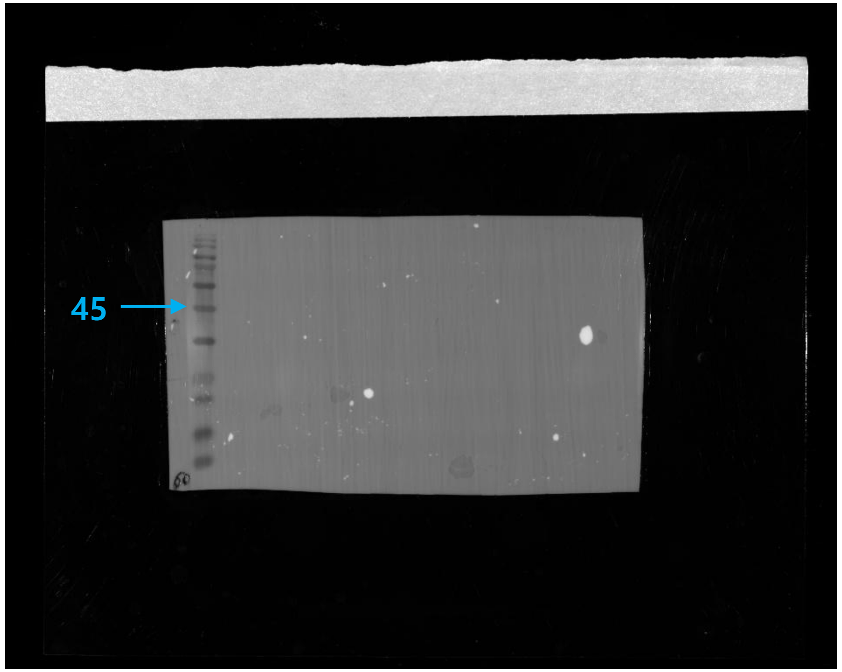

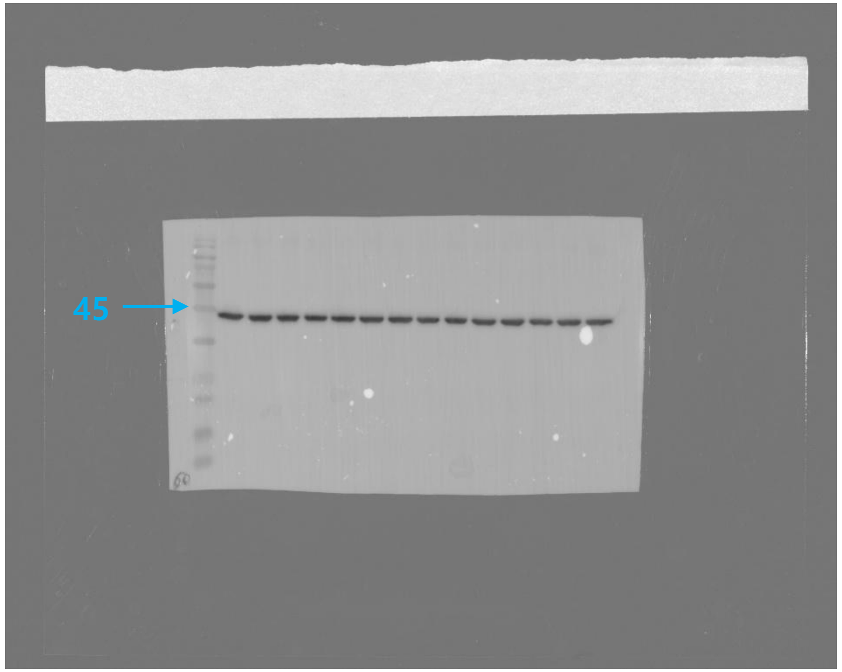

Figure3 F → ATF5

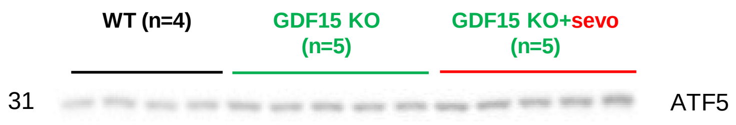

Sample bind

Protein marker

Merge

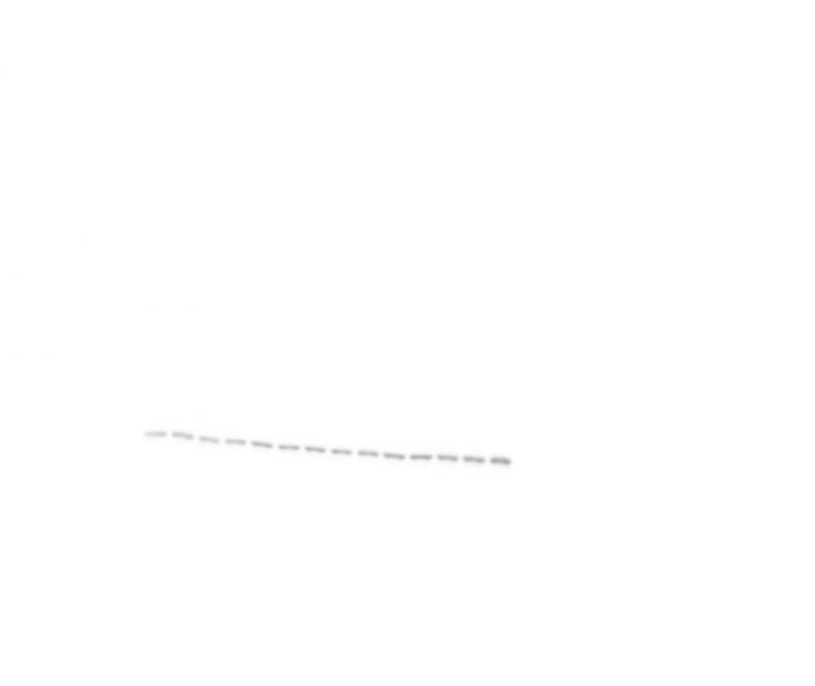

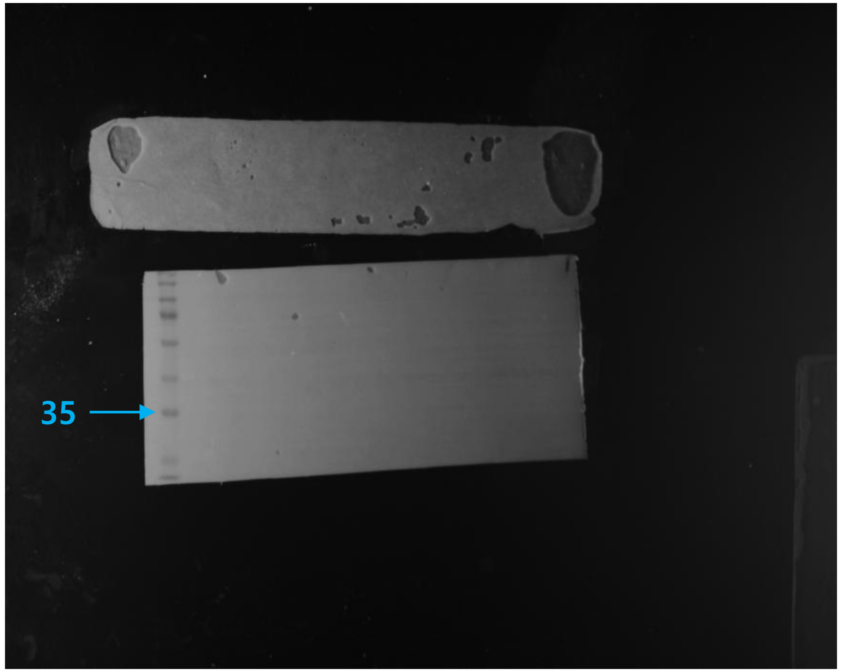

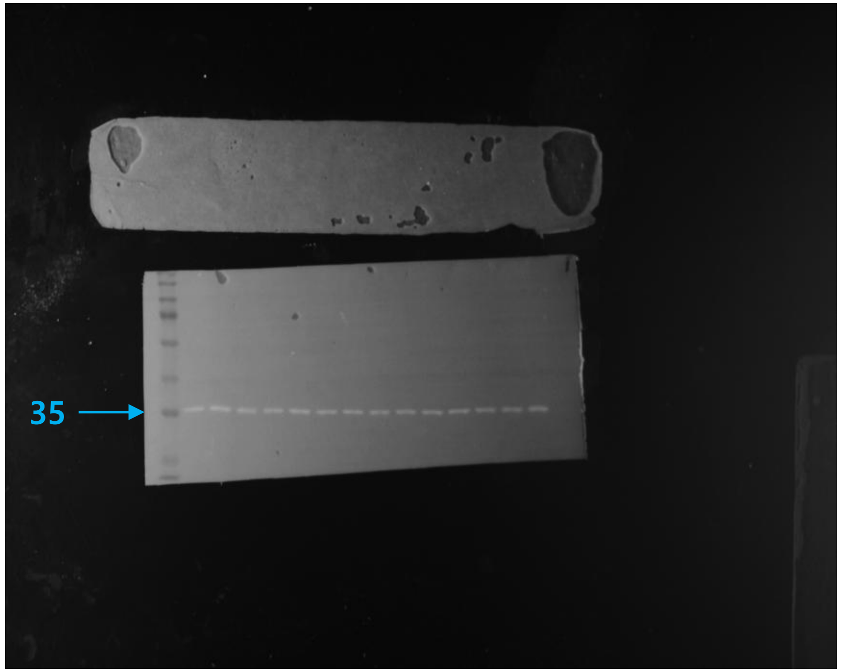

Figure3 F → β-actin of ATF5

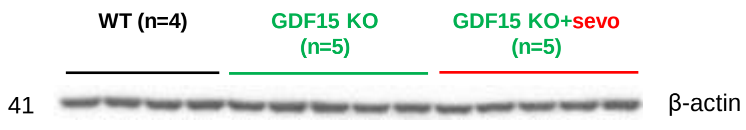

Sample bind

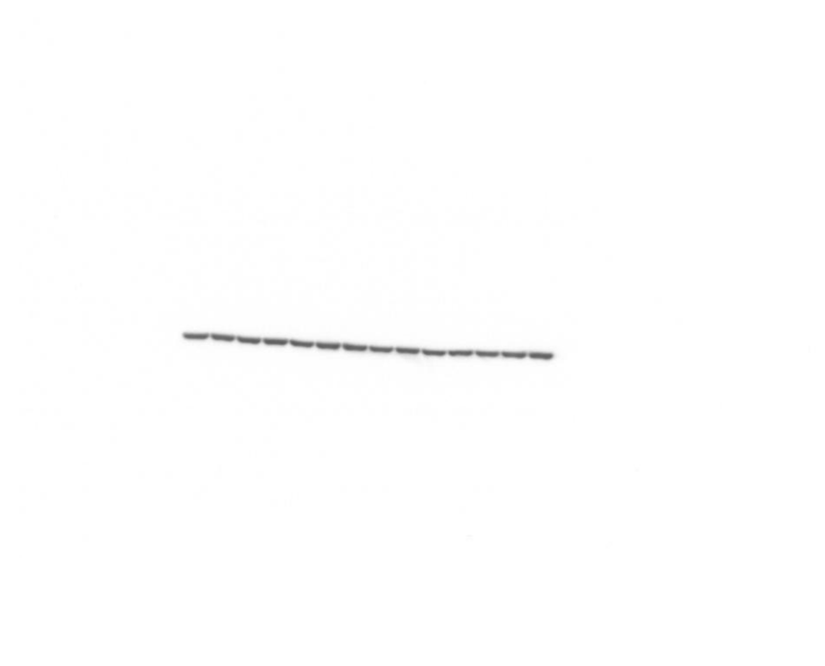

Protein marker

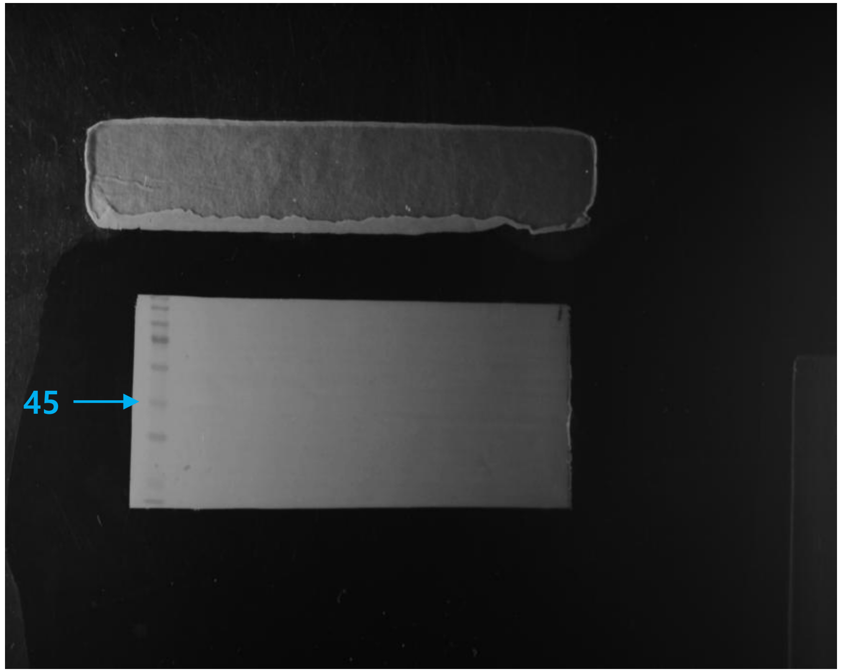

Merge

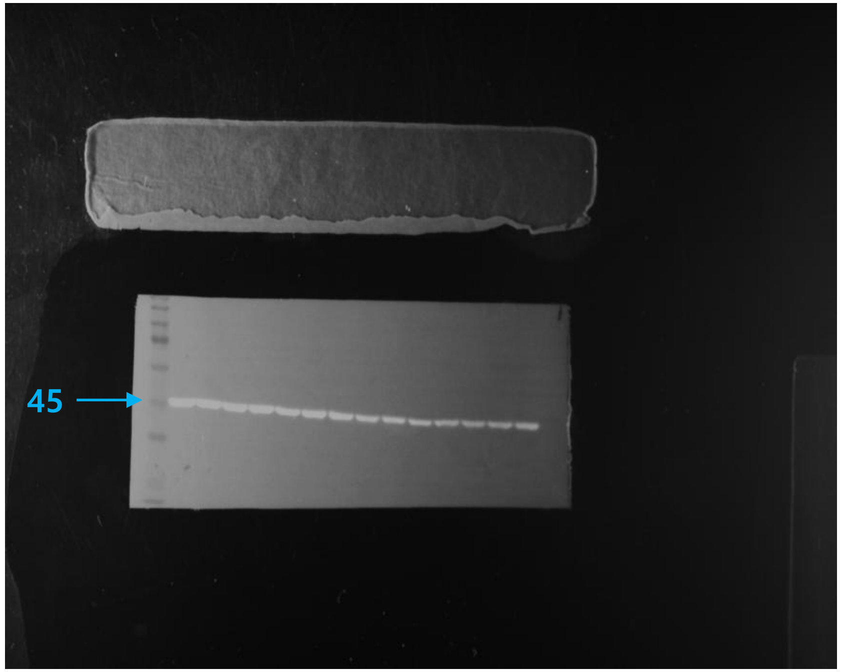

Figure3 F → CLPP

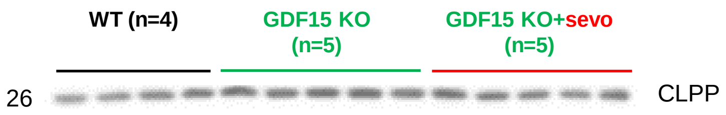

Sample bind

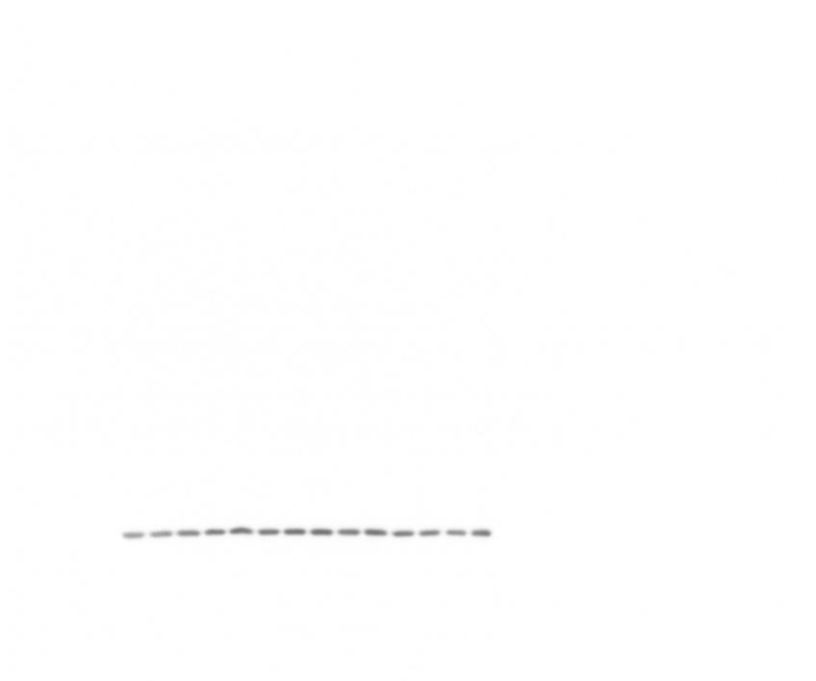

Protein marker

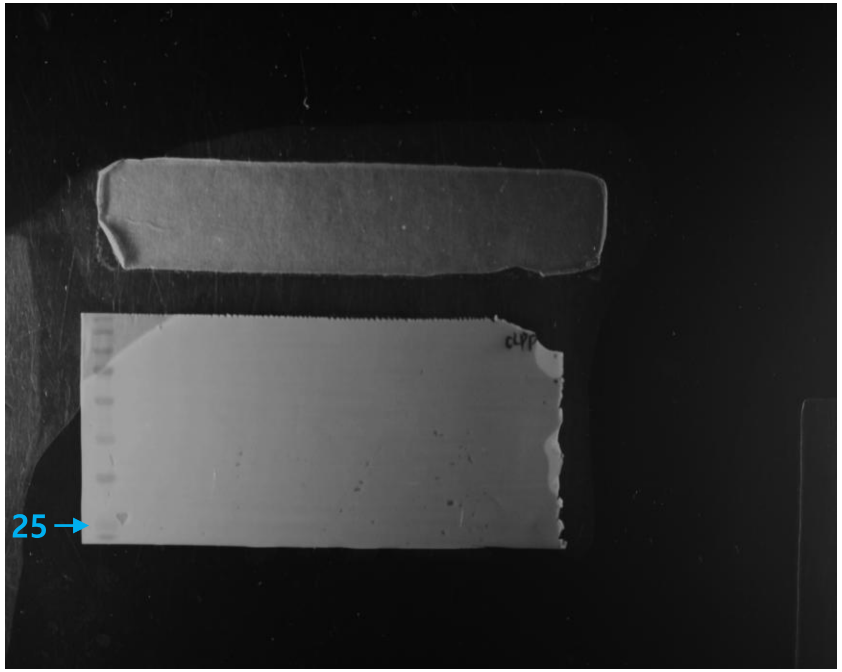

Merge

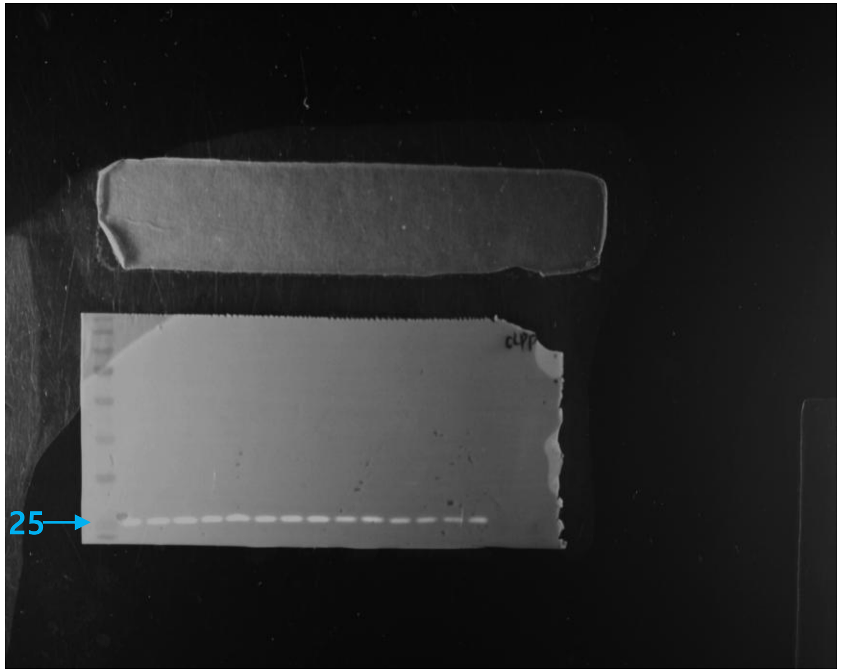

Figure3 F → β-actin of CLPP

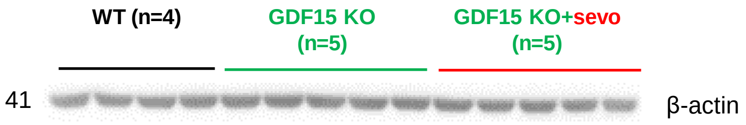

Sample bind

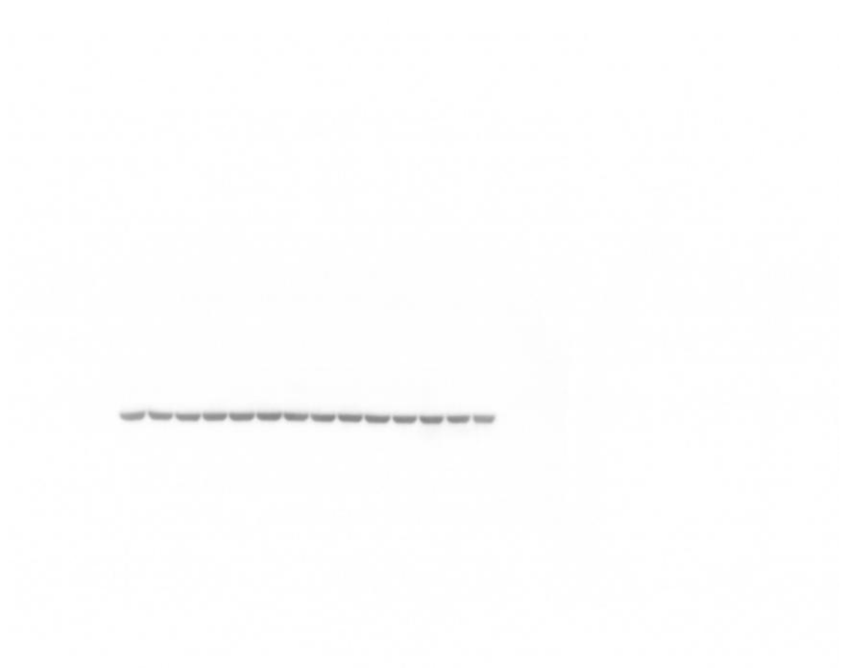

Protein marker

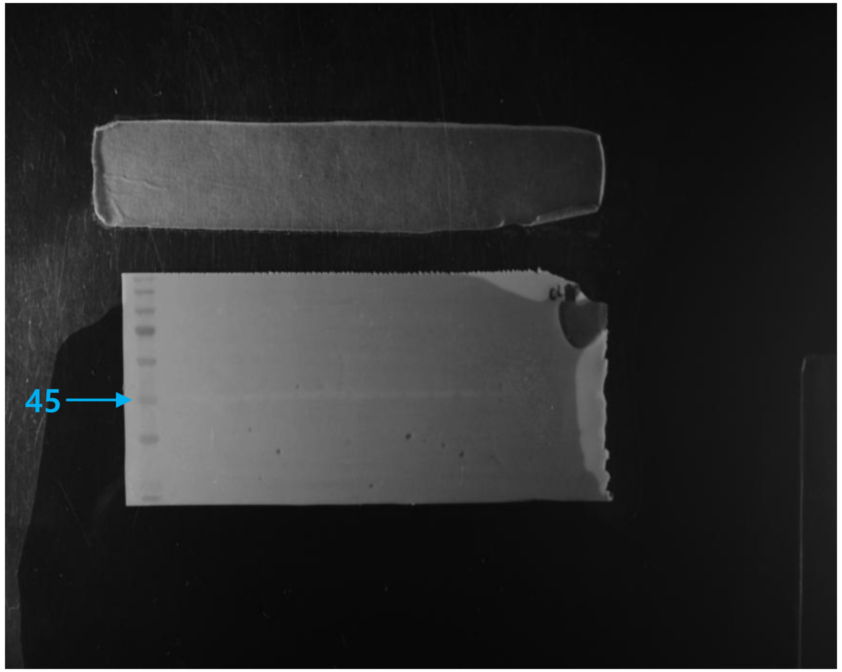

Merge

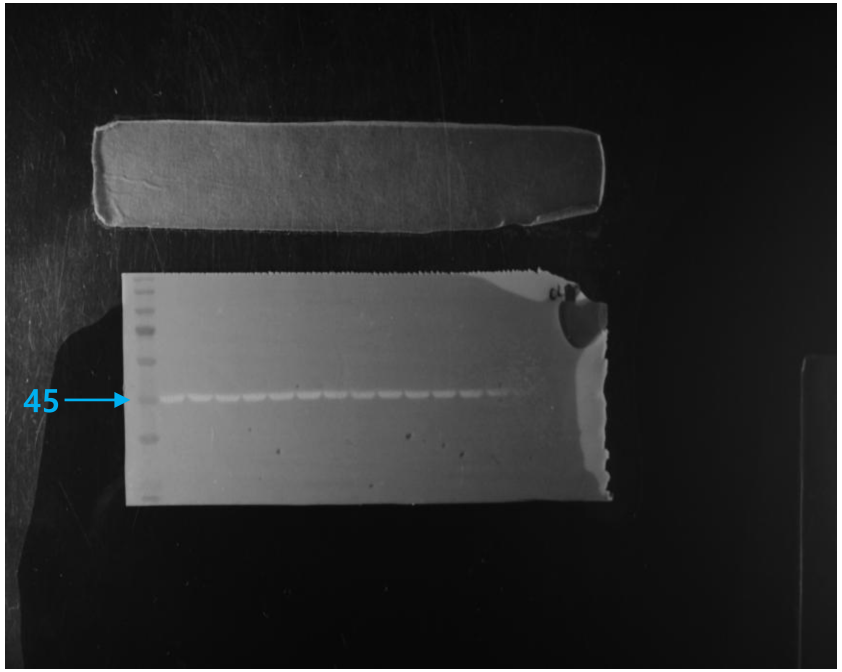

Figure4 B → LONP1

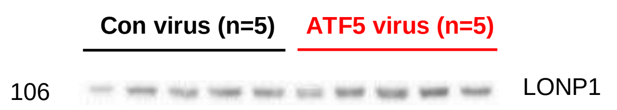

Sample bind

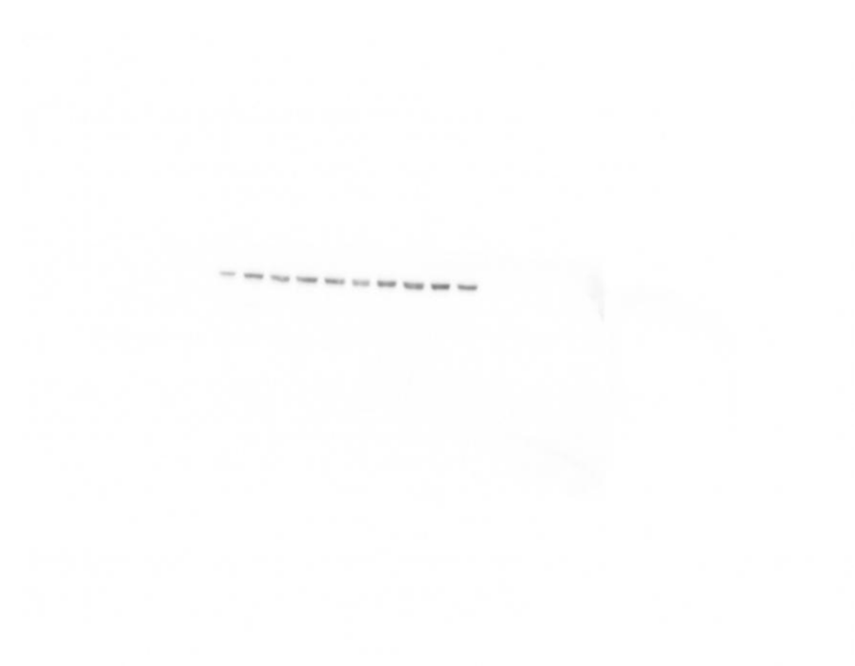

Protein marker

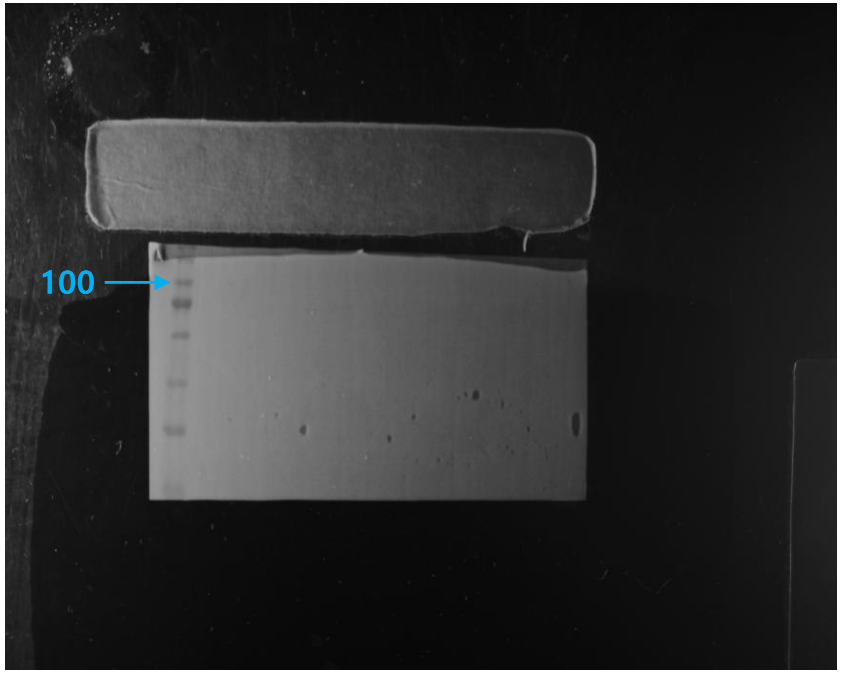

Merge

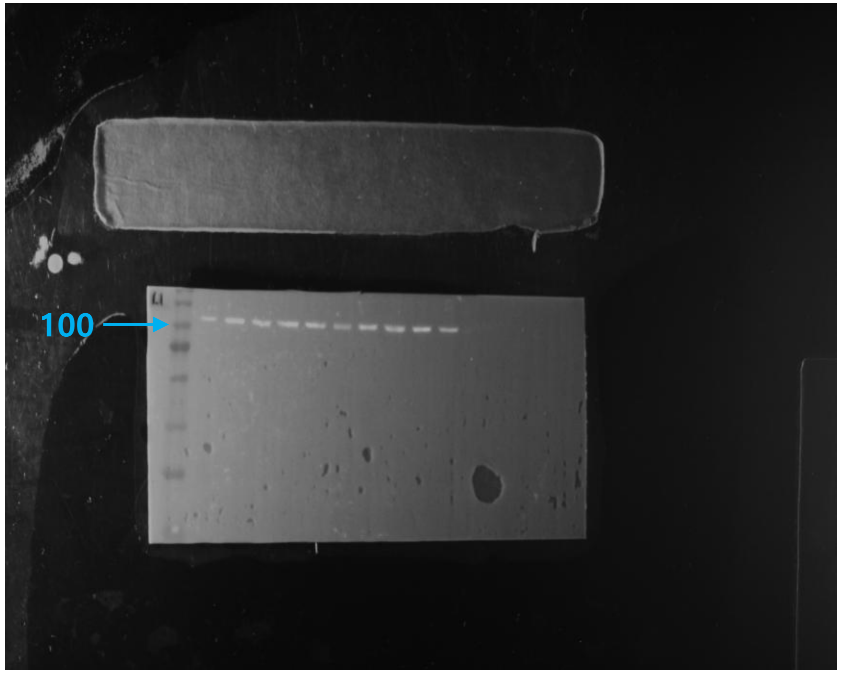

Figure4 B → β-ACTIN of LONP1

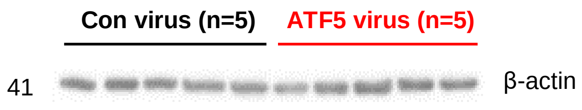

Sample bind

Protein marker

Merge

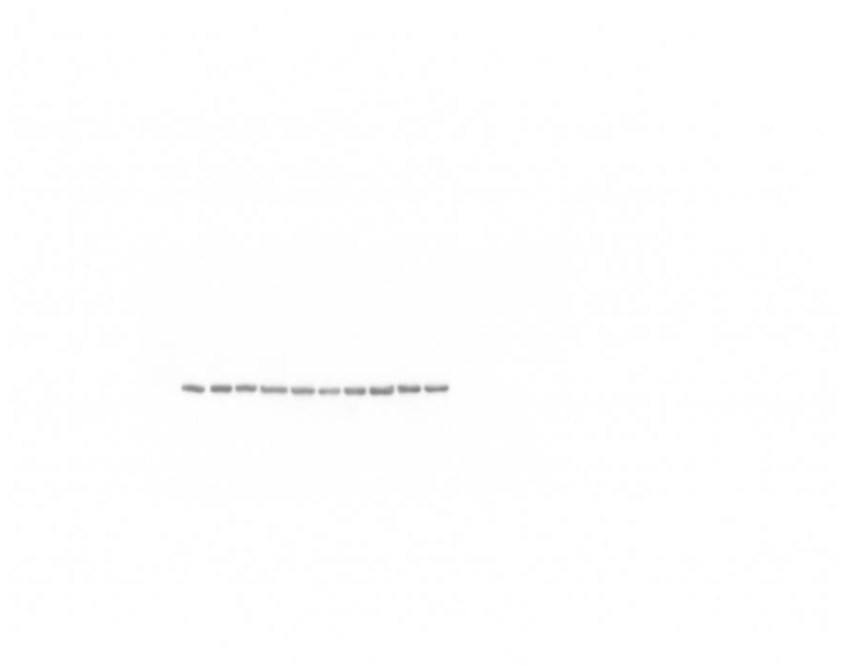

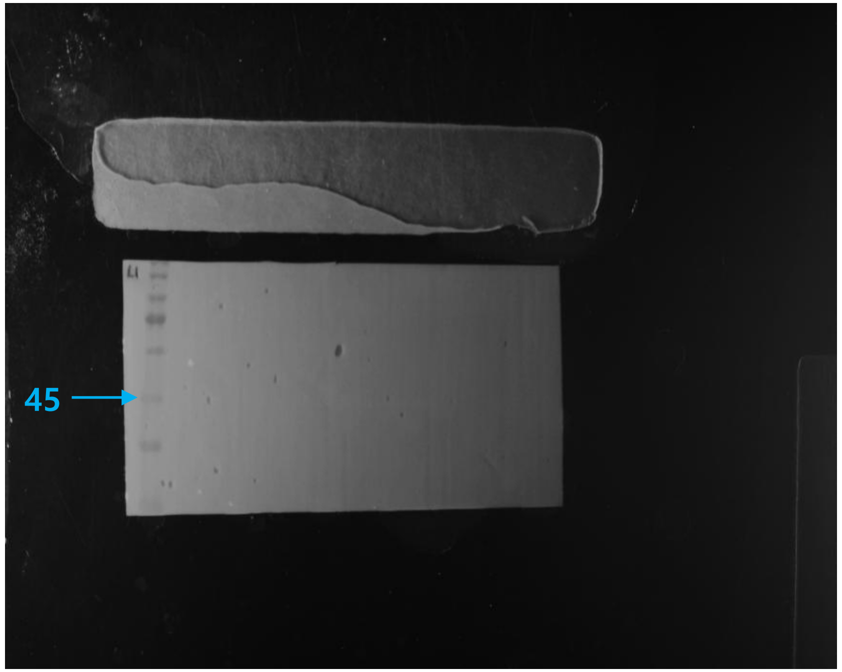

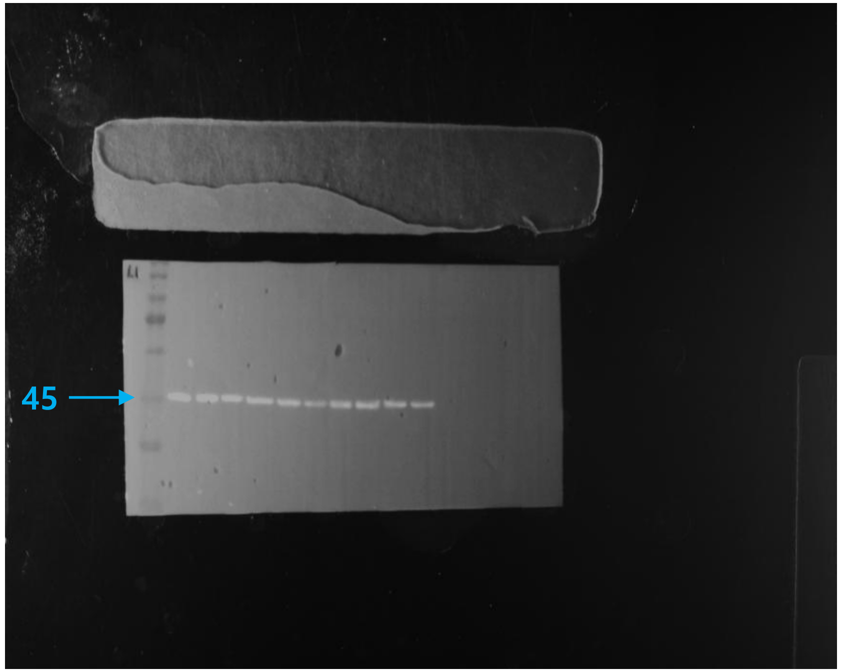

Figure4 B → HSP70

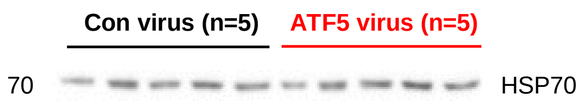

Sample bind

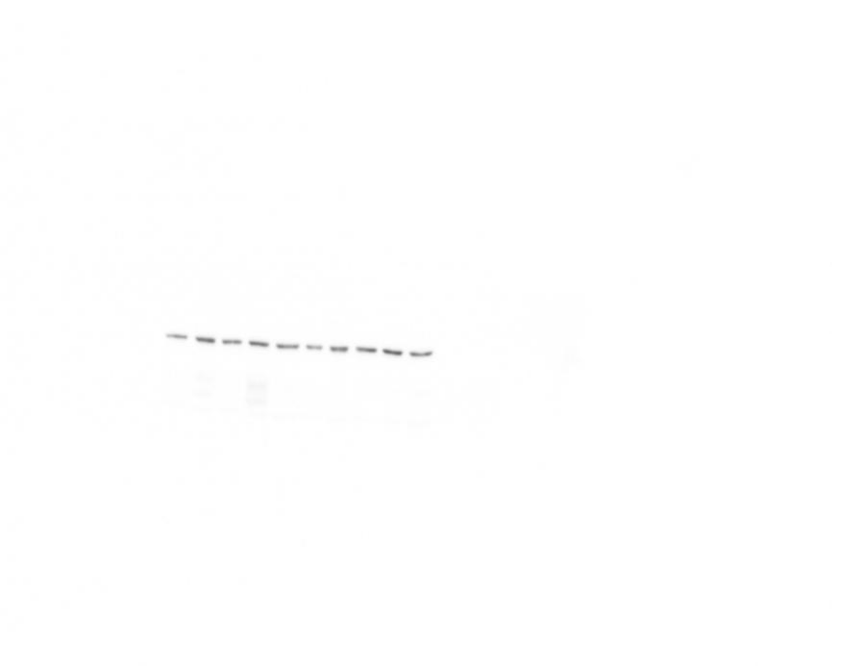

Protein marker

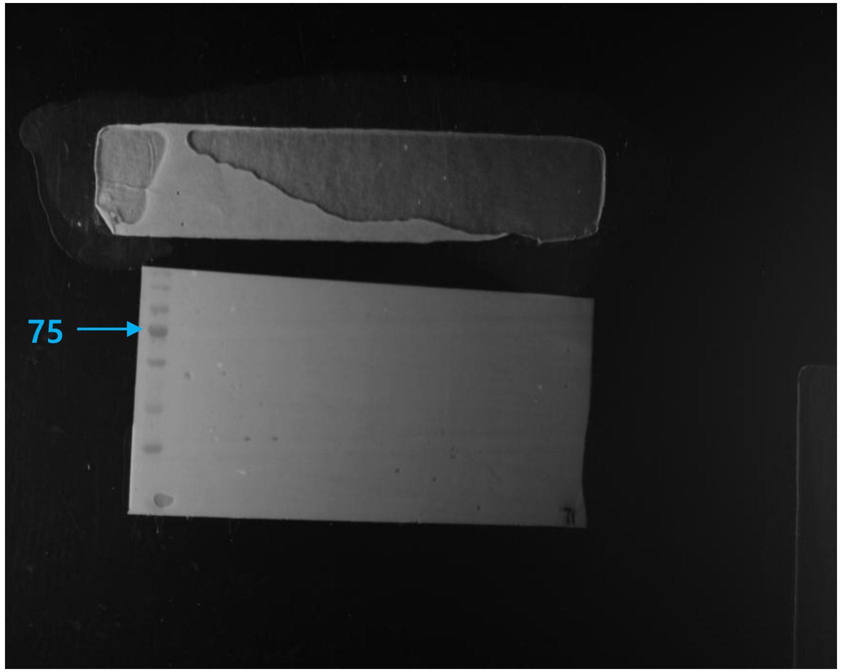

Merge

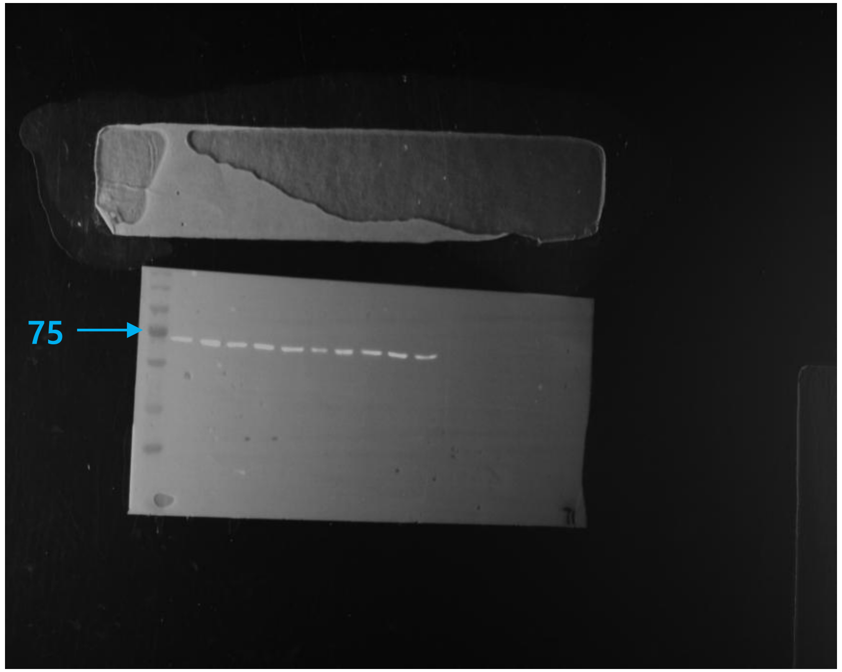

Figure4 B → β-actin of HSP70

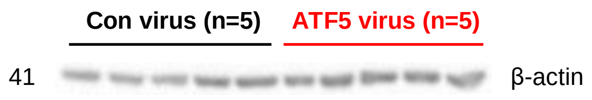

Sample bind

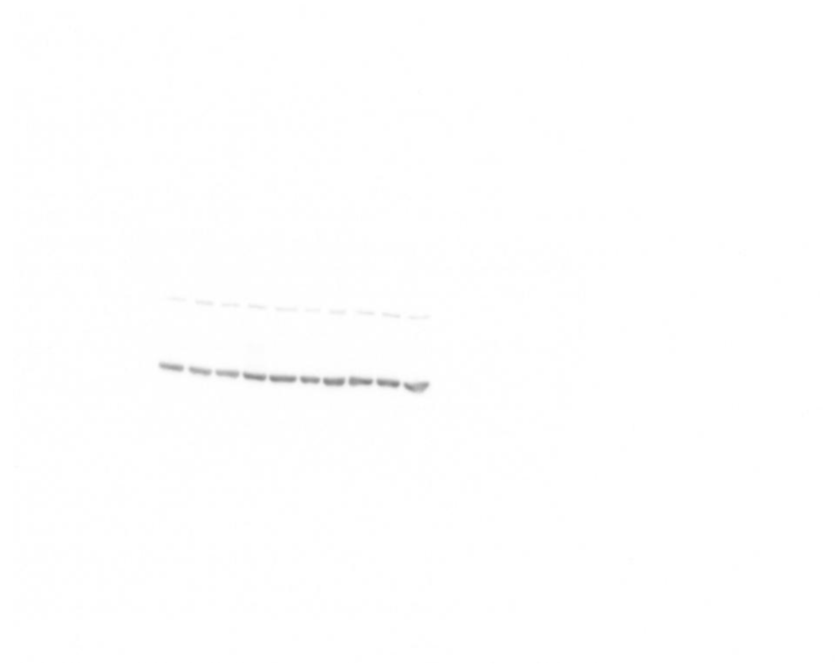

Protein marker

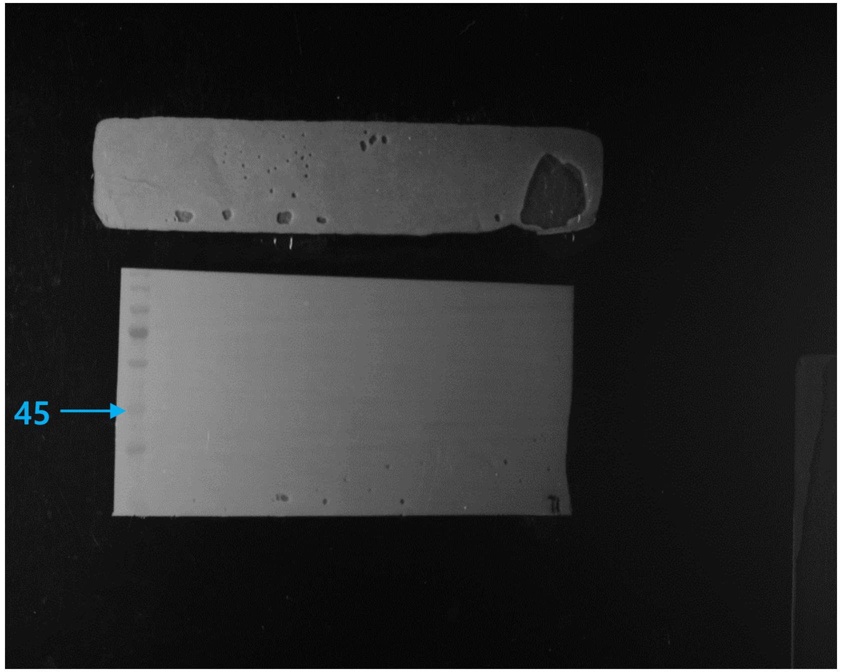

Merge

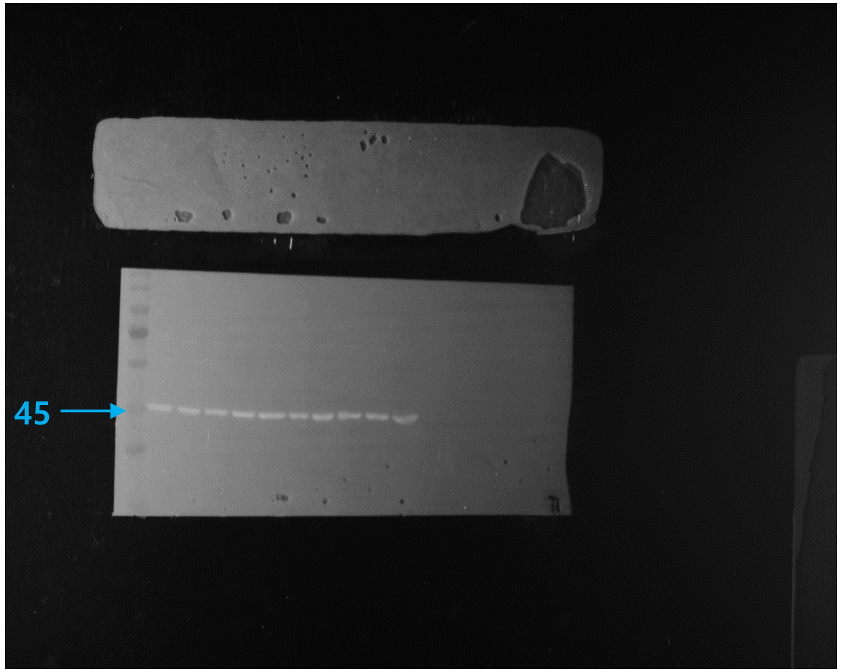

Figure4 B → HSP60

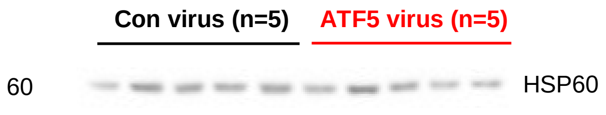

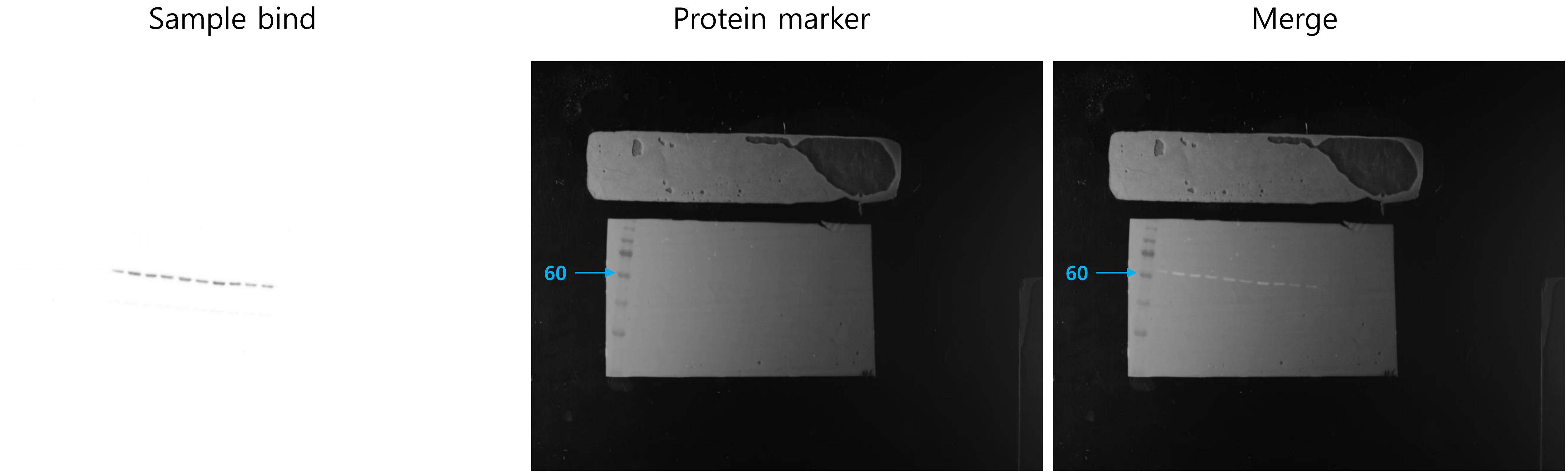

Figure4 B → β-actin of HSP60

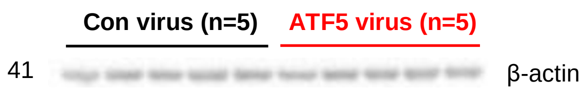

Sample bind

Protein marker

Merge

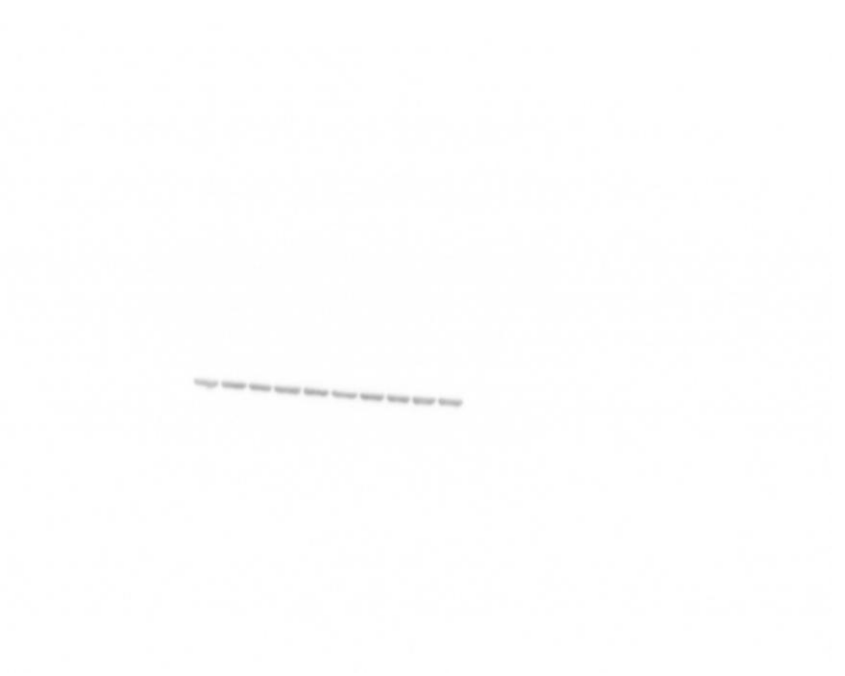

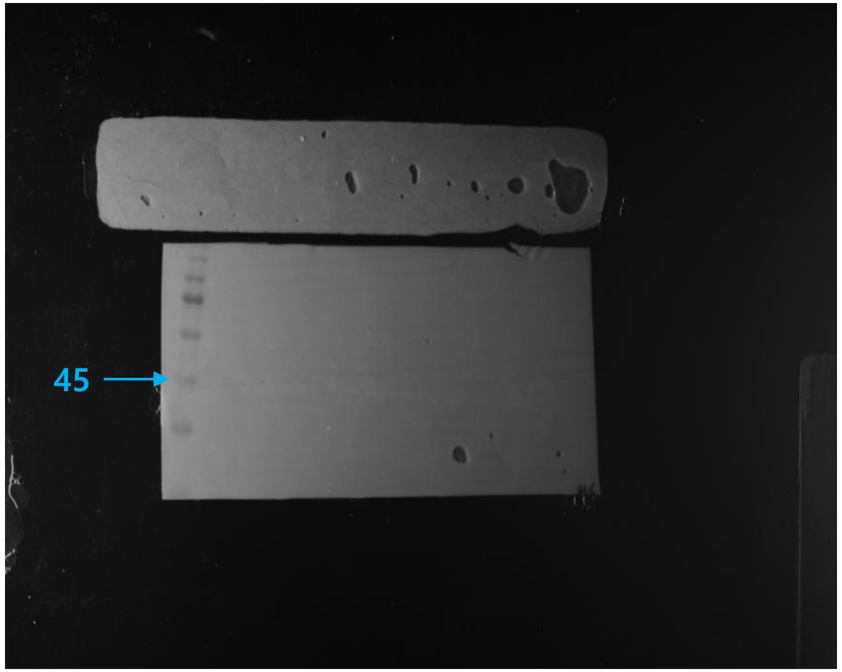

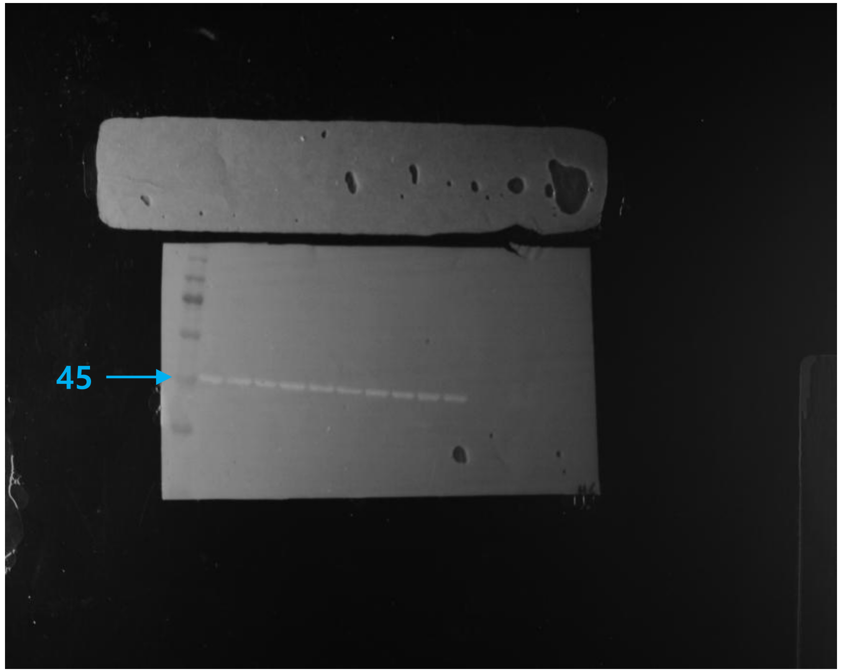

Figure4 B → ATF5

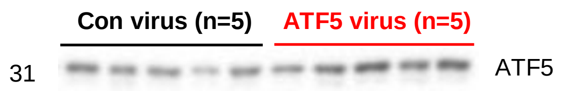

Sample bind

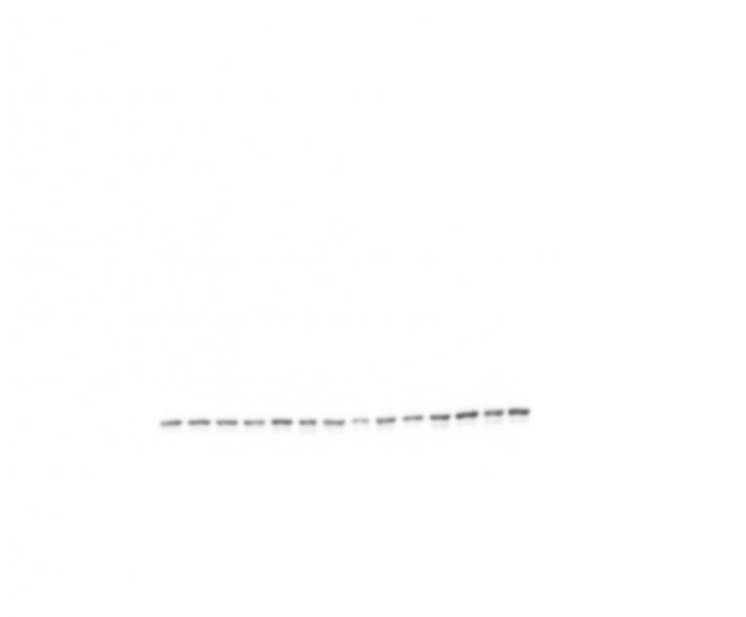

Protein marker

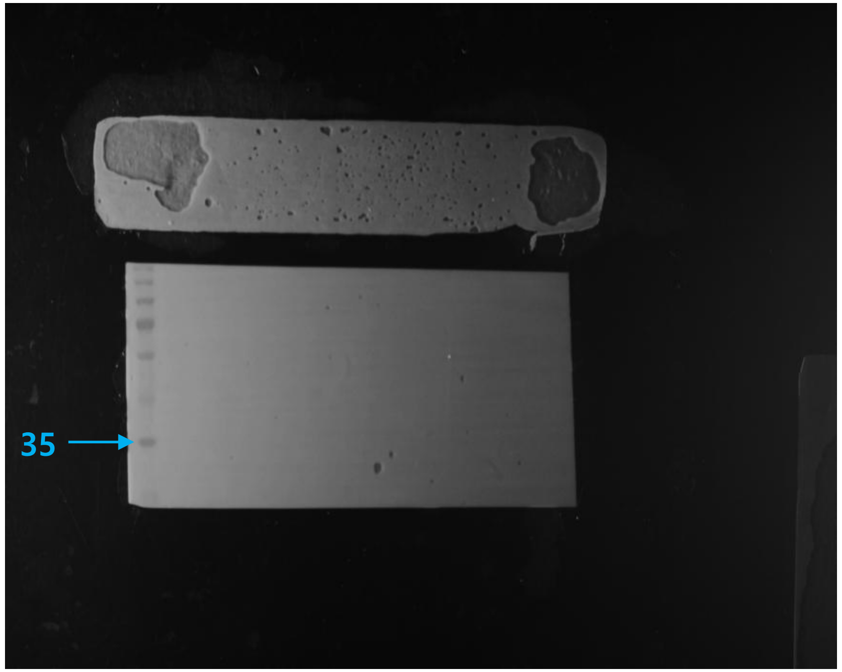

Merge

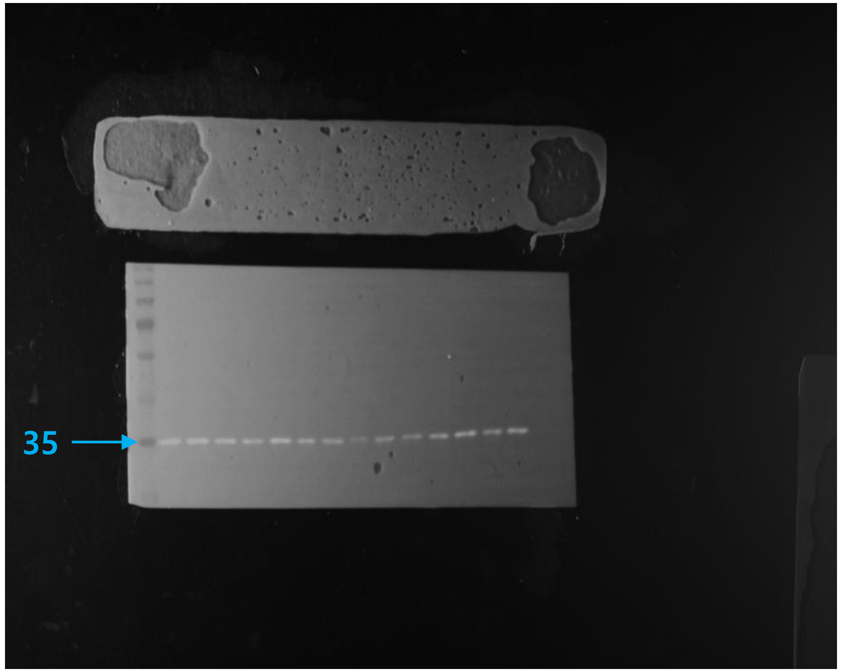

Emx1-cre mice: left 4 samples
Con virus: middle 5 samples
ATF5 virus: right 5 samples

Figure4 B → β-actin of ATF5

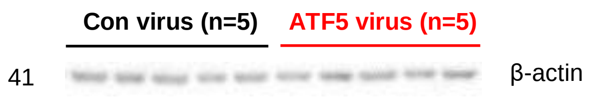

Sample bind

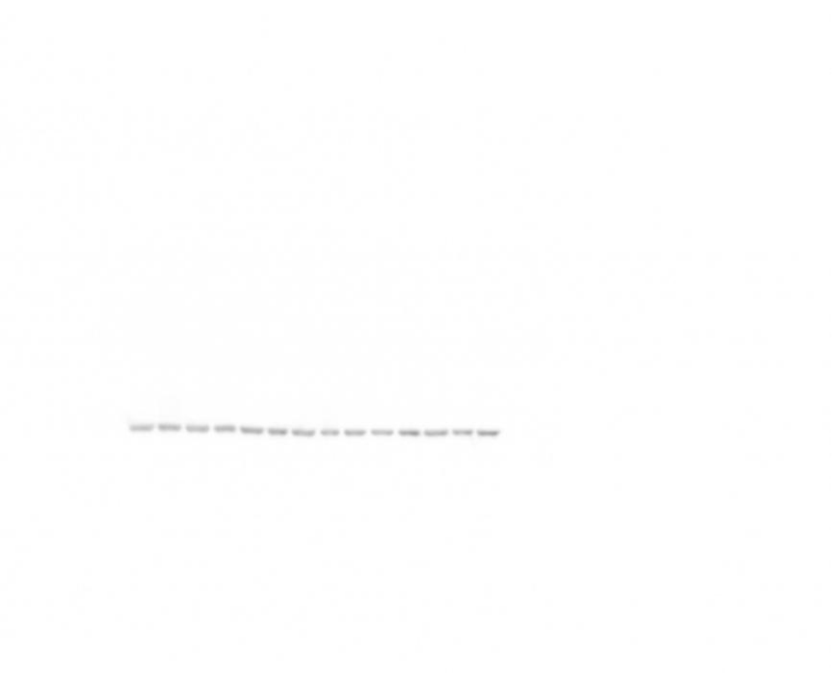

Protein marker

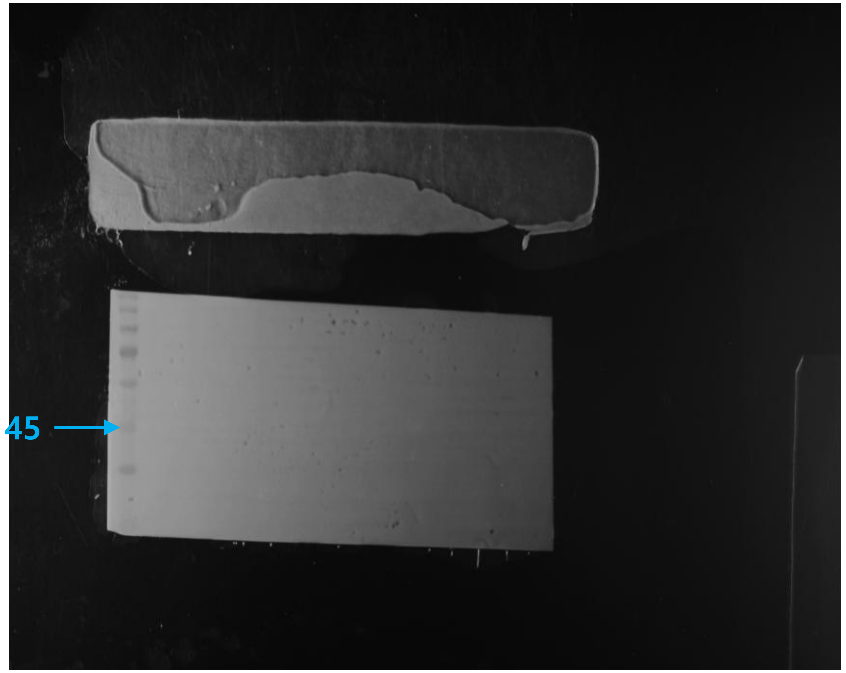

Merge

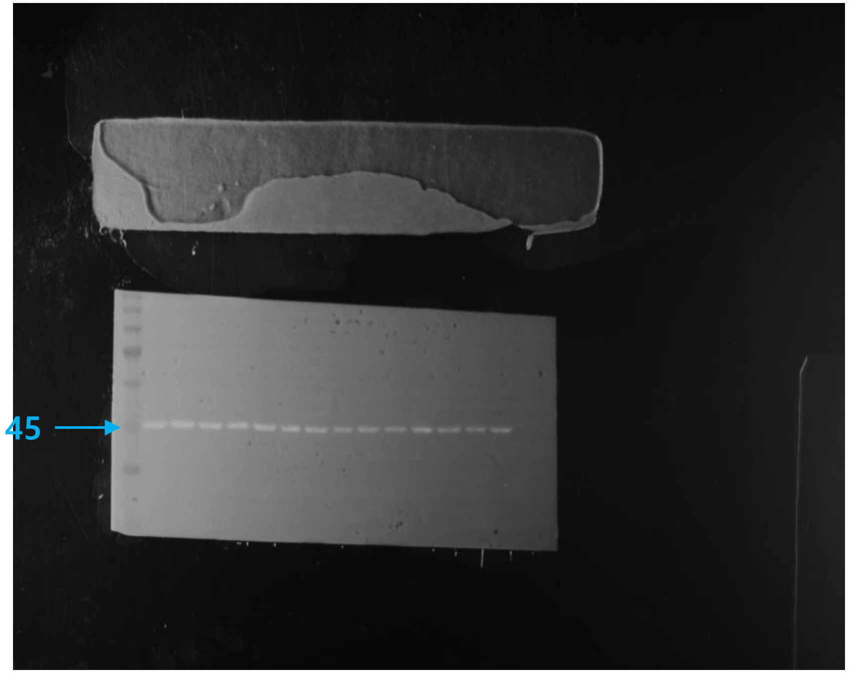

Emx1-cre mice: left 4 samples
Con virus: middle 5 samples
ATF5 virus: right 5 samples

Figure4 B → CLPP

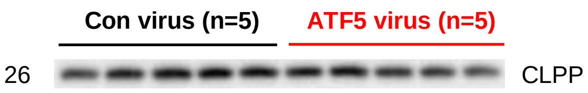

Sample bind

Protein marker

Merge

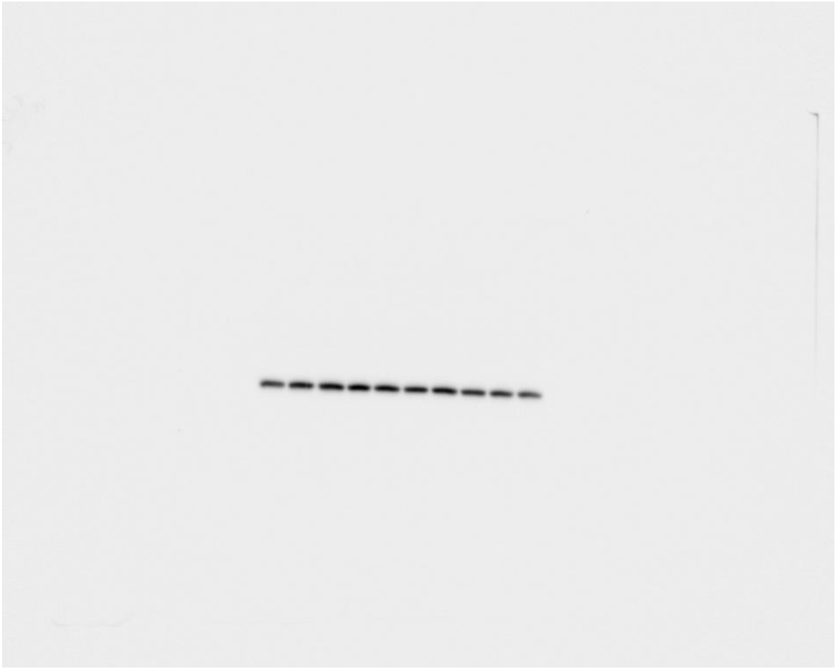

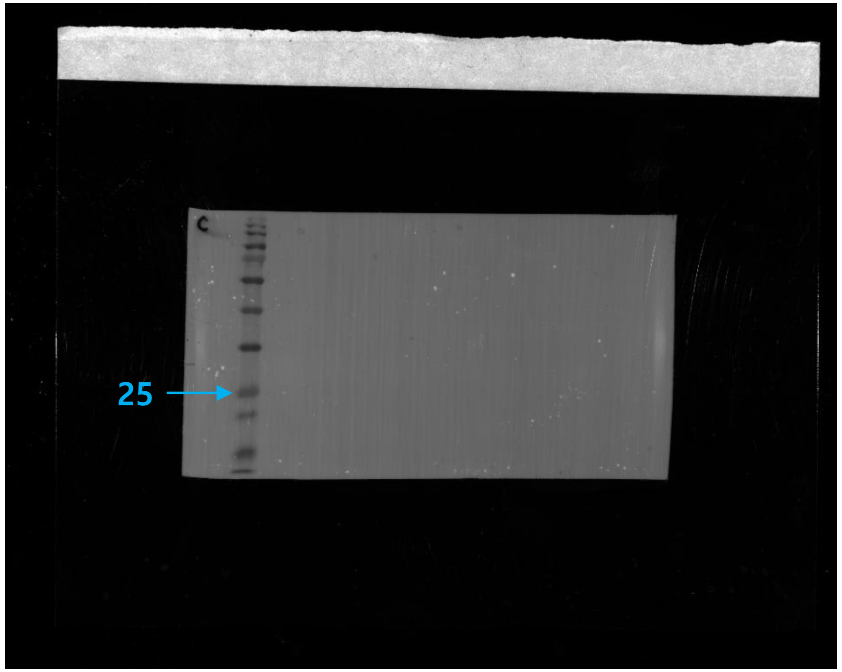

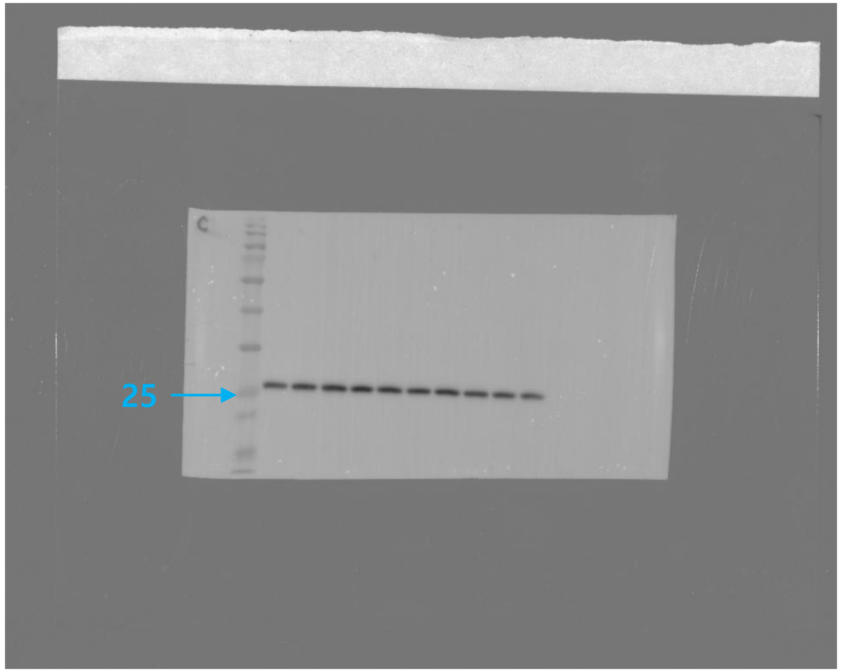

Figure4 B → β-actin of CLPP

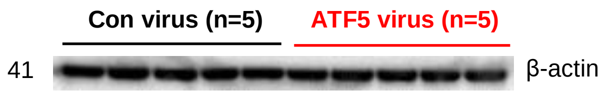

Sample bind

Protein marker

Merge

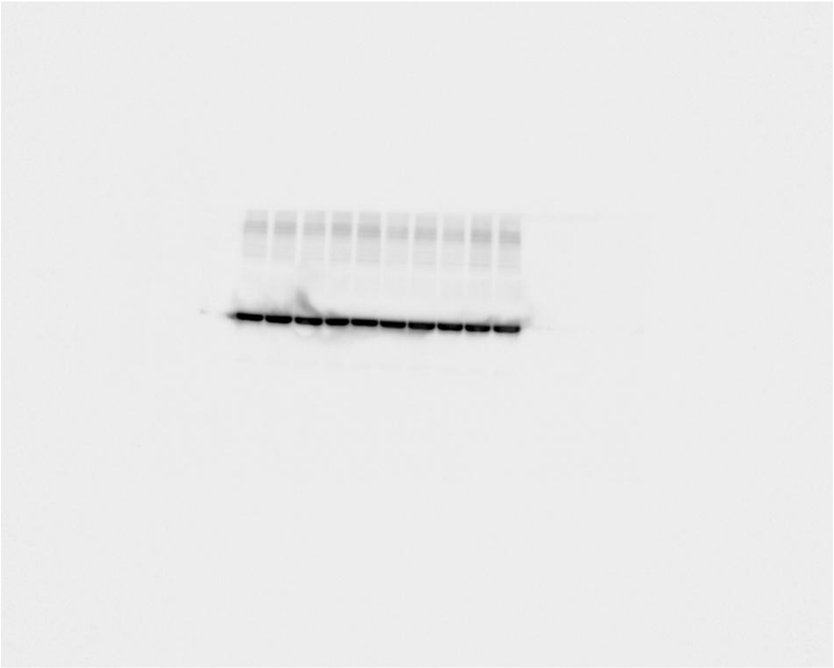

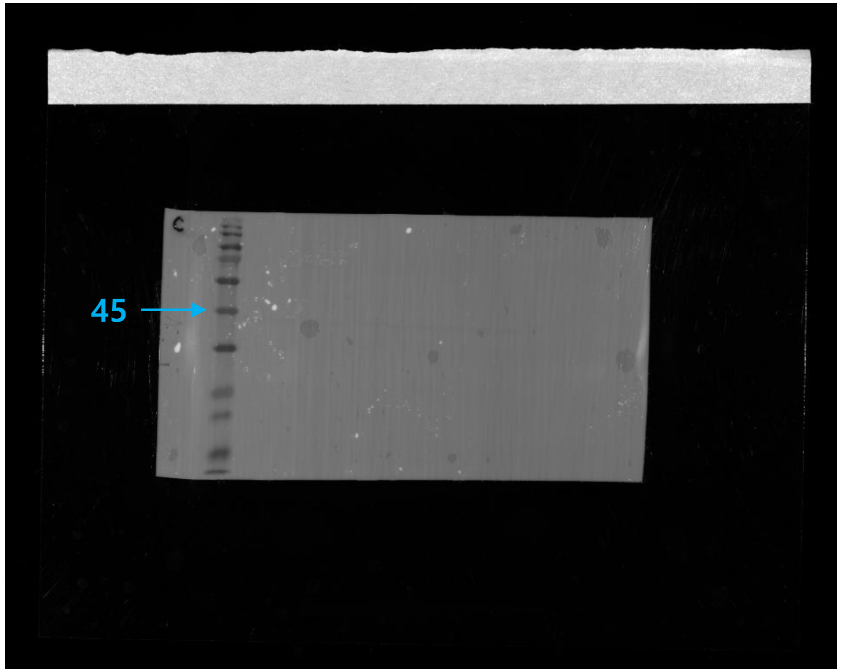

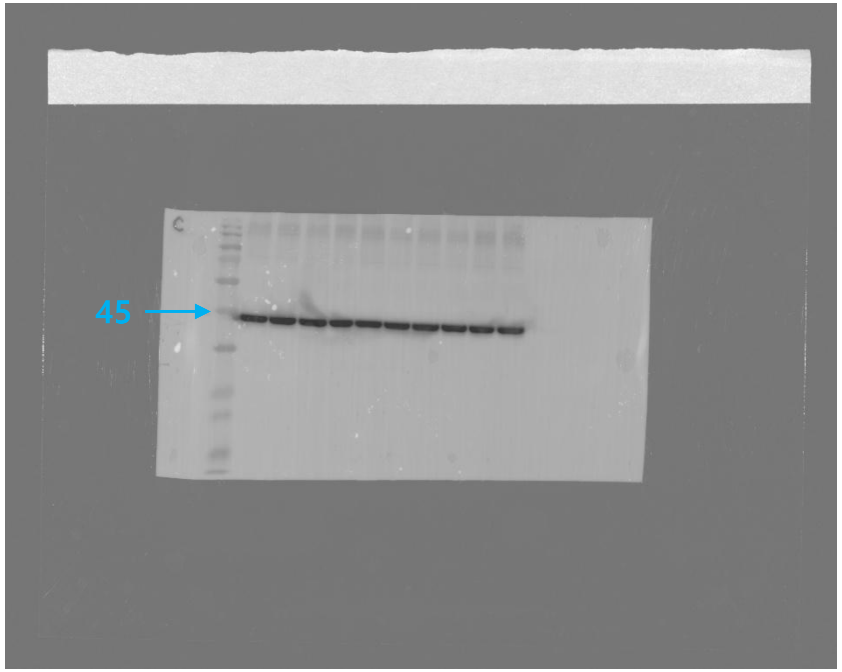

Figure5 B → ATF5

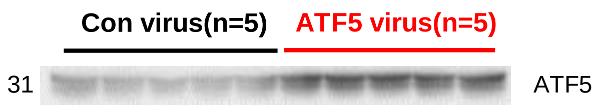

Sample bind

Protein marker

Merge

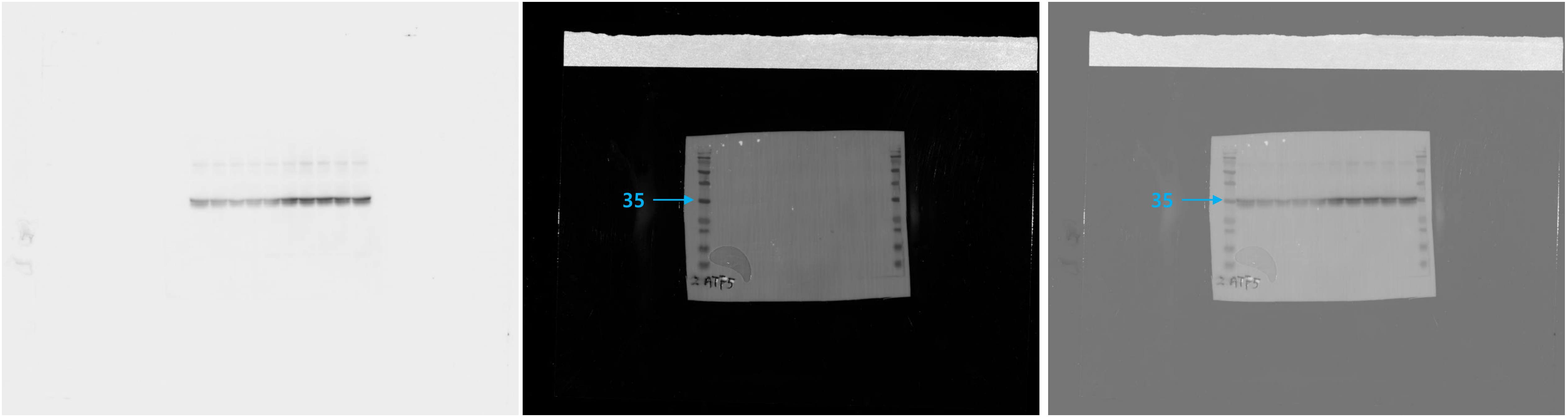

Figure5 B → β-actin of ATF5

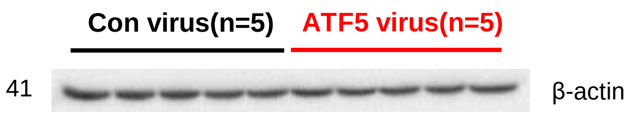

Sample bind

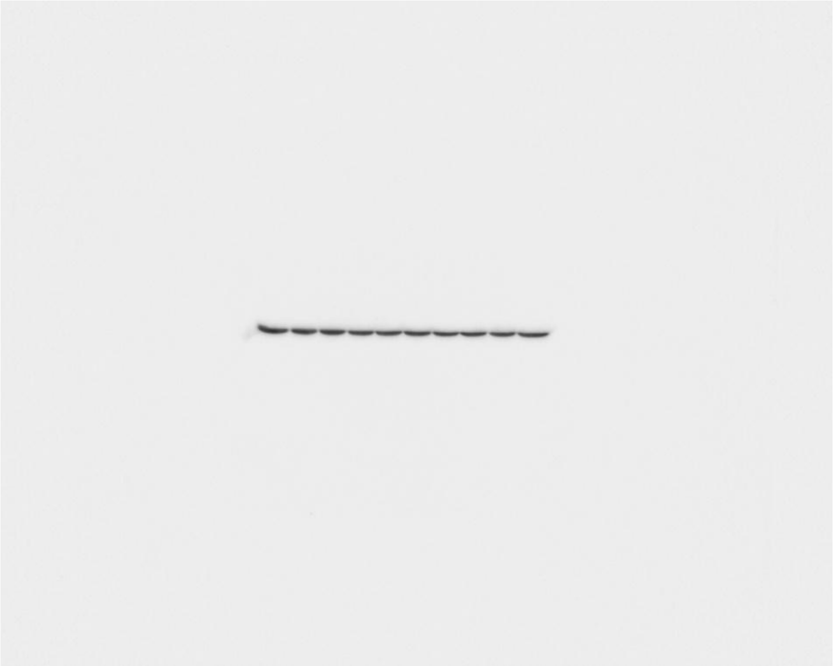

Protein marker

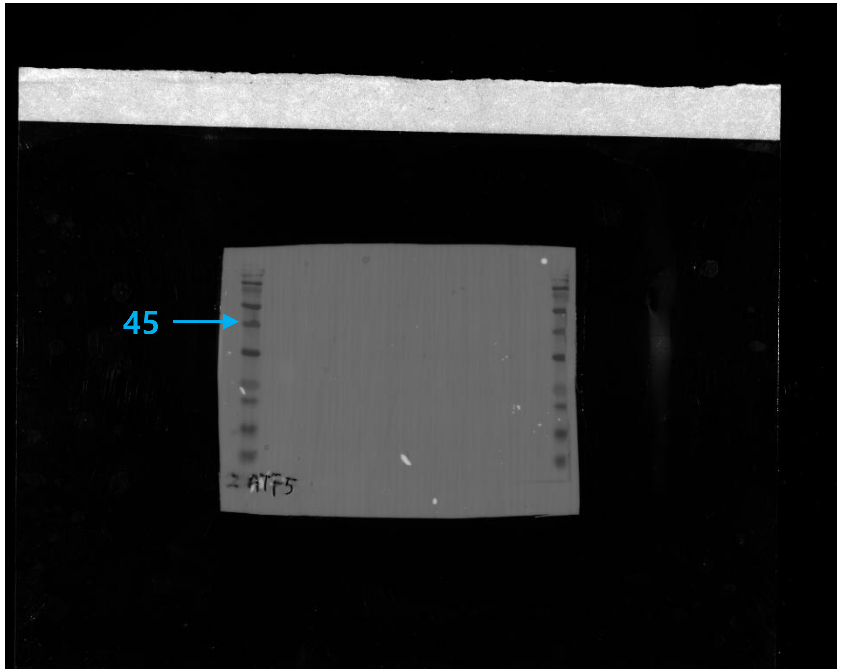

Merge

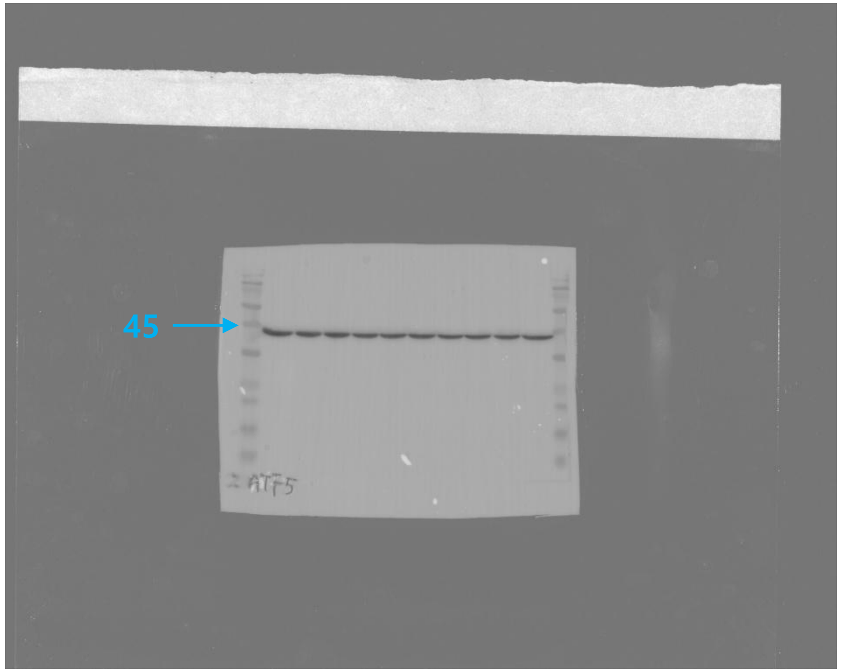

Figure6 E→ LONP1

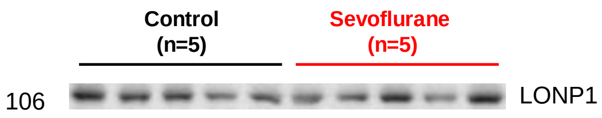

Sample bind

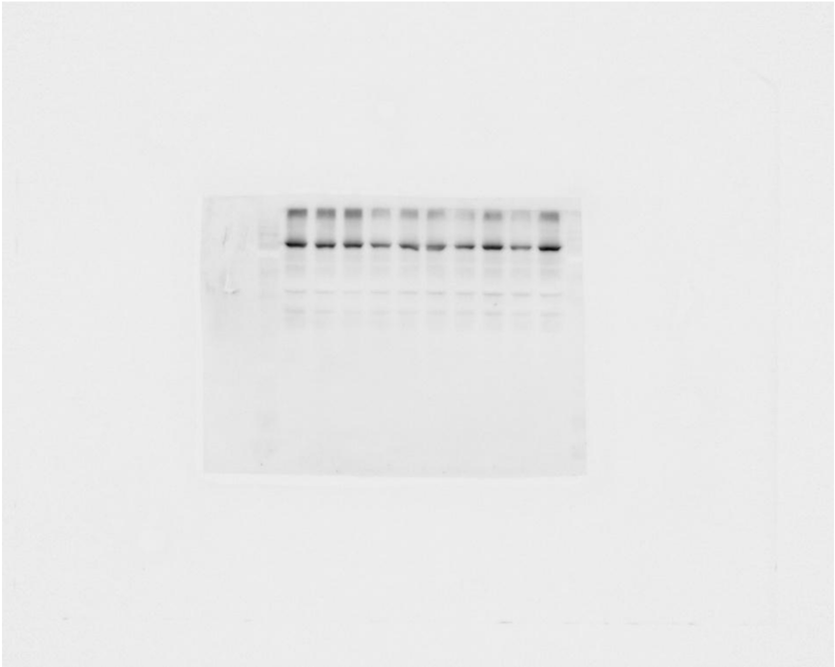

Protein marker

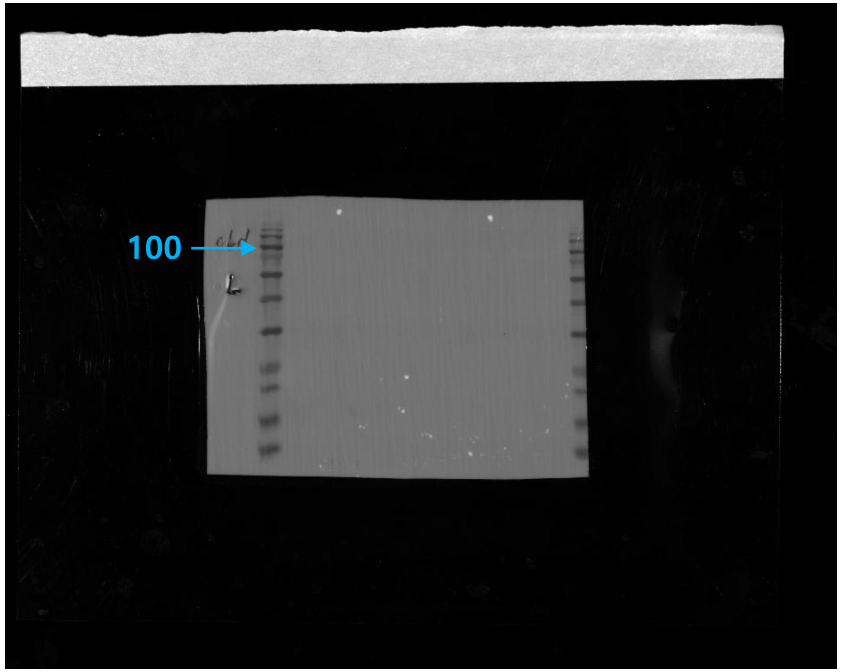

Merge

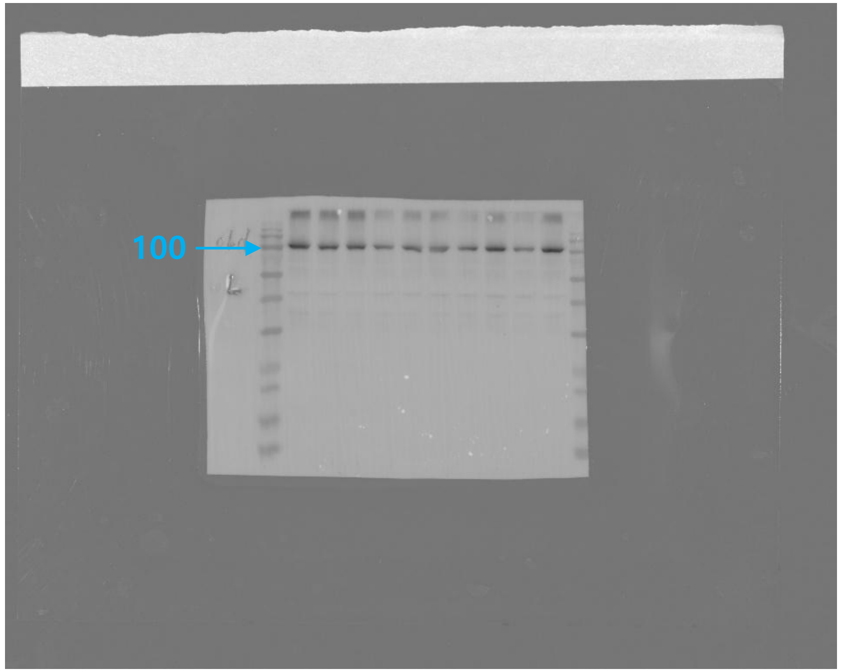

Figure6 E→ β-actin of LONP1

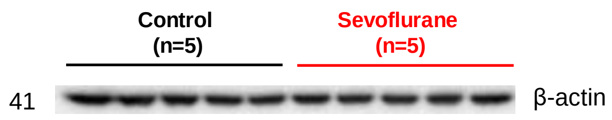

Sample bind

Protein marker

Merge

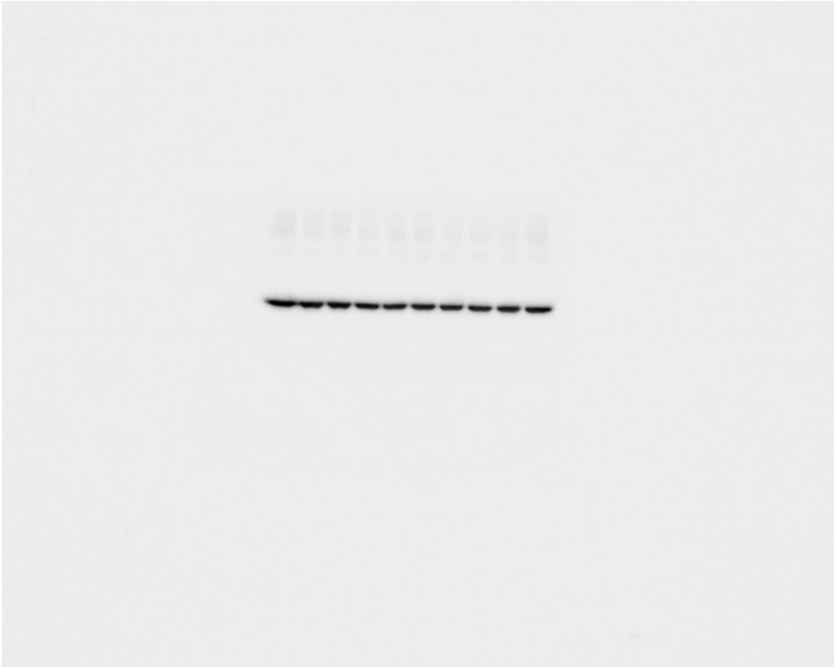

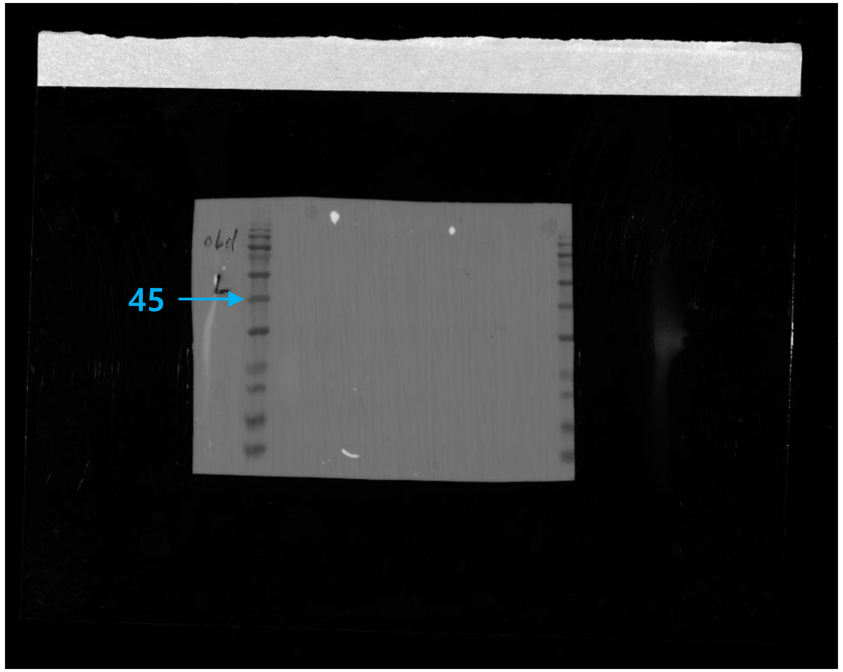

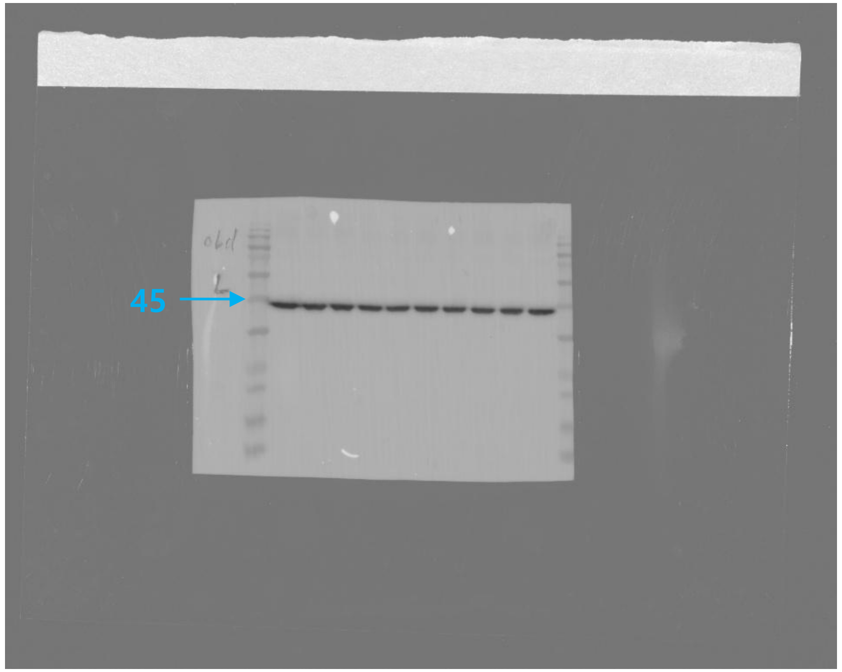

Figure6 E→ HSP70

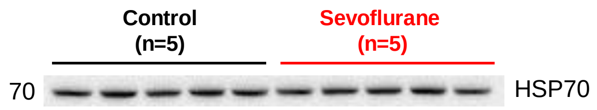

Sample bind

Protein marker

Merge

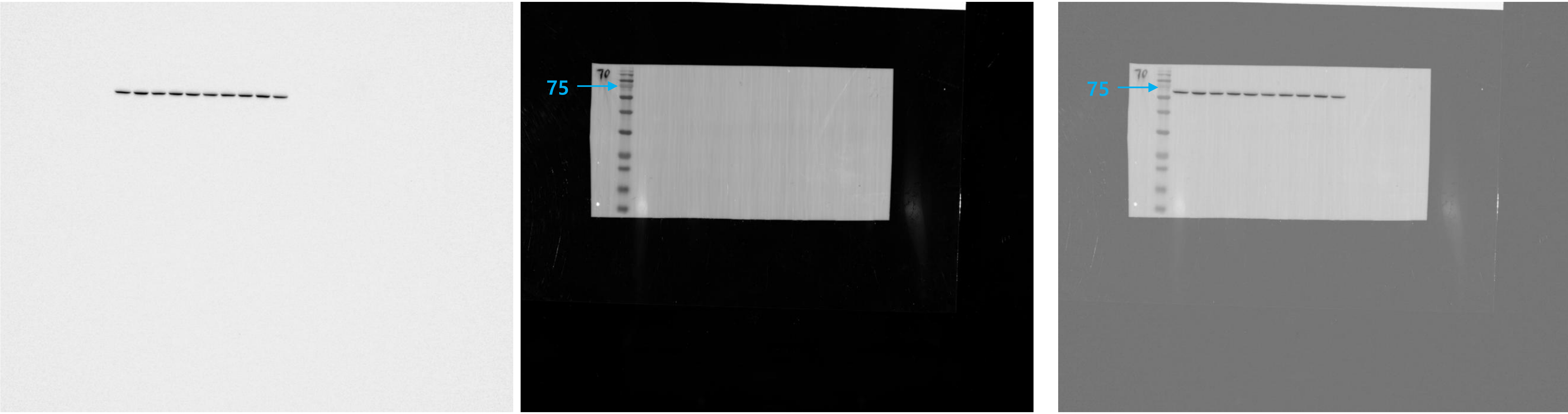

Figure6 E→ β-actin of HSP70

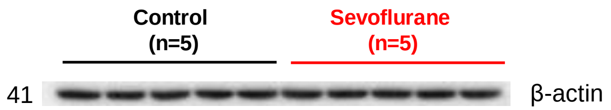

Sample bind

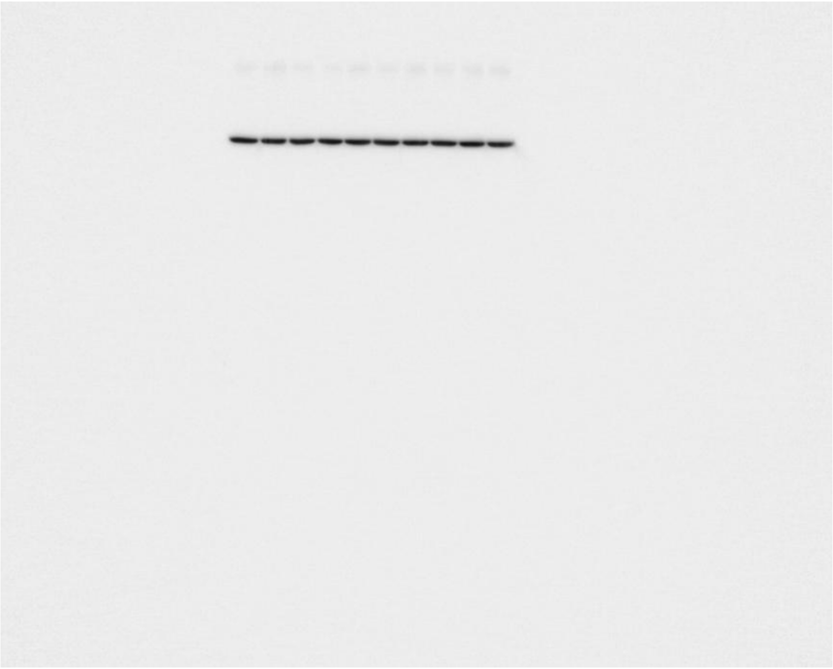

Protein marker

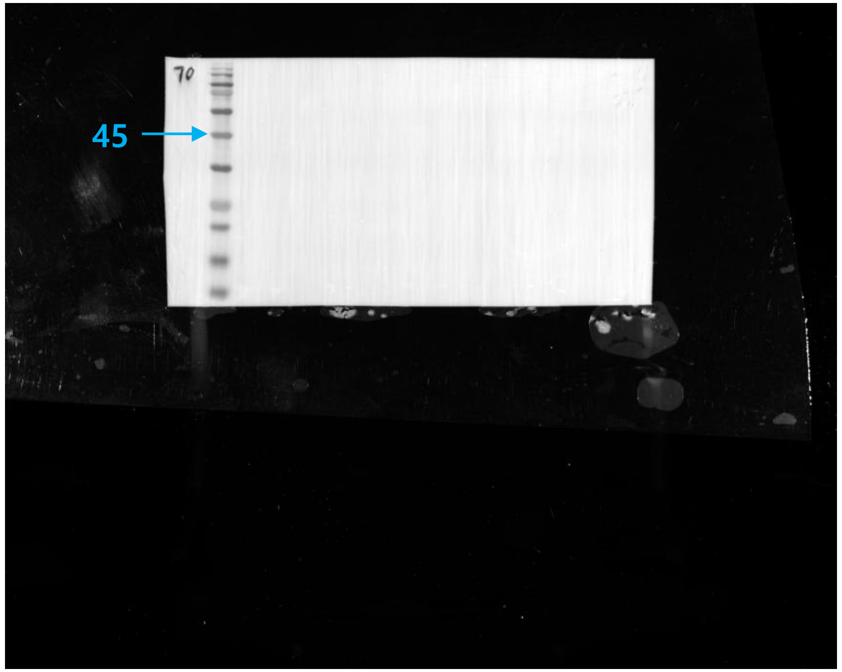

Merge

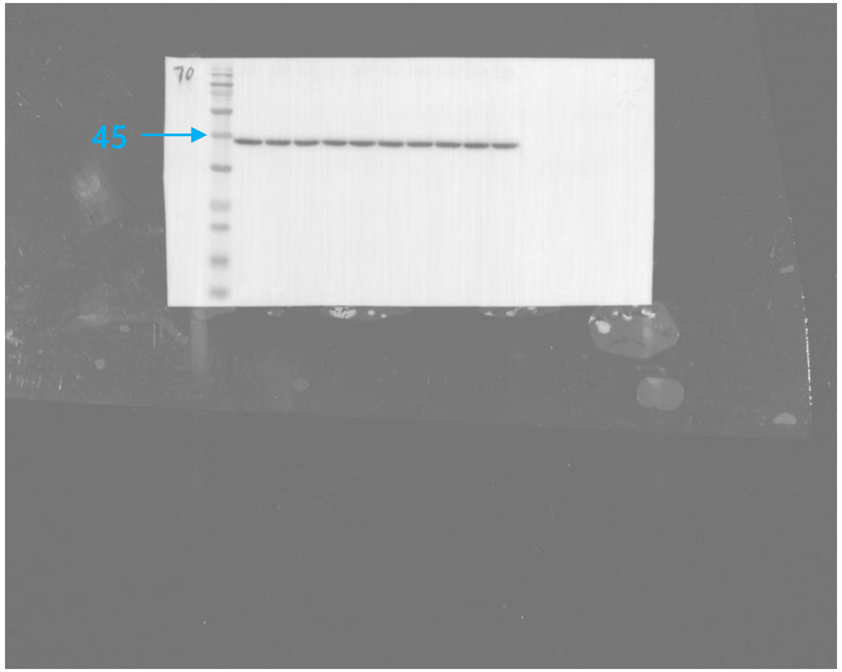

Figure6 E→ HSP60

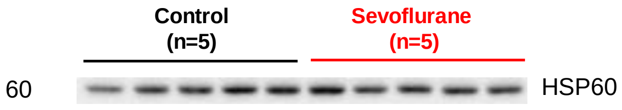

Sample bind

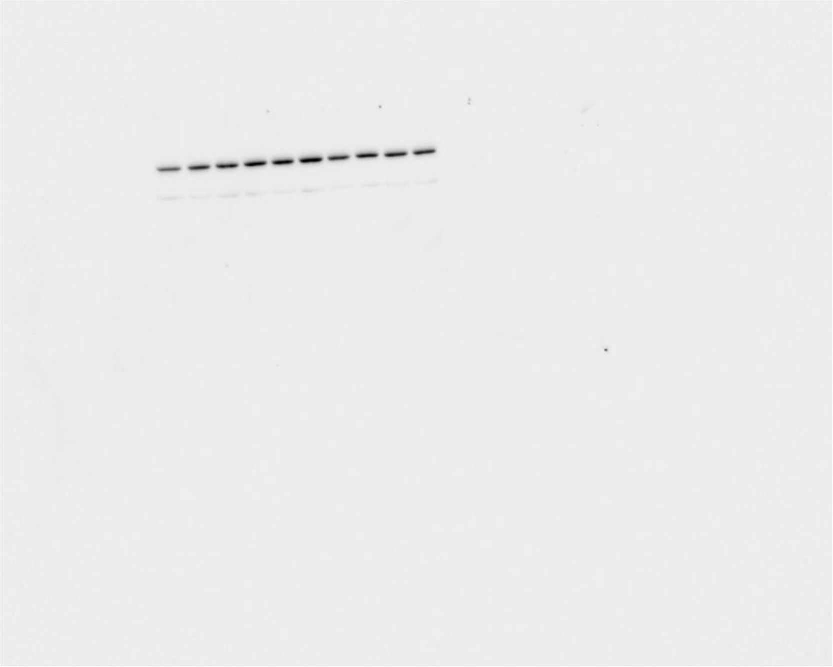

Protein marker

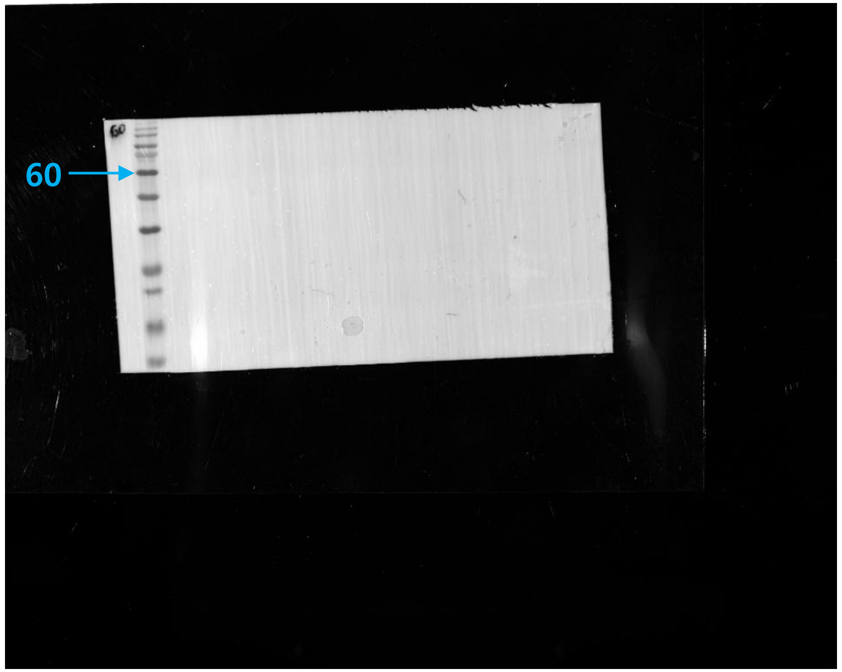

Merge

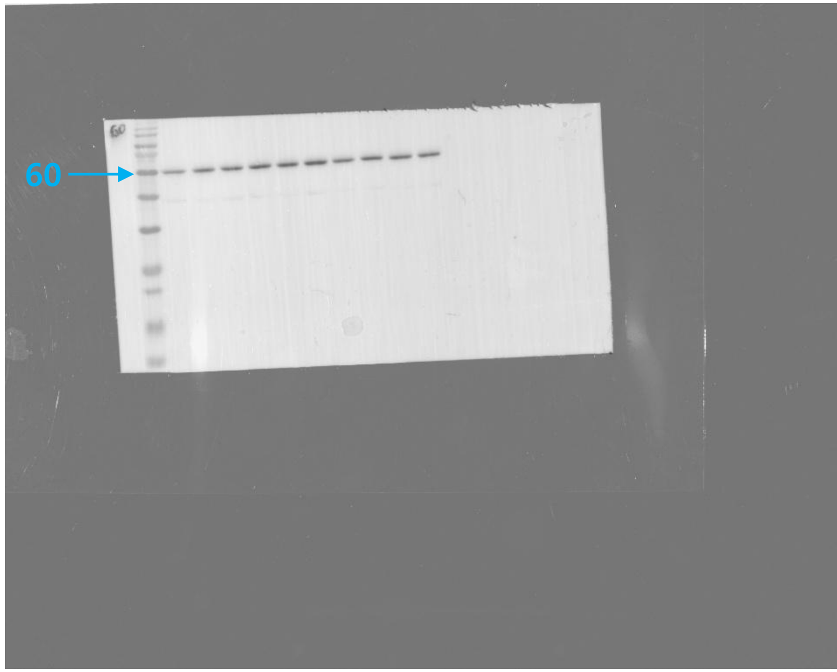

Figure6 E→ β-actin of HSP60

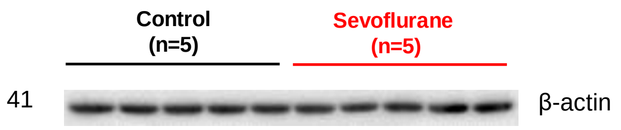

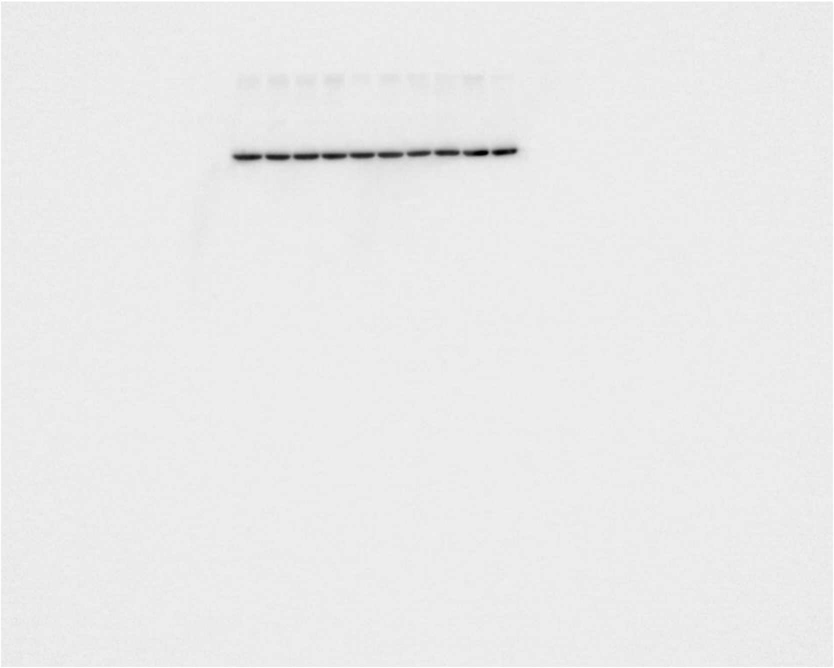

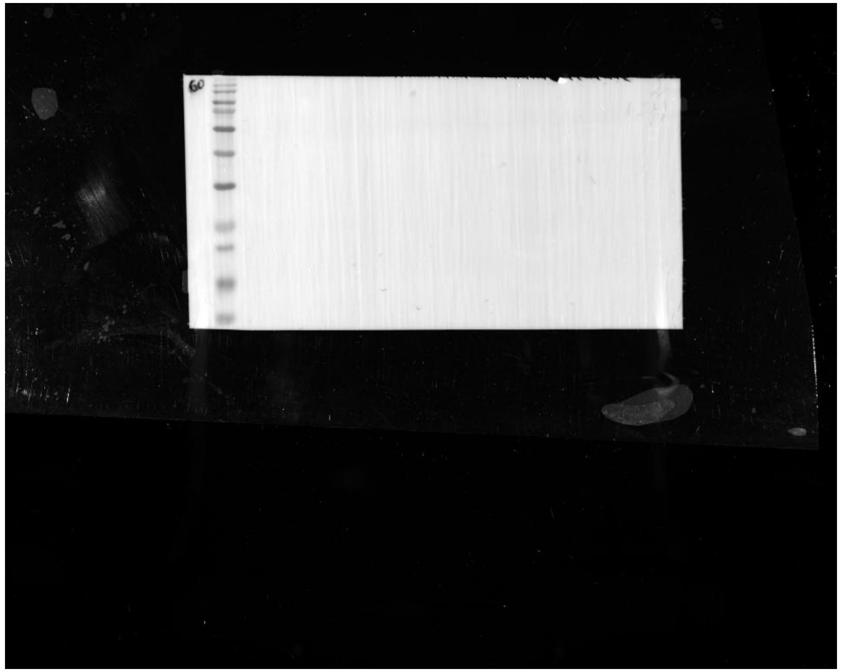

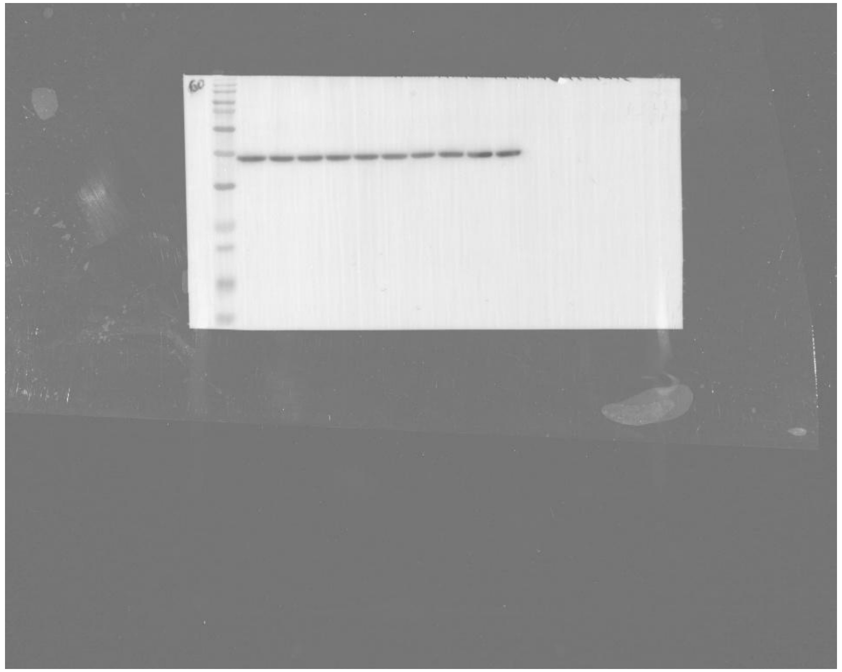

Figure6 E→ ATF5

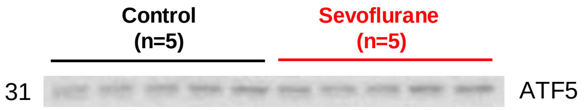

Sample bind

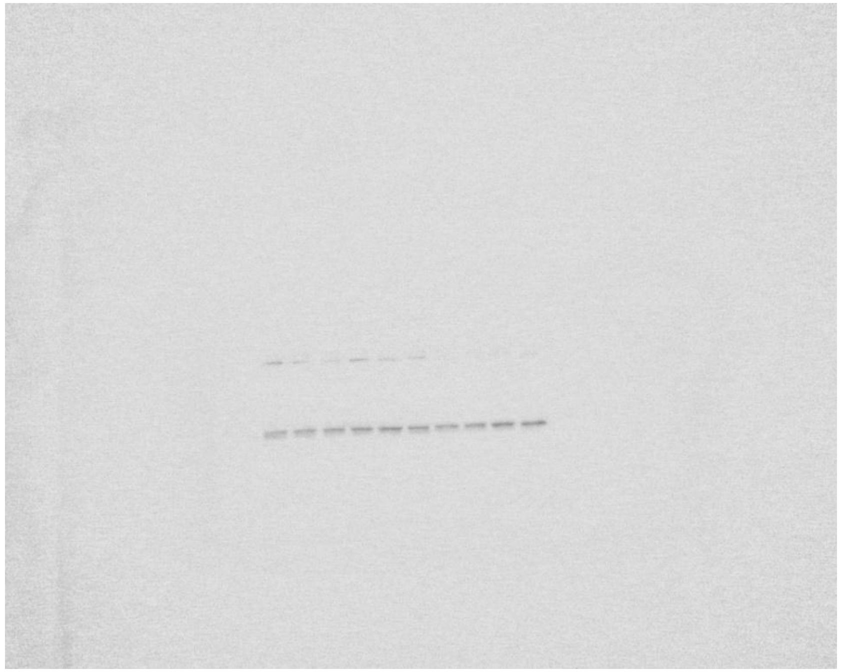

Protein marker

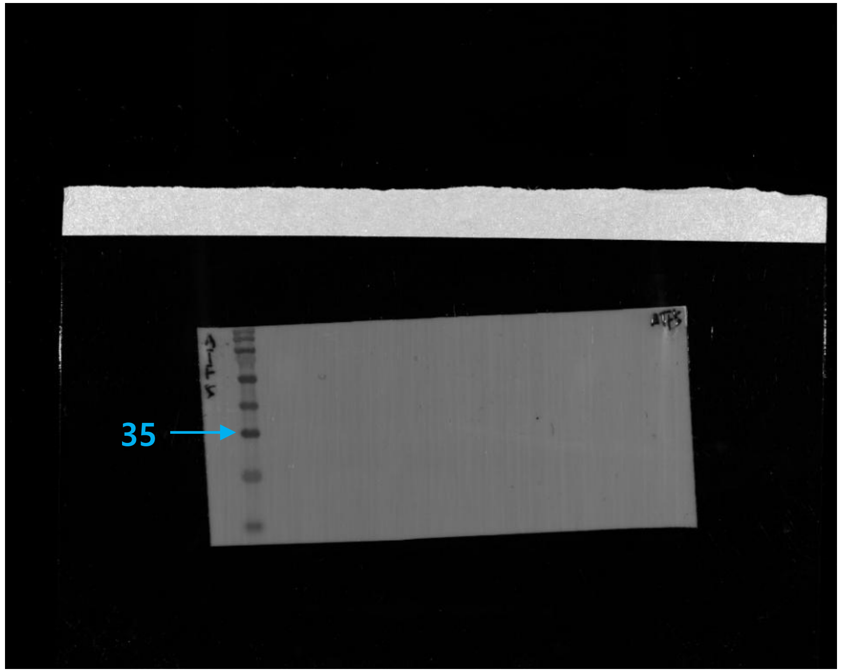

Merge

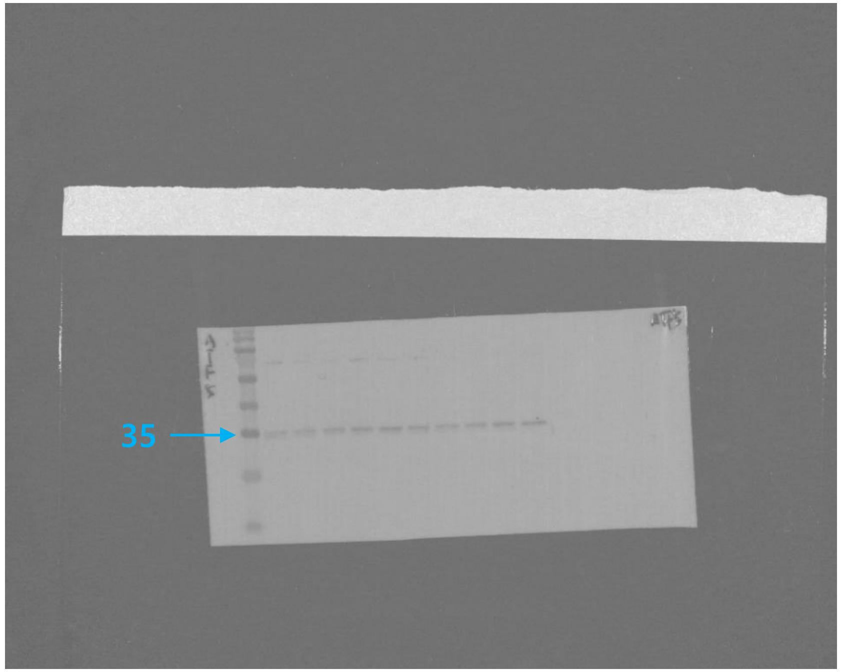

Figure6 E→ β-actin of ATF5

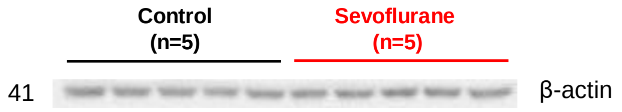

Sample bind

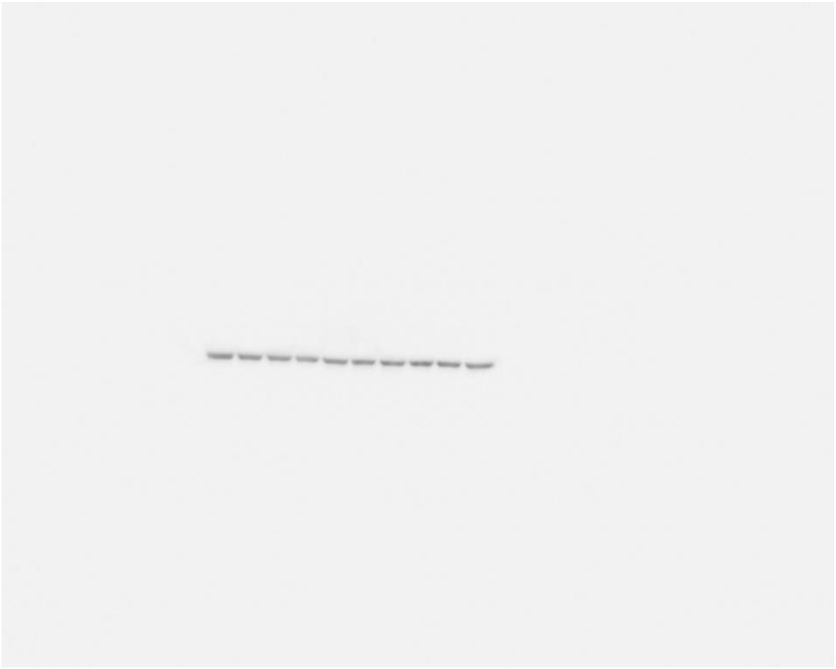

Protein marker

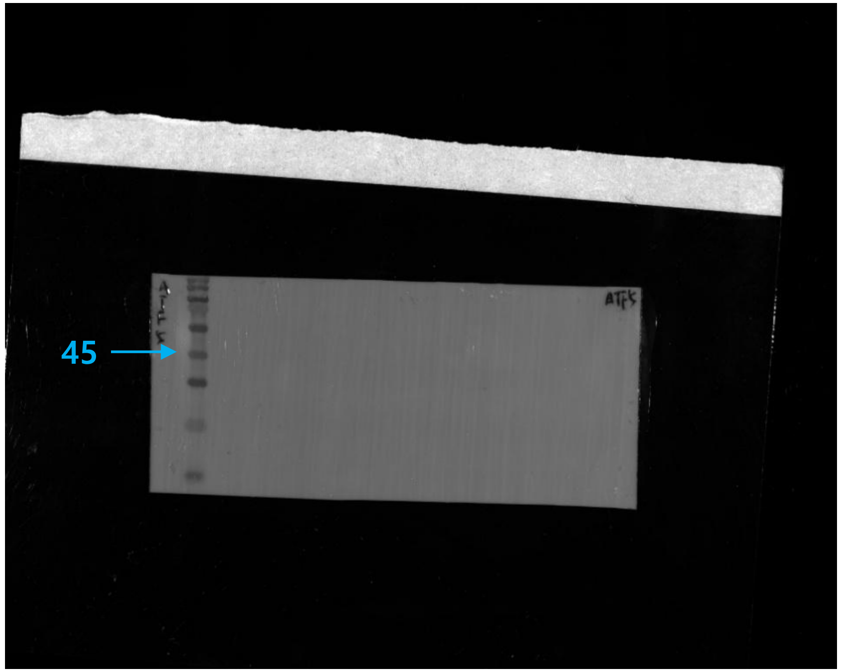

Merge

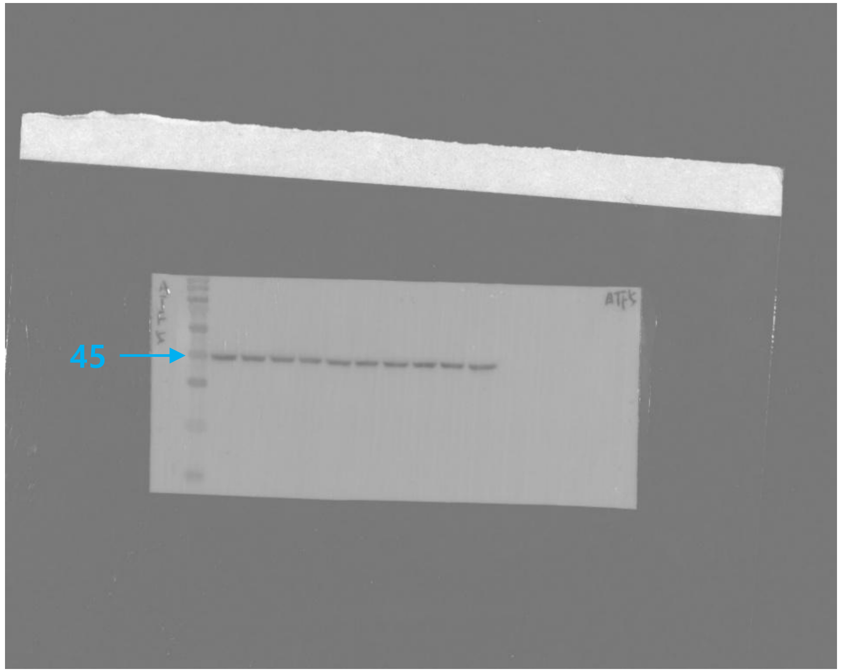

Figure6 E→ CLPP

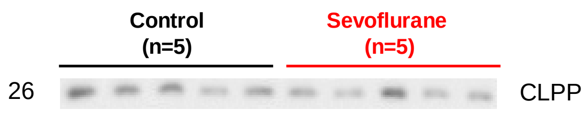

Sample bind

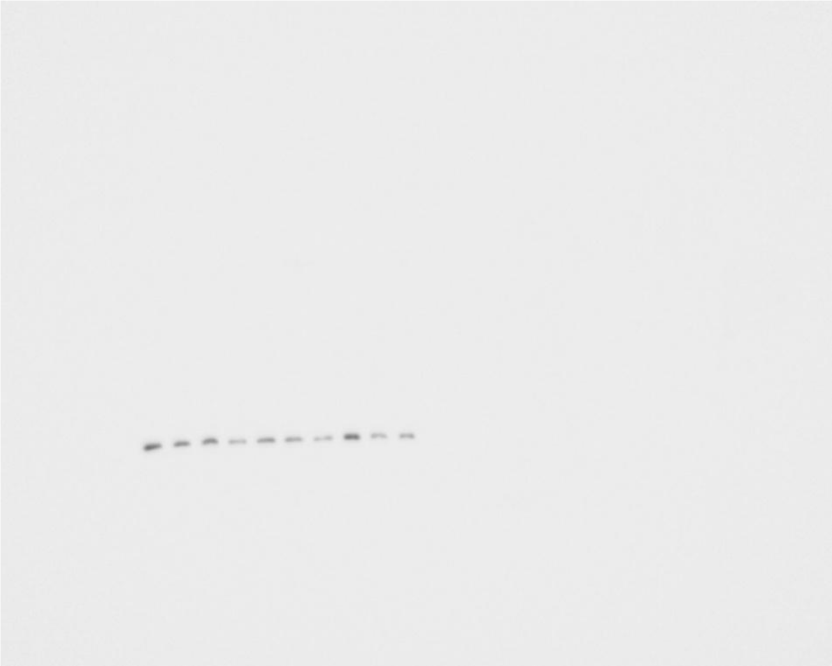

Protein marker

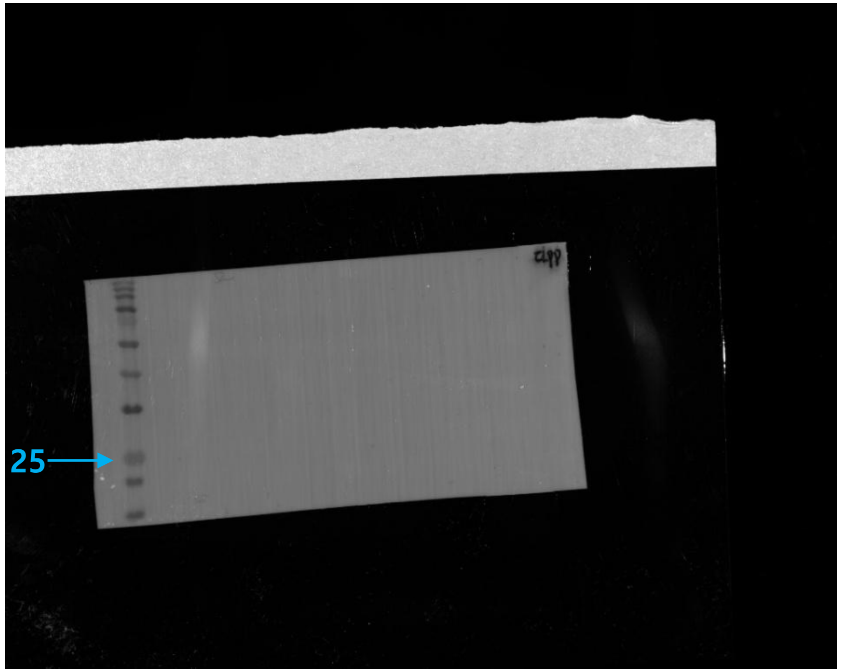

Merge

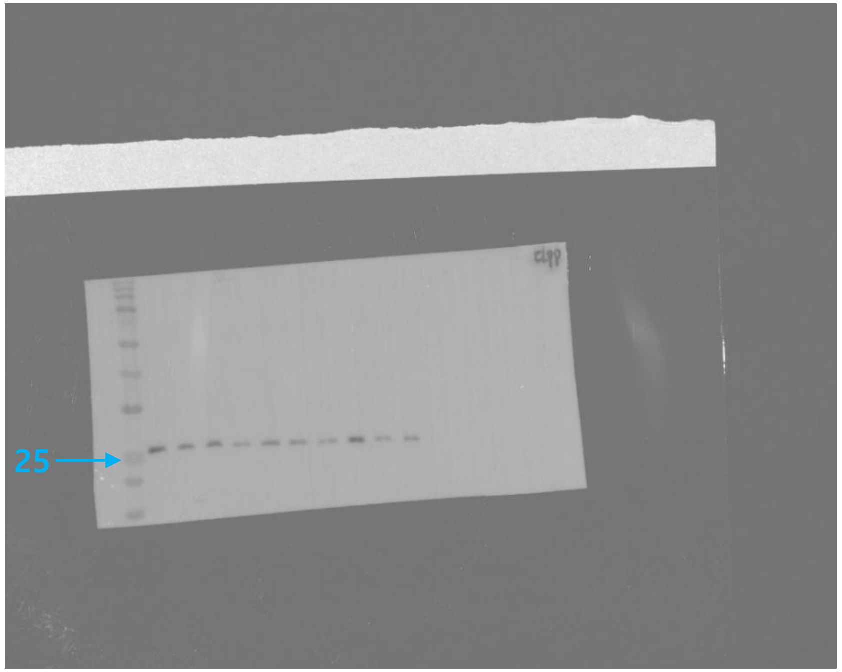

Figure6 E→ β-actin of CLPP

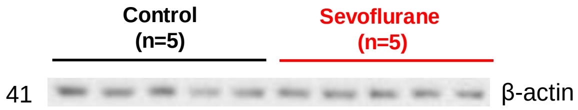

Sample bind

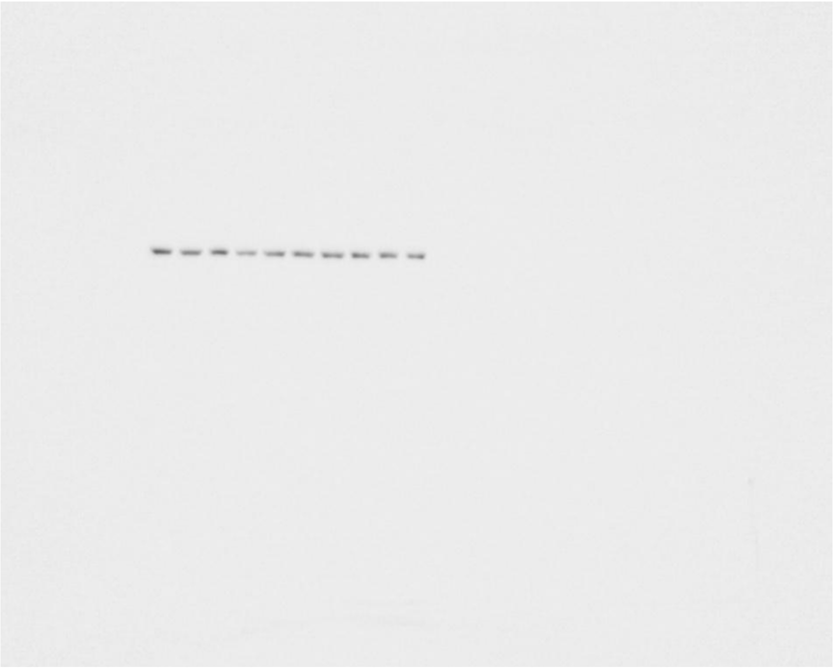

Protein marker

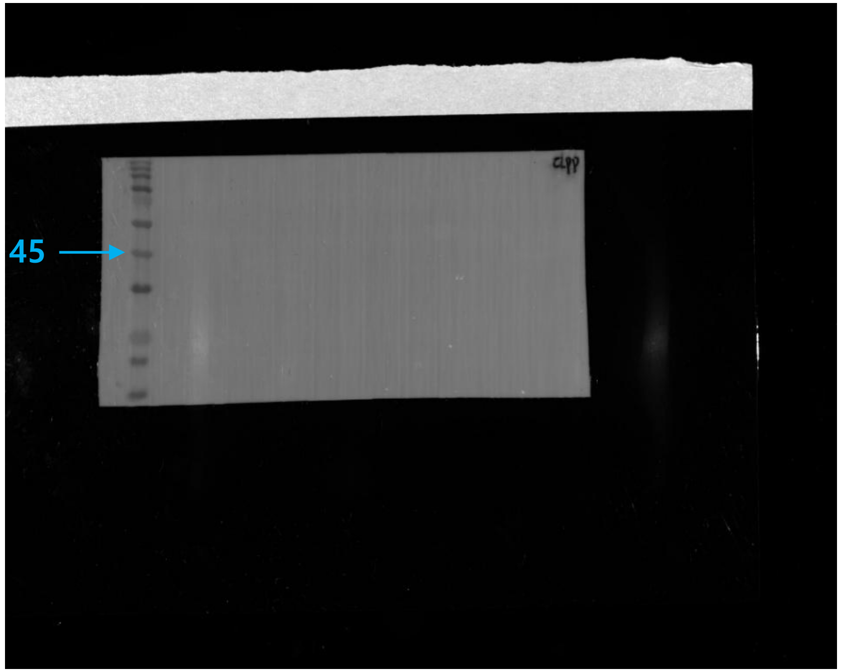

Merge

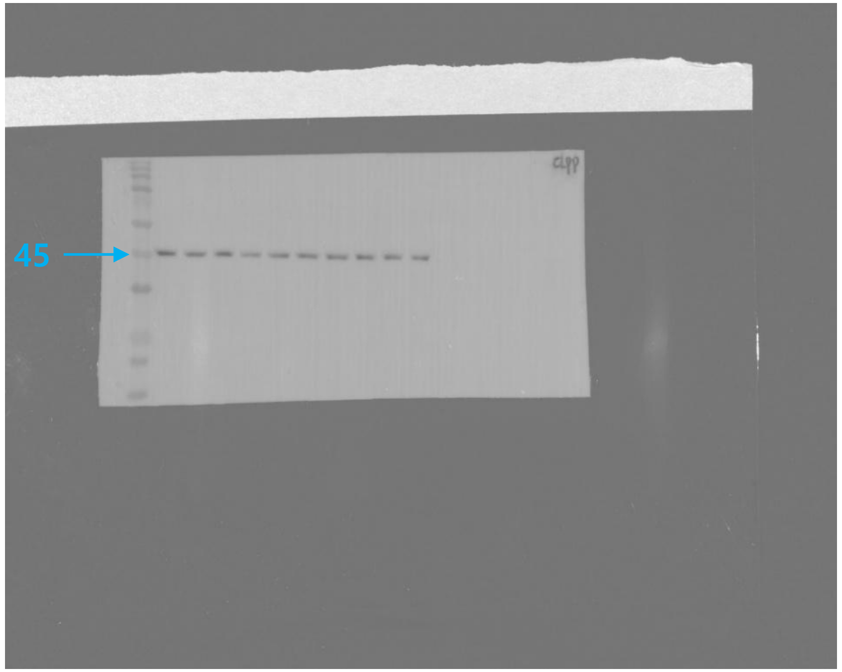

Supplement: Supplementary file 3 — Supporting Information [file ADVS-13-e17086-s005.pdf]
